# Chemical and Redox Noninnocence of Pentane-2,4-dione Bis(*S*-methylisothiosemicarbazone) in Cobalt Complexes and Their Application in Wacker-Type Oxidation

**DOI:** 10.1021/jacsau.4c00005

**Published:** 2024-03-12

**Authors:** Vincent Porte, Miljan N. M. Milunovic, Ulrich Knof, Thomas Leischner, Tobias Danzl, Daniel Kaiser, Tim Gruene, Michal Zalibera, Ingrid Jelemenska, Lukas Bucinsky, Sergio A. V. Jannuzzi, Serena DeBeer, Ghenadie Novitchi, Nuno Maulide, Vladimir B. Arion

**Affiliations:** † University of Vienna, Institute of Organic Chemistry, Währinger Strasse 38, A-1090 Vienna, Austria; ‡ University of Vienna, Institute of Inorganic Chemistry, Währinger Strasse 42, A-1090 Vienna, Austria; § Novartis Pharma AG, CH-4056 Basel, Switzerland; ∥ Institute of Physical Chemistry and Chemical Physics, Faculty of Chemical and Food Technology, Slovak University of Technology in Bratislava, Radlinského 9, SK-81237 Bratislava, Slovak Republic; ⊥ Max Planck Institute for Chemical Energy Conversion, Stiftstraße 34-36, 45470 Mülheim an der Ruhr, Germany; # CNRS-LNCMI, 38042 Grenoble Cedex, France

**Keywords:** cobalt, pentane-2,4-dione bis(*S*-methylisothiosemicarbazone), noninnocence, redox, multiproton, ketone, olefin, oxidation

## Abstract

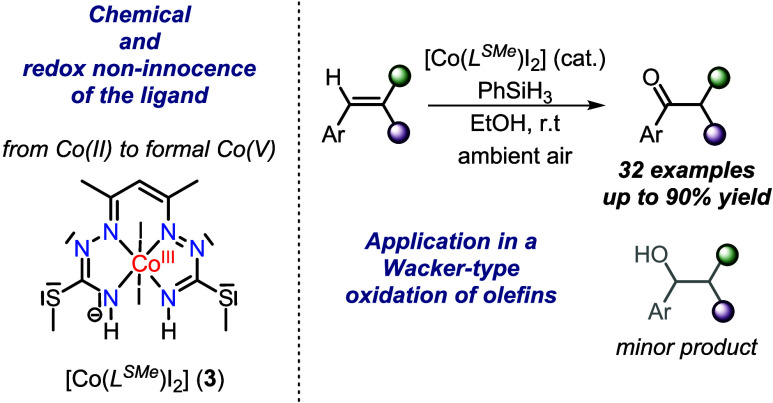

Cobalt complexes with multiproton- and multielectron-responsive ligands are of interest for challenging catalytic transformations. The chemical and redox noninnocence of pentane-2,4-dione bis(*S*-methylisothiosemicarbazone) (PBIT) in a series of cobalt complexes has been studied by a range of methods, including spectroscopy [UV–vis, NMR, electron paramagnetic resonance (EPR), X-ray absorption spectroscopy (XAS)], cyclic voltammetry, X-ray diffraction, and density functional theory (DFT) calculations. Two complexes [Co^III^(H_2_L^SMe^)I]I and [Co^III^(*L^SMe^*)I_2_] were found to act as precatalysts in a Wacker-type oxidation of olefins using phenylsilane, the role of which was elucidated through isotopic labeling. Insights into the mechanism of the catalytic transformation as well as the substrate scope of this selective reaction are described, and the essential role of phenylsilane and the noninnocence of PBIT are disclosed. Among the several relevant species characterized was an unprecedented Co(III) complex with a dianionic diradical PBIT ligand ([Co^III^(L^SMe••^)I]).

## Introduction

**Scheme 1:**
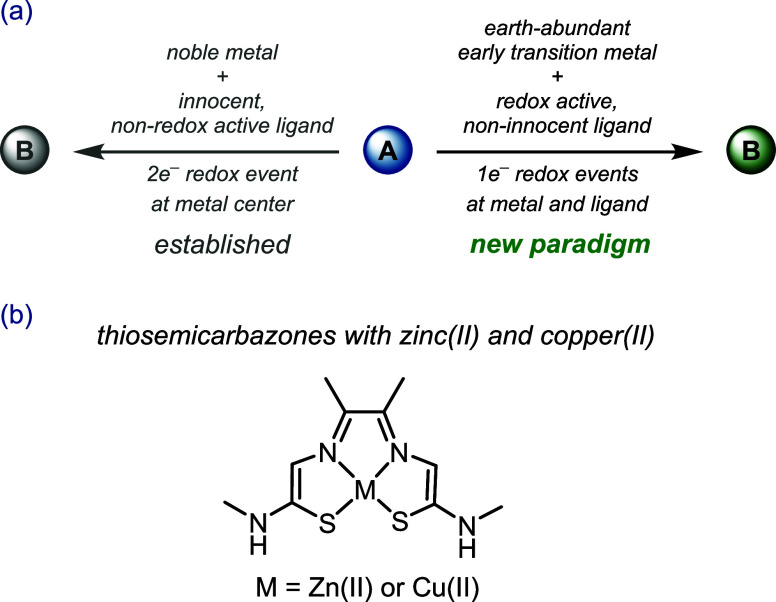
Use of Early Transition Metals in Combination with Redox-Active Ligands Has Led to a Paradigm Shift

Metal complexes with redox-active and redox noninnocent ligands^1−7^ continue to spark the interest of the chemical community^8−11^ as they not only boast intriguing electronic structures,^12−14^ as well as spectroscopic^15−17^ and magnetochemical properties,^18−21^ but also promise new opportunities for catalysis.^22−28^ A particular point of interest of redox-active ligands is their ability to act as electron reservoirs, enabling substrate activation at a metal center via intramolecular ligand–metal electron transfer.^3,29^ The propensity of redox-active ligands to bring about new reactivity at a bound transition metal and to promote challenging multielectron catalytic transformations by avoiding pathways with high-energy intermediates also motivates their use in catalysis.^23,30^ The recent replacement of noble metals (classically undergoing 2e^–^ transformations) by a combination of earth-abundant first-row transition metals and redox-active ligands (both enabling 1e^–^ redox events under mild conditions) in catalytic transformations such as water oxidation is just one prominent example (Scheme 1a).^23,30^

Distribution of the charges over several sites (metals and ligands), avoiding charge accumulation at a single metal center, endows the employed base metal with noble metal character.^3^ At the same time, the stability of higher-oxidation-state intermediates can be controlled by the redox-active ligand bound tightly to the metal center.^2,31^ Other important roles of redox-active ligands in metal complexes used in catalysis have been disclosed recently as (i) regulation of Lewis acidity/basicity of a transition metal by changing the ligand redox state, (ii) generation of ligand-centered radicals, (iii) substrate activation by the formation of a new ligand–substrate bond through radical reactivity, which might also result in inducing different spin states of the metal, and (iv) metal–ligand bifunctional substrate activation by a combined two-electron transfer.^5,23^ In addition, the search for new strategies relying on multiproton-storage ligand platforms, featuring reversible protonation and proton-releasing abilities, and redox activity for cooperative metal–ligand bifunctional substrate activation via proton-coupled electron transfer (PCET)—with the goal of finding new applications in catalysis—has intensified.^5,23,32^ This is exemplified by the inclusion of redox and proton-transfer sites into the ligand of a ruthenium catalyst used for water oxidation, which facilitated simultaneous transfer of electrons and protons with a decrease of redox potential, leading to new reaction pathways.^33,34^ Of particular note in terms of our interest in coordination chemistry with redox-active (iso)thiosemicarbazones^35^ are zinc(II) and copper(II) complexes (Scheme 1b) with a redox-active and proton-responsive tetradentate ligand, namely, diacetyl-bis(*N*-4-methyl-3-thiosemicarbazone), which were reported to catalyze the electrocatalytic hydrogen evolution reaction (HER).^36,37^ Cobalt complexes with redox noninnocent ligands have found successful application in a wide variety of catalytic transformations, including Negishi-type C–C cross-coupling reactions,^26^ (electro)catalytic C–C bond formation mediated by ligand-centered reduction,^38^ and a variety of other C–C and C–heteroatom bond-forming reactions,^17,39−44^ among others.^45−48^ Multielectron and multiproton catalytic hydrogen evolution reactions with cobalt(II) porphyrin that contains a xanthene moiety with a pendant carboxylic group to assist in proton relay are also well-documented.^49^

The pentane-2,4-dione bis(*S*-methylisothiosemicarbazone) (PBIT) backbone is unique in that it can be coordinated by a metal ion either as fully protonated species H_3_L^SMe^, as monoanion (H_2_L^SMe^)^−^, dianion (HL^SMe^)^2–^, trianion (L^SMe^)^3–^, or as a 2e^–^-oxidized species of the trianion (*L^SMe^*)^−^.^35^ Although its redox noninnocent behavior has been disclosed in nickel^50^ and iron complexes,^51,52^ it remains largely unknown for cobalt coordination compounds (Scheme S1).

**Scheme 2:**
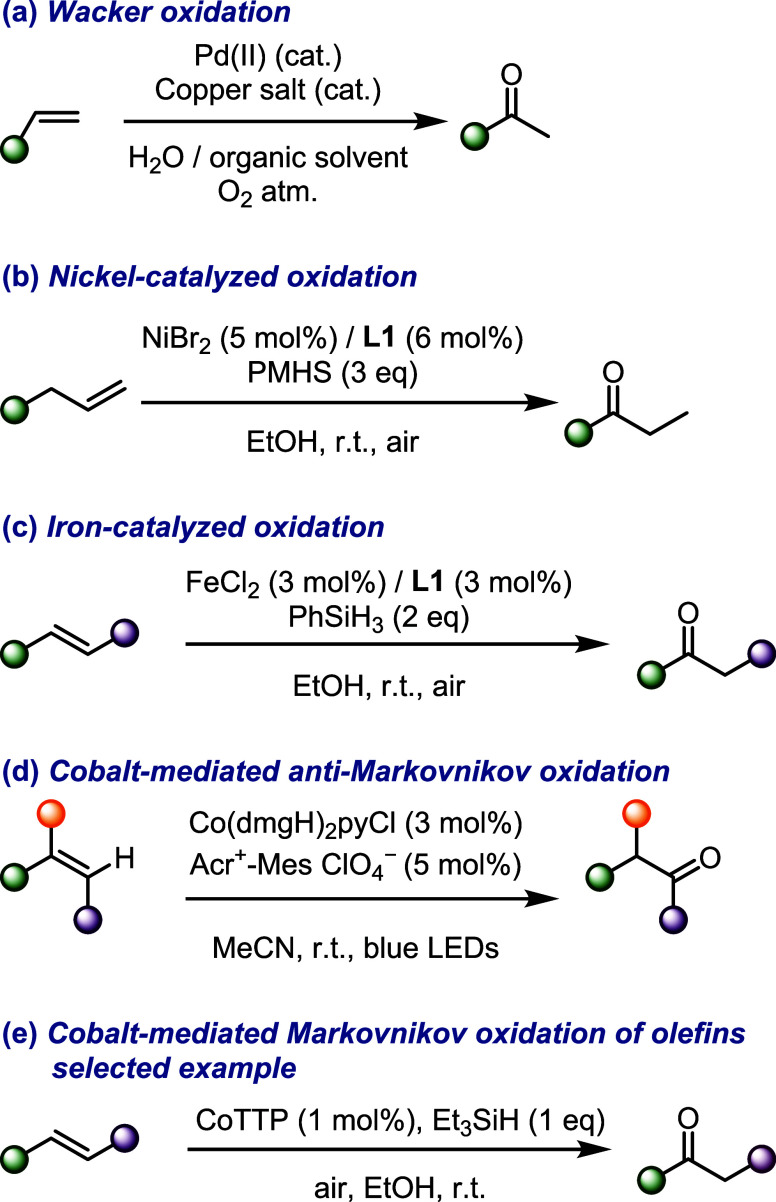
Catalytic Oxidation of Olefins (a) Wacker oxidation, (b) nickel-catalyzed oxidation, (c) iron-catalyzed oxidation, (d) cobalt-mediated *anti*-Markovnikov oxidation, (e) cobalt-mediated Markovnikov oxidation. **L1** = 2,9-Dimethyl-1,10-phenanthroline; PMHS = polymethylhydrosiloxane; dmgH = dimethylglyoximate monoanion; py = pyridine; TPP = 5,10,15,20-tetraphenyl-21*H*,23*H*-porphin.

Ketones are ubiquitous building blocks in man-made chemicals^53^ and natural products,^54^ and the remarkable breadth of synthetic transformations available to the carbonyl group renders ketones crucial building blocks in organic synthesis.^55^ Although a range of classical protocols have been developed to access this key functional handle (e.g., alcohol oxidation, ozonolysis, Weinreb ketone synthesis),^56^ ketone syntheses relying on catalytic pathways offer unique benefits related to waste reduction and atom economy.^57^ One such well-established, catalytic method for the synthesis of ketones is the Pd-catalyzed Wacker oxidation (Scheme 2a). Initially developed for the conversion of simple ethylene into acetaldehyde on an industrial scale (Hoechst–Wacker process), it was further optimized for more general use,^58^ typically relying on a Pd(II) catalyst, a redox cocatalyst, and dioxygen as the reoxidant. While Pd and other noble metals have risen to the forefront of this chemistry (see Scheme 1), they present issues of cost and toxicity. The past decade has seen renewed interest in developing earth-abundant base-metal catalysis, with advantages in environmental friendliness and economic viability, of these (and related) oxidations.^59,60^ An ideal homogeneous oxidation catalyst is expected to be stable and highly selective, perform well in nontoxic solvents and use air as the sole oxidant.^61^ Important contributions in this direction are nickel- and iron-based catalysts to promote the Wacker-type oxidation of olefins (Scheme 2b,c).^62−67^ A dual catalytic system involving a cobaloxime and an acridinium photocatalyst that yielded the anti-Markovnikov products under milder conditions was also recently reported (Scheme 2d).^68^ With few exceptions (Scheme 2e),^69^ previous reports on Co-mediated oxidation of olefins to ketones, in a Markovnikov fashion, either suffer from limitations in scope,^70−74^ require harsh conditions,^75^ or lead to unspecific reactivity.^76,77^ Some works also showed that, in the presence of Co and dioxygen, olefins can undergo oxidative cleavage, rather than oxidative hydration.^78,79^

**Scheme 3:**
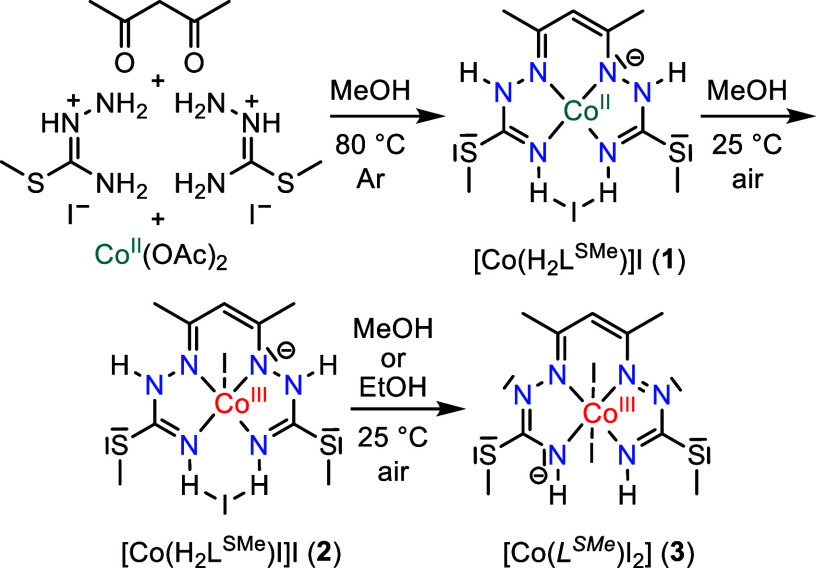
Conversion of **1** into **2**, and **2** into **3**

**Scheme 4:**
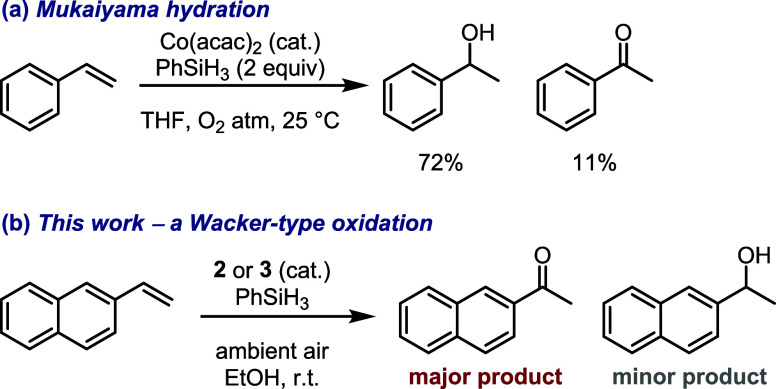
(a) Mukaiyama Hydration and (b) Wacker-Type Oxidation

Herein, we report the synthesis of a series of cobalt complexes supported by a chemically noninnocent and redox noninnocent PBIT ligand (**1**–**3**, Scheme 3), together with their characterization by NMR, UV–vis, and electron paramagnetic resonance (EPR) spectroscopies, X-ray absorption spectroscopy (XAS), cyclic voltammetry, and spectroelectrochemistry, variable-temperature magnetic-susceptibility measurements, single-crystal and powder X-ray diffraction, and density functional theory (DFT) calculations. We also describe the catalytic activity of these complexes, which, in the presence of a silane and dioxygen—in contrast to the related Mukaiyama hydration, which produces alcohols (Scheme 4a)—chemoselectively transforms olefins into the corresponding ketones in a Wacker-type process (Scheme 4b).

We also present mechanistic studies highlighting the essential role of phenylsilane and the noninnocence of the PBIT platform to enable this new catalytic oxidation of olefins.

## Results and Discussion

### Synthesis and Characterization of Cobalt Complexes in Various Protonation and Oxidation States

**Figure 1:**
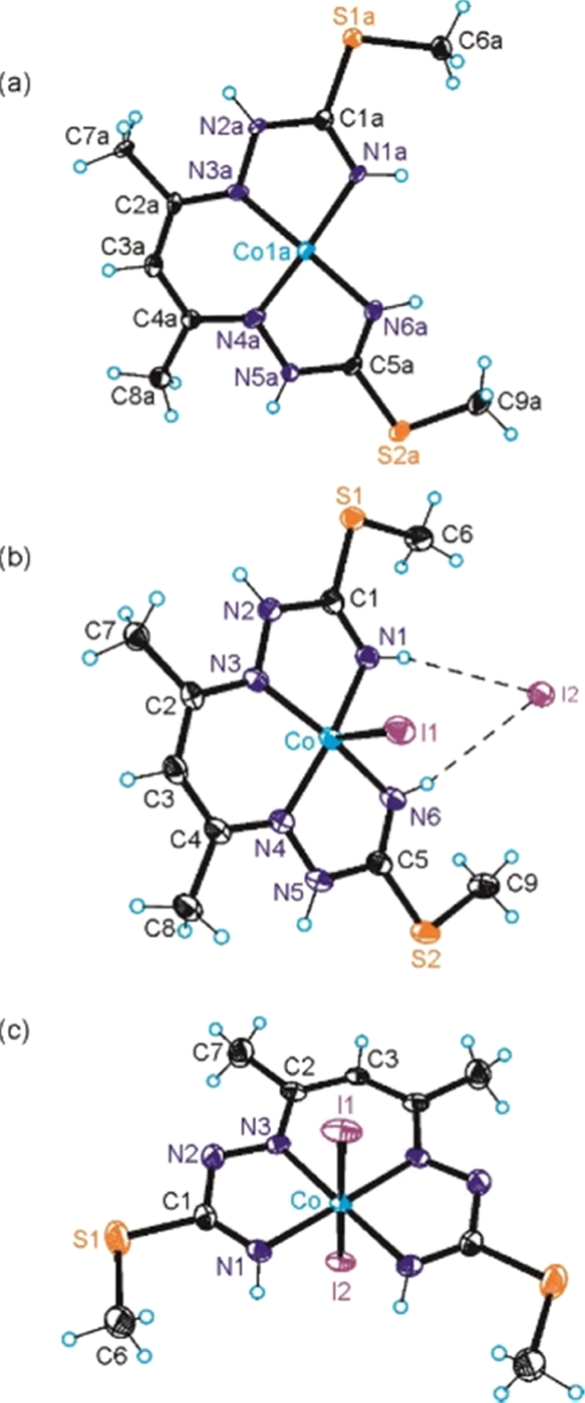
Oak ridge thermal-ellipsoid plot program (ORTEP) views of complex cations in (a) **1·0.5CH**_**3**_**OH** and complexes (b) **2·CH**_**3**_**OH** and (c) **3** with atom labeling schemes. Thermal ellipsoids are drawn at 50% probability level.

Our studies began with the synthesis of [Co(H_2_L^SMe^)]I (**1**, see Scheme 3 and Section S1) through condensation of pentane-2,4-dione (Hacac) with *S*-methylisothiosemicarbazidium iodide in the presence of cobalt(II) acetate. Reaction in methanol, heating at 80 °C under inert atmosphere, afforded a brown-cherry crystalline product upon slow cooling. The structure of the square-planar cobalt(II) complex [Co(H_2_L^SMe^)]I·0.5CH_3_OH (**1·0.5CH**_**3**_**OH**),^80^ in which the tetradentate ligand acts as a monoanion (H_2_L^SMe^)^−^, was established by single-crystal X-ray diffraction (SC-XRD) (vide infra, Figure 1).

**Scheme 5:**
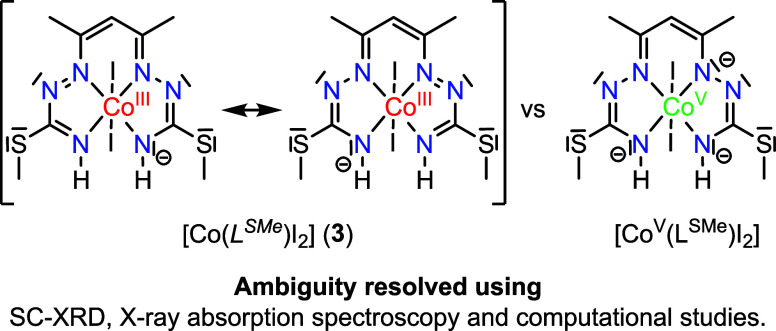
Ambiguity in the Assignment of the Oxidation Level of PBIT and Co in **3**

Notably, exposure of a solution of **1** to air resulted in gradual oxidation, as indicated by a striking color change of the brown-cherry solution to green-blue. The resulting solution, when left in a closed flask, produced blue-black crystals of [Co^III^(H_2_L^SMe^)I]I·CH_3_OH (**2·CH**_**3**_**OH**), the structure of which was determined by SC-XRD (see Sections S1–S4 for detailed characterization).^81^ Recrystallization of five-coordinate [Co^III^(H_2_L^SMe^)I]I (**2**) from ethanol or methanol in the presence of air afforded a six-coordinate complex, formulated as [Co^III^(*L^SMe^*)I_2_] (**3**) (see Scheme 5), through a 2e^–^ oxidation followed by the release of two protons. The central atom preserves its oxidation state +3 (rather than +5; see in-depth characterization below and in Sections S1–S3), where the fully deprotonated ligand is a two-electron-oxidized 12π-electron monoanion, which is denoted herein in italic style as (*L^SMe^*)^−^, to contrast it to the 14π electronic species (L^SMe^)^3–^ of the putative Co^V^ complex [Co^V^(L^SMe^)I_2_] (Scheme 5, right), in which the tetradentate ligand is fully deprotonated ((L^SMe^)^3–^), and two iodido coligands are bound axially.^82^

To find out whether other coordination geometries and/or protonation levels of the PBIT ligand in cobalt complexes can be reached, we performed a set of reactions with reductants and oxidants.

### Other Cobalt(III) Complexes

Air exposure of an ethanolic solution of **1** produced dark-red crystals of the dimeric associate [Co^III^(L^SMe,O^)I]_2_·C_2_H_5_OH (**4·C**_**2**_**H**_**5**_**OH**, see Section S1), in which each monoanionic ligand is oxidized at the central carbon of the Hacac moiety with the formation of a keto group H_2_L^SMe,O^ = 3-oxo-pentane-2,4-dione bis(*S*-methylisothiosemicarbazone), a reaction typical for transition-metal complexes with pentane-2,4-dione bis(thiosemicarbazones).^83−85^ The dimeric structure was disclosed by SC-XRD and IR spectroscopy (see Sections S1 and S2).

The reduction of complex **2** with phenylsilane under inert conditions and the consecutive exposure to air yielded mononuclear [Co^III^(L^SMe,O^)I(CH_3_OH)] (**5**, see Sections S1 and S2 for details).

### Other Cobalt(II) Complexes

In addition, it was found that the reaction of complex **2** in the presence of PhSiH_3_ under inert conditions afforded six-coordinate cobalt(II) complex [Co^II^(H_3_L^SMe^)I(CH_3_OH)]I·CH_3_OH (**6**·CH_3_OH), where the PBIT ligand preserved its fully protonated form (a similar behavior was observed in other cobalt(II) complexes, see Sections S1 and S2).

High-resolution X-ray crystallography allows for reliable assignment of the oxidation state of the redox-active or redox noninnocent ligand via comparison of bond metrics in similar compounds available in the literature,^86^ while the oxidation state of the central metal ion can be established by X-ray absorption spectroscopy,^87^ even if the ligand is a radical.^88^ Both tools have been successfully applied herein to resolve the ambiguity in the assignment of oxidation states of both PBIT and cobalt.

### X-ray Crystallographic Investigations of **1**–**3**

In order to facilitate structural comparison of the cobalt complexes with different levels of protonation of PBIT and different oxidation states of both the ligand and metal, single crystals of **1·0.5CH**_**3**_**OH**, **2·CH**_**3**_**OH** and **3** were subjected to SC-XRD investigations (see Section S2 for data collection, refinement, and metrical parameters, also for the other cobalt(II) and cobalt(III) complexes).

The coordination geometry of the Co^II^ ion (d^7^) in **1·0.5CH**_**3**_**OH** was found to be square-planar (Figure 1a). While, in the solid state, the vast majority of cobalt(II) complexes are distorted five- or six-coordinate species,^89−94^ square-planar cobalt(II) complexes have also been reported.^95−99^

The complex **2·CH**_**3**_**OH** is square-pyramidal (Figure 1b), with the monoanionic ligand (H_2_L^SMe^)^−^ coordinated to cobalt(III) in the basal plane and one iodido coligand in apical position. The second iodide acts as a counteranion and as a proton acceptor to two end NH groups in the plane of the tetradentate ligand. In addition, one methanol molecule is present in the asymmetric unit. The cobalt-to-apical iodido coligand bond distance of 2.5285(4) Å is well-comparable to that in a similar, previously reported cobalt complex^100^ but markedly shorter than that in coenzyme B_12_ model cobaloxime at 2.697(2) Å.^101^

In contrast to five-coordinate complex **2**, complex **3** is six-coordinate with the fully deprotonated tetradentate ligand coordinated equatorially to the central metal ion, while two iodido coligands are bound axially (Figure 1c). As expected, due to increased electronic repulsion in accord with the VSEPR theory, the bond lengths Co–N1, Co–N3, Co–I1, and Co–I2 in this six-coordinate complex are markedly longer than the corresponding bonds in the five-coordinate complex **2**. Given the full deprotonation of the original ligand by loss of three protons and binding of two iodido coligands to the central atom, its formal oxidation state should be +5 (see Scheme 5). As an alternative option, we analyzed the situation where the 14π trianionic ligand (L^SMe^)^3–^ is oxidized by loss of 2e^–^ to give a new 12π monoanionic system (*L^SMe^*)^−^. The sum of the bond distances Σ[6(C–N) + 2(N–N) + 2(C–C)] comes to 13.556 Å for [Co^III^(H_2_L^SMe^)I]I and is well-comparable with those in [Fe^IV^(L^SMe^)I],^51^ its *S*-ethyl derivative,^52^ and [{Fe^IV^(L^SMe^)}_2_(μ-O)]^102^ at 13.503, 13.491, and 13.495 Å, respectively. For [Co(*L^SMe^*)I_2_], in contrast, this sum is clearly smaller of 13.468 Å and is almost identical with the average value for two crystallographically independent anions in AsPh_4_[Fe^II^(*L^SMe^*)(CN)_2_]^52^ of 13.470 Å. Thus, inspection of SC-XRD metrical parameters provides strong evidence for physical oxidation states −1 for the tetradentate ligand^103^ and +3 for Co in the six-coordinate di-iodido complex **3**.

The electronic structures of catalytically relevant complexes **1**–**3** were further investigated by X-ray absorption spectroscopy (XAS).

### X-ray Absorption Spectroscopy

**Figure 2:**
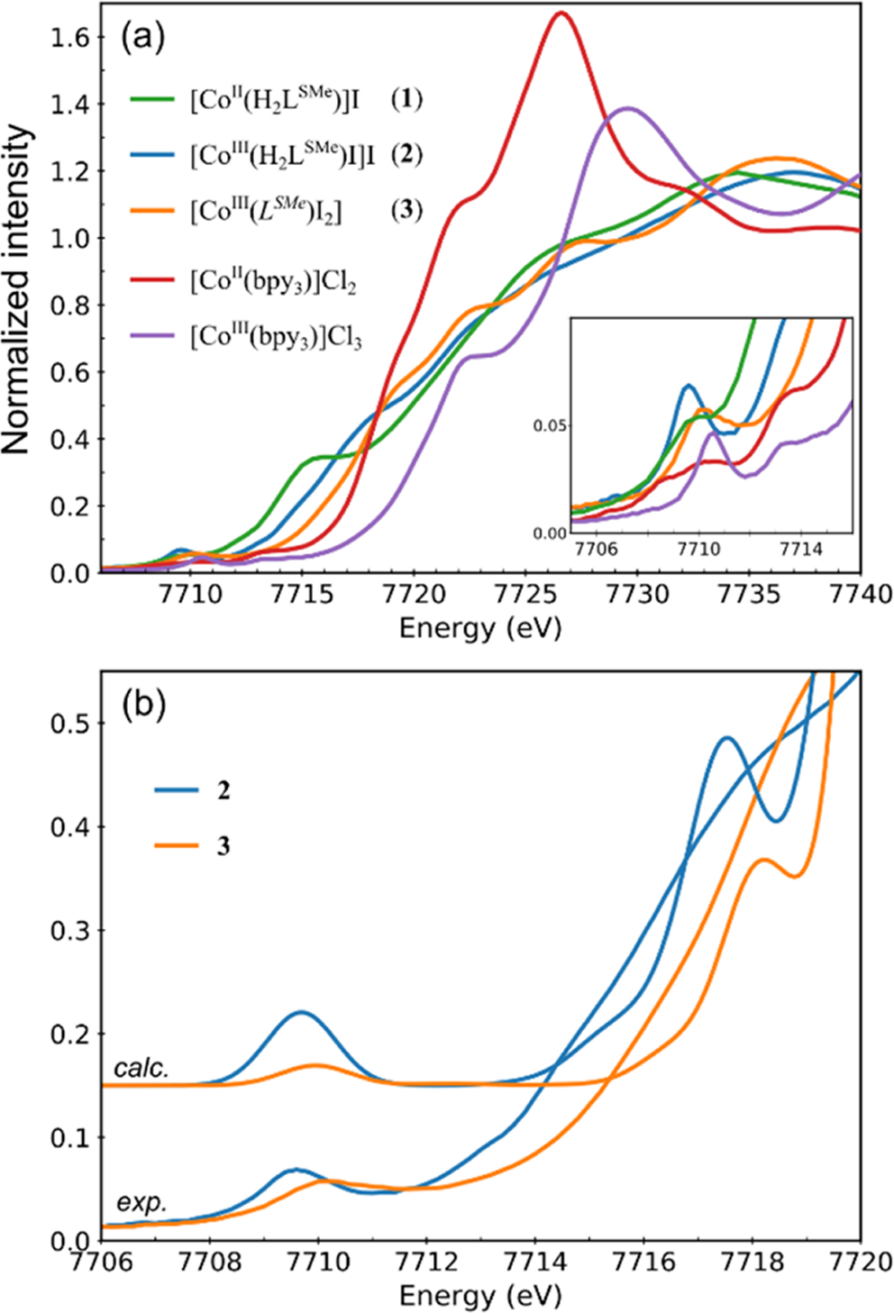
(a) Co K-edge XAS of **1**–**3**, [Co^II^(bpy)_3_]Cl_2_, [Co^III^(bpy)_3_]Cl_3_, and expanded pre-edge region (inset). (b) Overlay of the experimental spectra of [Co^III^(H_2_L^SMe^)I]I (**2**) and [Co(*L*^*SMe*^)I_2_] (**3**) with the respective calculated spectra by time-dependent density functional theory (TDDFT)/B3LYP (energy shift: 93.3 eV, scaling factor: 0.1, line shape: Gaussian, line width: 1.5 eV).

The Co K-edge XAS of [Co^II^(H_2_L^SMe^)]I (**1**), [Co^III^(H_2_L^SMe^)I]I (**2**), and [Co^III^(*L*^*SMe*^)I_2_] (**3**) were measured and compared to those of Co^II^ and Co^III^ tris-2,2′-bipyridine complexes [Co^II^(bpy)_3_]Cl_2_ and [Co^III^(bpy)_3_]Cl_3_ as reference (Figure 2a). As the oxidation state decreases, the lower effective nuclear charge further destabilizes the Co 1s electron, decreasing the energy required to remove it. This effect is evident in the pair of tris-bipyridine reference compounds in the rising edge region at 7717–7725 eV, as the rising edge of the Co^II^ complex is shifted to lower energies. The complexes **1**–**3** show the rising edge region at 7714–7717 eV shifted to even lower energies than [Co^II^(bpy)_3_]Cl_2_ as a result of the larger covalency of the metal–ligand bonds with the PBIT ligand relative to bipyridine and not due to the lower oxidation state. The effect of covalency on the position of the K-edge is known and is nicely illustrated by the ferric halide series.^104^ Despite both **2** and **3** being trivalent and the PBIT ligands both being monoanionic, the rising edge of the former is shifted to lower energies relative to the latter. The (H_2_L^SMe^)^−^ ligand in **2** is the two-proton two-electron reduced equivalent with respect to (*L*^*SMe*^)^−^ in **3**, which is associated with Co–N bonds ca. 0.02 Å shorter and hence more covalent (**2**: 1.860/1.881 Å and **3**: 1.876/1.899 Å). This finding evidences the effect of the ligand oxidation state in the modulation of the metal–ligand bond covalency. A closer inspection revealed that **2** has inflection points at 7713.0 and 7715.9 eV, whereas in **3** these occur at 7714.6 and 7717.4 eV. These features should be associated with the differences in the ligand-based low-lying empty molecular orbitals. Finally, the rising edge of **1** is marked by a distinctive intense feature at 7715 eV, usually associated with the transition to Co 4p_*z*_, which is low-lying due to the square-planar geometry.^105^

The pre-edge peaks of the PBIT-based complexes **1** and **3** are weaker than for **2**, as expected from the centrosymmetric Co site revealed by X-ray crystallography (see Figure 2 and Section S3 for detailed discussion).^106^ The calculated XAS spectra in Figure 2b correctly capture the weaker pre-edge of **3**, its pre-edge peak and rising edge both shifted to higher energies relative to **2**, and the overall number of features. These findings lend credence to the interpretation of the excited states in the pre-edge region under the TDDFT framework.^107−112^ The key excited states are assigned in Figure S8 in Section S3.^113^ The weak single-featured pre-edge peak of the six-coordinate **3** at 7710.2 eV is associated with two nearly degenerate quadrupole-allowed transitions into the empty d_*z*^2^_ and d_*x*^2^–*y*^2^_. The ground-state electronic configuration is d_*xz*_^2^ d_*yz*_^2^ d_*xy*_^2^ d_*z*^2^_^0^ d_*x*^2^–*y*^2^_^0^ with the d_*z*^2^_ lower than the d_*x*^2^–*y*^2^_ due to the weaker field imposed by the axial *trans*-di-iodido ligands. The relatively stronger single-featured pre-edge peak of the five-coordinate **2** at 7709.6 eV is dominated by the transition into the d_*z*^2^_ admixed with Co 4p_*z*_. The electronic configuration and d-orbital splitting are similar, with the main difference being that d_*z*^2^_ is stabilized in **2** relative to **3** due to the presence of one apical ligand in **2** instead of two in **3**. This is consistent with the fact that the experimental pre-edge peak of **2** is shifted by 0.6 eV to lower energy relative to **3**.

Finally, the absorption features at the onset of the rising edge at 7715–7716 eV (Figure 2) in both compounds are attributed to transitions into π* with main C=N within the isothiosemicarbazone moiety (see Figure S8 in Section S3) and the prominent features at 7717−7718 eV are assigned to transitions into a π* on the three central carbons of the Hacac moiety. The first feature is 1.1 eV higher in **3**, relative to **2**, whereas the second feature is only 0.5 eV higher. This indicates that the oxidation state of the ligand affects mainly the π system located on C=N and offers an explanation why the rising edge of **3** appears more oxidized than **2**. Beyond these two features, the rising edge of **3** displays two more distinct intensity modulations at 7722.5 and 7727.5 eV. According to TDDFT, they are excitations to high-lying MOs with large Co 4p_*z*_ character combined with the *trans* iodido ligands.

### Magnetism and EPR Spectroscopy

Given the purity of the bulk sample (see Section S4, Figure S14), variable-temperature magnetic-susceptibility measurements for complex **1** were performed, attesting low-spin electronic configuration (*S* = 1/2 ground state, d^7^) for cobalt(II) (see Section S4, Figures S15 and S16). The X-band EPR spectroscopy of powdered sample **1** additionally confirmed the antiferromagnetic coupling in the cofacial cation dimers revealed in the magnetic measurements (see Section S4, Figure S17 for details). Together, this evidence further corroborated the proposed structure for **1**.

### Kinetics of Oxidation of 2 with Air Oxygen

**Figure 3:**
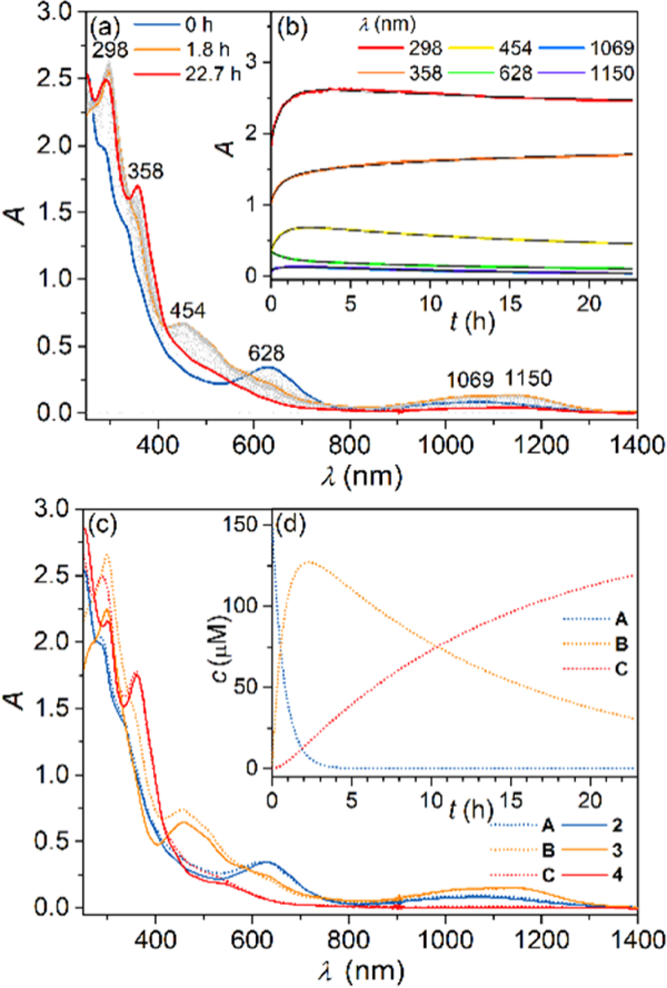
(a) UV–vis–NIR spectra of 150 μM **2** in EtOH oxidized by air oxygen over 23 h; (b) kinetic traces at selected absorption maxima together with the kinetic model fit (see above); (c) dotted lines UV–vis–NIR spectra obtained for the three compounds **A**, **B**, and **C** used in the kinetic model, solid lines UV–vis–NIR spectra of 150 μM **2**, **3** and 75 μM **4** in EtOH under Ar; (d) concentration profiles for the three compounds **A**, **B**, and **C** from the kinetic model.

The kinetics of the oxidation of **2** with air oxygen were studied in EtOH at 20 °C. UV–vis–NIR spectra were recorded over 23 h in an open optical cell and are shown in Figure 3. The evolution of optical bands indicated a consecutive reaction pathway with a relatively stable intermediate. Assuming the oxygen in the solution is in a significant excess to the studied complex, the kinetic traces (Figure 3b) were fit with a simple model involving two consecutive reactions obeying pseudo-first-order kinetics (eq 1).
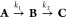
1

The global analysis approach with this simplified model reproduced the observed kinetics reasonably well, with the individual rate constants *k*_1_ = 3.72 ± 0.07 × 10^–4^ s^–1^ and *k*_2_ = 2.00 ± 0.07 × 10^–5^ s^–1^. The spectra of the three reaction components **A**, **B**, and **C** and the corresponding concentration profiles are shown in Figure 3c,d, respectively. Comparison of the spectra obtained from the kinetic model (Figure 3c, dotted lines) with the spectra of **2**, **3**, and **4** (Figure 3c, solid lines) gained with the authentic samples under Ar atmosphere revealed a very good match. Thus, the oxidation of **2** in EtOH with air oxygen proceeds as a consecutive two-step process with the six-coordinate complex **3** as an intermediate, and complex **4** as the final product.

### Electrochemical and Spectroelectrochemical Studies

**Figure 4:**
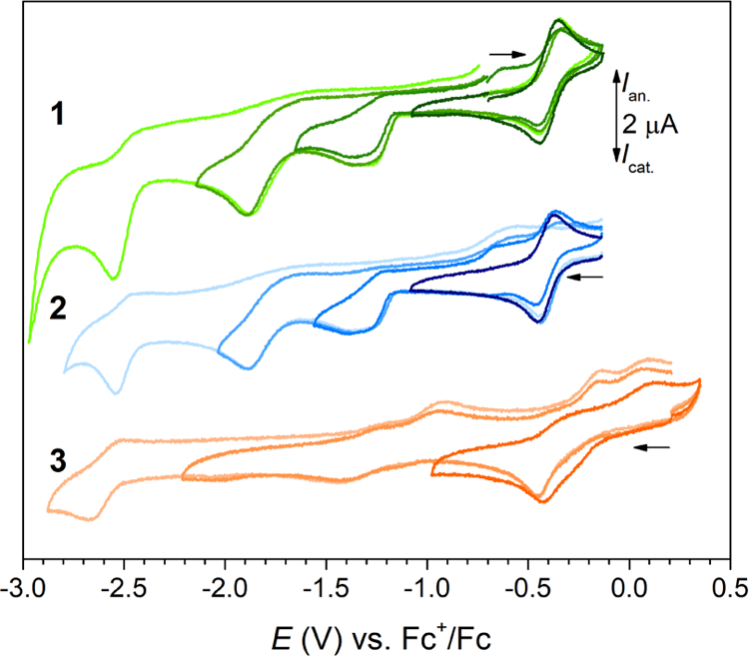
Cyclic voltammograms of ∼0.5 mM **1**–**3** in 0.1 M *n*Bu_4_NPF_6_/MeCN, recorded with glassy carbon working electrode at a scan rate of 0.1 V s^–1^. Scans with different vertex potentials examining the reversibility of individual redox processes are shown with varying color tone. Horizontal arrows indicate the CV scan direction.

Electrochemical investigation of complexes **1** and **2** (with PBIT ligand in (H_2_L^SMe^)^−^ form), **3** (with (*L*^*SMe*^)^−^ ligand), **4** (with (L^SMe,O^)^2–^ ligand), and **8** (with (H_3_L^SMe^)^0^ ligand) was carried out by cyclic voltammetry (Figure 4 and Section S5, Figure S18).

**Figure 5:**
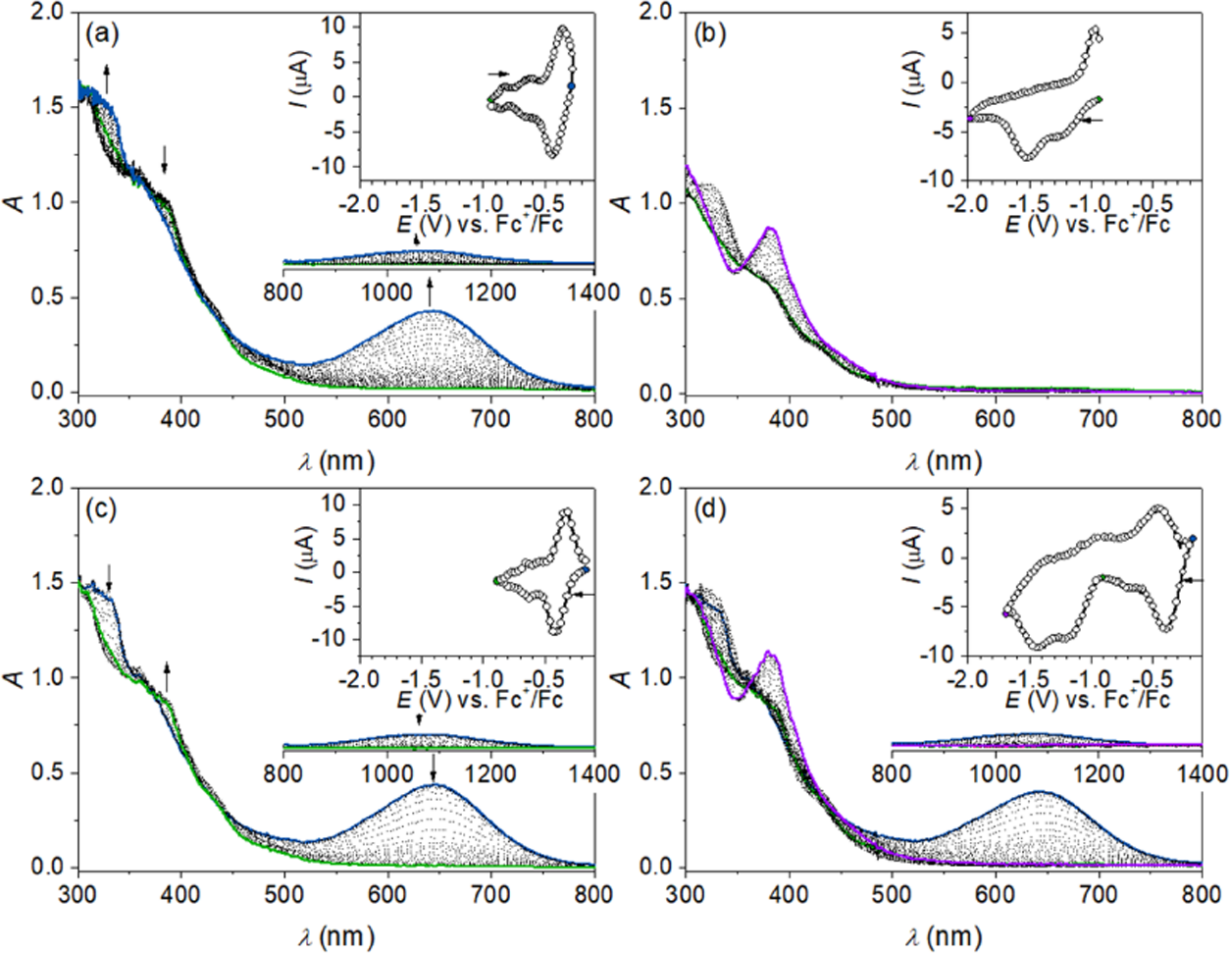
UV–vis–NIR spectra obtained upon the spectroelectrochemical measurement in 0.1 M *n*Bu_4_NPF_6_/MeCN: (a) oxidation of **1** and (b) reduction of **1**, as well as (c) first reduction of **2** and (d) first and second reductions of **2**. NIR regions are shown for relevant species. Insets show the corresponding CVs measured with the Pt working electrode at a scan rate of 3 mV s^–1^. Circles mark potentials where the spectra were sampled, and for highlighted records, the color matches the spectrum line color.

Half-wave potentials *E*_1/2_ for electrochemically reversible processes and cathodic peak potentials *E*_pc_ for electrochemically irreversible events for **1**–**3** (Figure 4), **4**, and **8** were measured (see Section S5 for detailed graphical representation; Table S6 and Figure S18). In the investigated potential window, the Co^II^ complex **1** features a reversible single-electron oxidation at *E*_1/2_ = −0.39 V vs Fc^+^/Fc and three irreversible reductions with *E*_pc_ of −1.36, −1.89, and −2.56 V, respectively. An essentially identical voltammetric record was obtained with Co^III^ complex **2**, where in turn the redox event at *E*_1/2_ = −0.40 V represents the first single-electron reduction of the compound. Complex **3** displays a rather complex cathodic response comprising three irreversible reductions at *E*_pc_ of −0.45, −1.43, and −2.67 V vs Fc^+^/Fc, respectively. Complexes **1** and **2** bear the same PBIT ligand, but differ by charge state and the formal oxidation state of the central metal. The reversible redox process found in their CV records at ca. −0.40 V can thus be assigned to the redox transformation of the central metal representing the Co^III^/Co^II^ redox couple (in **1** Co^II^ is oxidized to Co^III^, in **2** Co^III^ is reduced to Co^II^, vide supra). The assignment is corroborated by spectroelectrochemical analysis (Figure 5a,c).

Single-electron oxidation of **1** provides UV–vis–NIR spectra identical to those of complex **2** in *n*Bu_4_NPF_6_/MeCN, where the blue-green color of the solution is caused by characteristic absorption at 644 nm and the NIR band at 1063 nm also appears. Vice versa, the optical spectra obtained upon the first reduction of **2** almost lack the absorption in the visible range and match the bands of complex **1**.

The reduction of complex **3** at *E*_pc_ of −0.45 V is electrochemically irreversible, and repeated CV scans (not shown) reveal a successive decrease in cathodic peak current, indicating a follow-up chemical reaction. Therefore, the Co^II^ complex obtained from **3** is unstable and undergoes an as of yet unidentified transformation in solution.

Additional reductions were observed in the entire complex series at potentials more negative than −1.5 V. We believe these to be reductions of the PBIT ligand (see Section S5 for details).

### Computational Studies of Complexes **1**–**3**

Evaluation of the metrical charge of the ligands (oxidation level of the ligand,^114^ metrical oxidation state)^115^ was performed following previously reported approaches.^114,115^ Here, a Zn^2+^ cation is used to replace Co^2+/3+^ (see Section S6, Scheme S3) to optimize geometries of the differently charged Zn-containing species (as Zn^2+^ can be assumed as redox inert, the metrical charge of the ligand is well-established). Careful analysis (see Section S6 for details) revealed that the metrical charge of the ligand is −1 (see Section S6, Table S7) for all three species **1**–**3**. Thus, one can assign the oxidation state of the central Co atom in these complexes to +2, + 3, and +3, respectively. The obtained charges are further validated by means of localized orbitals that indicate d^7^, d^6^, and d^6^ electronic configuration of Co in the studied complexes **1**–**3** (see Section S6, Table S8), respectively.

**Figure 6:**
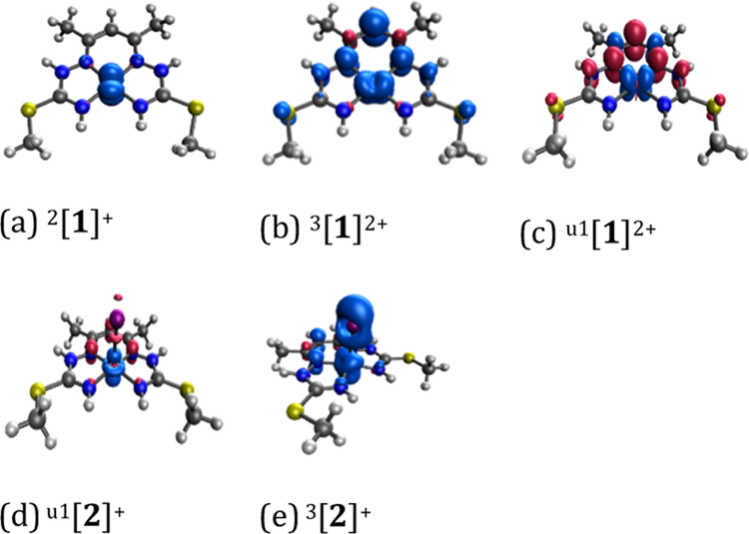
Spin density of chosen species studied, namely, (a) ^2^[**1**]^+^, (b) ^3^[**1**]^2+^, (c) ^u1^[**1**]^2+^, (d) ^u1^[**2**]^+^, and (e) ^3^[**2**]^+^; the isosurface value is 0.004 e·bohr^–3^.

The preferred spin state of the ^2^[**1**]^+^ complex is a doublet, with the spin density (as well as β-LUMO) being localized at the central Co atom (a mix of 3d_*xz*_ + 3d_*yz*_ AOs); see Figure 6. The formal 3d configuration of Co in ^2^[**1**]^+^ is d_*z*^2^_^2^ d_*xy*_^2^ d_*xz*_^1.5^ d_*yz*_^1.5^ d_*x*^2^–*y*^2^_^0^ according to d(Co)-like Mulliken population analysis (MPA). BS singlet spin state in ^u1^[**2**]^+^ is found just 0.3 kJ/mol below the closed shell ^1^[**2**]^+^ (see Section S6,Table S8).

The ^u1^[**2**]^+^ spin state has a low *S*^2^ value (0.137) and a low spin density at Co 0.260, of d_*z*^2^_ (Co) AO character, and an opposite spin density of −0.260 on the ligand (possibly to be considered as a half-electron BS open shell system, see Figure 6d and Table S8). Nevertheless, according to the localized orbitals, in both ^u1^[**2**]^+^ and ^1^[**2**]^+^, the central Co atom is in the oxidation state +3 (d^6^ electronic configuration for all considered spin states is found, see Table S8). The formal 3d configuration of ^1^[**2**]^+^ is d_*xy*_^2^ d_*xz*_^2^ d_*yz*_^2^ according to d(Co)-like MPA. In the case of ^1^[**3**]^0^, the restricted singlet state is found preferred (see Table S8) with a d^6^ electronic configuration of cobalt (d_*xy*_^2^ d_*xz*_^2^ d_*yz*_^2^ electronic configuration). In the case of dimer [**4**]^0^, the restricted singlet spin state is preferred (see Section S6, Table S8) with a d^6^ electronic configuration on both cobalt atoms (d_*xy*_^2^ d_*xz*_^2^ d_*yz*_^2^ electronic configuration), like in ^1^[**3**]^0^. All of the findings are in accordance with the XAS results and sharpen the experimental results of the studied species **1**–**3**.

Considering the electrochemistry of **1** and **2**, the frontier orbitals are worth to be discussed in further detail. The reduction (oxidation) locus of **1** can be directly related to the shapes of the LUMO (HOMO) orbitals. The LUMO has a 61% d_*x*^2^–*y*^2^_ Co character (see Section S5, Figure S20e), suggesting the reduction of Co^II^ to Co^I^ (physical charge +2 to +1) in **1**, despite the coordination of the axial coligand being deterministic for the redox locus assessment (vide supra). The analysis of localized orbitals and (MPA d-populations) suggests that the central atom is the reduction locus, but due to the ligand–metal noninnocent character, the situation is not a pure resonance structure with Co^I^ (one localized Co 3d orbital of **1** after reduction is occupied only by 1.7 electrons, and the MPA AO d-populations are shifted from the ideal value of two). Thus, the reduction of **1** can also partially affect the ligand (to ca. 25%). Notably, the oxidation locus of **1** is the ligand, when the axial position is left out of coordination, according to the β-HOMO (see Figure S20g), which is energetically above the α-HOMO Figure S20d. On the other hand, XAS and theoretical approaches identify Co^III^ in **2**. The α- and β-HOMO of **2** have 10 and 9% Co d_*z*^2^_ character (for the α- and β-LUMO, the corresponding values are 30 and 52%). Hence, the oxidation (locus relevant to HOMO) does not involve the central atom to a large extent in **1**, while especially the high Co d_*z*^2^_ character of β-LUMO (notice the singlet unrestricted BS regime) points to the Co atom as the reduction locus of **2**, i.e., Co^III^ is reduced to Co^II^. According to these findings, one has to hypothesize that the coordination of **1** and **2** is essentially the same in MeCN solution, i.e., iodide—which is contained in both **1**, as the counteranion, and **2**, within the coordination sphere—is either replaced by MeCN (upon dissolving or reduction) or plays a dynamic role in the redox cycle, leading to the same cyclic voltammograms for the first oxidation of **1** and reduction of **2** of the Co^II^/Co^III^ couple.

### Catalytic Studies

While investigating the potential for the catalysis of cobalt(III) complexes **2** and **3**,^69,77,116−118^ we found that in the presence of PhSiH_3_ and dioxygen,^119^ 2-vinylnaphthalene (**9a**) could be oxidized to the corresponding ketone (**10a**) in a Wacker oxidation-type fashion (cf. Scheme 2). Particularly striking is the fact that, while the conditions are reminiscent of the Mukaiyama hydration of alkenes to alcohols (cf. Scheme 4a), the process we uncovered almost exclusively delivers ketones as the reaction products (cf. Scheme 4b). While this selectivity is not entirely unknown, as evidenced by a seminal report by Matsushita,^71^ the underlying strategy did not retain the attention of the community—at least in part, as it suffered from the inconvenient requirement for an O_2_ atmosphere.

Following the discovery of the ability of complex **3** to oxidize **9a** to **10a**, we embarked on optimization of the reaction. Remarkably, using a catalyst loading of only 1 mol %, in the presence of PhSiH_3_ in EtOH at room temperature and under exposure to ambient air, a high yield for the ketone product was obtained (Table 1, entry 1). Complex **2** was also found to be capable of mediating the reaction, but the transformation was found to be less robust (entry 2). Reaction with complex **4** did not lead to any appreciable amounts of product, indicating the necessity for a ligand capable of participating in redox events (entry 3).^120^ Substitution of PhSiH_3_ for the silicon industry byproduct polymethylhydrosiloxane (PMHS) resulted in a drastic decrease in yield (entry 4), while the negative effect of performing the reaction under more concentrated conditions was less pronounced (entry 5), and the use of a pure O_2_ atmosphere proved to have little effect (entry 6). Control experiments conducted in the absence of complex **3**, without PhSiH_3_ or under anoxic conditions did not yield any product (entries 7–9). To demonstrate that alcohol **11a** was not formed by the reduction of the ketone with PhSiH_3_, acetophenone was exposed to the reaction conditions but remained intact.

**Table 1:**
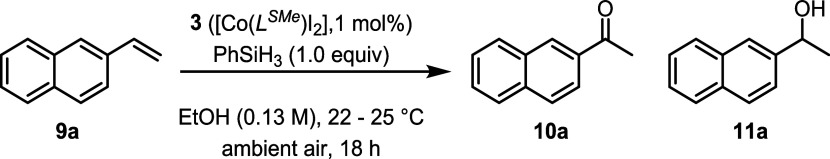
Optimization of the Reaction Conditions^a^

entry	deviation from standard conditions	yield of **10a** (%)
1	none	80 (76)
2	**2** ([Co(H_2_L^SMe^)I]I) instead of **3** ([Co(*L*^*SMe*^)I_2_])	72^b^
3	**4** ([Co^III^(L^SMe,O^)I]_2_) instead of **3**	26
4	PMHS instead of PhSiH_3_	29
5	0.5 M instead of 0.13 M	67
6	O_2_ atmosphere instead of ambient air	74
7	No precatalyst	<5
8	No PhSiH_3_	<5
9	anoxic solvent + Ar atmosphere instead of ambient air	<5

a Reactions were performed on a 0.5 mmol scale and yields were quantified by ^1^H NMR using 1,3,5-trimethoxybenzene as the internal standard. The isolated yield is given in parentheses.

b An increase in the amount of undesired alcohol (**11a**) was also observed.

**Chart 1:**
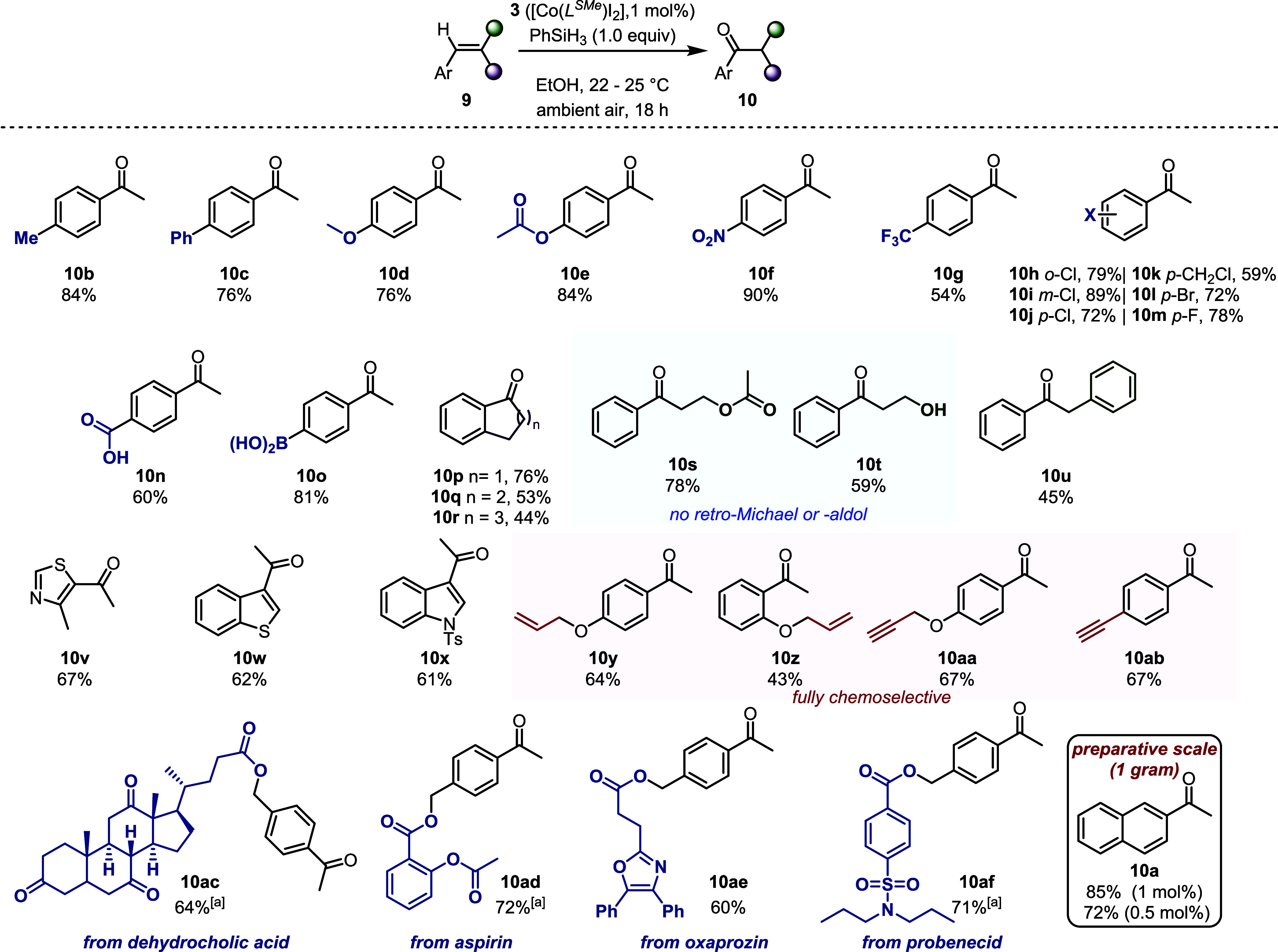
Scope of Oxidized Productsa

Having established optimized reaction conditions, the applicability of the transformation to different olefins was investigated (Chart 1). A wide range of substituents on the phenyl ring, including various functional groups with different electronic effects, were successfully oxidized in a fully regioselective manner (**10b**–**o**). As illustrated by the series of chloro-substituted derivatives, the reaction was not limited to *para* substituents but also tolerated *ortho* and *meta* substitution (**10h**–**j**). 1,2-Disubstituted styrenes, either in their cyclic or linear form, afforded the desired products in moderate to good yields (**10p**–**u**), and the mild reaction conditions allowed for the preparation of products **10s** and **10t** without the formation of retro-Michael or retro-Aldol byproducts. Given the importance of heterocycles in the discovery of new biologically active compounds, we investigated the tolerance of this protocol toward a range of electron-rich heterocycle-substituted alkenes, with the obtention of **10v**–**x** underlining the chemoselectivity of this oxidation. In addition, the chemoselectivity of the transformation using substrates containing other alkene- or alkyne substituents was studied. We were pleased to find that the reaction displayed complete selectivity for styrene-like olefins, leaving all other unsaturation untouched (**10y**–**ab**). Moreover, the applicability of the transformation to more complex substrates derived from natural products or pharmaceuticals was also investigated. Gratifyingly, we found that, even in these more complex scenarios, the reaction proved to be robust, and the products could be obtained in good yields (**10ac**–**af**). Finally, to demonstrate the scalability of oxidation, 1 g of model substrate **9a** was subjected to the reaction conditions, providing **10a** in high yield—in this context, we also showed that the catalyst loading could be reduced to 0.5 mol % without significant deterioration of the isolated yield.

a Reactions were performed on a 0.5 mmol scale. All yields refer to isolated materials. Tetrahydrofuran (THF) was used instead of EtOH due to solubility issues.

Apart from the potential synthetic utility of the method, the mechanistic aspects and its deviation from the venerable Mukaiyama reaction are intriguing. Therefore, we performed a series of experiments to gain insight into the mechanism of this catalytic reaction.

### Insight into the Mechanism of Catalytic Oxidation

**Scheme 6:**
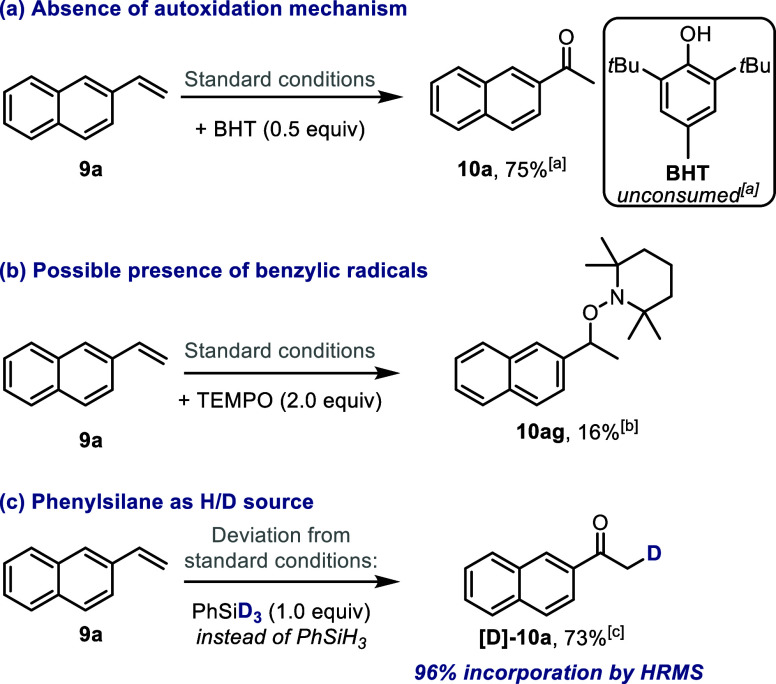
Additional Mechanistic Studies (a) The addition of BHT (dibutylhydroxytoluene) indicates a mechanism other than autoxidation. (b) Observation of TEMPO-adduct **10ag** points toward the involvement of a benzylic radical. (c) Crucial role of the reducing agent (phenylsilane) in promoting the reaction. [a] NMR yield. [b] NMR conversion. [c] Isolated yield.

Initially, poisoning experiments were conducted in order to assess whether catalysis occurs under homogeneous or heterogeneous conditions. Thus, while the presence of PPh_3_ or pyridine led to diminished yields being obtained, addition of elemental Hg did not lead to a significant change (see Section S7 for details). These results support the notion of homogeneous catalysis and the noninvolvement of Co nanoparticles. The fact that, under inert atmosphere and in an anoxic solvent, no desired product could be observed (Table 1, entry 9) suggests that air dioxygen is most likely incorporated into the products. Next, to rule out an autoxidation mechanism, we conducted the reaction in the presence of butylated hydroxytoluene (BHT), an efficient oxygen-centered radical trap (Scheme 6a).^121^ Notably, **10a** was formed in the expected yield, while BHT remained unconsumed. In addition, replacing the catalyst with an organic initiator such as azobis(isobutyronitrile) (AIBN) did not result in any conversion.^121^ These collected data suggest that highly reactive oxygen species are not involved in the oxidation process. Other mechanistic possibilities such as alkene hydration and subsequent dehydrogenation or Meinwald rearrangement^122^ were also excluded due to the lack of reactivity of 1-(2-naphthyl)ethanol or styrene oxide under standard conditions. Subsequently, when 2,2,6,6-tetramethyl-1-piperidinyloxy (TEMPO) was used as an additive, most of the reactivity was quenched, but we were able to identify the TEMPO-adduct **10ag**, suggesting the formation of a benzylic radical (Scheme 6b).

As a control, in the absence of complex **3**, no detectable amount of **10ag** was found. Finally, when deuterated phenylsilane (PhSiD_3_) was used as the reducing agent, 96% deuterium incorporation in the methyl group was obtained, as measured by HRMS (see Scheme 6c and Section S7 for details), while the use of PhSiH_3_ in EtOD did not lead to any labeling. Typically, the mechanism of the Mukaiyama hydration is represented by the formation of Co–H species after reduction by a silane.^118^ However, considering the studies of Peters^123^ and Norton,^124^ in which the formation of transient Co(III)–H species within the cobaloxime system was questioned, we decided to study the reduction of the precatalysts used (**2** and **3**) by PhSiH_3_ in more detail.

The chemical transformations of PhSiH_3_ in MeOH-*d*_4_ in the presence of complex **2** (0.3:1) and in its absence were investigated by ^1^H and ^29^Si NMR spectroscopy.^125^

In the absence of **2**, the spectra remain practically time-independent, while the presence of **2** elicits significant changes, leading to almost complete disappearance of the quartet signal between 7.32 and 7.37 ppm after 155 min, implying solvolysis of PhSiH_3_ (see Section S7, Figure S80). Similarly, ^29^Si NMR spectra were measured over time (60 min), again in the presence and absence of **2** (see Section S7, Figure S81). We noticed that the ^29^Si resonance of PhSiH_3_ at δ(^29^Si) = −61.16 ppm decreased gradually in the presence of **2** and three additional signals appeared at −14.90, – 26.23, and −54.56 ppm. While the former two resonances disappeared over time, the third signal reached maximal intensity after the first two species had disappeared completely. These time-dependent spectra mirror the consecutive dehydrogenative coupling of PhSiH_3_ with MeOH-*d*_4_ mentioned in many other catalytic studies.^62,65,66,126−129^ Stepwise formation of PhSiH_2_(OCD_3_), PhSiH(OCD_3_)_2_, and PhSi(OCD_3_)_3_ in the presence of **2** was confirmed by the measurement of the ^29^Si signal of PhSi(OCH_3_)_3_ and compared with the time-independent spectra of PhSiH_3_ measured in the absence of **2** (see Figure S81). It is worth noting that Ph(*i*-Pr–O)SiH_2_ has been found to be an exceptionally efficient reductant in catalytic reactions,^130^ with detailed mechanistic pictures of such transformations recently reported.^131^

The ^1^H NMR spectrum of the SCD_3_-labeled complex **2** in the presence of PhSiH_3_ (1 equiv) in MeOD-*d*_4_ after 24 h showed a singlet at 4.46 ppm and a 1:1:1 triplet at 4.42 ppm with *J*_HD_ ≈ 43 Hz (Figure S82), which can be attributed to H_2_ and HD, respectively, in solution.^132,133^

Interestingly, in a ^1^H NMR spectrum recorded at room temperature, the signals of the initial complex **2** disappeared and four new signals, at 11.02 and −11.67 ppm (attributed to one species) and 4.20 and −21.48 ppm (attributed to a second distinct species), emerged. The line width of the four proton resonances is large and the chemical shifts are spread into the negative region of the spectrum, which is not typical for diamagnetic species and therefore potentially point to the existence of two paramagnetic Co(II) species.^123^ However, similar signals in the negative range have also been attributed to Co(III)–H^124,132^ or to transient Co(I)–H species^129^ in hydrogenation reactions.

**Figure 7:**
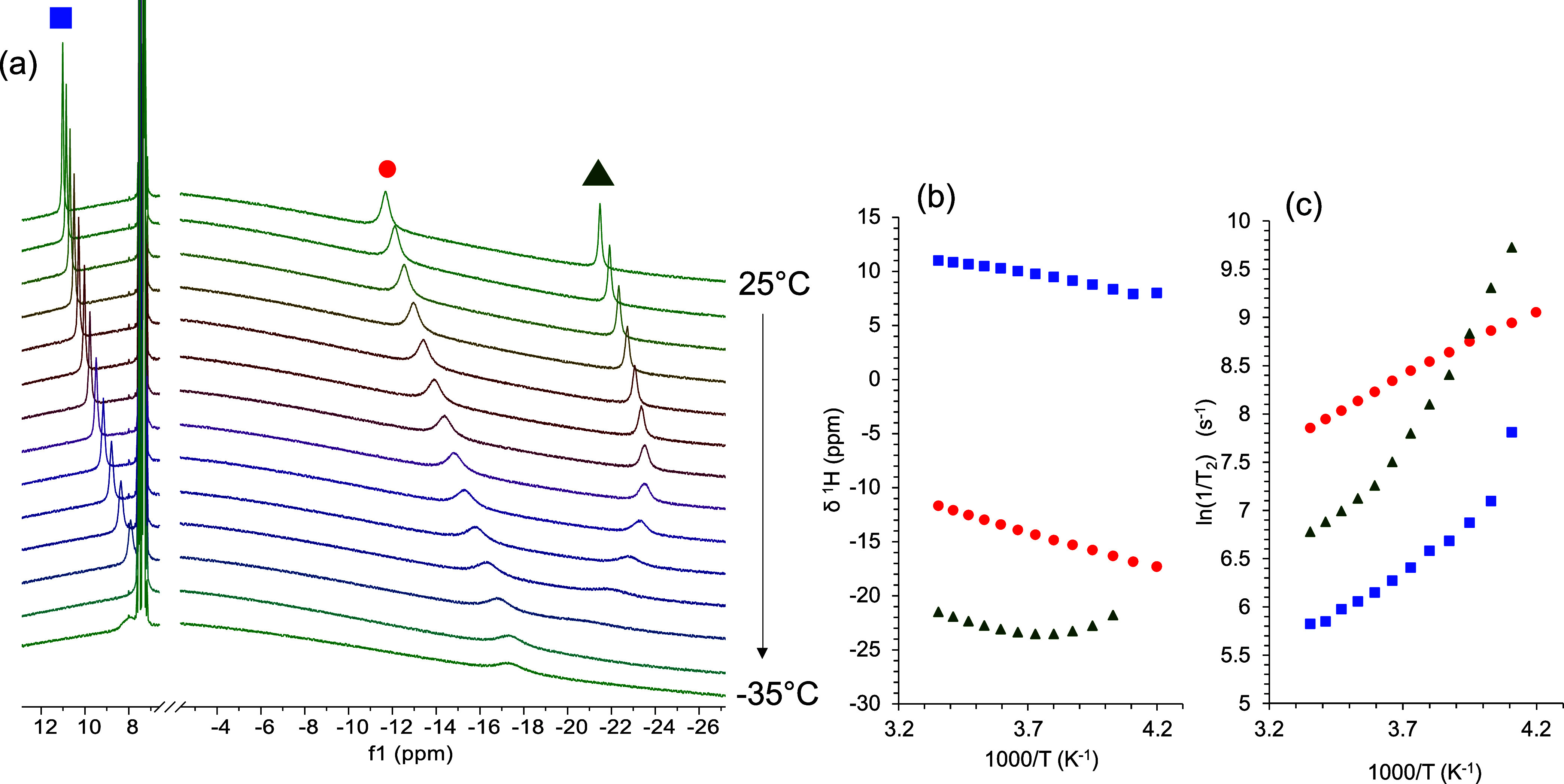
(a) Stack plots of ^1^H NMR spectra for **2** + PhSiH_3_ in MeOH-*d*_4_ in the temperature range from 25 to −35 °C; (b) temperature dependence of the chemical shifts for paramagnetic signals displayed in panel (a); and (c) evolution of transverse relaxation rates 1/*T*_2_ of the three resonance signals in panel (a).

To get more insight into the nature of cobalt species formed by the reaction of **2** with PhSiH_3_, we measured ^1^H NMR spectra in MeOH-*d*_4_ in the temperature window from 25 to −35 °C and investigated the temperature dependence of the chemical shifts and the relaxation times of three out of the four prominent resonances. The fourth signal, at 4.20 ppm, was not involved in this analysis, as it partially overlapped with other resonances. A strong temperature dependence of the chemical shifts and a significant resonance line broadening upon temperature decrease^134,135^ (Figure 7) indicated the presence of the paramagnetic Co(II) species.^136−140^

Given the reducing properties of phenylsilane, the formation of Co(II) complex **1** was considered likely. The comparison of the ^1^H NMR spectrum of **1** in MeOH-*d*_4_ with the NMR spectrum of the reaction system **2** + PhSiH_3_ at the same temperature showed the coincidence of the two broad resonances of **1** with the two of the four resonances observed for the reaction mixture **2** + PhSiH_3_ (Figure S83). In addition, SCD_3_ labeling allowed for the identification of the SCH_3_ resonances. Furthermore, integration of the resonances permitted the assignment of another signal to methyl groups of the Hacac moiety. These findings indicate that complex **1** is one of the products of the reaction of **2** with PhSiH_3_, while the other two resonances present in the ^1^H NMR spectrum likely arise from another paramagnetic Co(II) species formed. Attempts to identify this second species were successful as well.

### Further Investigation of Potentially Catalytically Active Cobalt Complexes and Ligand Noninnocence

**Figure 8:**
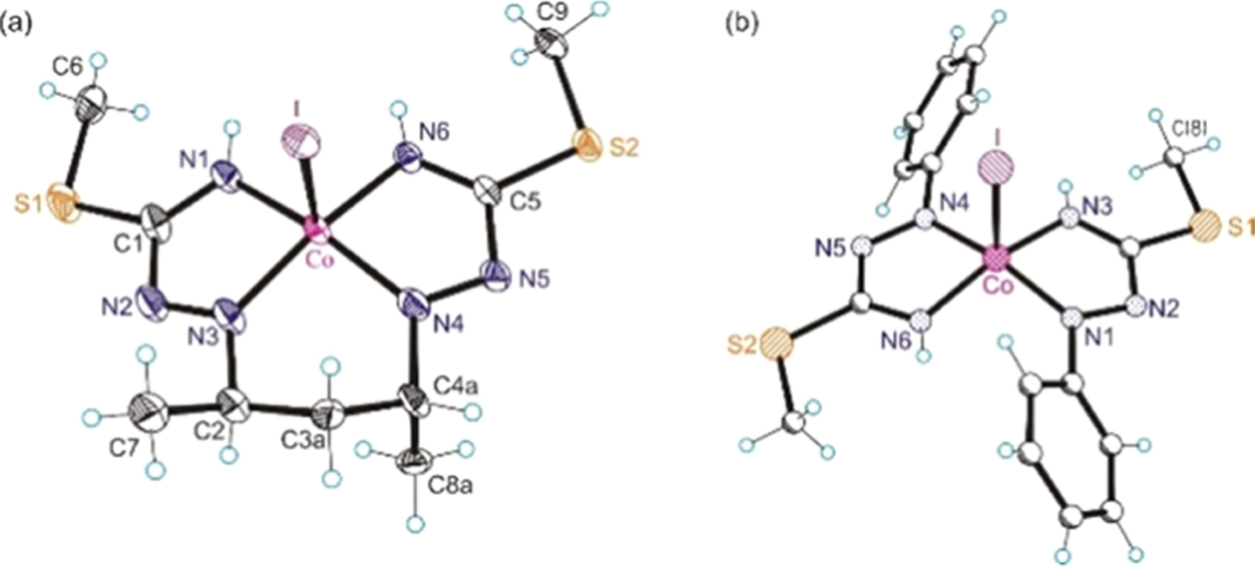
(a) ORTEP view of the Co(III) complex [Co^III^(L^SMe••^)I] (**13**) with a trianionic ligand diradical with the atom labeling scheme and thermal ellipsoids drawn at 50% probability level; and (b) ball-and-stick view of the Co(III) complex [Co^III^(Q^Me•^)_2_I].^141,142^

**Scheme 7:**
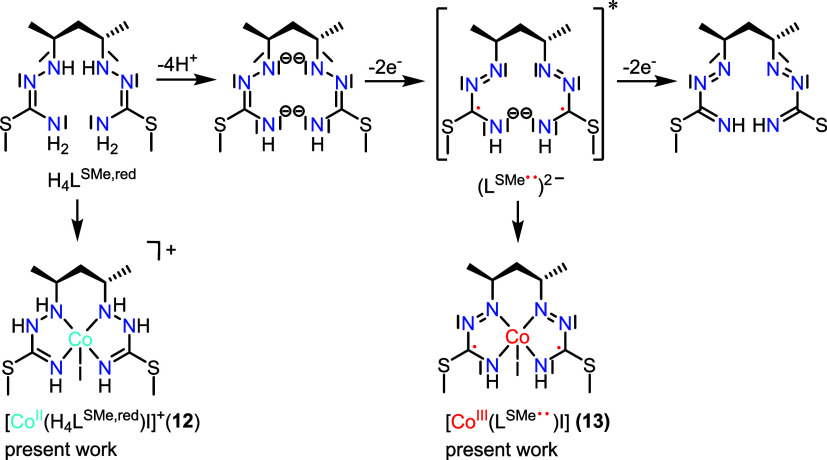
Possible PCET in the Oxidation Processes of the Reduced Phenylsilane Complex; * Only One of Three Resonance Forms of (L^SMe••^)^2–^ is Shown; All Three Resonance Structures Are Displayed in Chart S1 (see Section S8)

The reaction of **2** with PhSiH_3_ (1 equiv) in MeCN in a closed NMR tube after long standing at room temperature generated green crystals of X-ray diffraction quality, which were studied by SC-XRD and confirmed the formation of complex **12** (Scheme 7 and Section S8, Figure S84). The reduced reaction mixture of **2** with PhSiH_3_ (1 equiv) in a closed NMR tube in MeOH-*d*_4_ was allowed to stand at room temperature with very slow diffusion of air into the closed tube with the formation of a violet-red solution at the top of the tube (see Section S8, Figure S85) from which single crystals of X-ray diffraction quality of **13** were isolated and investigated by SC-XRD. The result is shown in Figure 8a.

The comparison of the metrical parameters in **13** ([Co^III^(L^SMe••^)I], Figure 8a) with those in a previously described square-pyramidal Co(III) complex with two 1-phenylisothiosemicarbazide monoanionic ligand radicals *trans*-[Co^III^(Q^Me•^)_2_I] (Figure 8b) led to the conclusion that a diradical species must be present.^142^ This implies that the investigated crystals have been obtained by air oxidation of **12**, forming a dianionic diradical Co(III) species **13**. The collected evidence suggests that this last species resulted from 2e^–^ oxidation of Co(II) complex **12** and release of four protons from the ligand (Scheme 7).

As the precise localization of the unpaired electrons of the diradical species **13** was not clear, further comparison with literature precedent^138^ was performed. *S*-Methyl-1-phenyl-isothiosemicarbazide (H_2_Q^Me^), in the presence of air oxygen, was recently shown to bind in a *N*,*N*-mode to cobalt(III) (as well as nickel(II) and iron(III)) in the 1e^–^ oxidized form as the monoanionic ligand radical (Q^Me•^)^1–^.^141−143^

**Chart 2:**
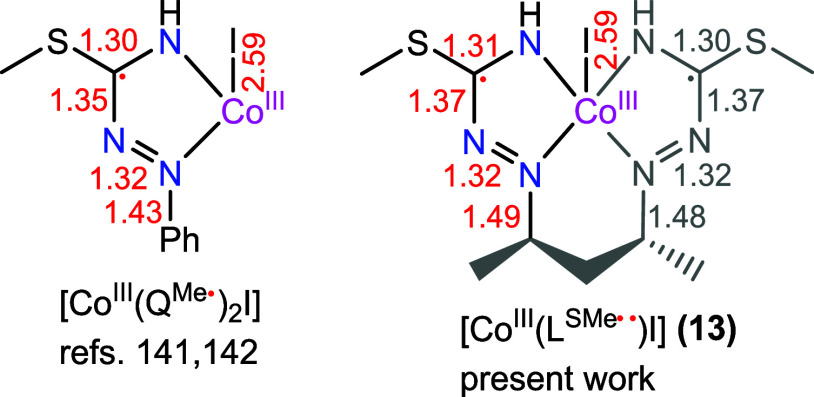
Metric Parameters of the Five-Membered Chelate Ring(s) in *trans*-[Co^III^(Q^Me•^)_2_I] from Refs (141,142) and in [Co^III^(L^SMe••^)I] (**13**) Reported Herein; Only One Resonance Structure is Shown for Both Complexesa

Comparison of the two C–N and N–N bond distances in the ligand in complex [Co^III^(Q^Me•^)_2_I]^139,140^ (Figure 8b) and in **13** (Figure 8a) revealed close similarity of the corresponding metrical parameters, as shown in Chart 2. Based on this, we can assign the oxidation level of the ligand as π-diradical dianion (L^SMe••^)^2–^.^144^

a Adapted partly from ref (142), American Chemical Society, Copyright (2004).

**Figure 9:**
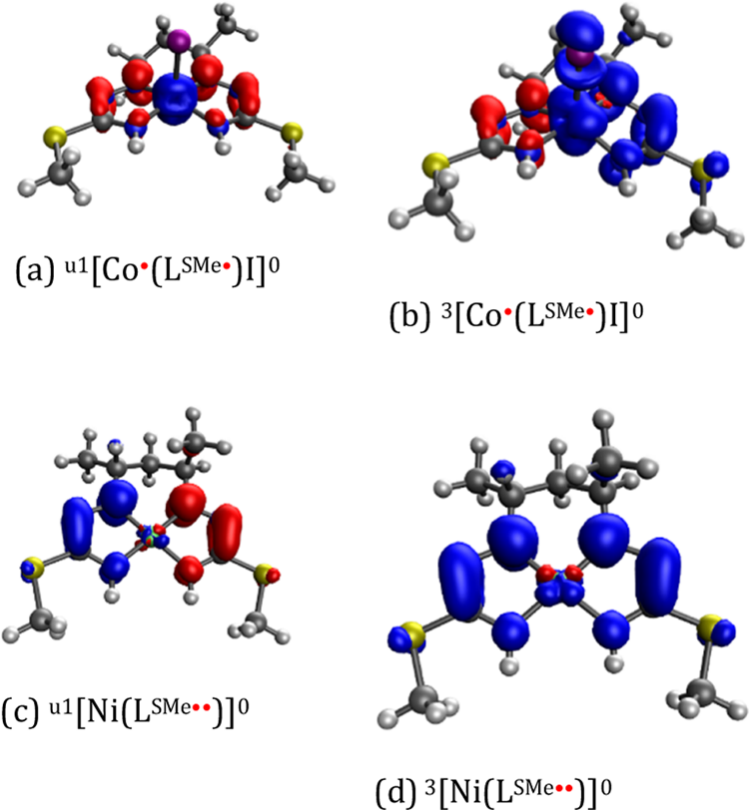
Spin density of chosen species [Co^•^(L^SMe•^)I] and [Ni(L^SMe••^)] studied; the isosurface value is 0.004 e·bohr^–3^.

To further corroborate our findings, DFT calculations of **13** were performed. For this, we considered two possible scenarios: (i) both unpaired electrons of the diradical species could be situated on the ligand (as indicated in **13**—[Co^III^(L^SMe••^)I]) or (ii) an electron each could be localized on the ligand and on cobalt ([Co^•^(L^SMe•^)I]). Broken-symmetry singlets (u1) with antiparallel spins on the central Co ion and the ligand in [Co^•^(L^SMe•^)I], resulted from DFT calculations, providing further evidence for a diamagnetic ground state *S* = 0 (see Figure 9 and Section S8, Table S11), as was also the case for *trans*-[Co^III^(Q^Me•^)_2_I], which gives a ^1^H NMR spectrum typical for diamagnetic compounds (see Section S8, Figure S86). Here, we have to emphasize that the Co oxidation state is rather unresolved and/or an intermediate oxidation state. One reasonable resonance structure (bonding picture) to mention is likely a Co^II^ with a strong π back-donation to the ligand that results in the unresolved oxidation state. This has been further confirmed in the absence of iodido ligand (see Section S8, Table S11). In contrast, by calculation, we observed a ligand–biradical interaction in the case of other diamagnetic complexes [Ni(L^SMe••^)] (Figure 9 and Table S11) and [Zn(L^SMe••^)] (Table S11), where the existence of antiferromagnetic *S* = 0 states was previously reported for a similar square-planar complex [Ni^II^(Q^Me•^)_2_].^86^

### Other Mechanistic Considerations

Complex **3** was shown to be reduced to **1** by 1 equiv of PhSiH_3_ in methanol and reoxidized by slow air oxygen supply back to **3**, as confirmed by SC-XRD.

**Scheme 8:**
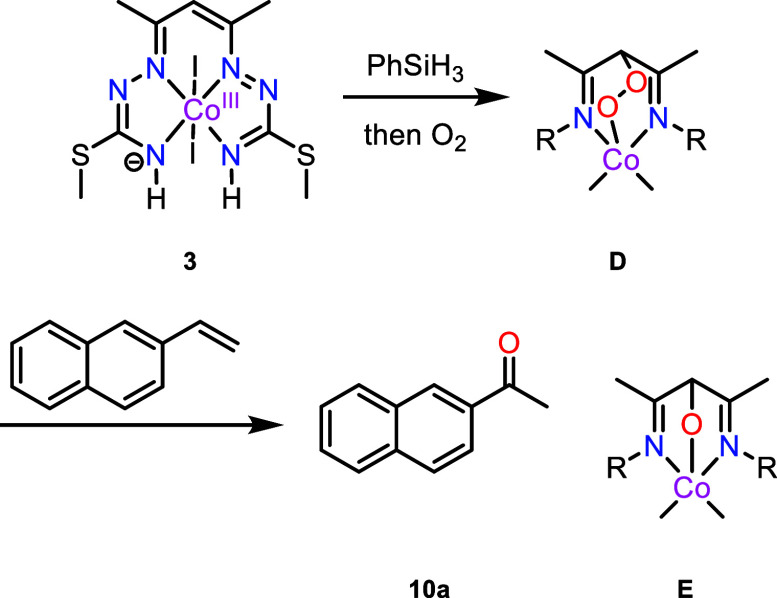
Proposed Intermediate **D** Presumably Involved in Catalysis

Formation of dimeric di-Co(III) complex [Co^III^(L^SMe,O^)I]_2_ (**4**) and its monomeric counterpart [Co^III^(L^SMe,O^)I(CH_3_OH)] (**5**) (see Section S2 and Scheme S2) is assumed to occur via activation of molecular oxygen by square-planar or square-pyramidal Co(II) complexes with the formation of intermediate Co(III) peroxido species (**D**, Scheme 8).^145^ This is supported by reported ^18^O_2_ labeling experiments with redox-active copper(II) complexes, which revealed that the oxygen atom in the ketone diimine product originated from molecular oxygen.^146^ Additionally, activation of O_2_ by five-coordinate Pt(IV) complexes was recently disclosed to occur with the involvement of both the electrophilic metal center and a Lewis basic site on the central carbon atom of Hacac, as concluded from the X-ray diffraction structure of a peroxido complex.^147^ These Pt(IV) peroxido complexes were shown to be effective oxidants of organic substrates, e.g., PPh_3_, Me_2_S, or CO. Addition of molecular oxygen to both a cobalt center and a ligand radical with the formation of a Co(III)-peroxido species was also confirmed quite recently by X-ray diffraction.^148,149^

Two identified species from the reaction mixture of **2** with 1 equiv of PhSiH_3_ appear to be of primary importance for the investigated catalytic transformations. The first, the cobalt(II) complex generated by the reduction of **2** or **3** with PhSiH_3_, namely, **1**, was found to catalyze the formation of nascent hydrogen. The dehydrogenative reaction of PhSiH_3_ with MeOH/MeOH-*d*_4_ was found to be minor in the absence of complex **2**, as confirmed by both ^1^H and ^29^Si NMR spectral measurements, as discussed previously. The produced nascent hydrogen is likely involved in the reduction of two azomethine bonds in **1** with the formation of a second paramagnetic Co(II) species, shown as [Co^II^(H_4_L^SMe,red^)I]I (**12**) in Scheme 7, Figure S84, and Scheme S2. This latter complex can be considered as a reservoir of H equivalents stored in the form of C–H/N–H bonds and as a source of H incorporated into the olefin oxidation products. Exposure of both of these species to oxygen afforded complexes with a ligand oxidized at the central carbon atom of the Hacac moiety, i.e., [Co^III^(L^SMe,O^)I(CH_3_OH)] (**5**), or generation of complex [Co^III^(L^SMe••^)I] (**13**). The formation of **5** might occur via a transient Co^III^-peroxido complex (**D**, Scheme 7) in which the O_2_^2–^ group bridges the central carbon atom of the Hacac moiety with Co^III^. **D** could be involved in intramolecular oxidation of the ligand or in intermolecular oxygen-atom transfer, as reported for other Pt(IV) complexes (vide supra). The role of the second species, **13** [Co^III^(L^SMe••^)I], in the catalytic transformations is still to be elucidated. However, identification of this species as a cobalt(III) complex with a π-diradical dianionic ligand with a spin ground state *S* = 0 has disclosed a new facet of redox chemistry for the PBIT platform, unprecedented reduction of the two azomethine bonds, and redox noninnocent behavior of the reduced ligand H_4_L^SMe,red^ upon oxidation.

## Conclusions

Access to cobalt complexes with the chemically and redox noninnocent PBIT ligand and their comprehensive characterization are reported. The factors that make the PBIT system particularly attractive as a platform for disclosure of unexplored chemistry include the capacity to stabilize Co and the ligand in two different physical oxidation states at relatively modest potentials, along with an impressive flexibility in coordination numbers and geometry, as well as the adopted protonation states and reactivity of the ligands. Several discernible protonation states of the ligand have been elucidated by spectroscopic and SC-XRD analyses of cobalt(II) and cobalt(III) complexes. The ligand in [Co^III^(*L*^*SMe*^)I_2_] proved to be a 12π electronic monoanionic system resulting from 2e^–^ oxidation of the 14π trianionic ligand (L^SMe^)^3–^. The oxidation states +3 for Co and −1 for the tetradentate ligand in the six-coordinate di-iodido complex [Co^III^(*L*^*SMe*^)I_2_] were corroborated by X-ray absorption spectra and inspection of metrical parameters in the PBIT ligand resulting from SC-XRD studies. Slow oxidation of complex [Co^II^(H_4_L^SMe,red^)I]I (**12**) obtained by reduction of [Co^III^(H_2_L^SMe^)I]I·CH_3_OH with 1 equiv of PhSiH_3_ in methanol afforded an unprecedented cobalt(III) complex with a dianionic diradical ligand formulated as [Co^III^(L^SMe••^)I] (**13**). The ligand in **12** was found to remotely incorporate four equivalents of hydrogen when compared to the coordinated ligand H_3_L^SMe^ (Scheme S1), which are stored in the form of C–H or N–H bonds, a thus far unexplored feature of the PBIT platform. Effective hydrogen storage implies reversible addition and release of H atoms,^150^ which still has to be confirmed. Multiproton-responsive ligands^151^ have already been successfully used in important chemical transformations and are expected to be valorized in the future. The described complexes, in combination with PhSiH_3_ as the reductant, ambient air as the oxidant, and ethanol as the solvent, offer a simple catalytic system for the chemo- and regioselective oxidation of styrene-like olefins to their corresponding ketones. The transformations proceed at room temperature and with good efficiency, with a low precatalyst loading (down to 0.5 mol %). In addition to a broad functional group tolerance, the established protocol proved to be suitable for late-stage oxidation of molecules derived from natural products. As catalytically competent species, the cobalt(II) complex with a fully reduced ligand, [Co^II^(H_4_L^SMe,red^)I]I, and cobalt(III)–peroxido complex have been suggested based on insights into the mechanism of the catalytic oxidation of olefins. Attempts to spectroscopically identify and characterize the cobalt(III)-peroxido complex by SC-XRD are ongoing in our laboratory.

## Methods

### General Procedure for the Cobalt-Catalyzed Wacker-Type Oxidation

The olefin substrate (0.5 mmol, 1.0 equiv; either neat, followed by EtOH (4 mL) or as a solution in EtOH (4 mL)) was added to a vial containing the Co precatalyst **3** (2.9 mg, 1 mol %) and a stirring bar. The mixture was then stirred at 1000 rpm, without being in direct contact with the stirring plate. After 1 min, phenylsilane (62 μL, 0.5 mmol, 1.0 equiv) was added and the reaction mixture was stirred for 18 h at room temperature (22–27 °C) under ambient atmosphere. Following full conversion of the olefin, as determined by TLC analysis of the reaction mixture, volatile components were removed under reduced pressure, and the resulting crude material purified by flash chromatography on silica gel using a suitable solvent system.

## Supporting Information Available

Synthesis, experimental, and computational details; further characterization data of the complexes (CHN combustion analyses, ^1^H NMR, UV–vis–NIR electron absorption spectra, additional XAS data with associated TDDFT calculations), computational results, single-crystal X-ray crystallographic data, and details of data collection along with the characterization of 32 oxidation products resulting from catalytic oxidation reactions (PDF)
