# Supplementary material for: Chemical and Redox Noninnocence of Pentane-2,4-dione Bis(S-methylisothiosemicarbazone) in Cobalt Complexes and Their Application in Wacker-Type Oxidation
Source: JACS Au. 2024 Mar 12;4(3):1166–83. doi: 10.1021/jacsau.4c00005 (PMC10976605; doi:10.1021/jacsau.4c00005)
Supplement: Supplementary file 1 — au4c00005_si_001.pdf [file au4c00005_si_001.pdf]

Supporting Information

for

Chemical and Redox Non-innocence of Pentan-2,4-dione Bis-(*S*-methylisothiosemicarbazone) in Cobalt Complexes and their Application in a Wacker-Type Oxidation

Vincent Porte,[§†] Miljan N. M. Milunovic, [§≠] Ulrich Knof,[‡] Thomas Leischner,[†] Tobias Danzl,[†] Daniel Kaiser,[†] Tim Gruene,[≠] Michal Zalibera,[#] Ingrid Jelemenska,[#] Lukas Bucinsky,[#] Sergio A. V. Jannuzzi,[▽] Serena DeBeer,[▽,*] Ghenadie Novitchi,[∮] Nuno Maulide,[†,*] and Vladimir B. Arion[≠,*]

[†]*University of Vienna, Institute of Organic Chemistry, Währinger Strasse 38, A-1090 Vienna, Austria*

[≠]*University of Vienna, Institute of Inorganic Chemistry, Währinger Strasse 42, A-1090 Vienna, Austria*

[‡]*Unterm Schellenberg 30, CH-4125 Riehen, Switzerland*

[#]*Institute of Physical Chemistry and Chemical Physics, Faculty of Chemical and Food Technology, Slovak University of Technology in Bratislava, Radlinského 9, SK-81237 Bratislava, Slovak Republic*

[▽]*Max Planck Institute for Chemical Energy Conversion, Stiftstraße 34–36, 45470 Mülheim an der Ruhr, Germany*

[∮]*CNRS-LNCMI, 38042 Grenoble Cedex, France*

Contents

General Scheme 5

Section 1: Synthesis and Characterization of Co complexes 5

Starting Materials 5

Cobalt complexes 1–3 5

Other cobalt(III) complexes 7

Other cobalt(II) complexes 8

Section 2 : Single Crystal X-ray Crystallography 12

Other cobalt(III) complexes 12

Other cobalt(II) Complexes 14

Section 3: X-ray Absorption Spectroscopy 21

Section 4: Spectroscopic and Magnetic Characterization of the complexes 23

UV-vis-NIR Spectroscopy and TD transitions 23

NMR spectroscopy of the cobalt complexes 27

Powder diffraction of complex 1 30

Magnetism of complex 1 31

EPR spectroscopy of complexes 1 and 8 34

Section 5: Electrochemistry and DFT calculations 36

Section 6: Computational Studies 41

Section 7: Catalytic Studies 47

Starting material synthesis 47

9q 47

9r 49

9w 51

9x 53

9y 55

9z 57

9aa 61

9ab 63

9ac 65

9ad 67

Oxaprozin 69

9ae 71

9af 73

Synthesis of the ketone products 75

General procedure 75
10a 76
10b 78
10c 79
10d 80
10e 81
10f 82
10g 83
10h 84
10i 85
10j 86
10k 87
10l 88
10m 89
10n 90
10o 91
10p 92
10q 93
10r 94
10s 95
10t 96
10u 97
10v 98
10w 100
10x 101
10y 102
10z 103
10aa 104
10ab 105
10ac 106
10ad 108
10ae 110
10af 112
Mechanistic studies 114
10ae – TEMPO adduct 114

$PhSiD_3$ 116
[D]-10a 118
The chemical transformations of $PhSiH_3$ in the presence of 2 followed by ^{1}H NMR spectroscopy 119
Section 8: Further investigation of catalytically active Co-species 123
References 126

General Scheme

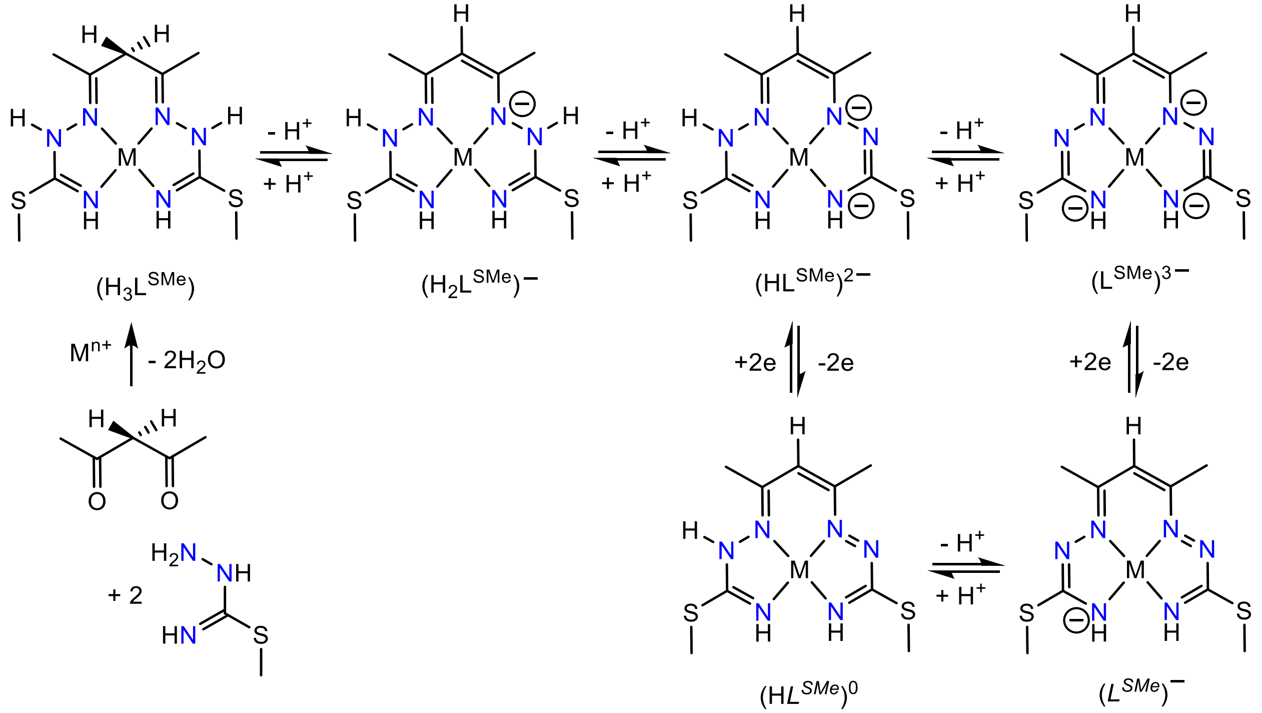

Scheme S1. Protonation steps for the 14 π-electron species $(L^{SMe})^{3-}$ and its 2-electron oxidised 12 π-electron species $(\mathit{L}^{SMe})^{-}$; adapted from ref. 1.

Section 1: Synthesis and Characterization of Co complexes

<u>Starting Materials</u>

S-methylisothiosemicarbazidium iodide was prepared as reported in the literature,[2] while *S*-methylisothiosemicarbazidium chloride by metathesis reaction using anionic exchange resin.

<u>Cobalt complexes 1–3</u>

$[Co^{II}(H_2L^{SMe})]I\cdot 0.5CH_3OH$ ($1\cdot 0.5CH_3OH$). To $Co(OAc)_2\cdot 4H_2O$ (2.5 g, 10.0 mmol) in methanol (25 ml) by purging argon through the solution were added *S*-methylisothiosemicarbazidium iodide (4.66 g, 20.0 mmol) in methanol (15 ml) and pentan-2,4-dione (1.2 ml, 12.0 mmol) and heated in oil bath at 80 °C under Ar atmosphere. As soon as fine crystals (cherry-brown) were clearly seen, the reaction mixture was allowed to cool slowly to room temperature. The solvent was removed under argon flow, the violet-brown precipitate was washed with anoxic methanol and dried in vacuo. Yield: 1.54 g. Calcd for $C_9H_{17}CoIN_6S_2\cdot 0.5CH_3OH$ (M_r = 475.26) %: C, 24.01; H, 4.03; N,

17.68; S, 13.49. Found, %: C, 23.86; H, 4.14; N, 17.21; S, 13.25. ESI-MS (+): *m/z* 345.10; calculated for $[Co^{III}(L^{SMe,O})]^+$ or $[C_9H_{14}CoN_6S_2O]^+$ 345.00; *m/z* 331.15; calcd for $[Co^{III}(HL^{SMe})]^+$ or $[C_9H_{16}CoN_6S_2]^+$; *m/z* 331.02. ESI-MS (–): *m/z* 598.77 (strong); calculated for $[\{Co^{III}(L^{SMe,O})I_2\}]^-$ or $[(C_9H_{14}CoI2N_6S_2O)]^-$ 598.81; *m/z* 584.83; calculated for $[Co^{III}(HL^{SMe})I_2]^-$ or $[C_9H_{16}CoN_6S_2I_2]^-$ 584.83; *m/z* 126.90 (I^-). Selected IR peaks (ATR, cm^{-1}, vs = very strong, br = broad, s = strong, m = medium, w = weak): 3366 (m), 3286 (vs), 3159 (br, vs), 1519 (s), 1505 (s), 1429 (s), 1361 (s), 1322 (s), 1305 (s), 1159 (s), 1057 (w), 1025 (s), 960 (w), 864 (w), 784 (w), 740 (m), 698 (m), 599 (w). UV-Vis-NIR λ_{max} [nm] (ε [$M^{-1}cm^{-1}$]): 222 (21000), 300 (9000), 357 (4950), 578 (450).

$[Co^{III}(H_2L^{SMe})I]I\cdot CH_3OH$ (2·CH_3OH). To $Co(OAc)_2\cdot 4H_2O$ (2.5 g, 10.0 mmol) in methanol (25 ml) by purging argon through the solution were added *S*-methylisothiosemicarbazidium iodide (4.66 g, 20.0 mmol) in methanol (15 ml) and pentan-2,4-dione (1.2 ml, 12.0 mmol) and heated in oil bath at 80 °C under Ar atmosphere. As soon as fine crystals (cherry-brown) were clearly seen on the Schlenk flask wall the reaction mixture was allowed to cool slowly to room temperature with exposure to air oxygen for 10 min. The color from brown-red turns into green-blue. This solution was filtered through filter paper in air and allowed to stand at room temperature in a closed round-bottom flask. Next day the crystals formed were filtered in air, washed with methanol and dried in air. Yield: 0.72 g. Calcd for $C_9H_{17}CoI2N_6S_2\cdot CH_3OH$ (M_r = 618.19) %: C, 19.43; H, 3.42; N, 13.59; S, 10.37. Found, %: C, 19.64; H, 3.38; N, 13.53; S, 10.72. ESI-MS (+): *m/z* 459.01 (strong); calculated for $[Co^{III}(H_2L^{SMe})I]^+$ or $[C_9H_{17}CoN_6S_2I]^+$ 458.93; *m/z* 332.03; calcd for $[Co^{II}(H_2L^{SMe})]^+$ or $[C_9H_{17}CoN_6S_2]^+$; *m/z* 332.14. ESI-MS (–): *m/z* 598.77 (strong); calculated for $[\{Co(L^{SMe,O})I_2\}]^-$ or $[(C_9H_{14}CoIN_6S_2O)I]^-$ 598.81; *m/z* 584.80 (strong); calcd for $[Co^{III}(HL^{SMe})I_2]^-$ or $[C_9H_{16}CoN_6S_2I_2]^-$ 584.83; *m/z* 126.90 (I^-). Selected IR peaks (ATR, cm^{-1}, vs = very strong, br = broad, s = strong, m = medium, w = weak): 1580 (br), 1488 (s), 1353 (m), 1275 (m), 1134 (vs), 997 (m), 926 (s), 848 (m), 711 (w), 655 (w). UV-Vis-NIR λ_{max} [nm] (ε [$M^{-1}cm^{-1}$]): 250 (17400), 288 (11600), 324 (8400), 367 (4700), 638 (2150), 1063 (450). Complex **2** to be used for further transformation into **3** should be stored in a Schlenk tube under inert atmosphere, better in the fridge.

$[Co(L^{SMe})I_2]$ (3). Freshly prepared $[Co^{III}(H_2L^{SMe})I]I\cdot CH_3OH$ (250 mg) was dissolved by using an ultrasound bath in ethanol (150 ml) at 36–40 °C. The solution was filtered and the solvent

evaporated to ca. 60-65 ml. Next day green-brown crystals with a characteristic metallic glance were filtered off, washed with ethanol and dried in air. Yield: 150 mg. Calcd for $C_9H_{15}CoI_2N_6S_2$ (M_r = 584.13) %: C, 18.51; H, 2.59; N, 14.39; S, 10.98. Found, %: C, 18.76; H, 2.47; N, 14.07; S, 10.90. ESI-MS (+) in MeCN: *m/z* 584.80 $[Co^{III}(L^{SMe})I_2+H]^+$ (calculated *m/z* 584.83), *m/z* 458.93 $[Co^{III}(H_2L^{SMe})I]^+$ (calculated *m/z* 458.93). ESI-MS (–): *m/z* 126.75 $[I]^-$, *m/z* 380.68 $[I_3]^-$, *m/z* 582.78 $[Co^{III}(L^{SMe})I_2–H]^-$ (calculated *m/z* 582.81). Selected IR peaks (ATR, cm^{-1}, vs = very strong, br = broad, s = strong, m = medium, w = weak): 3328 (s), 1492 (s), 1471 (m), 1376 (w), 1326 (s), 1235 (s), 1135 (vs), 1048 (m), 996 (vs), 923 (vs), 853 (s), 652 (w), 594 (w). The complex is diamagnetic in the solid state according to magnetic susceptibility measurements from 2 to 300 K at 1 T. UV-Vis-NIR λ_{max} [nm] (ε $[M^{-1}cm^{-1}]$): 295 (17500), 357sh, 457 (4500), 510sh, 626 (1320), 1030 (1020), 1150 (1120)

Other cobalt(III) complexes

$[Co(L^{SMe,O})I]_2$·EtOH (4·EtOH). $[Co^{II}(H_2L^{SMe})]I·0.5CH_3OH$ (**1**·$0.5CH_3OH$) (0.26 g) in ethanol (300 mL) was stirred at room temperature for 5 h to give a clear brown solution. Ethanol (220 mL) was removed under reduced pressure, the concentrated solution was filtered and allowed to stand at room temperature. Next day the dark-red crystals were separated by filtration, washed with ethanol and dried in air. Yield: 50 mg. ESI-MS (+): *m/z* 816.83 (very weak); calculated for $[\{Co(L^{SMe,O})I\}][\{Co(L^{SMe,O})\}]^+$ or $[(C_9H_{14}CoN_6S_2O)_2I]^+$ 816.90; *m/z* 344.97 (strong); calcd for $[Co^{III}(L^{SMe,O})]^+$ or $[(C_9H_{14}CoN_6S_2O)]^+$ 345.00. ESI-MS (–): *m/z* 598.60 (strong); calculated for $[\{Co(L^{SMe,O})I_2\}]^-$ or $[(C_9H_{14}CoIN_6S_2O)I]^-$ 598.81; *m/z* 470.70 (weak); calcd for $[Co^{III}(L^{SMe,O}–H^+)I]^-$ or $[(C_9H_{13}CoN_6S_2O)I]^-$ 470.90. Selected IR peaks (ATR, cm^{-1}, vs = very strong, br = broad, s = strong, m = medium, w = weak): 3362 (m), 3314 (s), 3278 (m), 3193 (s), 1616 (s) (attributed to $\nu_{C=O}$), 1593 (s) (attributed to $\nu_{C=O}$), 1535 (s), 1498 (s), 1474 (vs) 1293 (s), 1210 (s), 1126 (vs), 1083 (s), 948 (s), 867 (s), 712 (m). UV-Vis-NIR λ_{max} [nm] (ε $[M^{-1}cm^{-1}]$): 246 (37500), 280sh, 305 (28600), 359 (26400), 528 (3050).

$[Co^{III}(L^{SMe,O})I(CH_3OH)]$ (5). To a solution of complex **2** (30 mg) in anoxic EtOH (4.5 mL) in a Schlenk tube under argon, $PhSiH_3$ (1 equiv) was added. The green reaction mixture changed color to yellow and after 10 min a precipitate was formed, which was filtered under argon, washed with anoxic EtOH and dried *in vacuo* overnight. In a glove box, the precipitate was transferred in the Young-tube, dissolved in degassed MeOH-d_4 or MeOH (0.6 mL). The solution was yellow, on the

next day became green and after a few days produced brown crystals with characteristic green glance. Complex $[Co^{III}(L^{SMe,O})I(CH_3OH)]$ was characterized by SC-XRD (vide infra).

$[Co^{III}(HL^{SMe})I]\cdot3CH_3OH$ (2'·3CH₃OH). In rare cases, the oxidation reaction of **1** produced, in addition to the main product **2**, a small amount of red crystals of X-ray diffraction quality of $[Co^{III}(HL^{SMe})I]\cdot3CH_3OH$ (**2'·$3CH_3OH$**) with a dianionic ligand (Figure S3). ^1H NMR (500 MHz, CD_3CN): *δ* 13 (bs, 1H, NH); 8.58 (bs, 2H, NH); 7.58 (s, 1H, C–H) 2.77(s, 6H, 2 x S-CH_3); 2.99 (s, 6H, CH_3) (Figure S9).

<u>Other cobalt(II) complexes</u>

To find out whether other coordination geometries and/or protonation levels of the PBIT ligand in cobalt(II) complexes can be reached, we prepared a series of Co(II) complexes.

The PBIT ligand in its fully protonated form was observed in the complex $[Co^{II}(H_3L^{SMe})I(CH_3OH)]I\cdot CH_3OH$ (**6·CH_3OH**).

$[Co^{II}(H_3L^{SMe})I(CH_3OH)]I\cdot CH_3OH$ (6·CH_3OH). The cobalt(III) complex **2** (10 mg) was dissolved in anoxic MeOH or MeOH-*d_4* (1 ml) and $PhSiH_3$ (2 equiv) was added under argon in a Schlenk tube. The solution changed color from green to yellow. The tube was allowed to stand at –20 °C. After two weeks crystals of a six-coordinate cobalt(II) complex $[Co^{II}(H_3L^{SMe})I(CH_3OH)]I\cdot CH_3OH$ (**6·CH_3OH**) were obtained and measured by SC-XRD (Figure S4).

$[Co^{II}(H_2L^{SMe})]Cl\cdot CH_3OH$ (7·CH_3OH). In the first 4 h of the synthesis of **8** (vide infra), an intermediate species $[Co^{II}(H_2L^{SMe})]Cl\cdot CH_3OH$ (**7·CH_3OH**) was formed. The structure was confirmed by SC-XRD analysis (Figure S6).

The PBIT ligand preserved its full protonated form also in the complex 8.

$[Co^{II}(H_3L^{SMe})Cl]_2[Co^{II}Cl_4]\cdot CH_3OH\cdot2H_2O$ (8·$CH_3OH\cdot2H_2O$). To $Co(OAc)_2\cdot4H_2O$ (1.25 g, 5.0 mmol) in methanol (12 ml) by purging argon through the solution were added *S*-methylisothiosemicarbazidium chloride (1.50 g, 10.6 mmol) in methanol (10 ml) and pentan-2,4-dione (0.6 ml, 6.0 mmol) and heated in oil bath at 80 °C under stirring. After about 15 min a pink precipitate appeared. The solution was allowed to cool down slowly to 40 °C in a closed Schlenk

tube under argon. As soon as the temperature of 40 °C was reached the stirrer was disabled and the reaction mixture was left to stand in oil bath at 40 °C overnight. Next day some amount of brown crystals was observed in the flask. The mixture was heated again to 80 °C under stirring and bubbling argon. Some amount of methanol was evaporated under these conditions (ca. 5–7 mL). The heating was switched off and the mixture was allowed to cool down to 40 °C in oil bath. Upon cooling the formation of a green precipitate was observed. One crystal was analyzed by SC-XRD. Green product crystallized was isolated next day and washed with degassed methanol (10 ml), dried and collected under argon. The collected filtrate was concentrated in vacuo and the green precipitate was isolated under argon, washed with 5 ml of methanol, dried in vacuo and placed under argon. Total yield: 0.8 g, 48.0%. Calcd for $C_{18}H_{36}Cl_6Co_3N_{12}S_4 \cdot CH_3OH \cdot 2H_2O$ (M_r = 1006.41), %: C, 22.67; H, 4.41; N, 16.70; S, 12.74. Found, %: C, 22.93; H, 4.14; N, 16.30; S, 12.34. UV-vis-NIR λ_{max} [nm] (ε [$M^{-1}cm^{-1}$]): 235 (59020), 301 (18000), 360 (11100), 392 (7920), 440 (3200).

$[Co^{II}(H_4L^{SMe,red})I]I \cdot MeCN$ (12·MeCN). To a suspension of complex **2** (15 mg) in degassed MeCN (2 mL), $PhSiH_3$ (1 equiv) was added under argon and the reaction mixture was stirred for 25 min at room temperature. Afterwards, the flask was sealed and allowed to stand at room temperature. After 8 weeks several large crystals (brown sticks-rectangles) were found, isolated and investigated by SC-XRD.

$[Co^{III}(L^{SMe\bullet\bullet})I]$ (13). To complex **2** (10 mg) in Young NMR tube under argon $PhSiH_3$ (1 equiv) and MeOH-d_4 (0.6 mL) were added. The tube was closed with cap and teflon-tape. After standing at room temperature for 2 days, the upper part of solution in the tube turned from yellow to violet due to slow diffusion of air into the upper part of the solution. After 5 days crystals of complex **14** were formed in the upper part of the solution, isolated and investigated by SC-XRD. A few additional crystallization attempts under the same conditions were performed and resulted in violet solution, but the quick air diffusion precluded the formation of crystals.

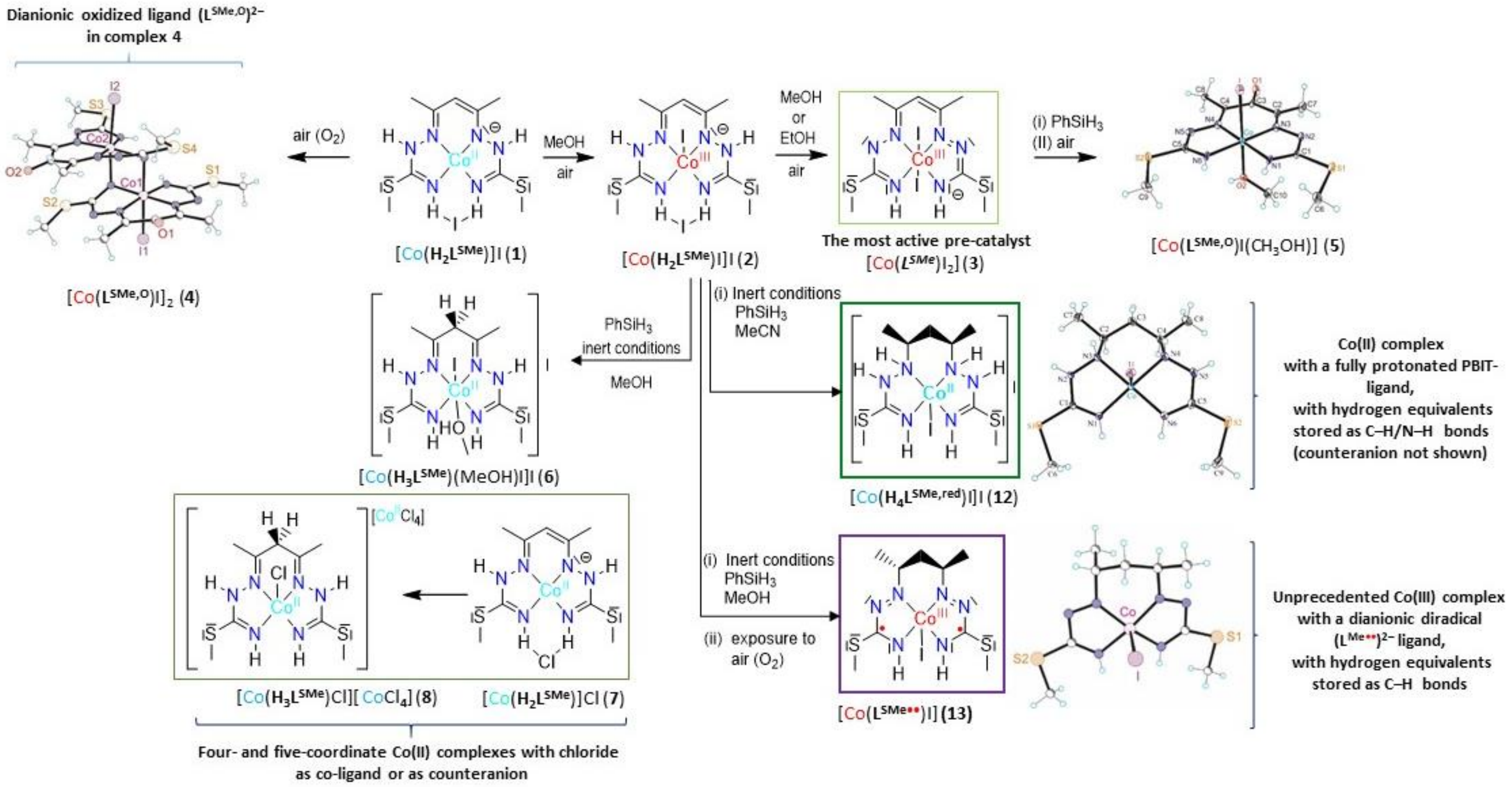

Scheme S2. Four-, five- and six-coordinate cobalt complexes with different levels of PBIT-ligand protonation and oxidation levels of Co and ligand.

Section 2 : Single Crystal X-ray Crystallography

X-ray diffraction quality single crystals of $[Co^{II}(H_2L^{SMe})]I\cdot 0.5CH_3OH$ **($1\cdot 0.5CH_3OH$)**, $[Co^{III}(H_2L^{SMe})I]I\cdot CH_3OH$ **($2\cdot CH_3OH$)**, $[Co(L^{SMe})I_2]$ **(3)**, $[Co(L^{SMe,O})I]_2\cdot C_2H_5OH$ **($4\cdot C_2H_5OH$)**, $[Co^{III}(L^{SMe,O})I(CH_3OH)]$ **(5)** $[Co^{II}(H_3L^{SMe})I(CH_3OH)]I\cdot CH_3OH$ **($6\cdot CH_3OH$)**, $[Co^{II}(H_2L^{SMe})]Cl\cdot CH_3OH$ **($7\cdot CH_3OH$)**, $[Co^{II}(H_3L^{SMe})Cl]_2[Co^{II}Cl_4]\cdot CH_3OH\cdot 2H_2O$, **($8\cdot CH_3OH\cdot 2H_2O$)**, and $[Co^{III}(HL^{SMe})I]\cdot 3CH_3OH$ **($2'\cdot 3CH_3OH$)**, $[Co^{II}(H_4L^{SMe,red})I]I\cdot MeCN$ **($12\cdot MeCN$)** and $[Co^{III}(L^{SMe\bullet\bullet})I]$ **(13)** were selected directly from the products prepared. The measurements were performed on Bruker D8 Venture, Bruker APEXII and STOE diffractometers. The data were processed using SAINT Plus[3] and STOE X-RED[4] softwares. Crystal data, data collection parameters, and structure refinement details are given in Tables S1–S3. The structures were solved by direct methods and refined by full-matrix least-squares techniques. Non-H atoms were refined with anisotropic displacement parameters. H atoms were inserted in calculated positions and refined with a riding model. The following computer programs and hardware were used: structure solution, *SHELXS-2014* and refinement, *SHELXL-2014*;[5] molecular diagrams, ORTEP;[6] computer, Intel CoreDuo. CCDC 2259330 (**$1\cdot 0.5CH_3OH$**), 2259333 (**$2\cdot CH_3OH$**), 2259332 (**3**), 2259338 **($6\cdot CH_3OH$)**, 2259331 **($9\cdot CH_3OH\cdot H_2O$)**, 2259340 **($8\cdot CH_3OH$)**, 2259341 **($2'\cdot 3CH_3OH$)**, 2259334 **($4\cdot C_2H_5OH$)**, 2312261 (**$12\cdot MeCN$**), 2259335 **(13)**, 2259337 **(5)**.

Other cobalt(III) complexes

The cobalt(III) complex $[Co^{III}(L^{SMe,O})I]_2\cdot C_2H_5OH$ **($4\cdot C_2H_5OH$)** is a dimeric associate of two essentially square-pyramidal cobalt complexes (Figure S1), in which each oxidized at central carbon atom dianionic ligand $(L^{SMe,O})^{2-}$ is coordinated in the base of the pyramid to Co(III) with one iodido co-ligand in apical position. The coordination geometry of each Co(III) is completed to six by involvement of a terminal NH group of the neighboring complex, which bridges between the two cobalt ions. The two interatomic distances Co1–N12 and Co2–N6 are remarkably short for this kind of bonds, indicating their covalent character, but still by 0.143 and 0.124 Å longer than in-plane bond lengths Co1–N6 at 1.933(5) and Co2–N12 at 1.944(5) Å. This agrees with ESI mass spectra, which showed a peak attributable to the dimeric structure.

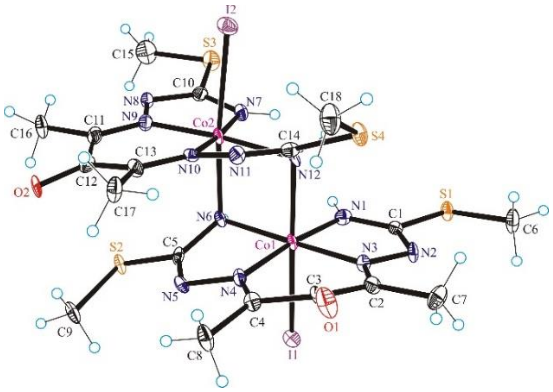

Figure S1. ORTEP view of complex **4·C_2H_5OH** with atom labeling schemes. Thermal ellipsoids are drawn at 50% probability level. Interstitial ethanol molecule is not shown.

A cobalt(III) complex [Co^{III}($L^{SMe,O}$)I(CH_3OH)] (**5**) with oxidized ligand ($L^{SMe,O}$) was obtained from diluted solutions upon reduction of **3** with $PhSiH_3$ in methanol and exposure to air oxygen (Figure S2).

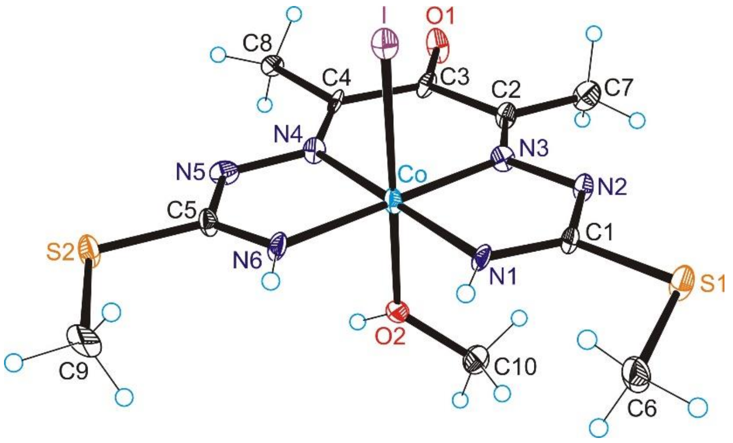

Figure S2. ORTEP view of the complex [Co^{III}($L^{SMe,O}$)I(CH_3OH)] **(5)** with atom labeling scheme. Thermal displacement parameters are drawn at 50% probability level.

In rare cases the oxidation reaction of **1** produced, in addition to the main product **2**, a small amount of red crystals of X-ray diffraction quality of square-pyramidal complex [Co^{III}(HL^{SMe})I]·3CH_3OH (**2’·3CH_3OH**), in which the tetradentate ligand acts as a dianion (Figure S3). Note that the dianionic form of the same ligand was previously documented in the iron complex with two redox non-innocent ligands NO and PBIT, namely [Fe(HL^{SMe})NO]NO_3.[7]

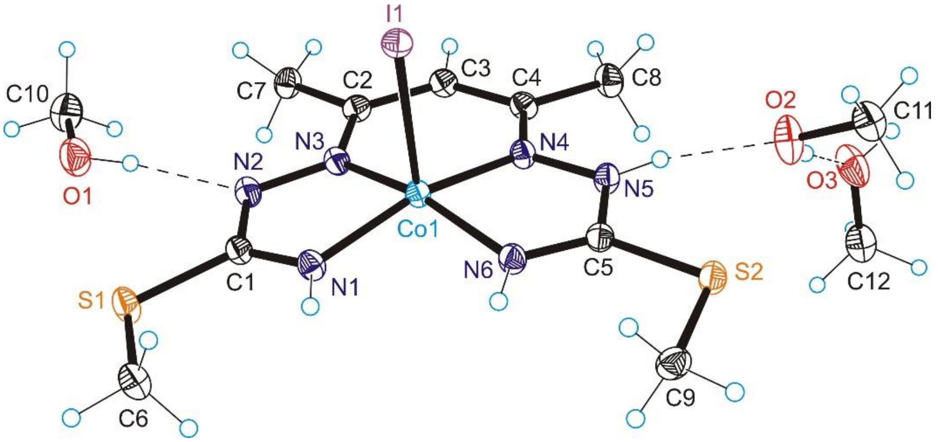

Figure S3. ORTEP view of the complex $[Co^{III}(HL^{SMe})I]\cdot 3CH_3OH$ **($2'\cdot 3CH_3OH$)** with atom labeling scheme. Thermal displacement parameters are drawn at 50% probability level.

Other cobalt(II) Complexes

A six-coordinate cobalt(II) complex $[Co^{II}(H_3L^{SMe})I(CH_3OH)]I\cdot CH_3OH$ **($6\cdot CH_3OH$)**, in which the ligand is fully protonated was obtained by reduction of complex **2** with $PhSiH_3$ in MeOH-*d4* under inert conditions (Figure S4).

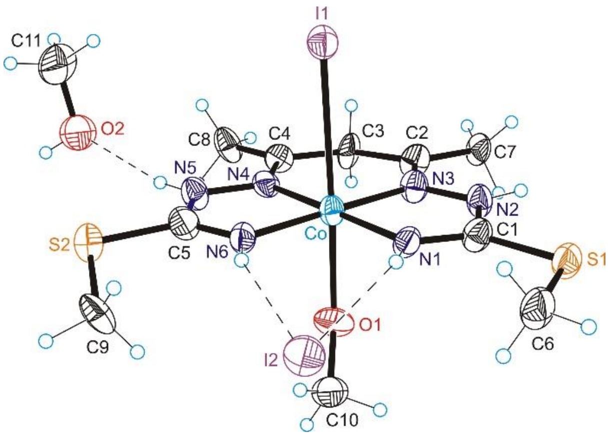

Figure S4. ORTEP view of the complex $[Co^{II}(H_3L^{SMe})I(CH_3OH)]I\cdot CH_3OH$ **($6\cdot CH_3OH$)** with atom labeling scheme. Thermal displacement parameters are drawn at 50% probability level.

A square-pyramidal cobalt(II) complex **8** with fully protonated ligand (H_3L^{SMe}) was obtained by using *S*-methylisothiosemicarbazidium chloride instead of *S*-methylisothiosemicarbazidium iodide. Cobalt(II) acetate with *S*-methylisothiosemicarbazidium chloride and Hacac in 1:2:1 molar ratio in methanol under inert atmosphere, accompanied by a gradual color change from brown-cherry to deep-green over 24 h, template assembly of PBIT was accomplished, which has been also isolated in its fully protonated form (H_3L^{SMe}) as green crystalline product of $[Co^{II}(H_3L^{SMe})Cl]_2[Co^{II}Cl_4]\cdot CH_3OH\cdot H_2O$ **($8\cdot CH_3OH\cdot H_2O$)** (Figure S5).

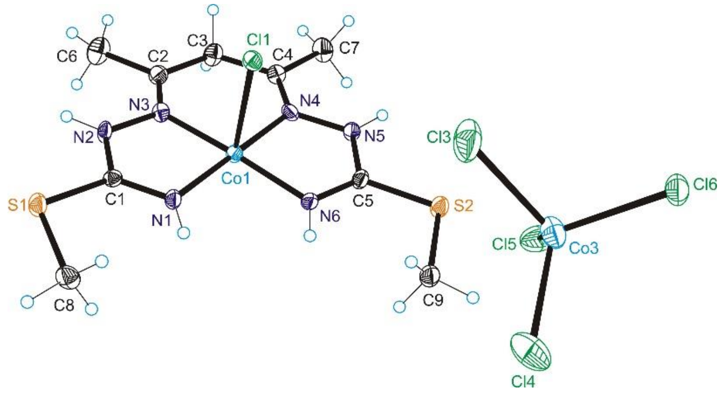

Figure S5. ORTEP view of one complex cation and complex anion in $[Co^{II}(H_3L^{SMe})Cl]_2[Co^{II}Cl_4]$ $\cdot CH_3OH \cdot H_2O$ **(8·CH_3OH·H_2O)** with atom labeling scheme. Thermal displacement parameters are drawn at 50% probability level.

The crystal of $[Co^{II}(H_3L^{SMe})Cl]_2[Co^{II}Cl_4] \cdot CH_3OH \cdot 2H_2O$ **(8·CH_3OH·$2H_2O$)** consists of two square-pyramidal complex monocations $[Co^{II}(H_3L^{SMe})Cl]^+$, in which the ligand is fully protonated, and one tetrahedral complex dianion $[Co^{II}Cl_4]^{2-}$ (Figure S5 shows one monocation), as well as of interstitial solvent molecules. The neutral form of the tetradentate ligand has been also identified in $[Zn(H_3L^{SMe})I]I$ by SC-XRD.[8] However, in that case the ligand is folded along C3–Zn vector as expected from the presence of sp^3-hybridized carbon atom in six-membered C_3N_2Zn ring. Flat bis(β-ketimine) rings have been reported for Ni(II) complexes with pentan-2,4-dione bis(dithiocarbazates) as well.[9]

The X-ray structure of the precursor cobalt(II) complex **7** is shown in Figure S6. The cobalt(II) complex adopts a square-planar coordination geometry, while the coordination environment is provided by a monoanionic tetradentate ligand $(H_2L^{SMe})^-$ with chloride as counteranion.

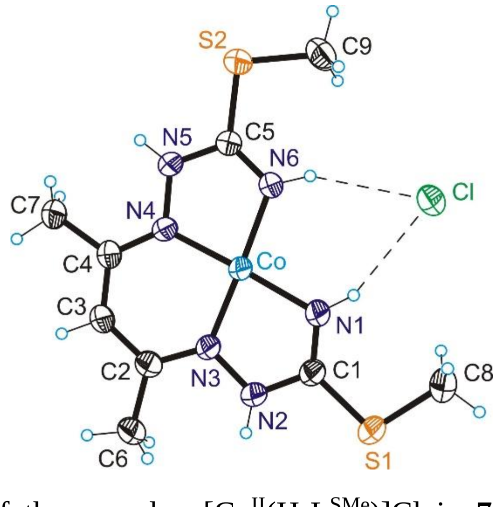

Figure S6. ORTEP view of the complex $[Co^{II}(H_2L^{SMe})]Cl$ in **7·CH_3OH** with atom labeling scheme. Thermal displacement parameters are drawn at 50% probability level. Interstitial methanol molecule is not shown.

Stack formation is a feature of complex **1·0.5CH$_3$OH**. This is worth noting given that only few cofacial dimers with Co^{II}–Co^{II} bond have been reported previously.[10,11,12] Relatively short distance between the two almost parallel complex cations in the asymmetric unit of **1·0.5CH$_3$OH** with a Co···Co separation of only ca. 3.147(2) Å, exhibiting a staggered geometry between the monomeric cations within the dimer was found (Figure S7). The pairs of dimers in **1·0.5CH$_3$OH** form stacks in the crystal with Co···Co contact of 3.801(2) Å. All odd number cations (the first and third in Figure S7) as well as all even number cations (the second and fourth) in the stack adopt eclipsed cofacial geometry, respectively.

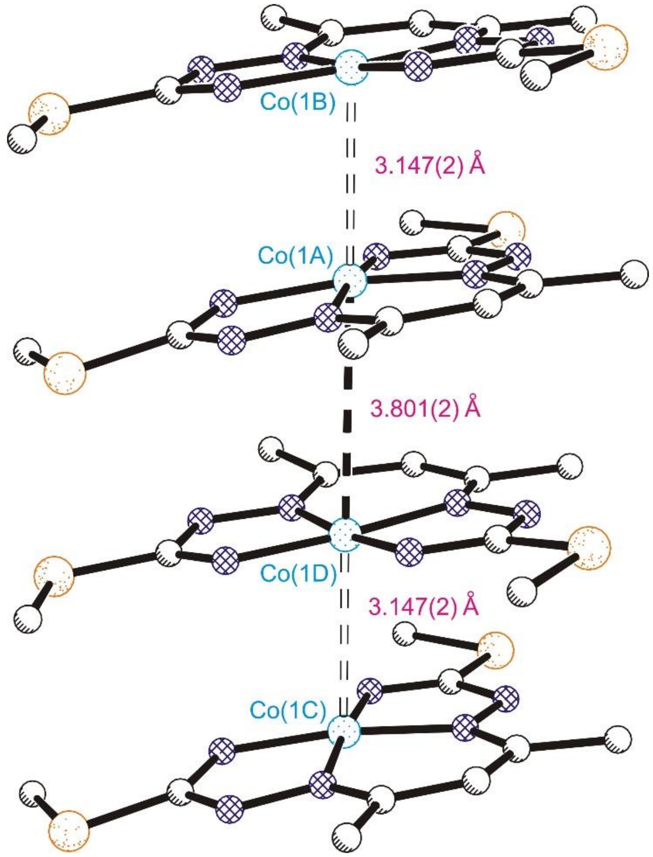

Figure S7. Stack formation in the crystal structure of **1·0.5CH$_3$OH**.

In contrast to **1·0.5CH$_3$OH** with iodide as counteranion the analogous chloride complex [Co^{II}(H$_2$L^{SMe})]Cl·CH$_3$OH **(7·CH$_3$OH)** in Figure S6 did not reveal any association of square-planar complexes in cofacial dimers.

Table S1. Crystal Data and Details of Data Collection for $[Co(H_2L^{SMe})]I\cdot0.5CH_3OH$ **(1·0.5CH₃OH)**, $[Co^{III}(H_2L^{SMe})I]I\cdot CH_3OH$ **(2·CH₃OH)**, $[Co^{III}(L^{SMe})I_2]$ **(3)**, $[Co^{II}(H_3L^{SMe})I(CH_3OH)]I\cdot CH_3OH$ **(6·CH₃OH)** and $[Co^{II}(H_3L^{SMe})Cl]_2[Co^{II}Cl_4]\cdot CH_3OH\cdot H_2O$ **(8·CH₃OH·H₂O)**.

Compound	**$1\cdot0.5CH_3OH$**	**$2\cdot CH_3OH$**	**3**	**$6\cdot CH_3OH$**	**$8\cdot CH_3OH\cdot H_2O$**
empirical formula	$C_{9.5}H_{19}CoIN_6O_{0.5}S_2$	$C_{10}H_{21}CoI_2N_6OS_2$	$C_9H_{15}CoI_2N_6S_2$	$C_{11}H_{26}CoI_2N_6O_2S_2$	$C_{19}H_{42}Cl_6Co_3N_{12}O_2S_4$
fw	475.26	618.18	584.12	651.23	988.37
space group	$Pna2_1$	$P2_1/n$	*Pnma*	$P2_1/n$	$P2_1$
a, Å	28.4795(19)	9.2788(1)	8.6077(7)	14.0467(7)	8.0381(4)
b, Å	6.9384(4)	16.5810(2)	19.5518(16)	7.3094(2)	16.1648(8)
c, Å	16.3426(11)	12.9229(2)	10.8934(6)	22.4578(10)	15.2996(9)
β, °		101.239(1)		106.528(4)	103.466(2)
V [Å^3]	3229.3(4)	1950.08(4)	1833.3(2)	2210.53(17)	1933.29(18)
Z	8	4	4	4	2
λ [Å]	0.71073	1.54186	0.71073	1.54178	0.71073
ρ_{calcd}, g cm^{-3}	1.955	2.106	2.116	1.957	1.698
cryst size, mm^3	0.10 × 0.08 × 0.02	0.16 × 0.06 × 0.05	0.10 × 0.09 × 0.02	0.08 × 0.01 × 0.01	0.04 × 0.03 × 0.01
T [K]	120(2)	100(2)	100(2)	100(2)	100(2)
μ, mm^{-1}	3.235	33.844	4.530	29.928	1.942
R_1^a	0.0400	0.0201	0.0424	0.0878	0.0543
wR_2^b	0.0687	0.0795	0.1274	0.2414	0.1438
GOF^c	1.054	0.973	1.058	1.127	1.039
CCDC no.	2259330	2259333	2259332	2259338	2259331

^{a} $R_1 = \Sigma||F_o| - |F_c||/\Sigma|F_o|$. ^{b} $wR_2 = \{\Sigma[w(F_o^2 - F_c^2)^2]/\Sigma[w(F_o^2)^2]\}^{1/2}$. ^{c} GOF = $\{\Sigma[w(F_o^2 - F_c^2)^2]/(n - p)\}^{1/2}$, where n is the number of reflections and p is the total number of parameters refined.

Table S2. Crystal Data and Details of Data Collection for $[Co^{II}(H_2L^{SMe})]Cl\cdot CH_3OH$ **(7·CH₃OH)**, $[Co^{III}(HL^{SMe})I]\cdot 3CH_3OH$ **(2'·3CH₃OH)**, $[Co^{III}(L^{SMe,O})I]_2\cdot C_2H_5OH$ **(4·C₂H₅OH)**.

Compound	**7·CH₃OH**	**2'·3CH₃OH**	**4·C₂H₅OH**
empirical formula	$C_{10}H_{21}ClCoN_6OS_2$	$C_{12}H_{28}CoIN_6O_3S_2$	$C_{20}H_{34}Co_2I_2N_{12}O_3S_4$
fw	399.83	554.35	990.49
space group	$P2_1/n$	$P2_1/c$	*Pbca*
a, Å	7.2135(3)	12.3912(2)	15.5100(9)
b, Å	15.8717(5)	22.9114(8)	16.5210(10)
c, Å	14.9785(6)	7.25740(10)	26.1376(16)
β, °	98.4129(12)	92.671(2)	
V [Å^3]	1696.45(11)	2058.14(8)	6697.5(7)
Z	4	4	8
λ [Å]	0.71073	0.56083	1.54178
ρ_{calcd}, g cm^{-3}	1.565	1.789	1.965
cryst size, mm^3	0.51 × 0.05 × 0.03	0.43 × 0.18 × 0.06	0.13 × 0.06 × 0.01
T [K]	273(2)	100(2)	100(2)
μ, mm^{-1}	1.422	1.346	24.951
R_1^a	0.0347	0.0521	0.0437
wR_2^b	0.0955	0.1562	0.1194
GOF^c	1.092	1.095	1.029
CCDC no.	2259340	2259341	2259334

^{a} $R_1 = \Sigma||F_o| - |F_c||/\Sigma|F_o|$. ^{b} $wR_2 = \{\Sigma[w(F_o^2 - F_c^2)^2]/\Sigma[w(F_o^2)^2]\}^{1/2}$. ^{c} GOF $= \{\Sigma[w(F_o^2 - F_c^2)^2]/(n - p)\}^{1/2}$, where n is the number of reflections and p is the total number of parameters refined.

Table S3. Crystal Data and Details of Data Collection for $[Co^{II}(H_4L^{SMe,red})I]I\cdot MeCN$ **(12·MeCN)**, $[Co^{III}(L^{SMe\bullet\bullet})I]$ **(13)** and $[Co^{III}(L^{SMe,O})I(CH_3OH)]$ **(5)**.

Compound	**12·MeCN**	**13**	**5**
empirical formula	$C_{11}H_{25}CoI_2N_7S_2$	$C_9H_{18}CoIN_6S_2$	$C_{10}H_{18}CoIN_6O_2S_2$
fw	632.23	460.24	504.25
space group	*P*-1	$P2_1/c$	*P*-1
a, Å	9.9236(4)	7.6969(2)	7.9809(10)
b, Å	10.5945(4)	19.6721(4)	10.3743(15)
c, Å	11.4418(5)	10.9146(2)	11.788(2)
α, °	62.637(3)		115.212(13)
β, °	75.464(4)	107.083(2)	100.262(12)
γ, °	79.855(3)		101.198(11)
V [Å^3]	1031.56(8)	1579.71(6)	827.9(2)
Z	2	4	2
λ [Å]	0.71073	0.71073	0.71073
ρ_{calcd}, g cm^{-3}	2.035	1.935	2.023
cryst size, mm^3	0.22 × 0.14 × 0.09	0.20 × 0.14 × 0.03	0.15 × 0.10 × 0.03
T [K]	100(2)	100(2)	100(2)
μ, mm^{-1}	4.035	3.301	3.168
$R_1{}^a$	0.0234	0.0599	0.0856
$wR_2{}^b$	0.0609	0.2047	0.3235
GOF^c	1.020	1.084	1.028
CCDC no.	2312261	2259337	2259335

^{a} $R_1 = \Sigma||F_o| - |F_c||/\Sigma|F_o|$. ^{b} $wR_2 = \{\Sigma[w(F_o^2 - F_c^2)^2]/\Sigma[w(F_o^2)^2]\}^{1/2}$. ^{c} GOF $= \{\Sigma[w(F_o^2 - F_c^2)^2]/(n - p)\}^{1/2}$, where n is the number of reflections and p is the total number of parameters refined.

Table S4. Selected experimental (X-ray diffraction data) and calculated (B3LYP/def2-TZVP) metric parameters for complexes **1–3**, **8** and **4.**

**Compound**	[**1**]	^{2}[**1**]^{1}		[**1**]	^{2}[**1**]^{1}
**Bond distances (Å)**	Experimental	Calculated	**Bond angles (deg)**	Experimental	Calculated
Co1a-N1a	1.898	1.925	N1a-Co1a-N6a	98.57	101.40
Co1a-N3a	1.848	1.888	N1a-Co1a-N3a	83.38	82.90
Co1a-N4a	1.842	1.888	N3a-Co1a-N4a	93.47	92.80
Co1a-N6a	1.882	1.925	N4a-Co1a-N6a	83.97	82.90
**Compound**	**[2]**	^{u1}**[2]**^{1}		**[2]**	^{u1}**[2]**^{1}
**Bond distances (Å)**	Experimental	Calculated	**Bond angles (deg)**	Experimental	Calculated
Co-N1	1.885	1.917	N1-Co-N6	96.96	99.55
Co-N3	1.846	1.878	N1-Co-N3	83.01	82.60
Co-N4	1.852	1.879	N3-Co-N4	94.22	92.67
Co-N6	1.877	1.917	N4-Co-N6	83.12	82.60
**Compound**	[**3**]	^{1}[**3**]^{0}		[**3**]	^{1}[**3**]^{0}
**Bond distances (Å)**	Experimental	Calculated	**Bond angles (deg)**	Experimental	Calculated
Co-N1	1.899	1.934	N1-Co-N1^{i}	100.3	102.62
Co-N3	1.876	1.908	N1-Co-N3	80.87	80.22
Co-I1	2.579	2.652	N3-Co-N3^{i}	98.00	96.94
Co-I2	2.578	2.652			
**Compound**	[**8**]	^{2}[**8**]$^{+}$		[**8**]	^{2}[**8**]$^{+}$
**Bond distances (Å)**	Experimental	Calculated	**Bond angles (deg)**	Experimental	Calculated
Co1a-N1	1.888	1.937	N1-Co1-N6	97.5	100.36
Co1a-N3	1.889	1.927	N1-Co1-N3	83.5	82.45
Co1a-N4	1.903	1.927	N3-Co1-N4	95.0	93.79
Co1a-N6	1.899	1.937	N4-Co1-N6	83.7	82.45
Co1-Cl1	2.593	2.404			
**Compound**	[**4**]	^{1}[**4**]^{0}		[**4**]	^{1}[**4**]^{0}
**Bond distances (Å)**	Experimental	Calculated	**Bond angles (deg)**	Experimental	Calculated
Co1-N1	1.906	1.920	N1–Co1–N6	101.30	101.07
Co1-N3	1.886	1.924	N1–Co1–N3	82.10	81.41
Co1-N4	1.912	1.925	N3–Co1–N4	95.80	95.70
Co1-N6	1.933	1.969	N4–Co1–N6	81.10	82.01
Co1-N12	2.068	2.070	N7–Co2–N12	101.60	101.07
Co1-I1	2.593	2.644	N7–Co2–N9	81.80	81.42
Co2-N7	1.895	1.921	N9–Co2–N10	96.30	95.70
Co2-N9	1.888	1.924	N10–Co2–N12	80.60	82.01
Co2-N10	1.897	1.925			
Co2-N12	1.944	1.969			
Co2-N6	2.076	2.070			
Co2-I2	2.597	2.643			

Section 3: X-ray Absorption Spectroscopy

Sample preparation

The samples were prepared by grinding 17–23 mg of the crystalline solid compounds with 83–77 mg of boron nitride and packing the powder into 1-mm thick aluminum cells sealed with 38-μm thick Kapton tape windows.

Experimental details

Co K-edge XAS data were collected at the PETRA III syncrotron (6 GeV, 100 mA, 40 bunches, top-up mode) at the P64 beam line using a liquid nitrogen cooled Si(311) double crystal monochromator calibrated versus the first inflection point of Co foil at 7709.0 eV. The beam spot of 0.25 × 0.25 mm was defined by slits and the estimated flux was 2×10^{10} photons/s. The sample environment between ionization chambers I_0 and I_1 was a He closed cycle cryostat operating at 4.8–5.5 K during the data collection. A silicon-drift detector was placed at 90° with respect to the beam path and the sample rotated 45° in its direction. Both partial fluorescence yield and transmission data were collected with simultaneous collection of Co foil placed downstream between I_1 and I_2 for energy calibration. The gas composition of the ionization chambers (4 cm path) were 100% N_2 at 760 torr and the amplifier gains were set to keep the signal in the linear regime. In order to assess the maximum exposure time for each spot on the surface of each sample, multiple quick scans (ca. 4.5 min) at the pre K-edge region on a single spot were performed until the spectrum showed modulation in intensities and edge drift as signs of photoreduction. The total time until observation of radiation-induced spectral changes for each sample was taken as the safe dwell time on each spot. The scans were done in step mode with 0.2 eV per point in the K-edge range 7689–7749 eV and coarser sampling before and after this energy range for normalization. Scans on multiple spots were obtained for each sample and averaged in Athena software.[13]

Symmetry arguments for the experimentally observed pre-edge intensity

Centrosymmetric metal sites such as those in **1** and **3** in local D_{4h} symmetry face a situation in which the p orbitals have the opposite parity relative to the d orbitals. This prevents the 4p from mixing with the molecular orbitals with main 3d character, whose vacancies give rise to the pre-edge absorptions. With this, the main intensity mechanism – electric dipole – in the 1s to 3d transition vanishes, leaving only the small, albeit noticeable, contributions of the electric

quadrupole. This picture is consistent with the weaker pre-edge peaks of the four- and six-coordinate compounds (**1** and **3** respectively) relative to the five-coordinate **2**. In its local C_{4v} symmetry, z-polarized transition from the 1s to $3d_{z^2}+4p_z$ and xy-polarized transitions to $3d_{xz,yz}+4p_{x,y}$ set have non-vanishing intensity by the electric dipole operator, which contributes to the markedly more intense pre-edge.

If on one hand the lack of p-d mixing in **3** precludes an intense pre-edge, it helps rationalize the interesting intensity modulations in the rising edge (7721-7730 eV) of the experimental spectrum of **3** (Figure 2 or S10a) as being associated with transitions from Co 1s to "3d-free" 4p molecular orbitals.

These insights from group theory are reflected in the TDDFT calculations, where only in **2** the Co $4p_z$ mixes with the predominantly $3d_{z^2}$ molecular orbital. Despite the apparent low 4p contribution of 2.6%, the p-d mixing is enough to give rise to more intense pre-edge in **2** relative to **1** or **3**. Regarding the rising edge of **3**, the calculated "3d-free" NTOs in that region (numbers 64 and 136 in Figure S10a) bear noticeably high Co 4p character: 23% and 5% respectively, apart from the p contribution of the *trans* iodide ligands.

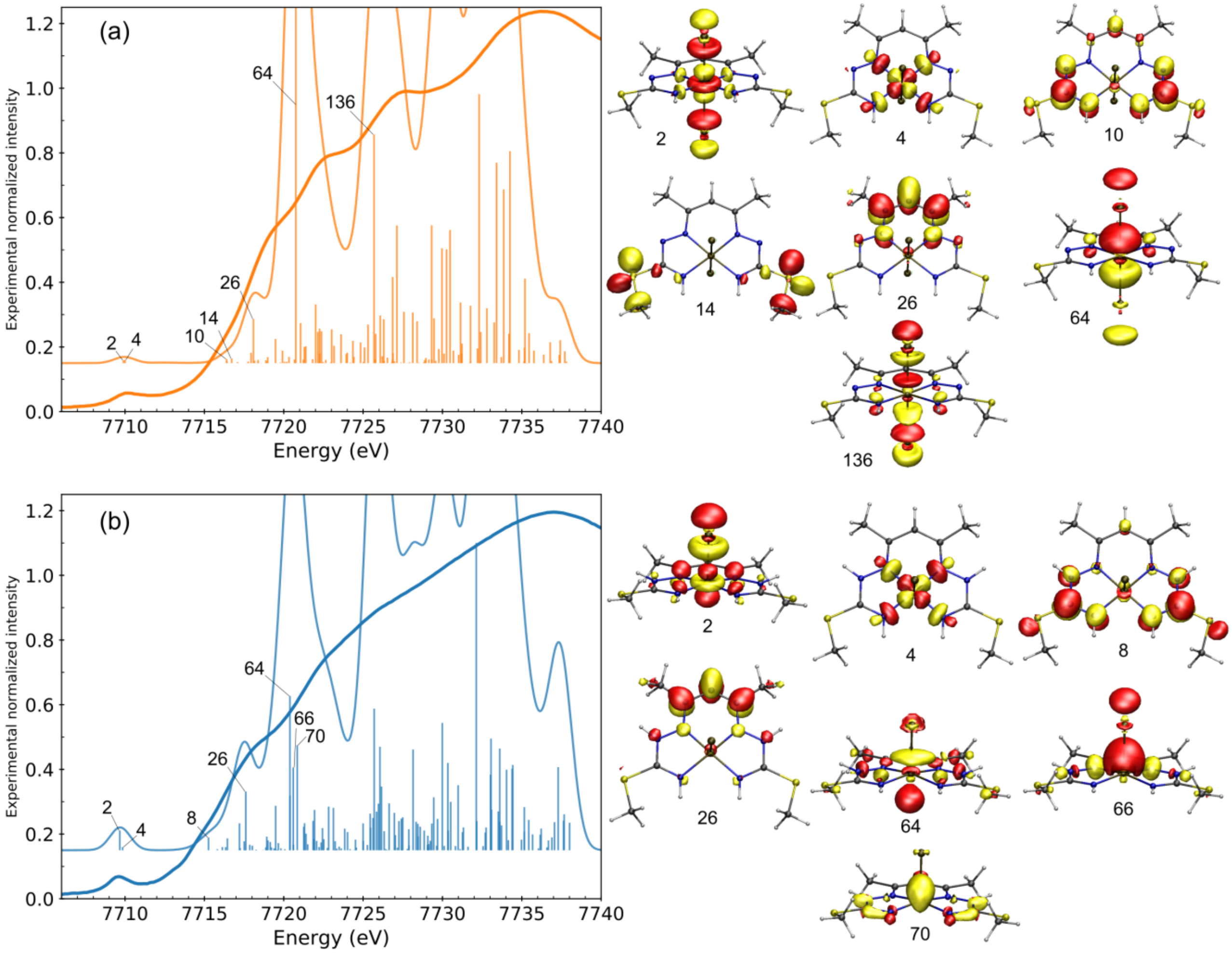

Figure S8. Experimental CoK-edge XAS spectrum and the respective calculated spectrum of (a) $[Co^{III}(L^{SMe})I_2]$ **(3)** and (b) $[Co^{III}(H_2L^{SMe})I]I$ **(2)**. Main excited states calculated by TDDFT/B3LYP are numbered and the respective acceptor natural transition orbitals are shown on the right. The natural transition orbitals (NTOs) are pairwise identical in the unrestricted Kohn-Sham calculations as the species are closed shell. Molecular orientation: *z* axis along Co-I bond, *x* and *y* axes along Co–N bonds.

Section 4: Spectroscopic and Magnetic Characterization of the complexes

UV-vis-NIR Spectroscopy and TD transitions

U**V–vis–NIR**. The electron absorption spectra of solutions of **1**−**3** in methanol are shown in Figure S11, while the absorption maxima together with the molar absorptivity values for **1−3**, **8** and **4** are summarized in Table S5. In general, the identification of the electronic transitions in the UV range

of the spectra of the studied complexes is difficult due to the low symmetry of the species and the various resulting π-π*, n-π* excitations within the conjugated PBIT ligands. The TD-DFT calculations are not of particular aid here, as a severe overlap of these transitions is predicted (Figure S10). An obvious observation is the roughly doubled molar absorptivity for **8** with respect to **1**, and for **4** with respect to **2** (or **3**) below 400 nm, which reflects the 2:1 ratio of complex cation to complex anion in **8** and dinuclear nature of **4**.

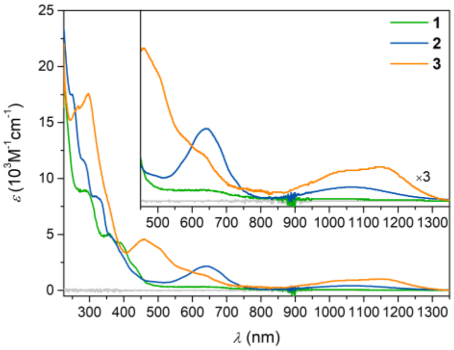

Figure S9. The UV–Vis–NIR spectra of **1**–**3** in degassed MeOH solutions. Inset shows the expansion of the Vis–NIR range.

The low energy bands in the Vis–NIR range are particularly well resolved for the Co^{III} complexes **2** and **3** (Figure S9). Complex **2** shows distinct absorptions at 638 and 1063 nm. The TD-DFT calculations (Figure S10) predict both bands as charge-transfer transitions involving the *d* orbitals of the central metal and the *p* orbitals of the axially coordinated iodido co-ligand. However, the direction of the transfer (MLCT vs LMCT) is difficult to identify because of the mixed character of the orbitals in both ground and excited state. Complex **3** shows absorptions at 457 nm with a shoulder at 620 nm and a NIR absorption around 1100 nm. The TD-DFT prediction identifies the latter as the *d*(Co)-*p*(I) CT transition, involving both iodido co-ligands and the band in the visible range of the spectrum as a mixture of two dominant *d*(Co)-*π*(*L*) excitations. As for complex **2**, the MLCT vs LMCT distinction is elusive due to the mixing of the metal and ligand orbitals in both the ground and excited state.

Table S5. UV-Vis-NIR characteristics of cobalt complexes **1–3**, **8** and **4**.[a]

Complex	λ_{max} [nm] (ε [$M^{-1}cm^{-1}$])
[Co^{II}(H_2L^{SMe})I] **(1)**	222 (21000), 300 (9000), 357 (4950), 578 (450)
[Co^{III}(H_2L^{SMe})I]I **(2)**	250 (17400), 288 (11600), 324 (8400), 367 (4700), 638 (2150), 1063 (450)
[Co^{III}(L^{SMe})I_2] **(3)**	295 (17500), 357sh, 457 (4500), 510sh, 626 (1320), 1030 (1020), 1150 (1120)
[Co^{II}(H_3L^{SMe})Cl]$_2$[$CoCl_4$] **(8)**	235 (59020), 301 (18000), 360 (11100), 392 (7920), 440 (3200)
[Co^{III}($L^{SMe,O}$)I]$_2$ **(4)**	246 (37500), 280sh, 305 (28600), 359 (26400), 528 (3050)

The theoretical TD transitions are in good accord with experiment for species ^{1}[**2**]$^+$, see Figure S10. The reduced species ^{2}[**2**]^0 has no TD transition with a contribution above 600 nm as found in experiment, see Figure S10. In the case of ^{1}[**3**]^0, the UV–vis spectrum in the 1000 nm region is not reproduced well enough, see Figure S10. The considered transitions have a strong d(Co)-p(I) character in ^{1}[**2**]$^+$. In the case of ^{1}[**3**]^0, p orbitals of iodine are involved in the first transition, while the other two transitions have a d(Co)-π(L) character. Nevertheless a straight assignment of the character of the transitions (with respect to Co→L and Co←L charge transfer) would be speculative.

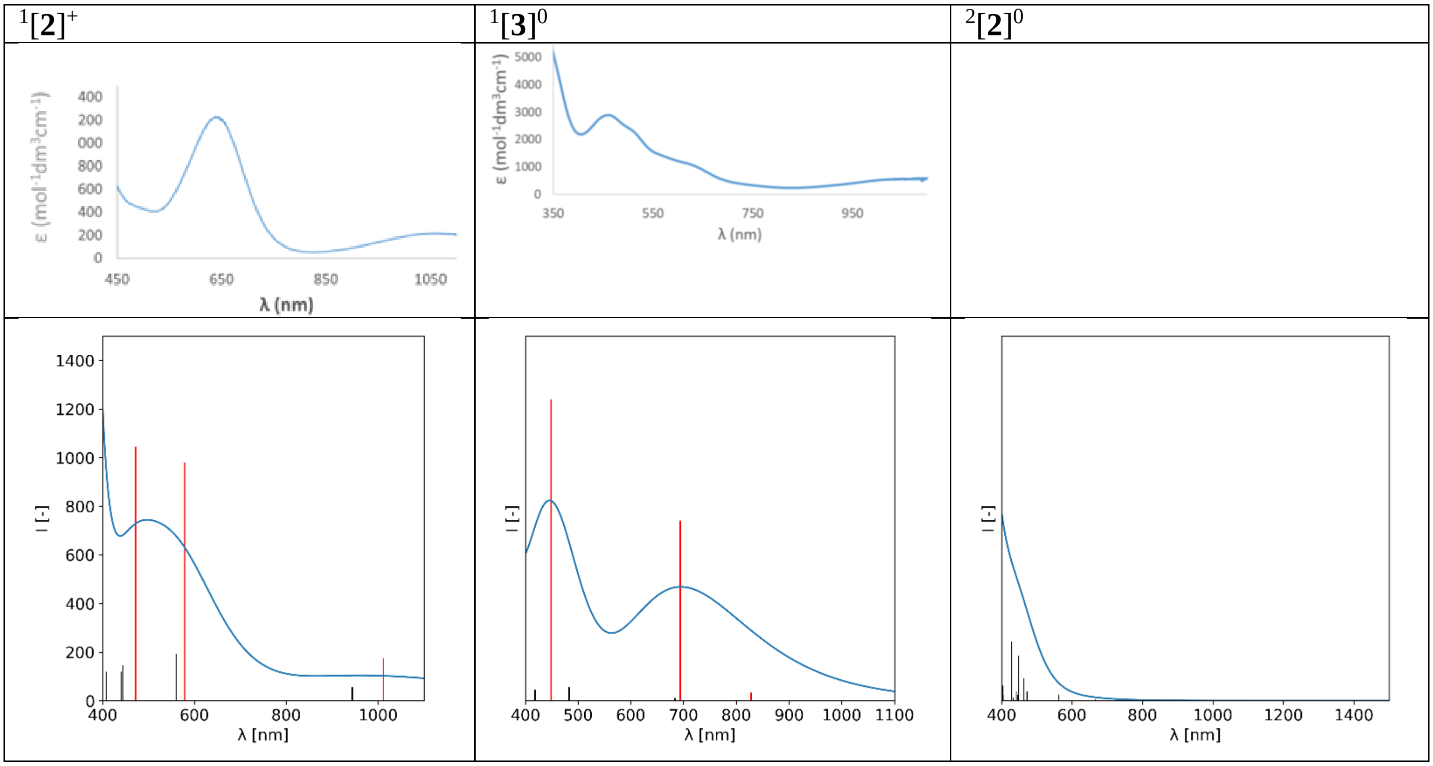

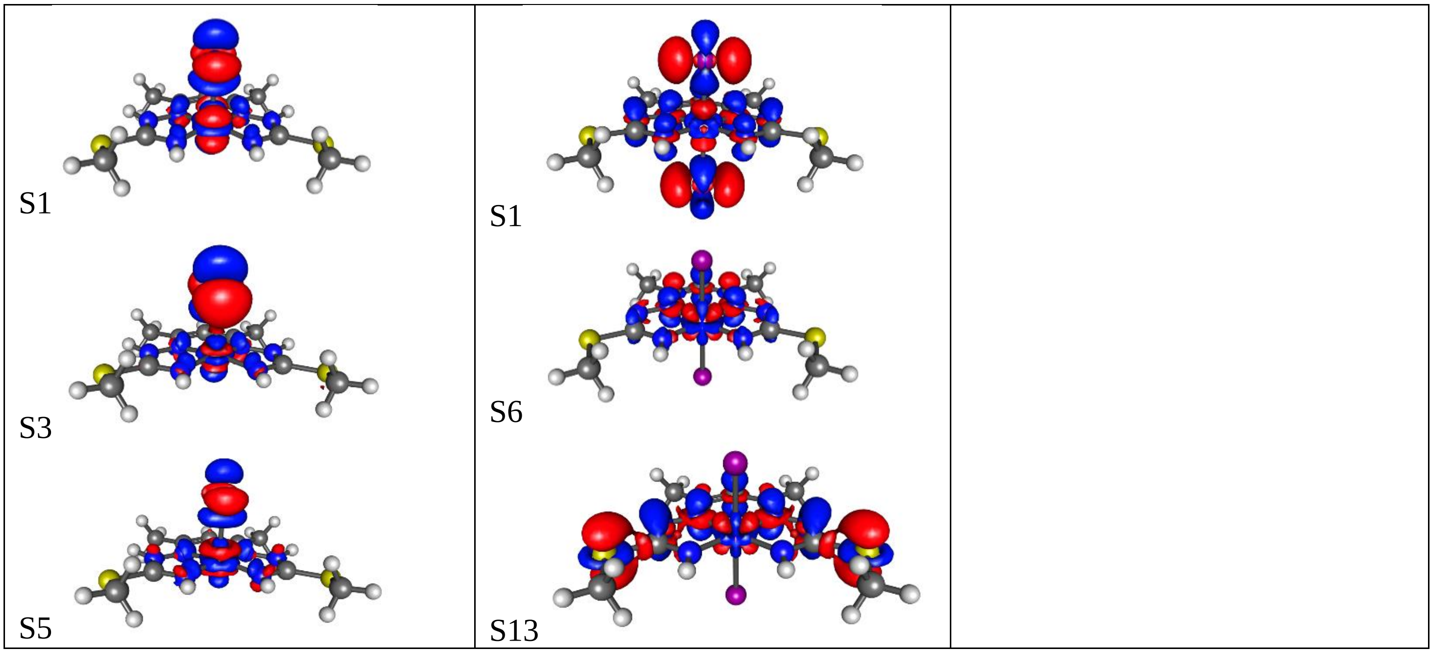

Figure S10. UV–vis spectra of (a) **2** and (b) **3** and B3LYP/ def2-TZVP TD transitions of (c) ^{1}[**2**]$^{+}$, (d) ^{1}[**3**]^{0} and (e) ^{2}[**2**]^{0}, including excited state to ground state difference figures (0.002 au isovalue) for the red bar colored transitions.

NMR spectroscopy of the cobalt complexes

The proton NMR spectra of cobalt(III) complexes **2·CH3OH**, **2'·3CH3OH** and **4·C2H5OH** are in agreement with their X-ray diffraction structures and are shown in Figures S11−S13.

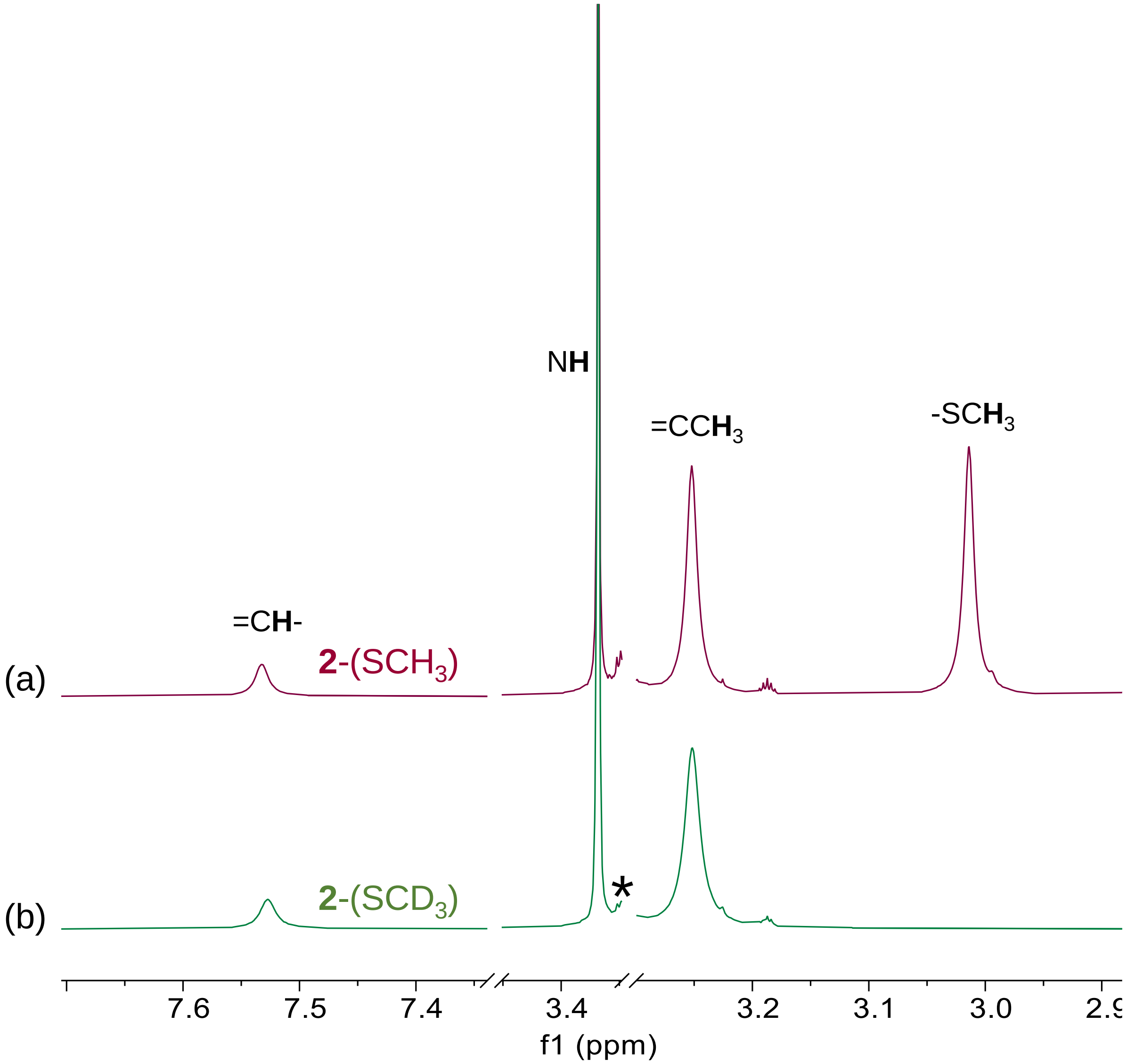

Figure S11. ^{1}H NMR spectrum of **2** in MeOH-*d*4.

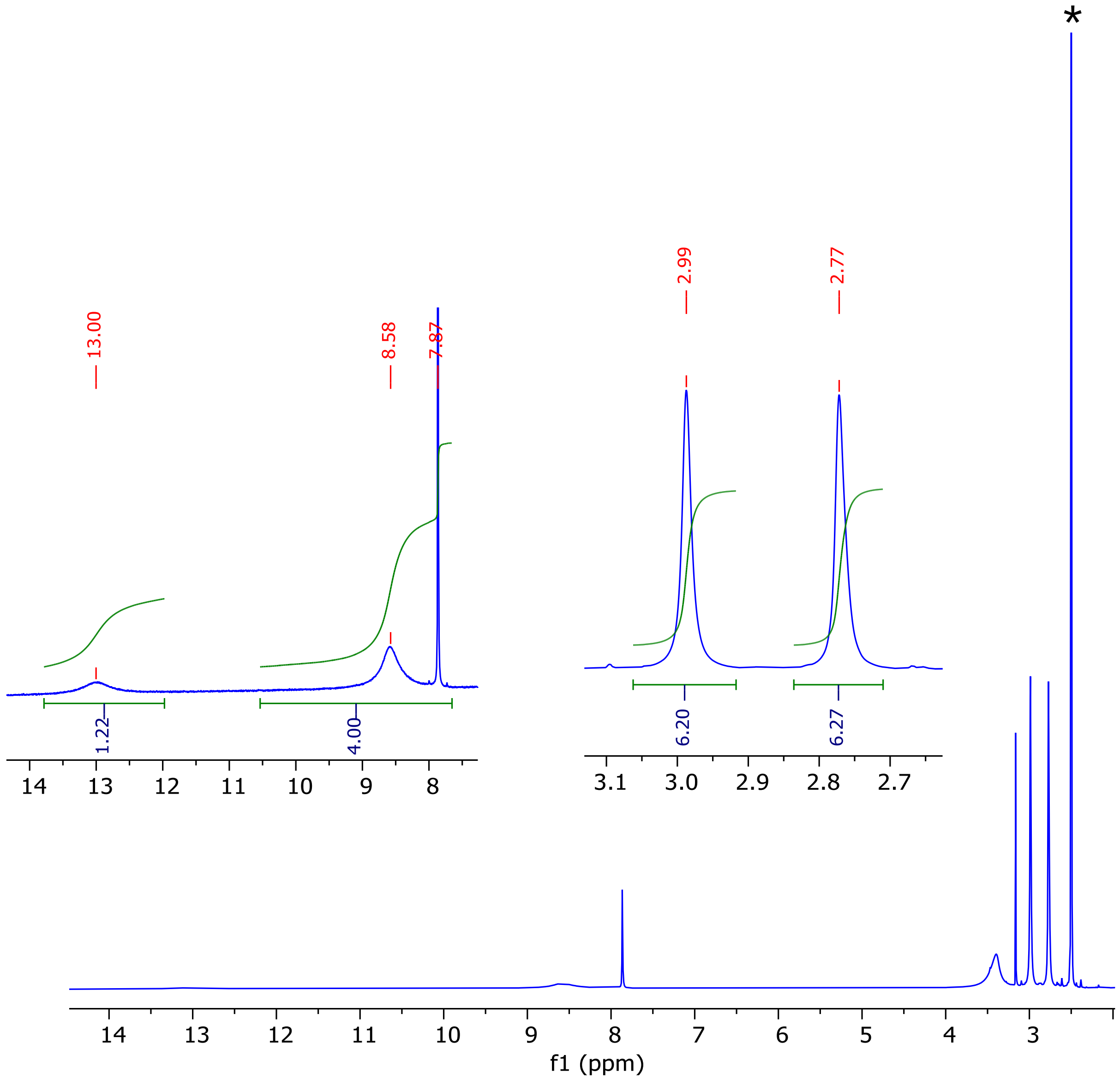

Figure S12. ^{1}H NMR spectrum of [Co^{III}(HL^{SMe})I]·3CH_3OH **(2'·3CH_3OH)** in CD_3CN (*****).

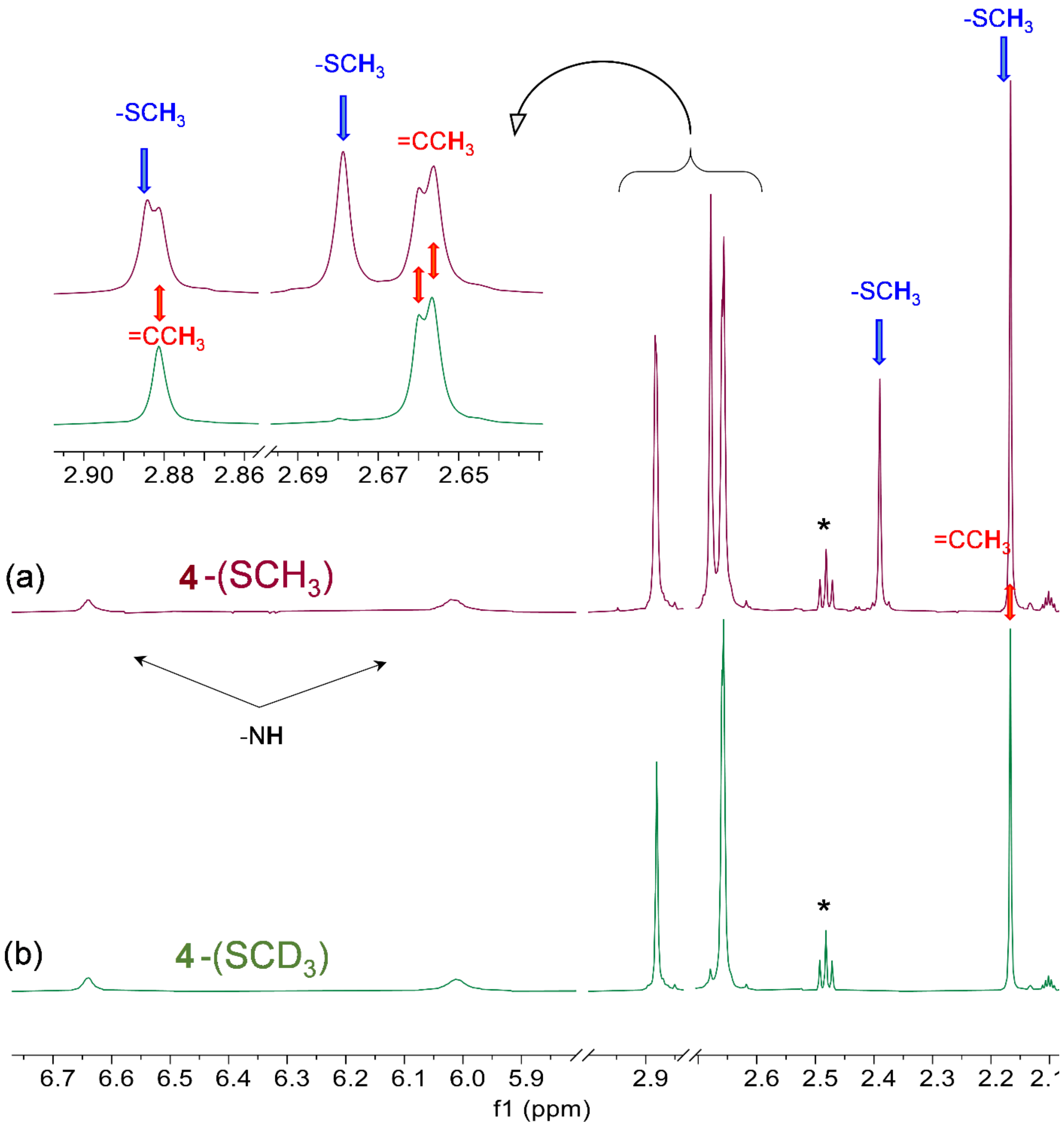

Figure S13. ^{1}H NMR spectrum of **4** in CD_3CN.

Powder diffraction of complex 1

An experimental powder X-ray diffractogram (PXRD) of complex **1** was measured and compared with that simulated from SC-XRD data showing an excellent agreement (Figure S16).

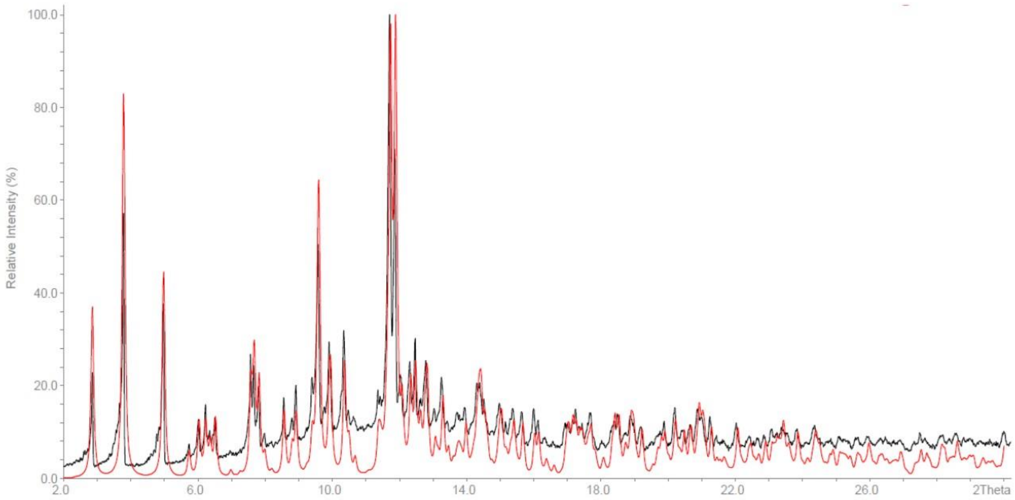

Figure S14. Experimental PXRD of bulk sample **1·0.5CH$_3$OH** (black trace) and simulated one from SC-XRD data (red trace) at 120 K.

Magnetism of complex 1

First, a Heisenberg model for alternating antiferromagnetic (AF) stacks of spin S = 1/2 was used to model the magnetic susceptibility of **1·0.5CH₃OH** (Figure S17a) according to the following Hamiltonian:[14,15]

$$H = -2J \sum_{i=1}^{n/2} \left[\hat{S}_{2i}\hat{S}_{2i-1} + \alpha \hat{S}_{2i}\hat{S}_{2i+1} \right] \qquad (1)$$

$$\chi_m = \frac{Ng^2\mu_B^2}{kT} \cdot \frac{A+Bx+Cx^2}{1+Dx+Ex^2+Fx^3} \qquad (2)$$

$$\chi = (1-\rho)\chi_m + \rho \cdot \frac{Ng^2\mu_B^2}{kT} \cdot S(S+1) + TIP \qquad (3)$$

J is the magnitude of the main interaction between nearest neighbors and αJ, the second interaction found along the stack.

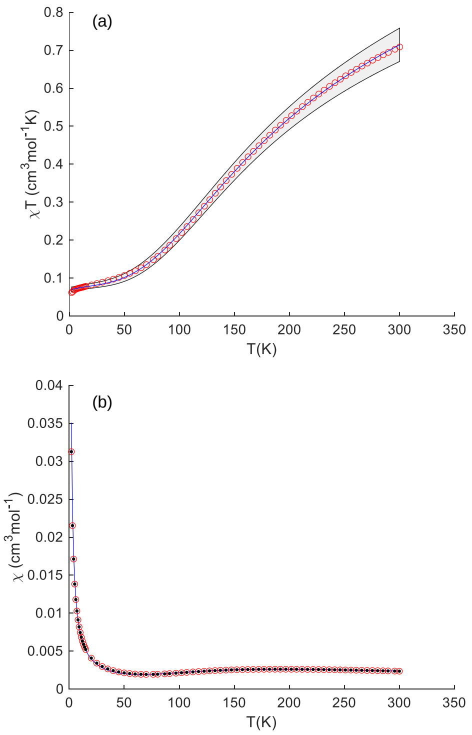

Figure S15. (a) The best fit of χT vs T for **1·0.5CH₃OH** with confident interval according to the Heisenberg model for alternating AF stacks; (b) The best fit of magnetic susceptibility vs T for **1**·0.5CH₃OH according to the Heisenberg model for alternating AF stacks.

The presence of spin-orbital contribution of Co(II) in the low spin configuration (S = ½)[16,17] is taken into account by variation of the g-factor.[18] The best fit of the experimental data (Figures S17a and S17b) yielded J = –119.7(8) cm^{-1} and α = 0.2(1), g = 2.89(8). Contribution of paramagnetic impurity from other than square-planar Co(II) configuration (S = 3/2) is ρ = 1.8% and TIP = 6.1×10^{-4}.

A strong anisotropy of Co(II) justifies the use of the alternation linear Ising model[19,20] for the interpretation of the magnetic data. The results of application of this model are shown in Figure S13.

$$\chi = \frac{Ng^2\mu_B^2}{4kT}\cdot\left[\frac{e^{K_1+K_2}}{\cosh(K_1-K_2)}\right] \qquad (4)$$

where $K_1=J_1/2kT$ and $K_2=J_2/2kT$.

The best fit according eq. 4 (Figure S13) led to the following parameters: J_a = –194(2) cm^{-1}, J_b = 0.0(3) cm^{-1}, g = 3.2(2); ρ = 1.5(2)% and TIP = $4.3(7)\times10^{-4}$, which corresponds at limit of Ising dinuclear cluster.[19]

In the literature for Co(II) square planar:

g_x	g_y	g_z	g
1.91	2.92	2.89	2.57333333
1.798	3.322	3.322	2.814
2	3.26	2.88	2.71333333
1.74	3.805	1.66	2.40166667
1.899	3.28	1.904	2.361

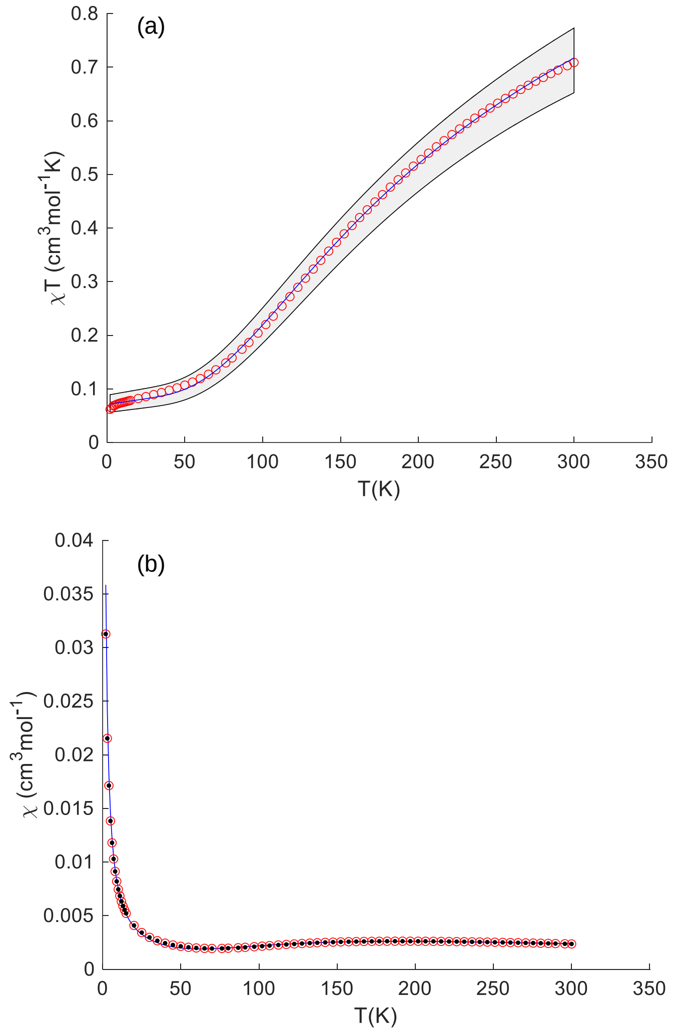

Figure S16. (a) The best fit of χT vs T for **1·0.5CH$_3$OH** with confident interval according to the Ising model for a 1D chain; (b) The best fit of magnetic susceptibility vs T for **1·0.5CH$_3$OH** according to the Ising model for a 1D chain.

EPR spectroscopy of complexes 1 and 8

The low spin cobalt(II) was also identified in the complex cation of 8 by EPR spectroscopy.

Continuous wave (CW) EPR spectra were recorded a Bruker Elexsys E-500 CW X-band spectrometer equipped with an ER 4116DM dual-mode resonator operating in perpendicular mode and a He-flow cryostat (Oxford Instruments ESR900). The microwave power and field modulation were fixed for all experiments at 0.2 mW and 0.5 mT, respectively. The g factor scale was calibrated with a DPPH standard (g = 2.0036)

Figure S19a shows the X-band EPR spectra of **8** and **1** in the solid state at 20 K, while Figure S19b displays the spectrum of **8** in frozen methanol glass at 20 K. The spectrum of **8** in the solid state is a broad line with ΔB_{pp} ~ 200 mT resulting from the ferromagnetic exchange interaction between the low-spin paramagnetic cations (S = 1/2) $[Co^{II}(H_3L^{SMe})Cl]^+$ and the high-spin paramagnetic anions (S = 3/2) $[Co^{II}Cl_4]^{2-}$. The powder spectrum of **1** showed only a weak signal of a high spin (S = 3/2) Co^{II} that was simulated with the Spin-Hamiltonian parameters for an effective S = ½ system given in the legend of Figure S19a. The intensity of this signal is compatible with the unidentified minor impurity found by magnetic measurements of the same sample. The major part of the powdered sample **1** is EPR silent because of the antiferromagnetic coupling in the cofacial cation dimers discussed above. The magnetic dilution in the frozen solution EPR spectrum of **8** resolves the signals of two paramagnetic ions. The characteristics of the high-spin component are compatible with the established parameters of the $[Co^{II}Cl_4]^{2-}$.[21,22] The low-spin component is assigned to the $[Co^{II}(H_3L^{SMe})Cl]^+$ cation and displays a well-resolved hyperfine structure due to the interaction of the unpaired electron spin with the central ^{59}Co nucleus (I = 7/2). The hyperfine splitting was included in simulation and the resulting Spin-Hamiltonian parameters are quoted in the caption of Figure S19b as well. Surprisingly, we were unable to record an EPR signal of $^{2}\mathbf{1}^{+}$ in the frozen methanol glass, down to 20 K, and we can only speculate that some form of the complex stacking prevails also in the solution.

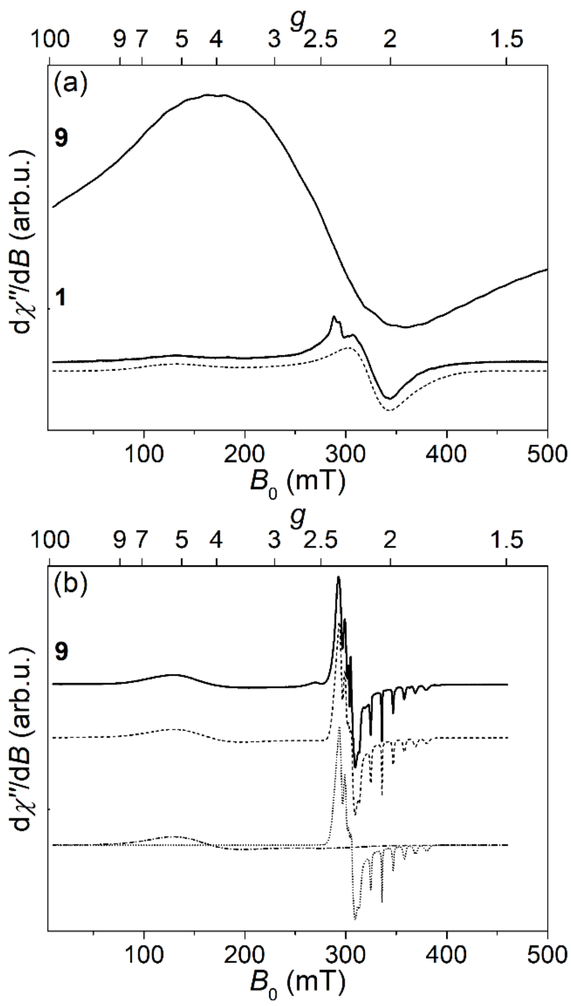

Figure S17. (a) EPR spectra of powdered samples **8** and **1** at 20 K (solid lines) and the simulation of the high spin S = 3/2 signal in **1** using parameters of an effective S = ½ Spin Hamiltonian g'_{eff} values of g'_1 = 1.99, g'_2 = 2.10 , g'_3 = 5.38 (dashed line). (b) EPR spectrum of **8** frozen solution in MeOH at 20 K (solid line) and the composite simulation (dashed line). The simulation of the high spin component, $[CoCl_4]^{2-}$, with g'_{eff} values g'_1 = 2.400, g'_2 = 4.690 , g'_3 = 4.694 (dash dot), and the low spin component, ^{2}**8**$^+$, with g_1 = 2.016, g_2 = 2.255 , g_3 = 2.295, and hyperfine coupling constants A_1 = 314 MHz, A_2 = 12 MHz, A_3 = 74 MHz for the central ^{59}Co (dot), are separated in the bottom trace.

Section 5: Electrochemistry and DFT calculations

Electrochemical investigation of cobalt (II/III) complexes with all three PBIT-ligand forms $(H_2L^{SMe})^-$ in **1** and **2**, $(L^{SMe})^-$ in **3**, $(L^{SMe,O})^{2-}$ in **4** and $(H_3L^{SMe})^0$ in **8** (manuscript, Figure 4 and Figure S18) was carried out by cyclic voltammetry. Cyclic voltammetry was performed in a homemade miniature electrochemical cell using glassy carbon (CG) disk working electrode (WE) (from Ionode, Australia), a platinum wire as the counter electrode, and silver wire as pseudoreference electrode. Ferrocene served as the internal potential standard and the potentials were determined vs ferricenium/ferrocene couple. A Heka PG310USB (Lambrecht, Germany) potentiostat with a PotMaster 2.73 software package was used in cyclic voltammetric and spectroelectrochemical studies. *In situ* spectroelectrochemical measurements were performed on a spectrometer Avantes, Model AvaSpec-2048x14-USB2 under an argon atmosphere in a spectroelectrochemical cell kit (AKSTCKIT3) with the Pt-microstructured honeycomb working electrode, purchased from Pine Research Instrumentation. Halogen and deuterium lamps were used as light sources (Avantes, Model AvaLight-DH-S-BAL). The cell was positioned in the CUV-UV Cuvette Holder (Ocean Optics) connected to the diode-array UV–vis–NIR spectrometer by optical fibres. UV–vis–NIR spectra were processed using the AvaSoft 7.7 software package.

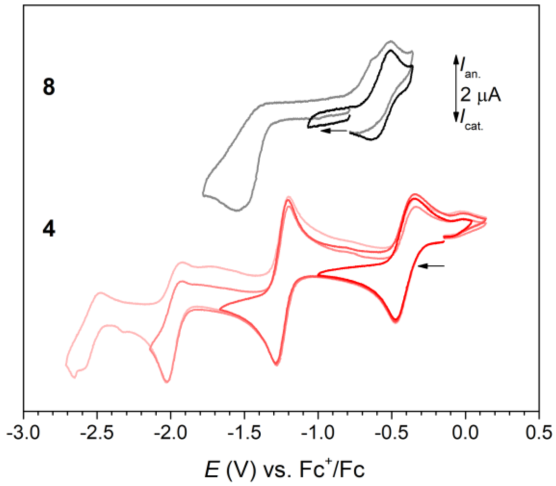

Figure S18. Cyclic voltammograms of ~ 0.5 mM **8** in 0.1 M nBu$_4$NPF$_6$/DMF and ~ 0.5 mM **4** in 0.1 M nBu$_4$NPF$_6$/MeCN, recorded with GC WE at the scan rate of 0.1 Vs^{-1}. Scans with different vertex potentials examining the reversibility of individual redox processes are shown with varying color tone. Horizontal arrows indicate the CV scan direction.

Table S6. Electrochemical data observed for **1**–**3**, **8** and **4** in MeCN on glassy carbon (GC) electrode.

Complex	$E_{1/2}$ (V) [a]	E_{pc} (V) [b]	ΔE_p (mV) [c]
**1**	−0.39		92
		−1.36	
		−1.89	
		−2.56	
**2**	−0.40		99
		−1.37	
		−1.89	
		−2.54	
**3**		−0.45	
		−1.43	
		−2.67	
**8**	−0.57[d]		106[d]
		−1.54[d]	
**4**	−0.41		133
	−1.24		87
	−1.98		100
	−2.54		125

[a] $E_{1/2} = (E_{pc} + E_{pa})/2$, where E_{pc} and E_{pa} correspond to the cathodic and anodic peak potentials of an electrochemically reversible process, in V vs Fc^+/Fc, respectively. [b] E_{pc}, cathodic peak potential of an electrochemically irreversible process. [c] $\Delta E_p = E_{pa} - E_{pc}$. [d] redox potentials in DMF

The CV of **8** in DMF reveals a reversible oxidation at $E_{1/2}$ = –0.57 V and an irreversible reduction at E_{pc} of –1.54 V. In addition, in this case, the increased peak currents reflect the double concentration of the mononuclear $[Co^{II}(H_3L^{SMe})Cl]^+$ species. Note that the negative shift of the redox potentials compared to complex **1** involves also the impact of different redox potentials of the reference Fc^+/Fc couple in the applied solvents (0.40 V vs. saturated calomel electrode (SCE) in MeCN and 0.45 V vs SCE in DMF).[23] Complex **4** shows two reversible reductions at $E_{1/2}$ = –0.41 V and –1.24 V, followed by two further quasi-reversible reductions at $E_{1/2}$ = –1.98 V and –2.54 V, respectively. The peak currents of the reversible redox couples are roughly doubled compared to complexes **1** or **2**, thus representing either two-electron events, or single-electron reductions of mononuclear $[Co^{III}(L^{SMe,O})I]$ units formed by the dissociation of binuclear **4** in the solution.

Following the similarity in redox potentials with complexes **1** and **2** (Table S6), we assign the reversible oxidation of **8** at –0.57 V (in DMF) and the reversible reduction of **4** at –0.41 V to single-electron transfers within Co^{III}/Co^{II} couples (Table S6). The reduced form of **4** induces the yellowish orange tone of the MeCN solution due to the absorption at 477 and 371 nm, respectively (Figure S19).

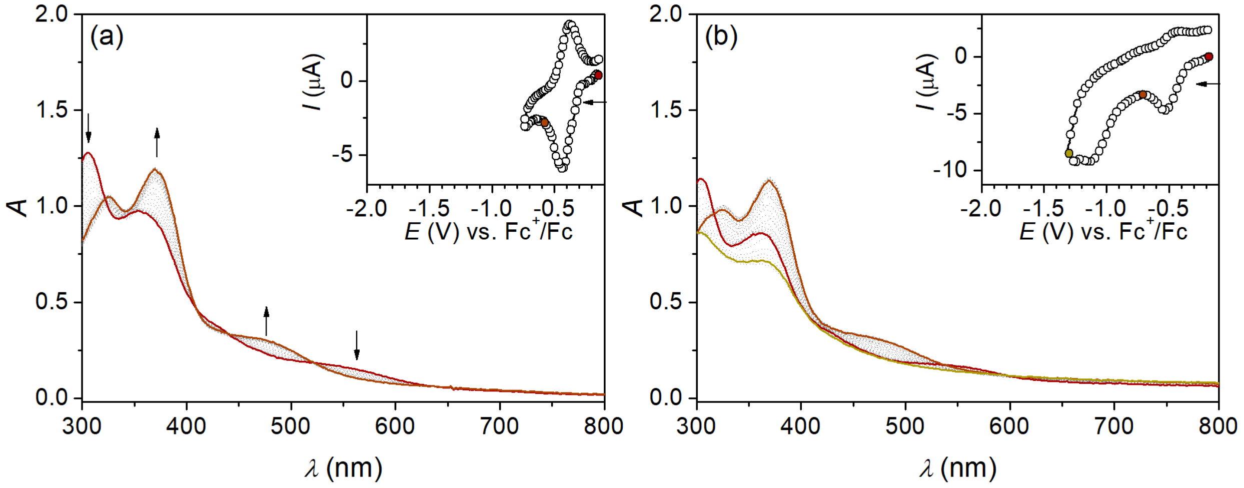

Figure S19. The spectra obtained during (a) 1st and (b) 1st and 2nd reduction of **4** in 0.1 M *n*Bu_4NPF_6/MeCN. Insets show the corresponding CVs measured with Pt working electrode at the scan rate of 3 mVs^{-1}. Circles mark potentials where the spectra were sampled, and for highlighted records, the color matches the spectrum line color.

The first reduction of **1** and the second reduction of **2** occur at virtually identical E_{pc} of –1.37 V, while the second reduction of complex **4** at –1.24 V is an electrochemically reversible event at a scan rate of 0.1 Vs^{-1} (Figure S19). However, at a much slower rate of 3 mVs^{-1} used in the spectroelectrochemical investigations (Figure S19), the cathodic reoxidation peak is absent, revealing a chemical follow-up reduction of the formal Co^{I} complex. Also, in this case, the bleaching of the solution color would be compatible with the release of the PBIT ligand, with absorptions only in the UV range. The locus of this redox event (the first reduction of **1** and the second reduction of **2** and **4,** which may lead to formal Co^{I} species) and the identity of the electron-withdrawing moiety have been assessed by DFT calculations (vide infra). The lowest vacant orbital in the parent ^{2}[**1**]$^{+}$ as well as the HOMO of the thermodynamically most stable reduced form ^{1}[**1**]^{0} (Figure S20) are predicted with a strong metal character.

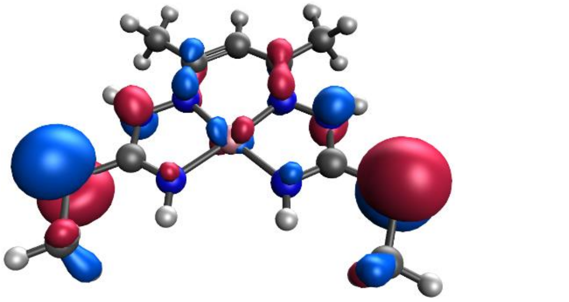

(a) MO83a α-HOMO-3 –0.370659 (–0.339948)

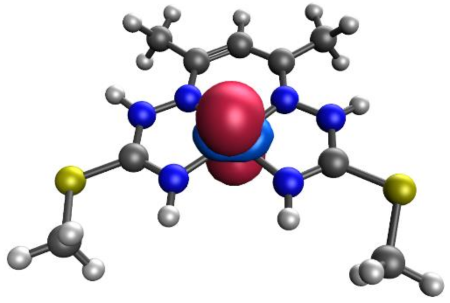

(b) MO84a α-HOMO-2 –0.361703 (–0.324819)

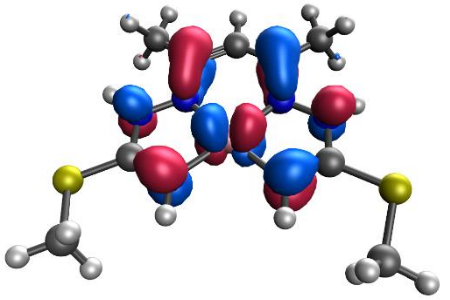

(c) MO85a α-HOMO-1 –0.341471 (–0.315672)

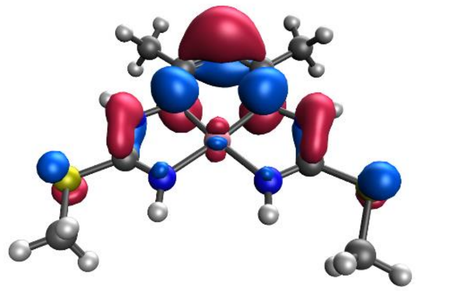

(d) MO86a α-HOMO –0.311467 (–0.194082)

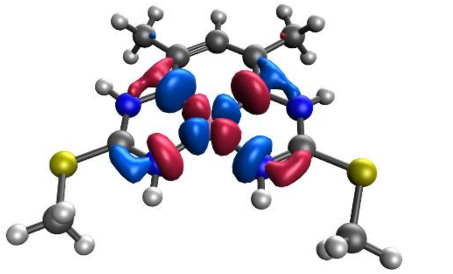

(e) MO87a α-LUMO –0.164482 (–0.157165)

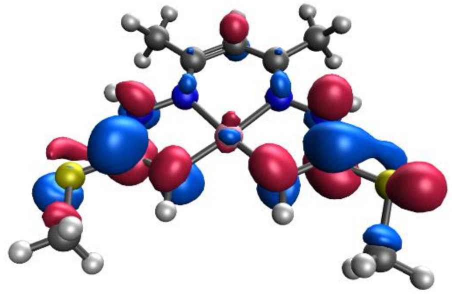

(f) MO88a α-LUMO+1 –0.129872 (–0.121014)

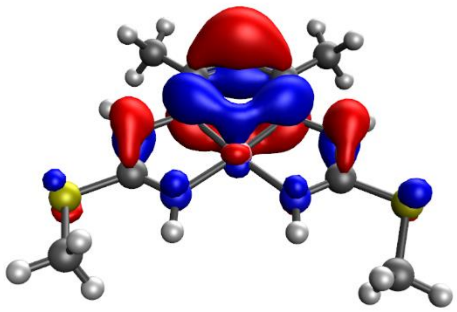

(g) MO85b β-HOMO (–0.315670)

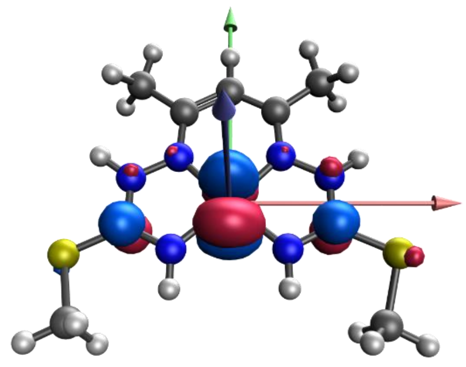

(h) MO86b β-LUMO (–0.194082)

Figure S20. Frontier orbitals of ^{2}[**1**]$^{+}$, α (β) eigenvalues in hartrees, isosurface value is 0.04 e.bohr^{-3}.

The electron transfer (reduction of $^{2}[\mathbf{1}]^{+}$) should thus represent the Co^{II}/Co^{I} reduction leading to the singlet spin state of $^{1}[\mathbf{1}]^{0}$, formally described as $[Co^{I}(H_2L^{SMe})]^{0}$. However, the CV records show an electrochemically irreversible event that indicates a chemical conversion of the putative Co^{I} complex in the MeCN solution. The optical spectra of the follow-up product(s) show the dominant absorption at 383 nm (Figure 5b). In the absence of detailed product analysis, we can only speculate on the release of the PBIT ligand, similar as previously observed upon the reduction of copper(II) isothiosemicarbazone complexes.[24]

The irreversible or quasi-reversible reductions occurring in the entire complex series at potentials more negative than –1.5 V could correspond to the reductions of the PBIT ligand. This hypothesis is also compatible with the CV response of the $[Zn^{II}(H_3L^{Et})Cl]Cl$ (Figure S21), where two irreversible ligand-centered reductions were found at –1.9 V and –2.5 V.

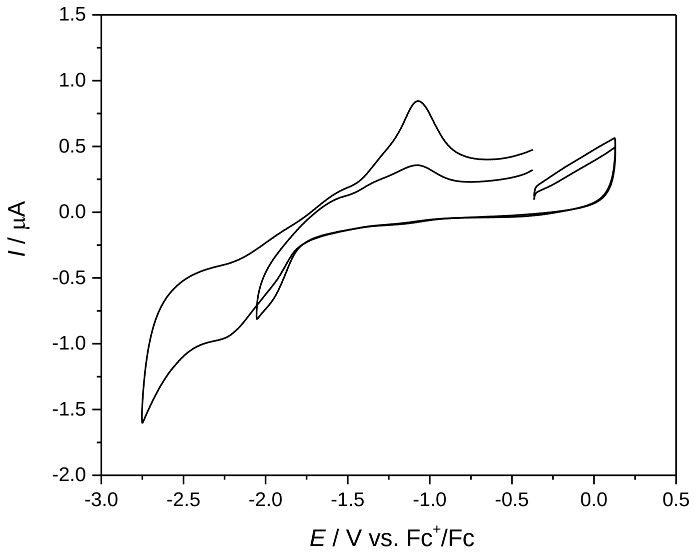

Figure S21. Cyclic voltammograms of $[Zn^{II}(H_3L^{SEt})Cl]Cl$ in 0.1 nBu$_4$NPF$_6$/MeCN.

Section 6: Computational Studies

In this section the electronic structure (oxidation state of Co) of **1–3** is first elucidated in detail. Metrical charge of the ligand, localized orbitals, Mulliken d(Co) atomic orbital (AO) populations and spin (unpaired electron) density are considered to support the XAS oxidation state assignment and prevent any ambiguity of experimental results of initial species **1–3** discussed in the mansucript. Selected bond lengths and bond angles in several X-ray diffraction structures are collected in Table S4. The metrical charge of the ligands[25,26] are shown in Table S7. B3LYP spin state preference, d(Co)-like localized orbitals, S^2-expectation values and Mulliken charges/spins of Co are summarized in Table S8 (identical HF and BLYP results are compiled in Tables S9 and S10, respectively). Spin densities of chosen systems are shown in Figure 9 (see manuscript). Frontier orbitals of selected spin states of **1–3** are compiled (see Figures S20, S22 and S23).

In vacuo single point calculations and geometry optimizations in various spin states have been performed at the B3LYP[27,28,29,30] level of theory [BLYP[27,28] Hartree-Fock (HF) results are reported in the ESI] using the def2-TZVP basis set[31,32] in Gaussian16 program package. For open shell systems, the unrestricted DFT formalism has been used, i.e. B3LYP implies UB3LYP. The stability of the optimized structures was confirmed by the vibrational analysis (no imaginary vibrations). The vibrational spectra (frequencies and intensities) have been visualized using the MOLDRAW package.[33] Localized orbitals, Mulliken charges, orbital and or spin density populations and cube files have been obtained with the Orca 4.2.0 package.[34] Orbitals and spin density visualization have been performed in the IQmol software upon the obtained cube files. *J*-coupling was evaluated according to the formula:

$$J_{AB} = -\frac{(E_{HS}-E_{BS})}{\langle S^2\rangle_{HS}-\langle S^2\rangle_{BS}} \quad (1)$$

where E_{HS} denotes the unrestricted high spin (triplet) total energy, E_{BS} denotes the broken symmetry (BS) singlet state energy, $\langle S^2\rangle_{HS}$ and $\langle S^2\rangle_{BS}$ are the particular expectation values of spin momentum squared.

Time-dependent density functional theory (TD-DFT) electronic transitions[35,36] in the UV–vis region were computed for chosen geometries as implemented in Gaussian16. The 80 lowest electron excitations from the ground states were accounted for.

Optimized geometries. In order to confirm the initial accuracy of the calculations in modelling the electronic structure and spectroscopic properties of the studied cobalt complexes, the optimized geometries for **1**–**3** and **4** were compared to the experimental structures obtained from SC-XRD analysis, see Table S4. Overall, the DFT calculations with the uB3LYP method show reasonable agreement with the SC-XRD experiment. The Co complexes are predicted to have an almost square-planar geometry with the tetradentate organic ligand H_3L^{SMe} or its anionic forms. The metal–ligand bonds show the largest variance, being consistently overestimated in all complexes up to 0.04 Å for the *r*(N–Co) bonds. All *r*(C–C) and *r*(C–N) bond lengths are very accurately reproduced and found to be within the experimental error for all structures.

K-edge X-ray absorption spectroscopy (XAS) **calculations** were performed with ORCA 4.2.1[37,38] by time-dependent DFT as described previously[39] within Tamm-Dancoff approximation on fully optimized geometries excluding counter ions. The protocol is the same as reported earlier.[40] B3LYP[41,42,30,27,29] functional was employed with RIJCOSX[43] "chain of spheres" approximation to accelerate the Hartree-Fock exchange calculations. Scalar relativistic effects were accounted for by the Douglas-Kroll-Hess approach. The all-electron contracted Aldrich's triple-ζ quality DKH-def2-TZVP(-f) basis set[44] was used for H-Co, old-DKH-TZVP for I and SARC/J was the auxiliary basis set.[45] The oscillator strength was computed as the sum of electric dipole, electric quadrupole and magnetic dipole contributions. The electronic transition were attributed by inspection of the acceptor canonical and natural transition orbitals.[46]

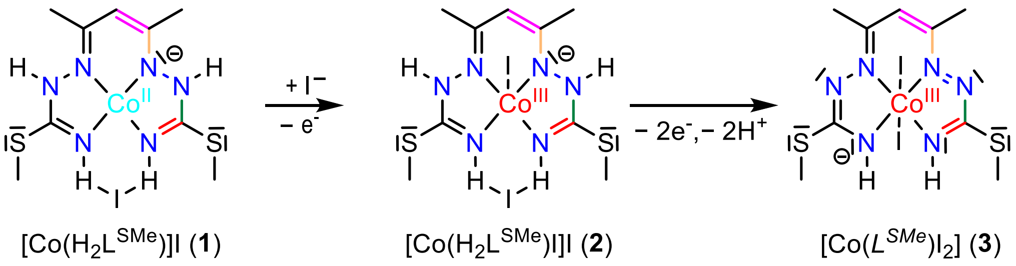

Scheme S3. Considered bonds and the metrical parameters in complexes **1**–**3**. For **1**: d1 – d(N1a–C1a); d2 – d(C1a–N2a); d3 – d(N2a–N3a); d4 – d(N3a–C2a); d5 – d(C2a–C3a); Δ=[(d1+d2+d4) / 3 – d3]. For **2**: d1 – d(N1–C1); d2 – d(C1–N2); d3 – d(N2–N3); d4 – d(N3–C2); d5 – d(C2–C3); Δ=[(d1+d2+d4) / 3 – d3]. For **3**: d1 – d(N1–C1); d2 – d(C1–N2); d3 – d(N2–N3); d4 – d(N3–C2); d5 – d(C2–C3); Δ=[(d1+d3+d5) / 3 – (d2+d4) / 2].

Table S7. Metrical parameters of the considered species.

^{2}[**1**]$^{+}$ and ^{1}[**2**]$^{+}$ $\Delta = [(d1+d2+d4) / 3 - d3]$ [$M^{X}(H_2L)^{q}I_n]^{X+q-n}$								
	q	X	d1	d2	d3	d4	d5	Δ
^{1}[**1**$_{\mathbf{Zn}}$]$^{+}$	**–1**	II	1.306	1.346	1.379	1.329	1.402	**–0.051**
^{2}[**1**$_{\mathbf{Zn}}$]^{0}	–2	II	1.330	1.347	1.355	1.361	1.399	–0.008
^{1}[**1**$_{\mathbf{Zn}}$]$^{-}$	–3	II	1.358	1.367	1.343	1.365	1.400	0.021
^{2}[**1**]$^{+}$	**–1**	II	1.307	1.345	1.388	1.330	1.399	**–0.061**
^{1}[**2**]$^{+}$	**–1**	III	1.301	1.351	1.376	1.332	1.397	**–0.048**
^{1}[**2**]^{0} $\Delta = [(d1+d3+d5) / 3 - (d2+d4) / 2]$ [M^{X} L $I_n]^{X+q-n}$								
	q	X	d1	d2	d3	d4	d5	Δ
^{2}[**3**$_{\mathbf{Zn}}$]$^{2+}$	0	II	1.296	1.421	1.275	1.373	1.404	–0.072
^{1}[**3**$_{\mathbf{Zn}}$]$^{+}$	**–1**	II	1.304	1.383	1.298	1.340	1.406	**–0.026**
^{2}[**3**$_{\mathbf{Zn}}$]^{0}	–2	II	1.319	1.340	1.343	1.329	1.410	0.023
^{1}[**3**]^{0}	**–1**	III	1.297	1.364	1.308	1.342	1.396	**–0.019**

The metrical parameters Δ are calculated from bond lengths that become elongated or shortened upon the change of the total charge of the Zn-containing complexes, see formulas in Scheme S3 and values in Table S7. Subsequently, the metrical charge of the ligand in a given Co complex is obtained and compared to Δ of the optimized Zn-containing complexes. It is found that the metrical charge of the ligand is –1 (see Table S7) for all three species **1−3.**

Table S8. Total B3LYP/def2-TZVP energies, DFT energies (ΔE), expectation values of S^2 and localization orbital analysis, MPA charges and spin on Co for studied complexes.

B3LYP	E [hartree]	ΔE [kJ/mol]	S^2	Co d LOC	Charge/spin
**^{2}[1]$^{+}$**	**–2861.09267**		**0.755**	**4α+3β**	**0.194/1.026**
**^{4}[1]$^{+}$**	-2861.07526	45.7	3.759	5α+2β	0.431/2.696
^{1}[**1**]$^{2+}$	–2860.73624	2.7	0.000	3α*+3β	0.193
**^{3}[1]$^{2+}$**	**–2860.73728**	**0**	**2.036**	**4α+3β**	**0.240/1.149**
^{u1}[**1**]$^{2+}$	–2860.73624	2.7	1.035	4α+3β	0.230/0.946
**^{1}[2]$^{+}$**	**–3158.90741**	**0.3**	**0.000**	**3α+3β**	**–0.029**
^{3}[2]$^{+}$	–3158.89398	35.6	2.024	4α+2β	0.014/1.297
**^{u1}[2]$^{+}$**	**–3158.90752**	**0**	**0.137**	**3α+3β**	**–0.026/0.260**
**^{1}[4]^{0}**	**–6462.47197**	**0**	**0**	**3α+3β/3α+3β**	**0.293**, **0.289**
^{3}[4]^{0}	–6462.44612	67.9	2.030	3α+3β/4α+4β	0.304, 0.474/0.001, 1.566
^{u1}[4]^{0}	–6462.47191	0.2	0	3α+3β/3α+3β	0.293, 0.290
**^{1}[3]^{0}**	**–3455.71414**	**0**	**0**	**3α+3β**	**–0.238**
^{3}[3]^{0}	–3455.68607	73.7	2.021	3α+3β	–0.210/–0.066
^{u1}[3]^{0}	–3455.71414	0.0064	0	3α+3β	–0.238

*a more delocalized orbital is also found with 0.5 e contribution of Co, hence the restricted ansatz points also to a formal 4a+3b localization scheme.

Table S9. Total BLYP/def2-TZVP energies, DFT energies (ΔE), expectation values of S^2

BLYP	E [hartee]	ΔE [kJ/mol]	S^2
^{2}[**1**]$^{+}$	–2860.85332		0.760
^{1}[**1**]$^{2+}$	–2860.50106	24.9	0
^{3}[**1**]$^{2+}$	**–2860.51056**	**0.0**	**2.0096**
^{u1}[**1**]$^{2+}$	–2860.50612	11.5	0.7981
^{1}[2]$^{+}$	**–3158.53822**	**0**	**0**
^{3}[2]$^{+}$	–3158.51950	49.1	2.0134
^{1}[3]^{0}	**–3455.21395**	**0**	**0**
^{3}[3]^{0}	–3455.18702	70.7	2.0053

Table S10. Total HF/def2-TZVP energies, DFT energies (ΔE), expectation values of S^2

HF	E [hartee]	ΔE [kJ/mol]	S^2
^{2}[**1**]$^{+}$	–2853.96371		1.251
^{1}[**2**]$^{2+}$	–2853.48366	465.0	0
^{3}[**2**]$^{2+}$	**–2853.66078**	**0**	**2.9643**
^{u1}[**2**]$^{2+}$	–2853.66074	0.1	1.9921
^{1}[**3**]$^{+}$	–3150.55039	264.6	0
^{3}[**3**]$^{+}$	–3150.58706	168.3	2.0918
^{u1}[**3**]$^{+}$	**–3150.65116**	**0**	**2.3046**

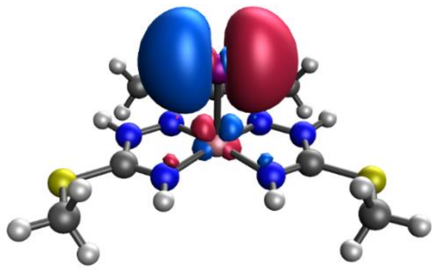

MO96a α-HOMO-2 –0.334170(–0.334598)

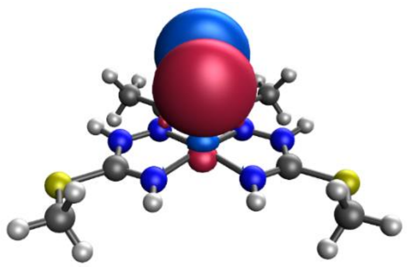

MO97a α-HOMO-1 –0.333203 (–0.334583)

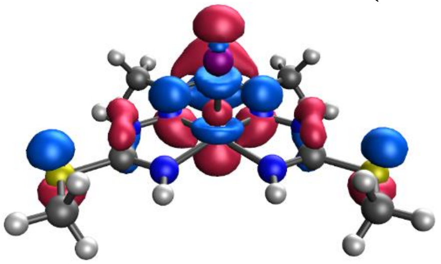

MO 98a α-HOMO –0.329323

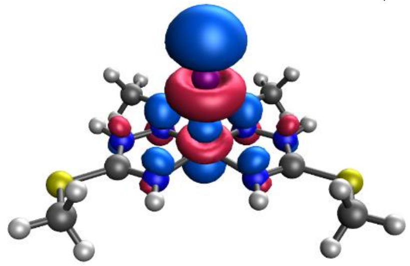

MO 99a α-LUMO –0.234466 (–0.212556)

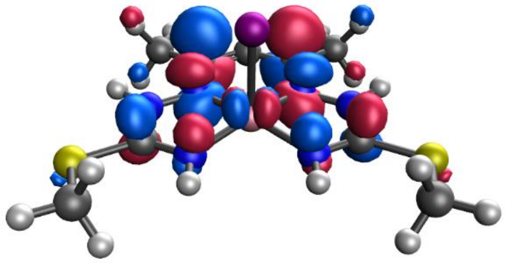

MO 100a α-LUMO+1 –0.172166 (–0.172397)

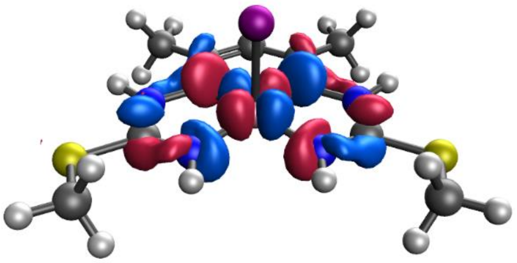

MO101a α-LUMO+2 –0.162737 (–0.156787)

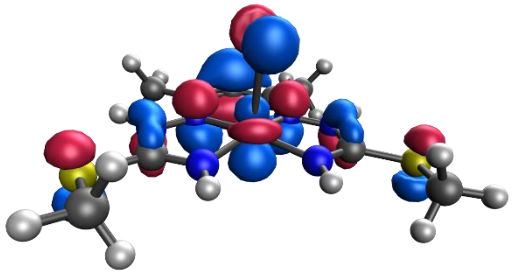

MO98b β-HOMO (–0.326882)

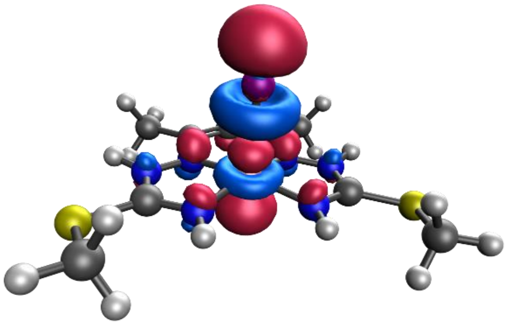

MO 99b β-LUMO (–0.212556)

Figure S22. Frontier orbitals of ^{u1}[**2**]$^{+}$ (eigenvalues are shown in parenthesis; in hartrees), isosurface value is 0.04 e.bohr^{-3}.

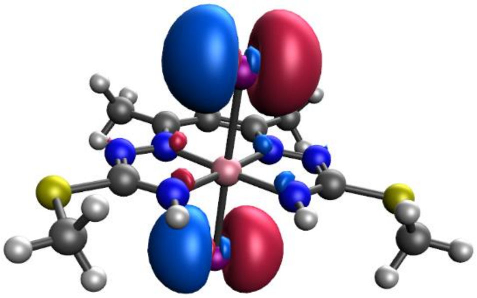

MO107 HOMO-3 (–0.23105)

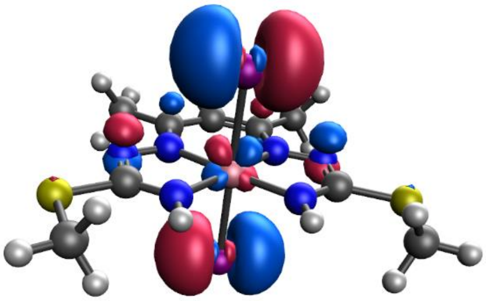

MO108 HOMO-2 (–0.22559)

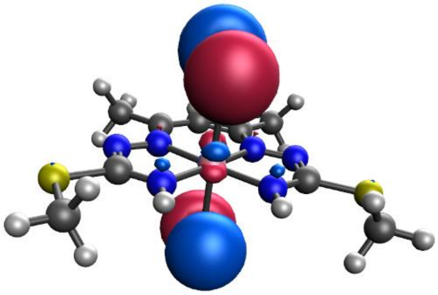

MO109 HOMO-1 (–0.22432)

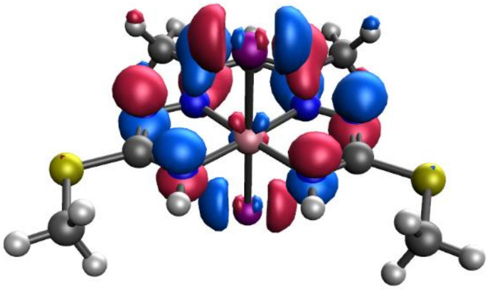

MO110 HOMO (–0.21531)

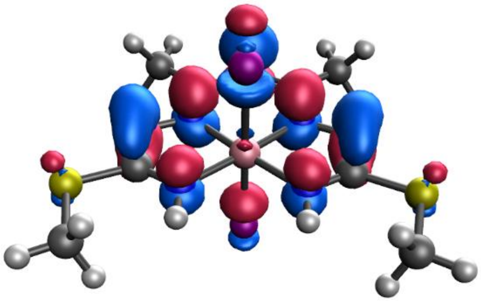

MO111 LUMO (–0.13835)

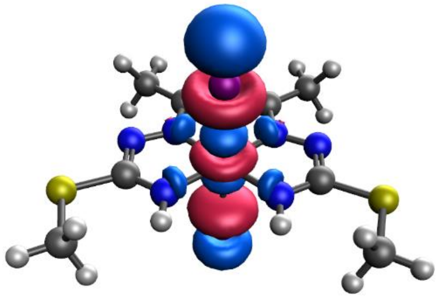

MO112 LUMO+1 (–0.10315)

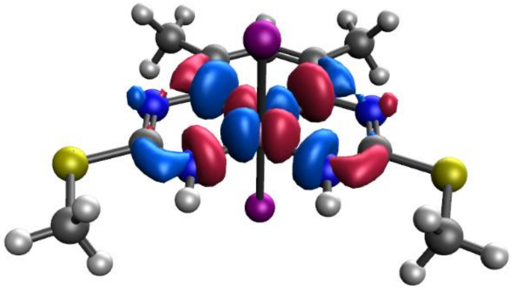

MO113 LUMO+2 (–0.05638)

Figure S23. Frontier orbitals of ^{1}[**3**]^{0} (eigenvalues are shown in parenthesis; in hartrees), isosurface value is 0.04 e.bohr^{-3}.

Section 7: Catalytic Studies

Starting material synthesis

9q

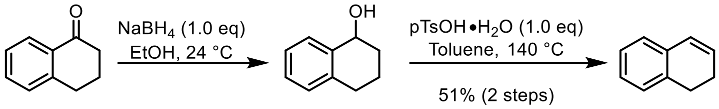

1-Tetralone (439 mg, 3.0 mmol, 1.0 eq) and ethanol (15 mL) were charged into a 25 mL round-bottomed flask. The solution was cooled to 0 °C with an ice bath and sodium borohydride (226 mg, 6.0 mmol, 2.0 eq) was added in two portions over 1 h. The mixture was stirred at 24 °C for 4 h. Then, the mixture was diluted with water (20 mL) and a 1 M HCl was added until no further gas evolution was observed. The mixture was diluted with EtOAc (20 mL), and the phases were separated. The aqueous phase was extracted with EtOAc (2 × 20 mL), the organic layers were combined, dried over Na_2SO_4 and concentrated under reduced pressure. To the crude mixture was added *p*-toluenesulfonic acid monohydrate (571 mg, 3 mmol, 1.0 eq), toluene (10 mL) and the mixture was heated at 140 °C for 5 h. Afterwards, the mixture was allowed to cool to 23 °C, diluted with EtOAc (20 mL) and washed with a sat. aq. solution of $NaHCO_3$ (2 × 20 mL) followed by a sat. aq. solution of NaCl (20 mL). The organic layer was dried over Na_2SO_4 and concentrated under reduced pressure. The crude mixture was purified by flash chromatography on silica gel using heptane as eluent to yield a light-yellow liquid (201 mg, 1.5 mmol, 51%). The analytical data were found to be good in accordance with the literature.[47]

^{1}H NMR (400 MHz, $CDCl_3$) δ 7.11 (m, 4H), 6.46 (dt, *J* = 9.6, 1.7 Hz, 1H), 6.03 (m, 1H), 2.80 (t, *J* = 8.2 Hz, 2H), 2.32 (tdd, *J* = 8.0, 4.4, 1.8 Hz, 2H).

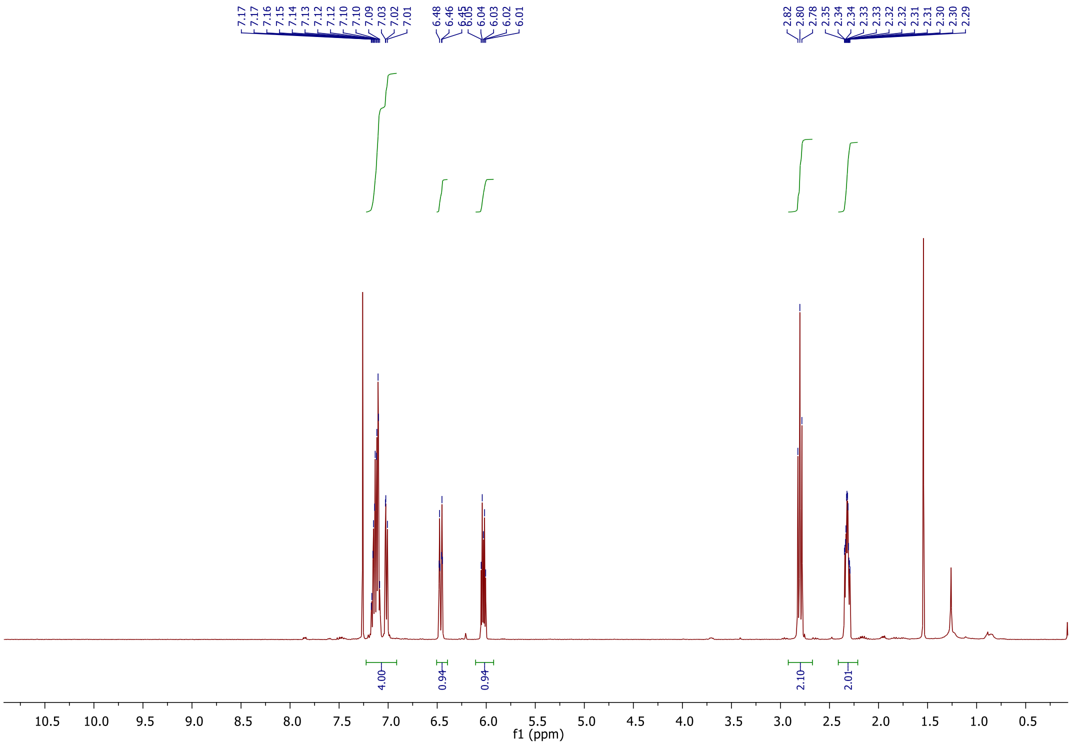

Figure S24. ^{1}H NMR (400 MHz, $CDCl_3$) of **9q**.

9r

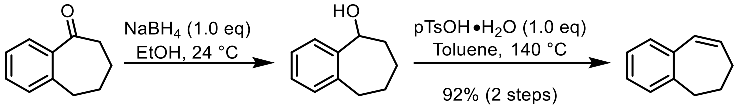

6,7,8,9-Tetrahydro-5*H*-benzocyclohepten-5-one (1.0 g, 6.2 mmol, 1.0 eq) and ethanol (15 mL) were charged into a 25 mL round-bottomed flask. The solution was cooled to 0 °C with an ice-bath and sodium borohydride (226 mg, 6.0 mmol, 0.96 eq) was added in two portions over 1 h. The mixture was stirred at 24 °C for 4 h. Afterwards, the mixture was diluted with water (20 mL) and 1 M HCl was added until no further gas evolution was observed. The mixture was diluted with EtOAc (20 mL) and the phases were separated. The aqueous phase was extracted with EtOAc (2 × 20 mL), the organic layers were combined, dried over Na_2SO_4 and concentrated under reduced pressure. To the crude mixture *p*-toluenesulfonic acid monohydrate (1187 mg, 6.24 mmol, 1.0 eq) and toluene (10 mL) were added and the mixture was heated at 140 °C for 5 h. Then, the mixture was allowed to cool to 23 °C, diluted with EtOAc (20 mL) and washed with a sat. aq. solution of $NaHCO_3$ (2 × 20 mL) followed by a sat. aq. solution of NaCl (20 mL). The organic layer was dried over Na_2SO_4 and concentrated under reduced pressure. The crude mixture was purified by flash chromatography on silica gel using heptane as eluent to yield a light-yellow liquid (826 mg, 5.7 mmol, 92%). The analytical data were found to be good in accordance with the literature.[48]

^{1}H NMR (400 MHz, $CDCl_3$) δ 7.13 (m, 4H), 6.41 (dt, *J* = 12.2, 2.0 Hz, 1H), 5.90 (dt, *J* = 12.2, 4.5 Hz, 1H), 2.85 (m, 2H), 2.43 (qd, *J* = 6.5, 2.0 Hz, 2H), 1.97 (dt, *J* = 8.4, 6.4 Hz, 2H).

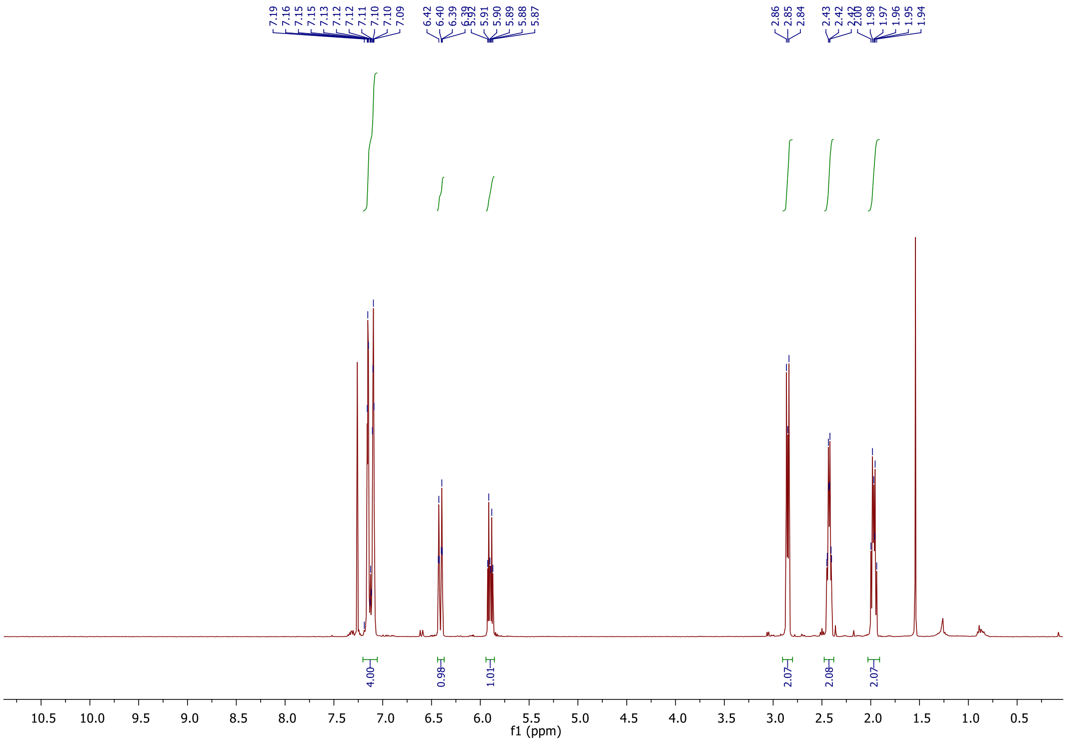

Figure S25. ^{1}H NMR (400 MHz, $CDCl_3$) of **9r**.

9w

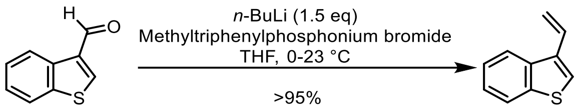

In a 50 mL round-bottomed flask, *n*-BuLi (2.5 mol/L in hexane, 1.8 mL, 4.5 mmol, 1.5 eq) was slowly added (5 min) to a cooled (~0 °C) solution of methyltriphenylphosphonium bromide (1.6 g, 4.5 mmol, 1.5 eq) in THF (26.5 mL). The mixture was stirred at this temperature for 20 min to give a red solution. Then, the red mixture was slowly transferred with a canula to a cooled (~0 °C) solution of benzo[b]thiophene-3-carboxaldehyde (497 mg, 3 mmol, 1.0 eq) in THF (8.5 mL) contained in a 50 mL round bottomed flask. After the addition, the mixture was stirred at 23 °C for 3 h and then quenched with sat. aq. solution of $NaHCO_3$ (5 mL). The phases were separated, and the organic phase was washed with a sat. aq. solution of $NaHCO_3$ (5 mL) and a sat. aq. solution of NaCl (5 mL). The organic layer was dried over Na_2SO_4 and concentrated under reduced pressure. The crude mixture was purified by flash chromatography on silica gel using heptanes as eluent to yield a colorless liquid (503 mg, 3.1 mmol, >95%). The analytical data were found to be good in accordance with the literature.[49]

^{1}H NMR (400 MHz, $CDCl_3$) δ 7.90 (m, 2H), 7.47 (s, 1H), 7.39 (dtd, *J* = 16.2, 7.2, 1.2 Hz, 2H), 6.99 (dd, *J* = 17.6, 11.1 Hz, 1H), 5.82 (dd, *J* = 17.6, 1.3 Hz, 1H), 5.39 (dd, *J* = 11.1, 1.3 Hz, 1H).

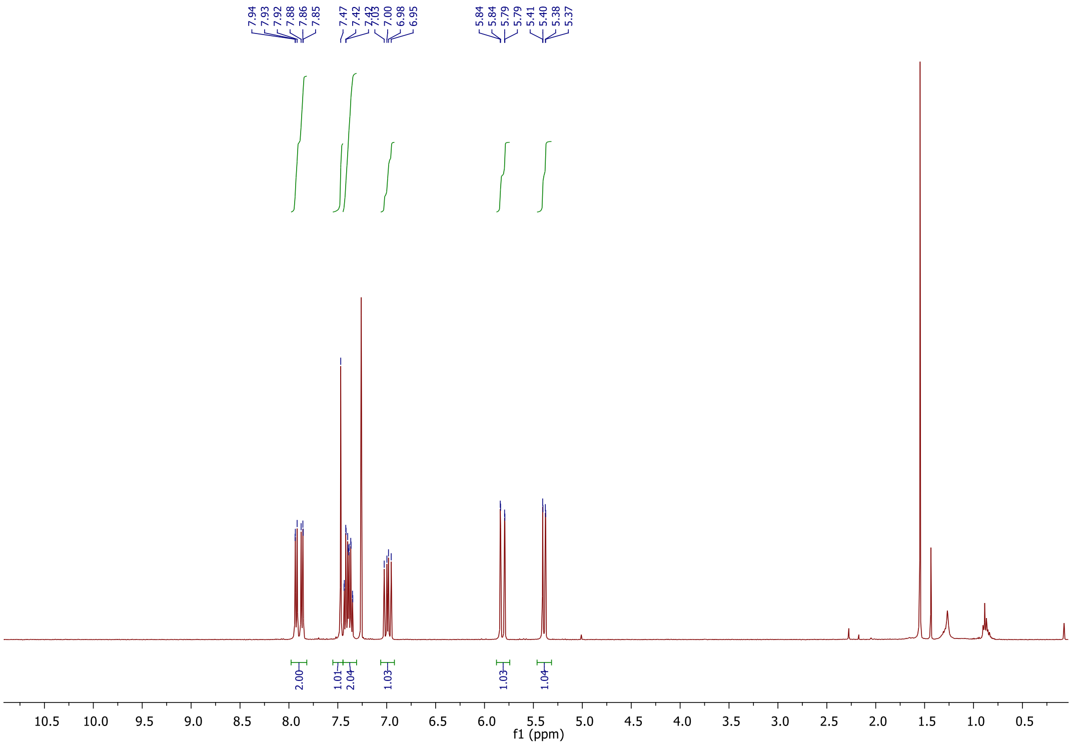

Figure S26. ^{1}H NMR (400 MHz, $CDCl_3$) of **9w**.

9x

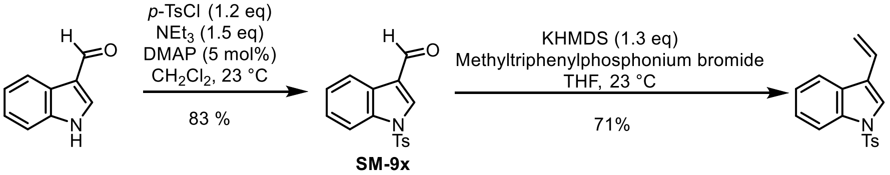

In a flame-dried 100 mL round-bottomed flask, indole-3-carboxaldehyde (499 mg, 3.44 mmol, 1.0 eq) was dissolved in CH_2Cl_2 (20 mL) under an Ar atmosphere followed by addition of *p*-TsCl (787 mg, 4.13 mmol, 1.2 eq), 4-dimethylaminopyridine (21 mg, 0.17 mmol, 0.05 eq) and Et_3N (719 µL, 5.16 mmol, 1.5 eq). The resulting mixture was stirred at 23 °C for 16 h. Then, H_2O (10 mL) was added, and the mixture was extracted with EtOAc (3 × 25 mL). The combined organic layers were dried over Na_2SO_4, filtered and concentrated under reduced pressure. The crude product was purified by flash chromatography using heptane/EtOAc as eluent to give **SM-9x** as a colorless solid (850 mg, 2.84 mmol, 83%) and the analytical data were found to be in good accordance with the literature.[50]

^{1}H NMR (400 MHz, $CDCl_3$) δ 10.10 (s, 1H), 8.36 – 8.11 (m, 2H), 7.95 (d, J = 8.2 Hz, 1H), 7.85 (d, J = 7.8 Hz, 2H), 7.39 (dt, J = 15.1, 7.3 Hz, 2H), 7.30 (d, J = 8.0 Hz, 2H), 2.38 (s, 3H).

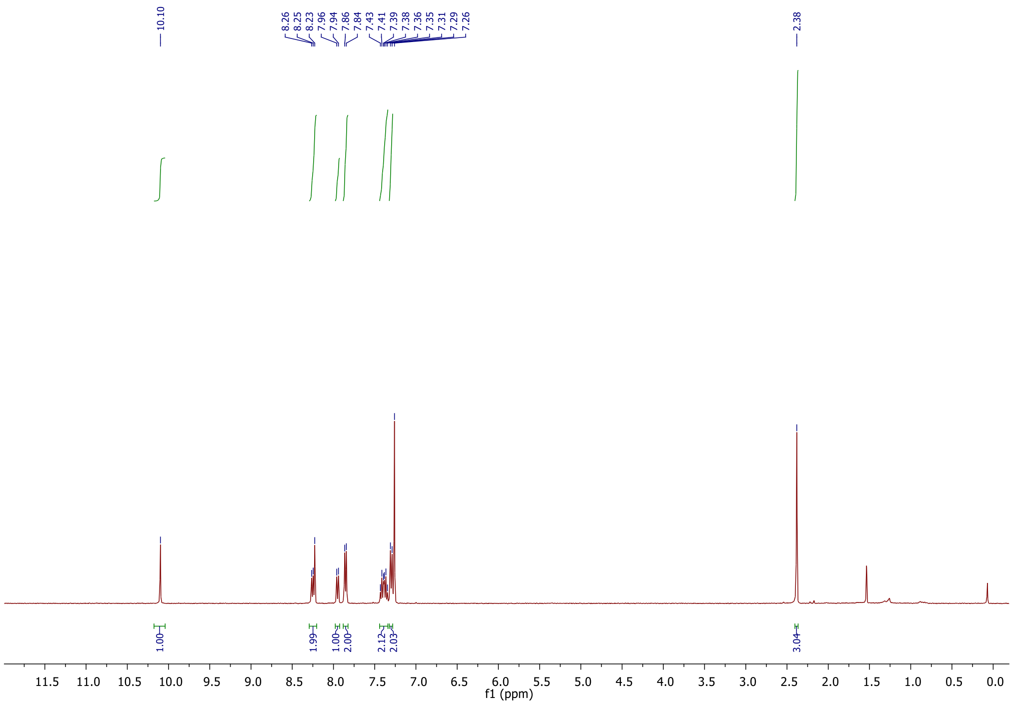

Figure S27. ^{1}H NMR (400 MHz, $CDCl_3$) of SM-**9x**.

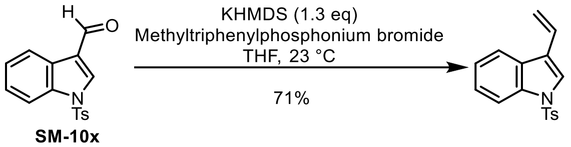

In a flame-dried 50 mL round-bottomed flask, methyltriphenylphosphonium bromide (3.0 mmol, 1.08 g, 1.5 eq) was suspended in THF (10 mL) under an Ar atmosphere. Then, KHMDS (0.5 M in toluene, 2.6 mmol, 5.2 mL, 1.3 eq) was added and the mixture was stirred at 23 °C for 2 h. Next, a solution of **SM-9x** (599 mg, 2.0 mmol, 1.0 eq) in THF (10 mL) was added dropwise to the freshly prepared ylide solution and the reaction mixture was stirred at 23 °C for additional 2 h. After quenching with a sat. aq. solution of $NaHCO_3$ and subsequent stirring for 10 min, the phases were separated and the organic phase was washed with a sat. aq. solution of $NaHCO_3$ and a sat. aq. solution of NaCl. After drying over Na_2SO_4 and filtration, the solvent was concentrated under reduced pressure (temperature of water bath set to 23 °C to avoid polymerization) and the crude product was purified by flash chromatography using heptane/EtOAc as eluent to yield the product as a white solid (422 mg, 1.42 mmol, 71%).

^{1}H NMR (400 MHz, $CDCl_3$) δ 7.99 (d, *J* = 8.1 Hz, 1H), 7.78 – 7.71 (m, 3H), 7.60 (s, 1H), 7.22 (d, *J* = 8.0 Hz, 2H), 6.77 (dd, *J* = 17.8, 11.3 Hz, 1H), 5.79 (d, *J* = 17.8 Hz, 1H), 5.35 (d, *J* = 11.2 Hz, 1H), 2.34 (s, 3H).

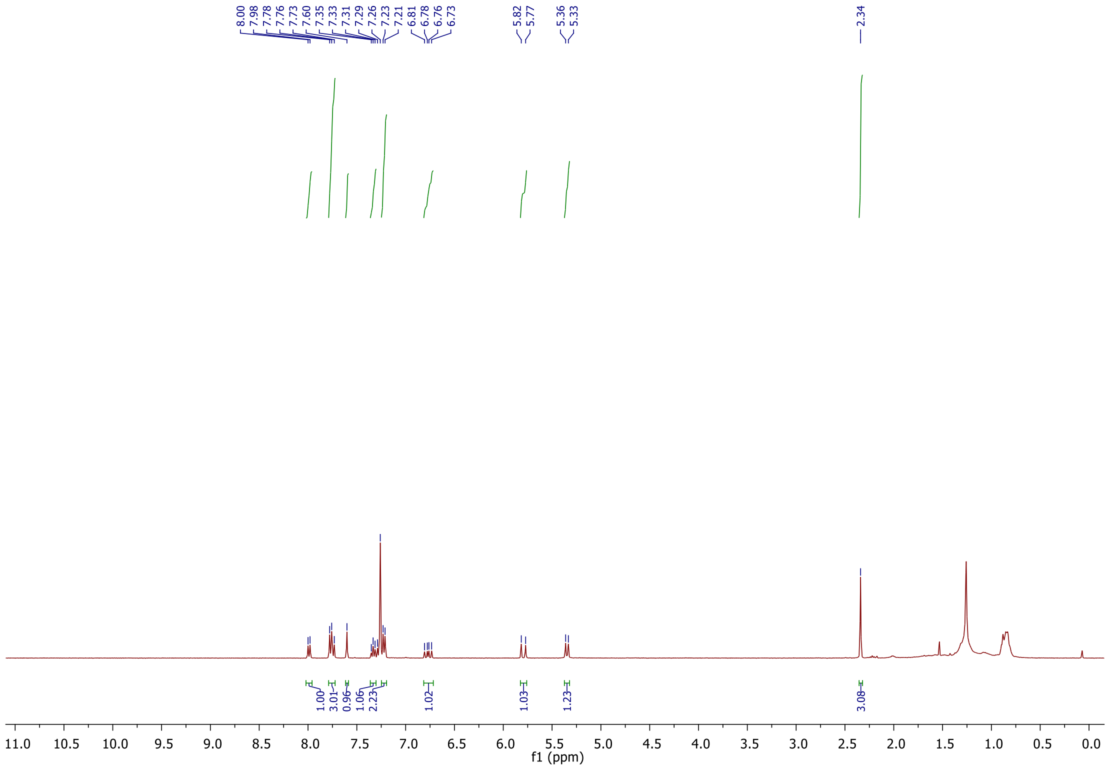

Figure S28. ^{1}H NMR (400 MHz, $CDCl_3$) of **9x**.

9y

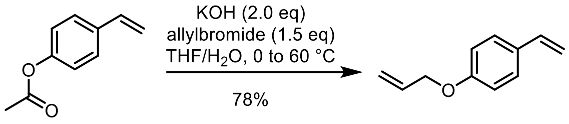

Adapted from the patent EP0881205A1.

4-Acetoxystyrene (250 mg, 1.54 mmol, 1.0 eq) and THF (5 mL) were charged into a 10 mL round-bottomed flask and the mixture was cooled with an ice-bath. KOH (173 mg, 3.08 mmol, 2.0 eq) was added followed by water (0.5 mL) and allyl bromide (200 µL, 2.31 mmol, 1.5 eq). After 30 min, the ice-bath was removed, and the mixture was heated at 60 °C for 4 h. The mixture was then allowed to return to ambient temperature and was diluted with EtOAc and water. The phases were separated and washed with a sat. aq. solution of NaCl, filtered over $MgSO_4$ and concentrated under reduced pressure. The crude mixture was purified by flash chromatography using heptane as eluent to yield a yellow oil (192 mg, 1.2 mmol, Y = 78%). The analytical data were in good accordance with literature.[51]

^{1}H NMR (400 MHz, $CDCl_3$) δ 7.34 (d, *J* = 8.7 Hz, 2H), 6.88 (d, *J* = 8.7 Hz, 2H), 6.66 (dd, *J* = 17.6, 10.9 Hz, 1H), 6.05 (ddd, *J* = 15.9, 10.6, 5.3 Hz, 1H), 5.61 (d, *J* = 17.6 Hz, 1H), 5.42 (app dd, *J* = 17.3, 1.4 Hz, 1H), 5.29 (app dd, *J* = 10.5, 1.2 Hz, 1H), 5.13 (d, *J* = 10.9 Hz, 1H), 4.55 (app d, *J* = 5.3 Hz, 2H).

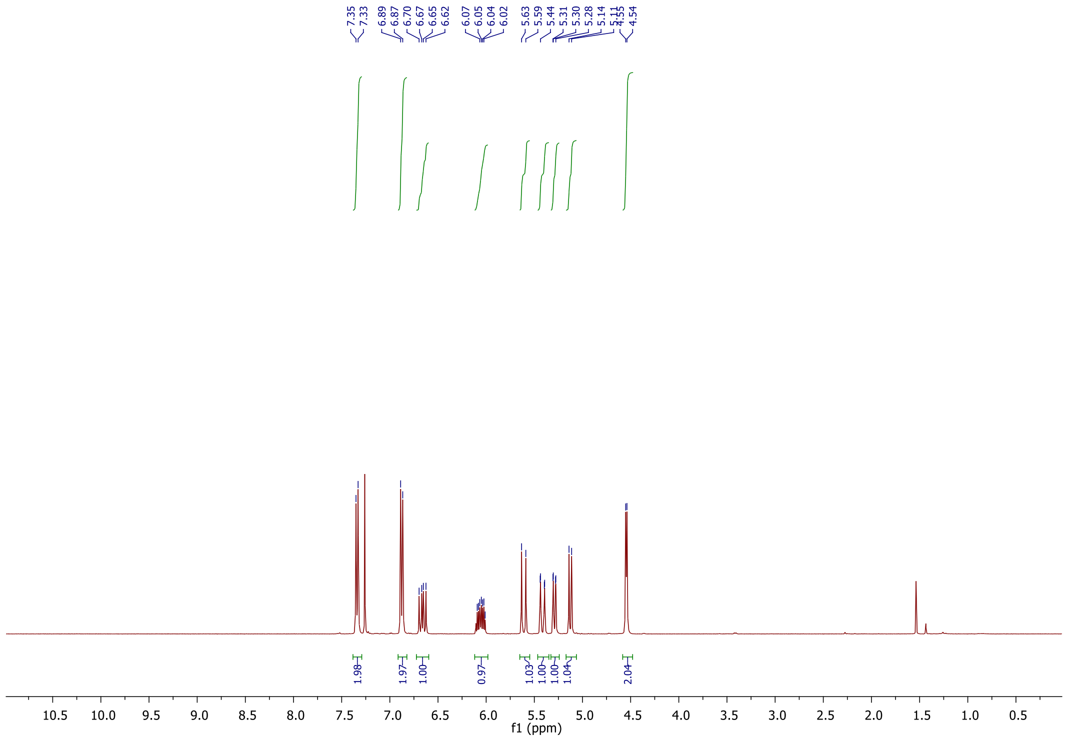

Figure S29. ^{1}H NMR (400 MHz, $CDCl_3$) of **9y**.

9z

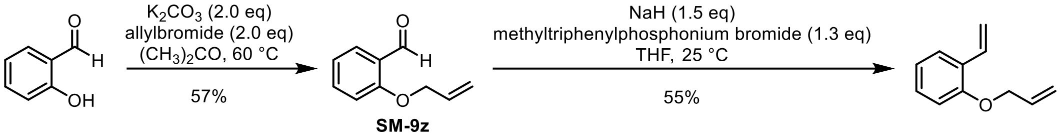

Acetone (30 mL), salicylaldehyde (1.35 mL, 10 mmol, 1.0 eq), K_2CO_3 (2.7 g, 20 mmol, 2.0 eq) and allylbromide (1.73 mL, 20 mmol, 2.0 eq) were charged into a 50 mL round-bottomed flask. The mixture was heated at 60 °C for 24 h. Afterwards, the mixture was filtered and eluted with acetone. The filtrate was concentrated under reduced pressure and the crude mixture was purified by flash chromatography using heptane/EtOAc as eluent to yield a colorless oil (0.92 g, 5.7 mmol, Y = 57%). The analytical data were found to be in good accordance with literature.[52]

^{1}H NMR (400 MHz, $CDCl_3$) δ 10.54 (s, 1H), 7.85 (dd, *J* = 7.7, 1.8 Hz, 1H), 7.58 – 7.41 (m, 1H), 7.03 (t, *J* = 7.5 Hz, 1H), 6.98 (d, *J* = 8.4 Hz, 1H), 6.08 (ddt, *J* = 17.2, 10.4, 5.1 Hz, 1H), 5.46 (ddd, *J* = 17.3, 3.1, 1.6 Hz, 1H), 5.34 (dq, *J* = 10.6, 1.3 Hz, 1H), 4.67 (dt, *J* = 5.1, 1.5 Hz, 2H).

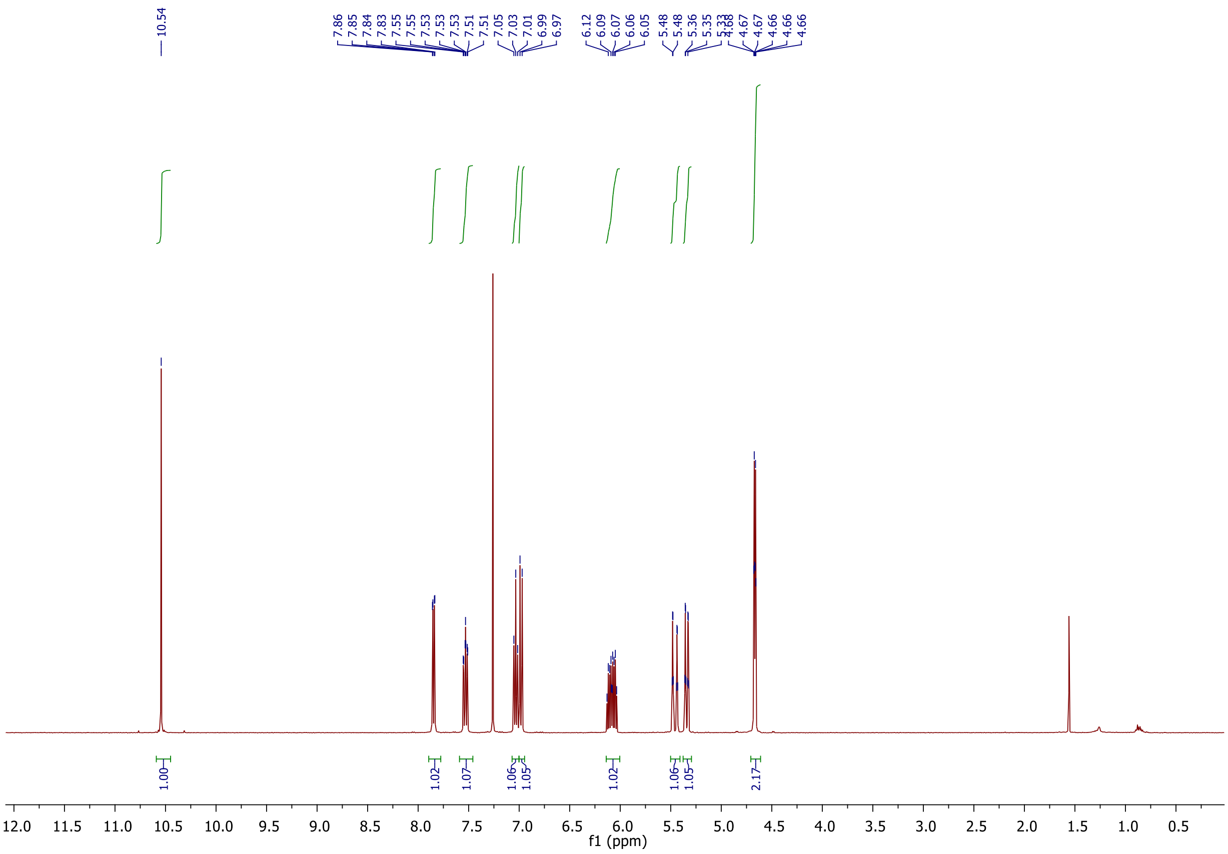

Figure S30. ^{1}H NMR (400 MHz, $CDCl_3$) of **SM-9z**.

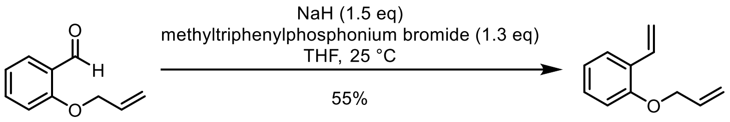

In a 100 mL round-bottomed flask was charged NaH (60%, 340 mg, 8.51 mmol, 1.5 eq) followed by THF (30 mL). The suspension was cooled to 0 °C and methyltriphenylphosphonium bromide (2.63 g, 7.37 mmol, 1.3 eq) was added portionwise. After 30 min, a solution of **SM-9z** (0.92 g, 5.67 mmol, 1.0 eq) in THF (10 mL) was added and the mixture was stirred at 25 °C for 4 h. Then, the solution was poured into water and diluted with EtOAc. The phases were separated and the aqueous phase was extracted again with EtOAc. Organic phases were combined, washed with a sat. aq. sol. of NaCl, dried over Na_2SO_4, filtered and concentrated under reduced pressure. The crude mixture was purified by flash chromatography using heptane/EtOAc as eluent to yield a light-yellow oil (495 mg, 3.09 mmol, Y = 55%). Analytical data was in good accordance with literature.[53]

^{1}H NMR (400 MHz, $CDCl_3$) δ 7.49 (dd, *J* = 7.6, 1.5 Hz, 1H), 7.24 – 7.16 (m, 1H), 7.10 (dd, *J* = 17.8, 11.2 Hz, 1H), 6.94 (t, *J* = 7.5 Hz, 1H), 6.87 (d, *J* = 8.3 Hz, 1H), 6.08 (ddt, *J* = 16.9, 10.4, 5.1 Hz, 1H), 5.75 (dd, *J* = 17.8, 1.4 Hz, 1H), 5.43 (dd, *J* = 17.3, 1.5 Hz, 1H), 5.33 – 5.22 (m, 2H), 4.63 – 4.52 (m, 2H).

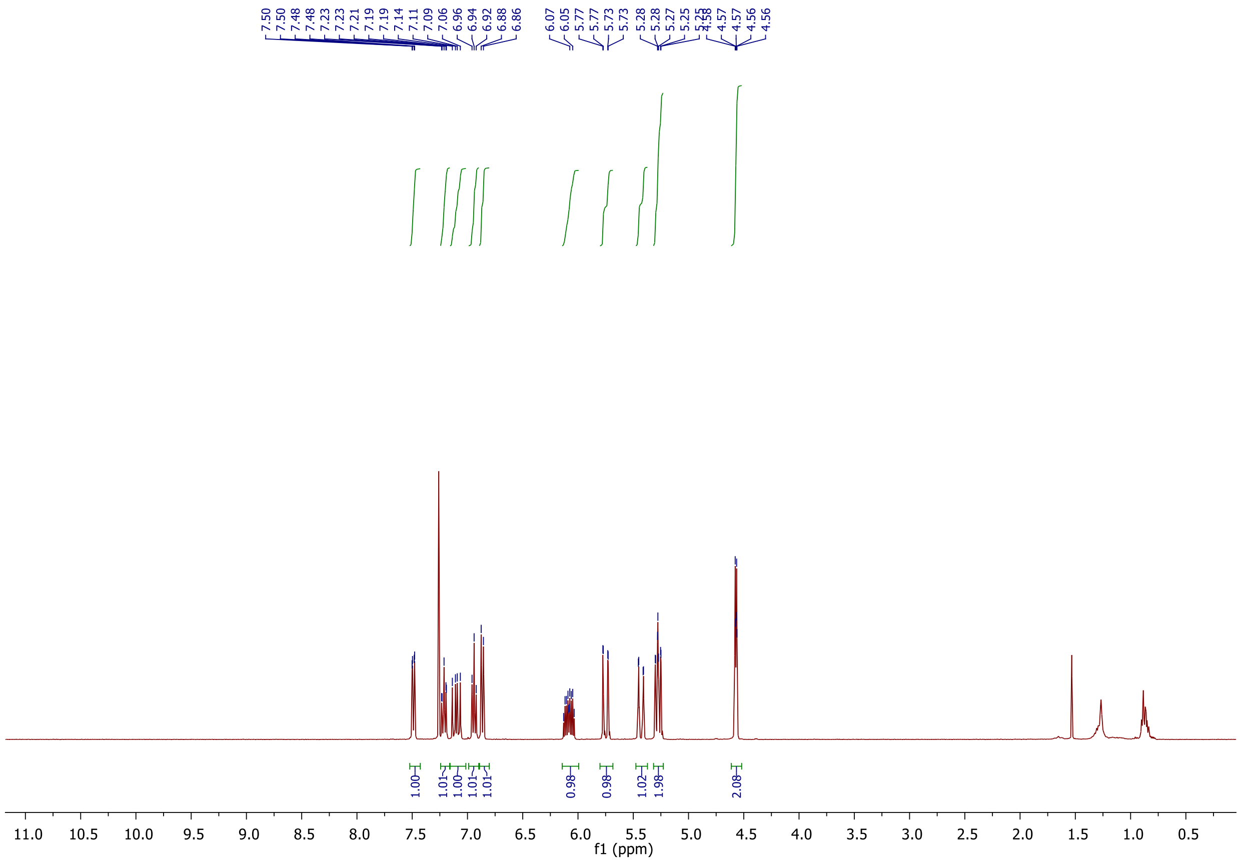

Figure S31. ^{1}H NMR (400 MHz, $CDCl_3$) of **9z**.

9aa

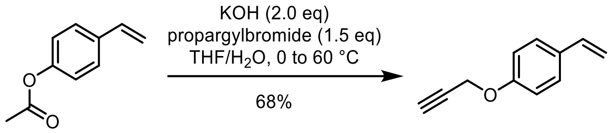

Adapted from the patent EP0881205A1.[4]

4-Acetoxystyrene (500 mg, 3.08 mmol, 1.0 eq) and THF (5 mL) were charged into 10 mL round-bottomed flask and the mixture was cooled with an ice-bath. KOH (1346 mg, 6.17 mmol, 2.0 eq) was added followed by water (0.5 mL) and propargyl bromide (515 µL, 4.62 mmol, 1.5 eq). After 30 min, the ice-bath was removed, and the mixture was heated at 60 °C for 4 h. The mixture was then allowed to cool to ambient temperature and was diluted with EtOAc and water. The phases were separated and washed with a sat. aq. solution of NaCl, filtered over $MgSO_4$ and concentrated under reduced pressure. The crude mixture was purified by flash chromatography using heptane as eluent to yield a yellow oil (332 mg, 2.1 mmol, Y = 68%) and analytical data were found to be in good accordance with the literature.[54]

^{1}H NMR (400 MHz, $CDCl_3$) δ 7.36 (d, *J* = 8.6 Hz, 2H), 6.94 (d, *J* = 8.7 Hz, 2H), 6.67 (dd, *J* = 17.6, 10.9 Hz, 1H), 5.63 (dd, *J* = 17.6, 0.5 Hz, 1H), 5.15 (d, *J* = 10.9 Hz, 1H), 4.70 (d, *J* = 2.3 Hz, 2H), 2.52 (t, *J* = 2.3 Hz, 1H).

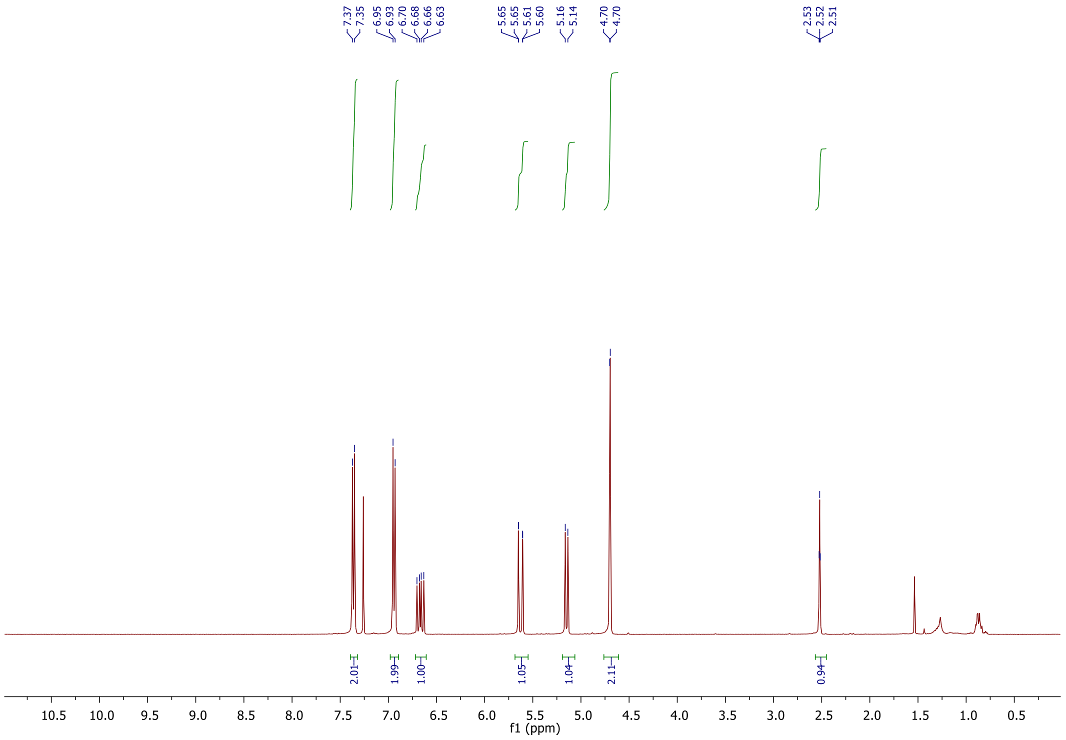

Figure S32. ^{1}H NMR (400 MHz, $CDCl_3$) of **9aa**.

9ab

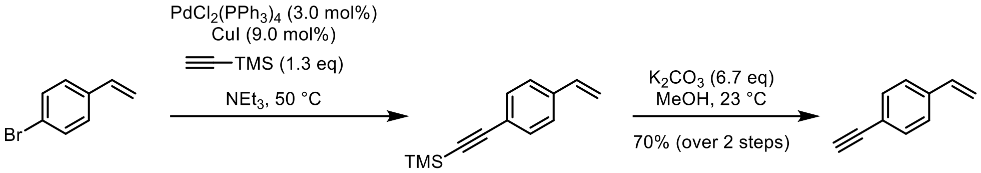

In a flame-dried 25 mL Schlenk flask were placed $PdCl_2(PPh_3)_2$ (63.2 mg, 0.09 mmol, 0.03 eq), CuI (51.7 mg, 0.27 mmol, 0.09 eq), NEt_3 (6.0 mL, 43.2 mmol, 14.0 eq), 4-bromostyrene (549 mg, 3.0 mmol, 1.0 eq) and trimethylsilylacetylene (551 μL, 3.9 mmol, 1.3 eq) under an Ar atmosphere. The reaction mixture was heated to 50 °C and stirred for 16 h. After cooling to ambient temperature, the reaction mixture was poured into a sat. aq. solution of NH_4Cl and stirred vigorously for 15 min. The phases were separated, and the aqueous phase was extracted with Et_2O (3 × 10 mL). The combined organic phases were washed with water, a sat. aq. solution of NaCl, dried over anhydrous Na_2SO_4, filtered through a silica pad and finally the volatiles were carefully removed under reduced pressure (***product is volatile***). The crude product obtained previously was dissolved in MeOH (20 mL) and K_2CO_3 (2.76 g, 20.0 mmol, 6.7 eq) was added in one portion at 23 °C and the reaction mixture was stirred for 18 h. Afterwards, a sat. aq. solution of NH_4Cl was carefully added followed by Et_2O (50 mL). The phases were separated, and the aqueous phase was extracted with Et_2O (3 × 10 mL). The organic phases were combined, washed with H_2O, a sat. aq. solution of NaCl, dried over Na_2SO_4, filtered and carefully concentrated under reduced pressure. The crude product was purified by flash chromatography using pentane as eluent to yield the product as a colorless oil (270 mg, 2.11 mmol, Y = 70 %). The analytical data were in good accordance with the literature.[55]

^{1}H NMR (400 MHz, $CDCl_3$) δ 7.36 (d, *J* = 8.6 Hz, 2H), 6.94 (d, *J* = 8.7 Hz, 2H), 6.67 (dd, *J* = 17.6, 10.9 Hz, 1H), 5.63 (dd, *J* = 17.6, 0.5 Hz, 1H), 5.15 (d, *J* = 10.9 Hz, 1H), 4.70 (d, *J* = 2.3 Hz, 2H), 2.52 (t, *J* = 2.3 Hz, 1H).

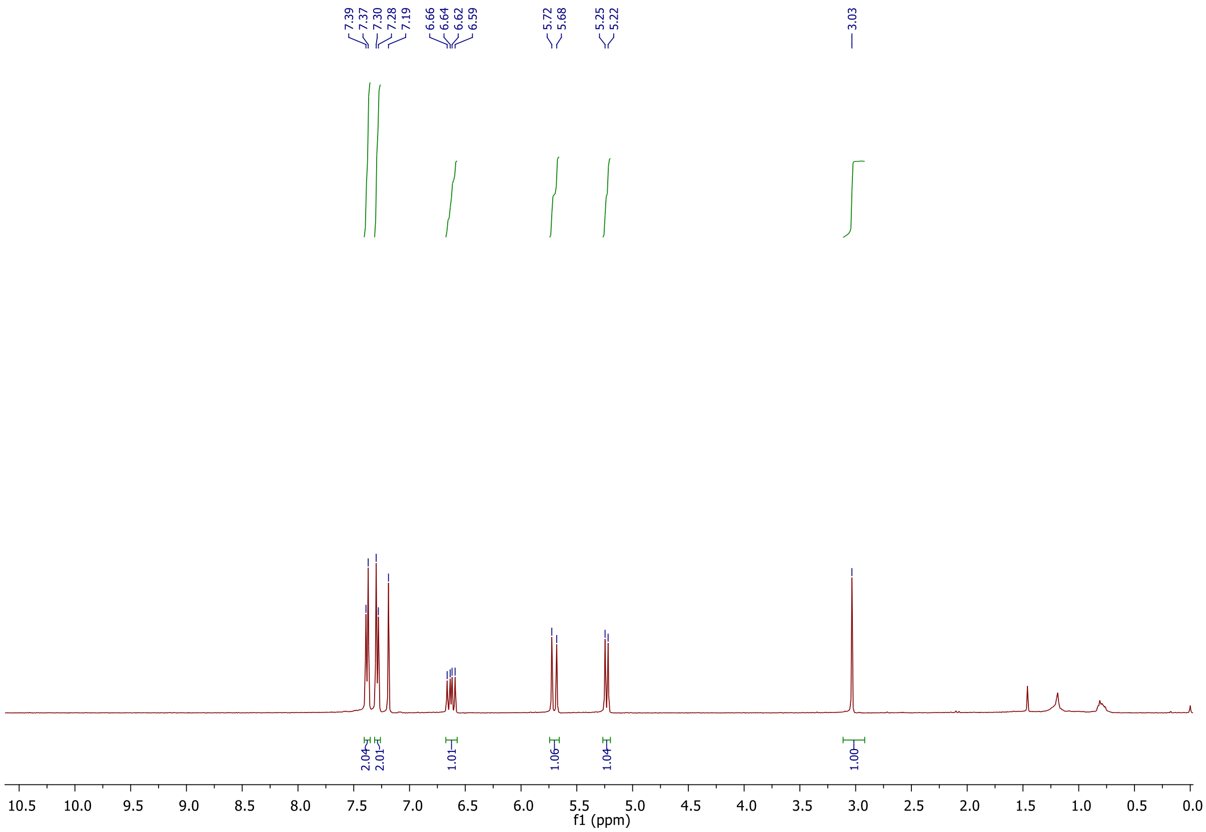

Figure S33. ^{1}H NMR (400 MHz, $CDCl_3$) of **9ab**.

9ac

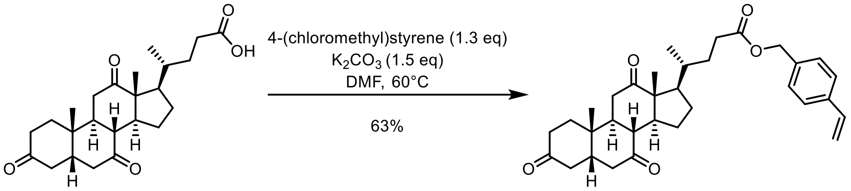

Dehydrocholic acid (805 mg, 2.0 mmol, 1.0 eq), 4-(chloromethyl)styrene (90%, 0.4 mL, 2.6 mmol, 1.3 eq) and potassium carbonate (415 mg, 3.0 mmol, 1.5 eq) were added successively into a 10 mL round-bottomed flask. DMF (4 mL) was added and the mixture was heated at 60 °C for 16 h. Afterwards, the mixture was allowed to cool to 23 °C and diluted with water (20 mL) and EtOAc (20 mL). The phases were separated and the aqueous phase was extracted with EtOAc (2 × 20 mL). The organic layers were combined, dried over Na_2SO_4 and concentrated under reduced pressure. The crude mixture was purified by flash chromatography on silica gel using heptane/EtOAc as eluent to yield a white solid (655 mg, 1.3 mmol, Y = 63%).

^{1}H NMR (400 MHz, $CDCl_3$) δ 7.40 (d, J = 8.2 Hz, 2H), 7.31 (d, J = 8.1 Hz, 2H), 6.71 (dd, J = 17.6, 10.9 Hz, 1H), 5.75 (d, J = 17.6 Hz, 1H), 5.26 (d, J = 10.9 Hz, 1H), 5.09 (m, 2H), 2.96 – 2.78 (m, 3H), 2.44 (m, 1H), 2.37 – 2.23 (m, 6H), 2.21 – 2.17 (m, 1H), 2.12 – 2.07 (m, 2H), 2.06 – 1.90 (m, 4H), 1.91 – 1.76 (m, 2H), 1.62 (m, 1H), 1.43 – 1.37 (m, 4H), 1.30 – 1.21 (m, 3H), 1.03 (s, 3H), 0.84 (d, J = 6.6 Hz, 3H).

^{13}C NMR (151 MHz, $CDCl_3$) δ 212.0, 209.2, 208.8, 174.0, 137.7, 136.5, 135.7, 128.7 (2C), 126.5 (2C), 114.4, 77.4, 66.0, 57.0, 51.9, 49.1, 47.0, 45.8, 45.7, 45.1, 42.9, 38.8, 36.6, 36.1, 35.6, 35.4, 31.7, 30.6, 27.7, 25.3, 22.0, 18.8, 11.9.

IR (neat) ν_{max}: 2977, 1737, 1712, 1168.

HRMS (ESI$^+$): *m/z* calculated for $[M+Na]^+$ ($C_{33}H_{42}NaO_5^+$) = 541.2924, found *m/z* = 541.2911.

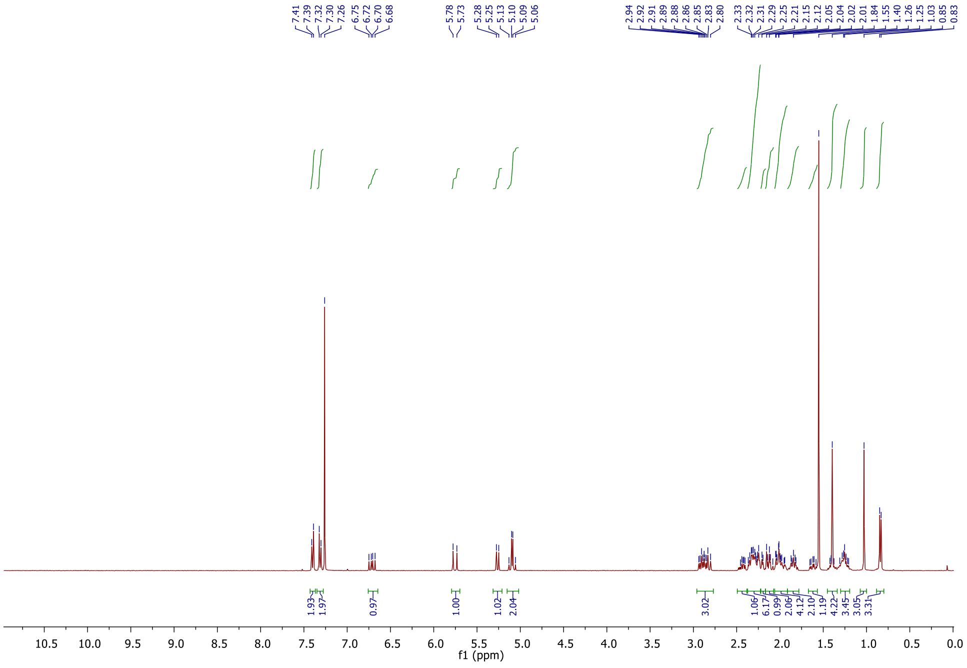

Figure S34. ^{1}H NMR (400 MHz, $CDCl_3$) of **9ac**.

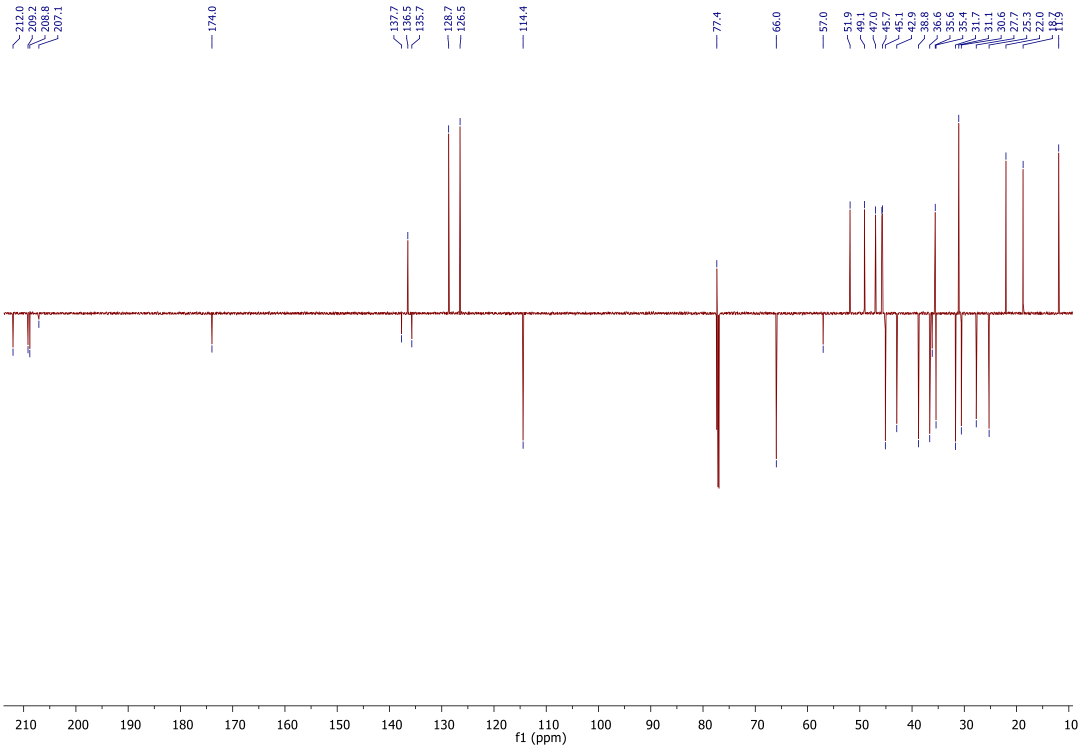

Figure S35. ^{13}C NMR (151 MHz, $CDCl_3$) of **9ac**.

9ad

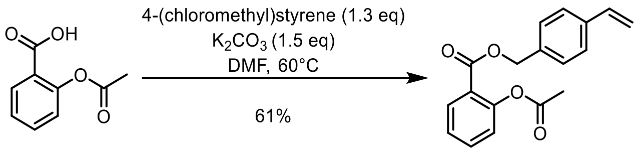

Aspirin (364 mg, 2.0 mmol, 1.0 eq), 4-(chloromethyl)styrene (90%, 0.4 mL, 2.6 mmol, 1.3 eq) and potassium carbonate (415 mg, 3.0 mmol, 1.5 eq) were added successively to a 10 mL round-bottomed flask. DMF (4 mL) was added and the mixture was heated at 60 °C for 16 h. Then, the mixture was allowed to cool to 23 °C and was diluted with water (20 mL) and EtOAc (20 mL). The phases were separated and the aqueous phase was extracted with EtOAc (2 × 20 mL). The organic layers were combined, dried over Na_2SO_4 and concentrated under reduced pressure. The crude mixture was purified by flash chromatography over silica gel using heptanes/EtOAc to yield a light-yellow oil (363 mg, 1.2 mmol, Y = 61%). The analytical data were in accordance with the literature.[56]

^{1}H NMR (400 MHz, CDCl$_3$) δ 8.06 (dd, *J* = 7.9, 1.7 Hz, 1H), 7.56 (td, *J* = 7.8, 1.7 Hz, 1H), 7.41 (dd, *J* = 22.1, 8.2 Hz, 4H), 7.31 (td, *J* = 7.7, 1.1 Hz, 1H), 7.09 (dd, *J* = 8.1, 1.0 Hz, 1H), 6.73 (dd, *J* = 17.6, 10.9 Hz, 1H), 5.78 (m, 1H), 5.28 (m, 3H), 2.14 (s, 3H).

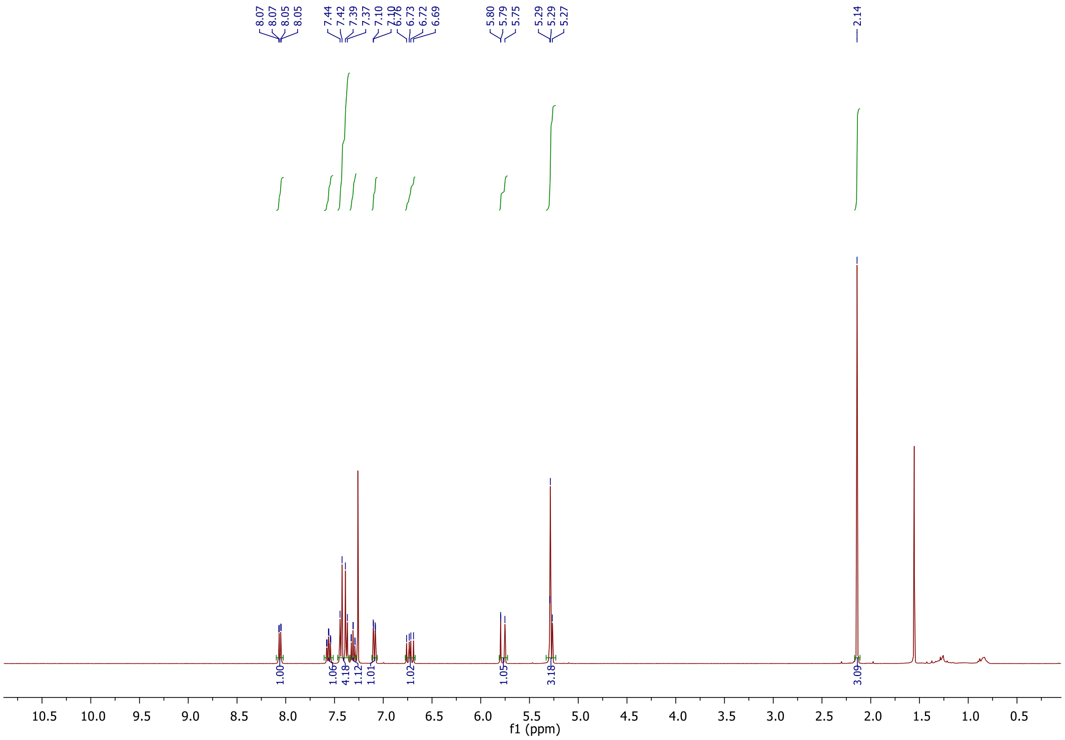

Figure S36. ^{1}H NMR (400 MHz, $CDCl_3$) of **9ad**.

Oxaprozin

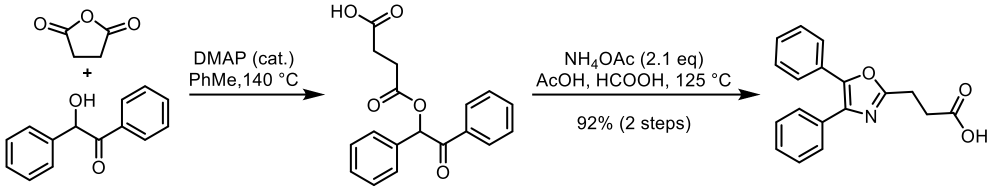

Adapted from the patent US6096896A.[57]

Benzoin (1750 mg, 8.1 mmol, 1.0 eq), succinic anhydride (1000 mg, 10.0 mmol, 1.2 eq) and 4-dimethylaminopyridine (100 mg, 0.82 mmol, 0.1 eq) were added successively into a 25 mL round-bottomed flask. Toluene (8.4 mL) was added and the mixture was stirred at 140 °C for 4 h and then at 24 °C for 16 h. The crude mixture was concentrated under reduced pressure and used as such in the next step without further purification. Acetic acid (5 mL, 5.2 mmol), formic acid (0.8 mL, 22 mmol) and ammonium acetate (1.3 g, 17 mmol) were added successively to the previously obtained residue. The mixture was heated at 125 °C for 16 h and then allowed to cool to 23 °C. The crude product was filtered, washed with water and concentrated under reduced pressure to yield a white solid (2160 mg, 7.4 mmol, 92%). The analytical data were in accordance with the literature.[58]

^{1}H NMR (400 MHz, $CDCl_3$) δ 7.60 (m, 4H), 7.35 (m, 6H), 3.20 (t, J = 7.3 Hz, 2H), 2.97 (t, J = 7.3 Hz, 2H).

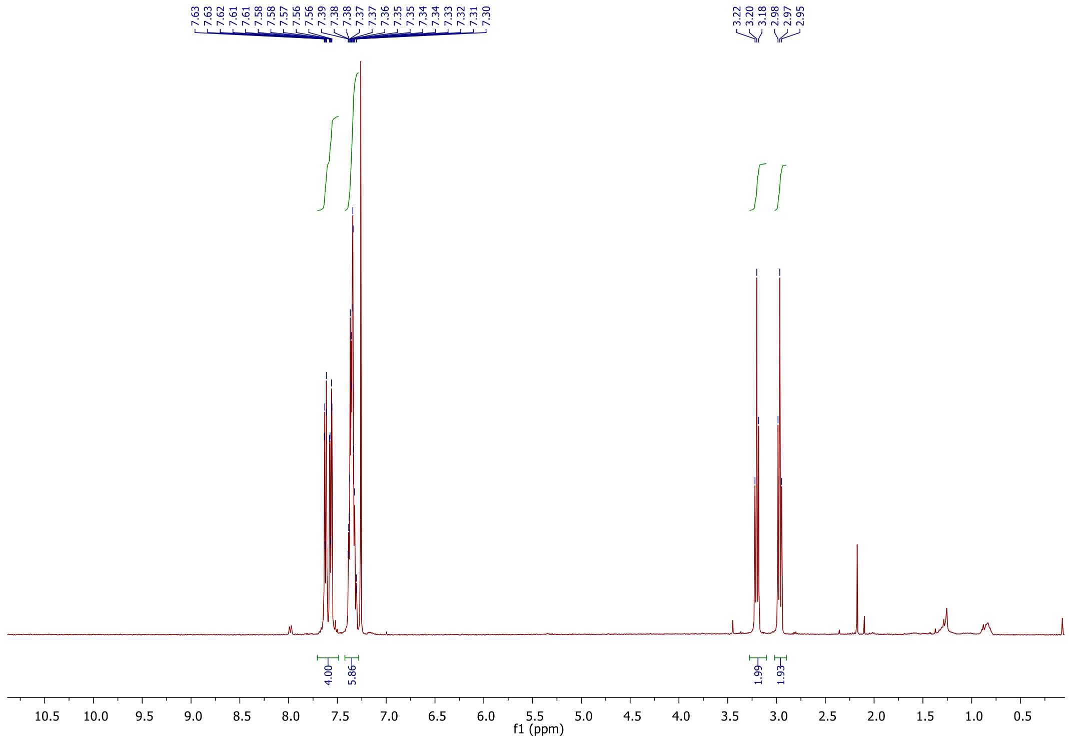

Figure S37. ^{1}H NMR (400 MHz, $CDCl_3$) of **oxaprozin**.

9ae

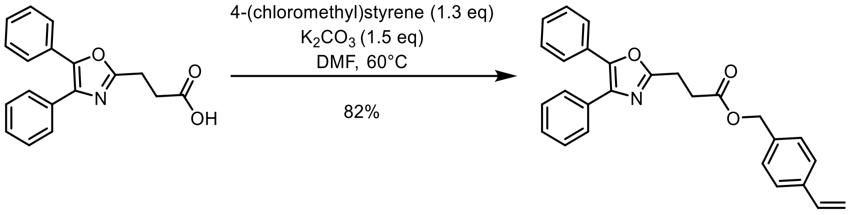

Oxaprozin (2171 mg, 7.4 mmol, 1.0 eq), 4-(chloromethyl)styrene (90%, 1.5 mL, 9.62 mmol, 1.3 eq) and potassium carbonate (1534 mg, 11.1 mmol, 3.0 eq) were charged successively into a 25 mL round-bottomed flask. DMF (15 mL) was added and the mixture was heated at 60 °C for 16 h. Then, the mixture was allowed to cool to 23 °C and was diluted with water (20 mL) and EtOAc (20 mL). The phases were separated and the aqueous phase was extracted with EtOAc (2 × 20 mL). The organic layers were combined, dried over Na_2SO_4 and concentrated under reduced pressure. The crude mixture was purified by flash chromatography on silica gel using heptane/EtOAc as eluent to yield a light-yellow oil (2.5 g, 6.1 mmol, 82%). The analytical data were in accordance with the literature.[56]

^{1}H NMR (400 MHz, $CDCl_3$) δ 7.61 (ddd, *J* = 11.0, 6.7, 4.4 Hz, 2H), 7.54 (m, 2H), 7.34 (m, 10H), 6.68 (dd, *J* = 17.6, 10.9 Hz, 1H), 5.72 (m, 1H), 5.25 (d, *J* = 10.9 Hz, 1H), 5.15 (s, 2H), 3.21 (t, *J* = 7.4 Hz, 2H), 2.97 (t, *J* = 7.4 Hz, 2H).

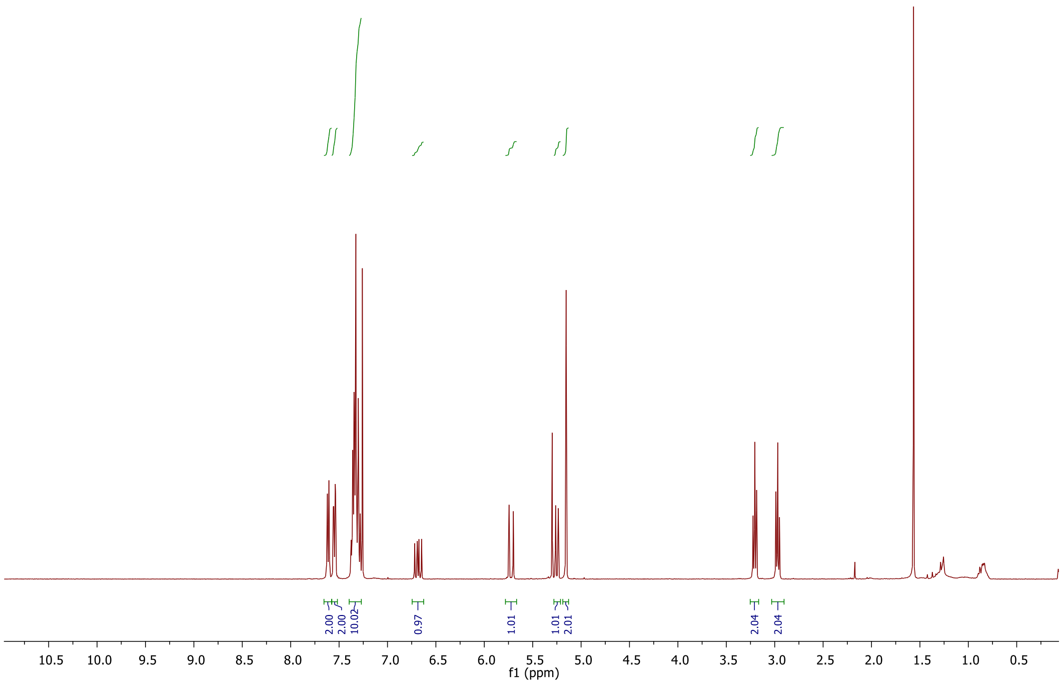

Figure S38. ^{1}H NMR (400 MHz, $CDCl_3$) of **9ae**.

9af

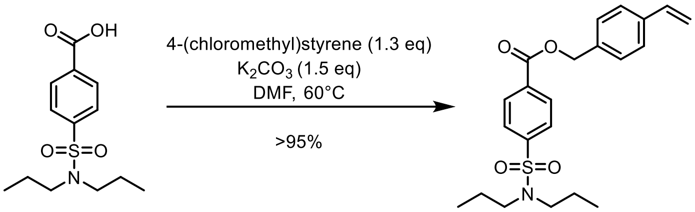

Probenecid (582 mg, 2.0 mmol, 1.0 eq), 4-(chloromethyl)styrene (90%, 0.4 mL, 2.6 mmol, 1.3 eq) and potassium carbonate (415 mg, 3.0 mmol, 1.5 eq) were charged successively into a 10 mL round-bottomed flask. DMF (4 mL) was added and the mixture was heated at 60 °C for 16 h. After this time, the mixture was allowed to cool to 23 °C and it was diluted with water (20 mL) and EtOAc (20 mL). The phases were separated and the aqueous phase was extracted with EtOAc (2 × 20 mL). The organic layers were combined, dried over Na_2SO_4 and concentrated under reduced pressure. The crude mixture was purified by flash chromatography on silica gel using heptane/EtOAc as eluent to yield a light green oil (875 mg, 2.2 mmol, > 95%). The analytical data were in accordance with the literature.[56]

^{1}H NMR (400 MHz, CDCl$_3$) δ 8.18 (d, *J* = 8.5 Hz, 2H), 7.87 (d, *J* = 8.5 Hz, 2H), 7.43 (q, *J* = 8.4 Hz, 4H), 6.73 (dd, *J* = 17.6, 10.9 Hz, 1H), 5.78 (d, *J* = 17.6 Hz, 1H), 5.37 (s, 2H), 5.29 (d, *J* = 10.9 Hz, 1H), 3.09 (m, 4H), 1.57 (d, *J* = 7.5 Hz, 1H), 1.52 (dd, *J* = 15.1, 7.5 Hz, 3H), 0.86 (t, *J* = 7.4 Hz, 6H).

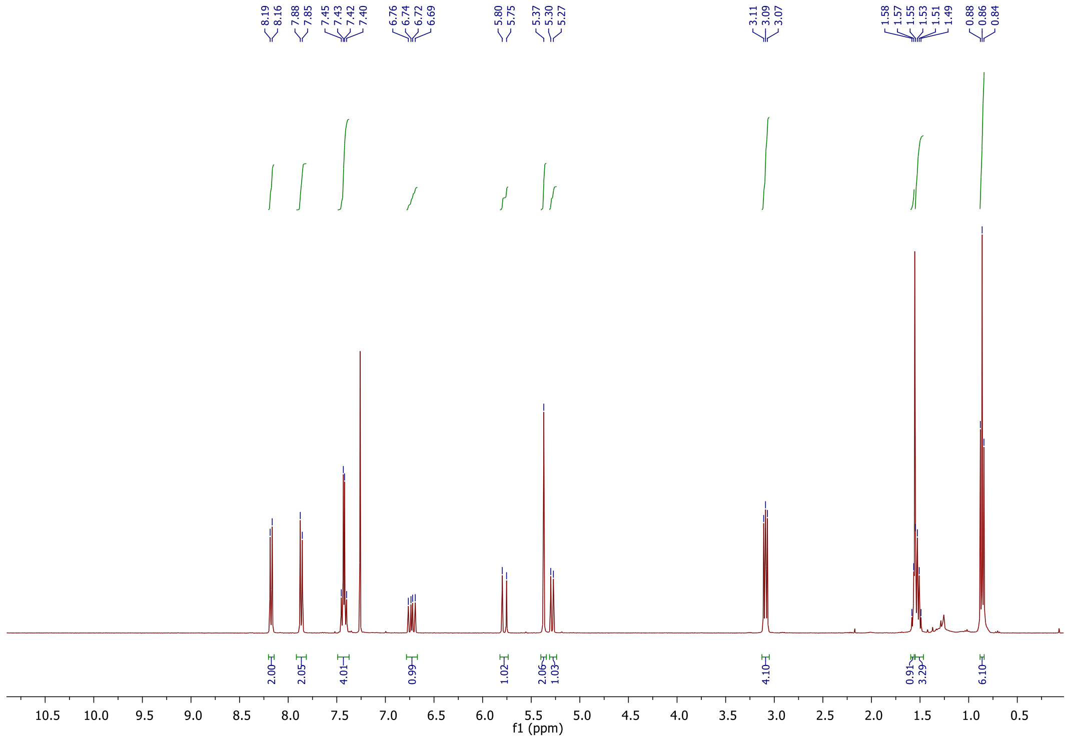

Figure S39. ^{1}H NMR (400 MHz, $CDCl_3$) of **9af**.

Synthesis of the ketone products

General procedure

In a vial containing the Co precatalyst (2.9 mg, 1 mol%) and a stirring bar, the substrate was added directly as a solid (0.5 mmol, 1.0 eq) followed by EtOH (4 mL) or as a solution (0.5 mmol in 4 mL of EtOH). The reaction mixture was stirred at 1000 rpm without being in direct contact with the stirring plate. After 1 min, phenylsilane (62 μL, 0.5 mmol, 1.0 eq) was added and the mixture was stirred for 18 h at room temperature (22 – 27 °C) under ambient air. The mixture was concentrated under reduced pressure and purified by flash chromatography using the adequate solvent system.

10a

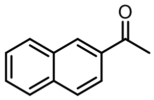

Synthesized according to the general procedure using 77.1 mg of 2-vinylnaphthalene. The product was purified by flash chromatography using heptane/EtOAc as eluent to yield a waxy solid that solidified overtime (64.5 mg, 0.38 mmol, Y = 76%). The analytical data was in good accordance with the literature.[59]

Gram-scale experiments: In a flask charged with the Co precatalyst (37.9 mg, 1 mol% or 18.9 mg, 0.5 mol%) and a stirring bar, 2-vinylnaphthalene (6.48 mmol, 1.0 g, 1.0 eq) was added followed by EtOH (52 mL). The reaction mixture was stirred at 1000 rpm and after 1 min, phenylsilane (800 µL, 6.48 mmol, 1.0 eq) was added and the mixture was stirred for 18 h at room temperature (22 °C) under ambient air. The mixture was concentrated under reduced pressure and purified by flash chromatography using heptane/EtOAc as eluent to yield a waxy solid that solidified overtime yielding respectively, 937 mg (5.51 mmol, Y = 85%) and 794 mg (4.67 mmol, Y = 72%) of the desired product.

^{1}H NMR (400 MHz, $CDCl_3$) δ 8.48 (s, 1H), 8.04 (dd, *J* = 8.6, 1.6 Hz, 1H), 7.98 (d, *J* = 8.0 Hz, 1H), 7.93 – 7.85 (m, 2H), 7.59 (ddd, *J* = 14.9, 13.6, 6.8 Hz, 2H), 2.74 (s, 3H).

HRMS (EI): exact mass calculated for $[M]^+$ ($C_{12}H_{10}O^{\bullet+}$) requires *m/z* = 170.0732, found *m/z* = 170.0726.

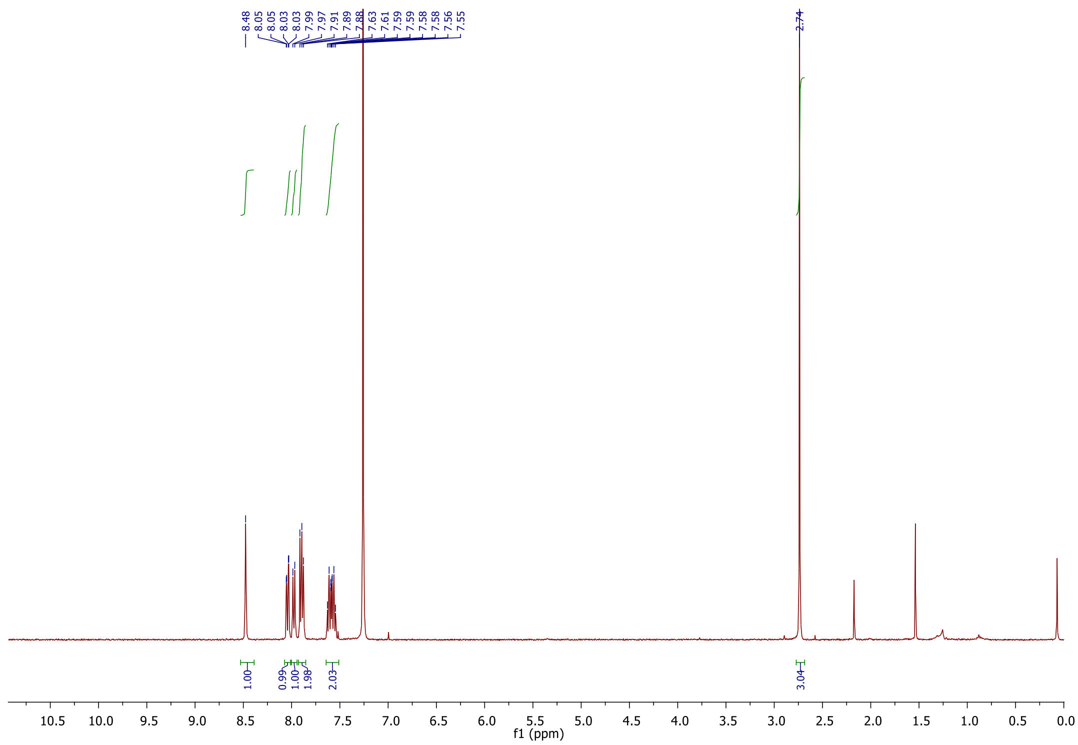

Figure S40. ^{1}H NMR (400 MHz, $CDCl_3$) of **10a**.

10b

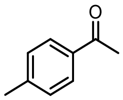

Synthesized according to the general procedure using 59.1 mg of 4-methylstyrene. The product was purified by flash chromatography using heptane/EtOAc as eluent to yield a colorless oil (56 mg, 0.42 mmol, Y = 84%). The analytical data was in good accordance with literature.[60]

^{1}H NMR (400 MHz, $CDCl_3$) δ 7.86 (d, *J* = 8.2 Hz, 2H), 7.26* (d, *J* = 8.2 Hz, 2H), 2.58 (s, 3H), 2.41 (s, 3H). *Solvent peak in the doublet

HRMS (EI): exact mass calculated for $[M-CH_3]^+$ ($C_8H_7O^{\bullet+}$) requires *m/z* = 119.0497, found *m/z* = 119.0487

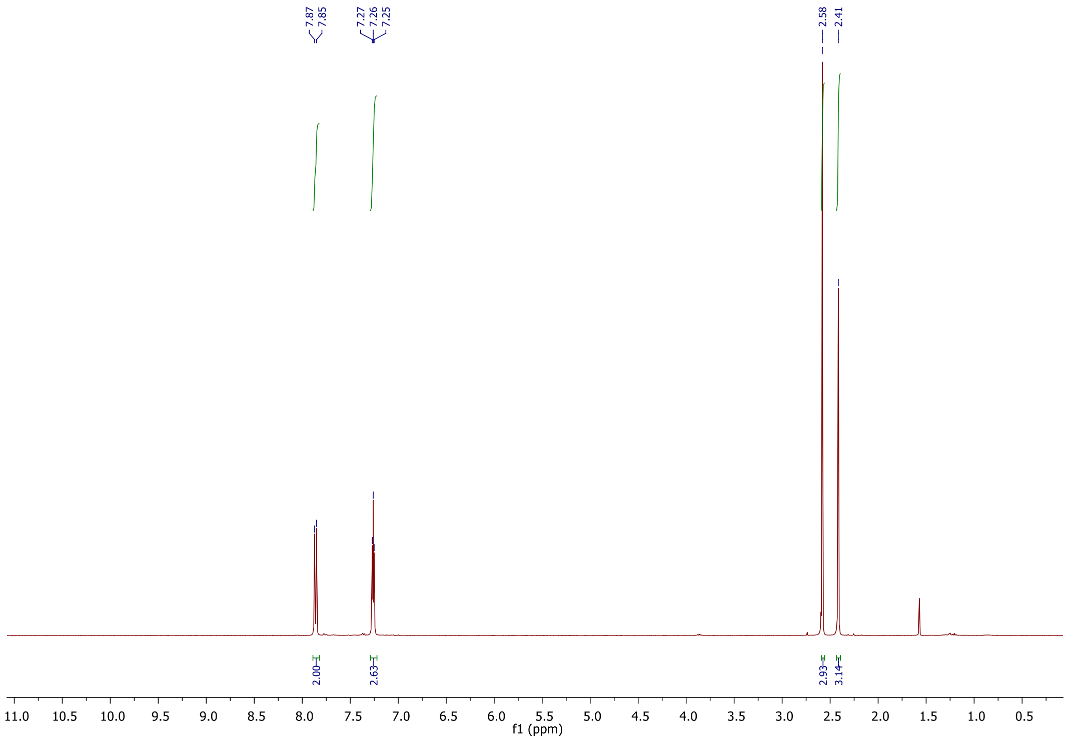

Figure S41. ^{1}H NMR (400 MHz, $CDCl_3$) of **10b**.

10c

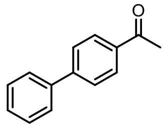

Synthesized according to the general procedure from 90.1 mg of 4-vinylbiphenyl. The product was purified by flash chromatography using heptane/toluene as eluent to yield a white solid (75 mg, 0.38 mmol, Y = 76%). The analytical data was in good accordance with literature.[61]

^{1}H NMR (400 MHz, CDCl$_3$) δ 8.04 (m, 2H), 7.69 (m, 2H), 7.63 (m, 2H), 7.48 (m, 2H), 7.41 (m, 1H), 2.64 (s, 3H).

HRMS (EI): exact mass calculated for $[M]^+$ ($C_{14}H_{12}O^{\bullet+}$) requires *m/z* = 196.0888, found *m/z* = 196.0879.

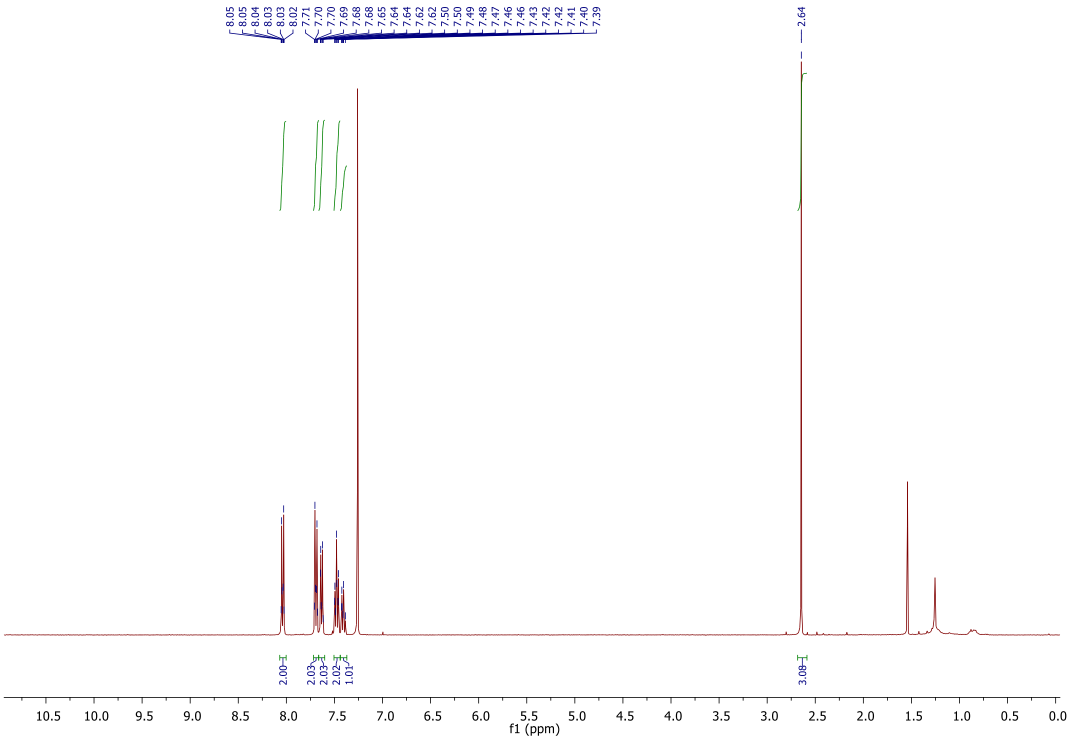

Figure S42. ^{1}H NMR (400 MHz, CDCl$_3$) of **10c**.

10d

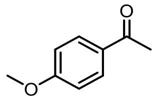

Synthesized according to the general procedure using 70.6 mg of 4-methoxystyrene (95% pure). The product was purified by flash chromatography using heptane/DCM as eluent to yield a colorless oil (56.8 mg, 0.38 mmol, Y = 76%). The analytical data was in good accordance with the literature.[62]

^{1}H NMR (400 MHz, $CDCl_3$) δ 7.92 (d, *J* = 8.8 Hz, 2H), 6.92 (d, *J* = 8.8 Hz, 2H), 3.85 (s, 3H), 2.54 (s, 3H).

HRMS (EI): exact mass calculated for $[M\text{-}OCH_3]^+$ ($C_8H_7O^{\bullet+}$) requires *m/z* = 119.0497, found *m/z* = 119.0486.

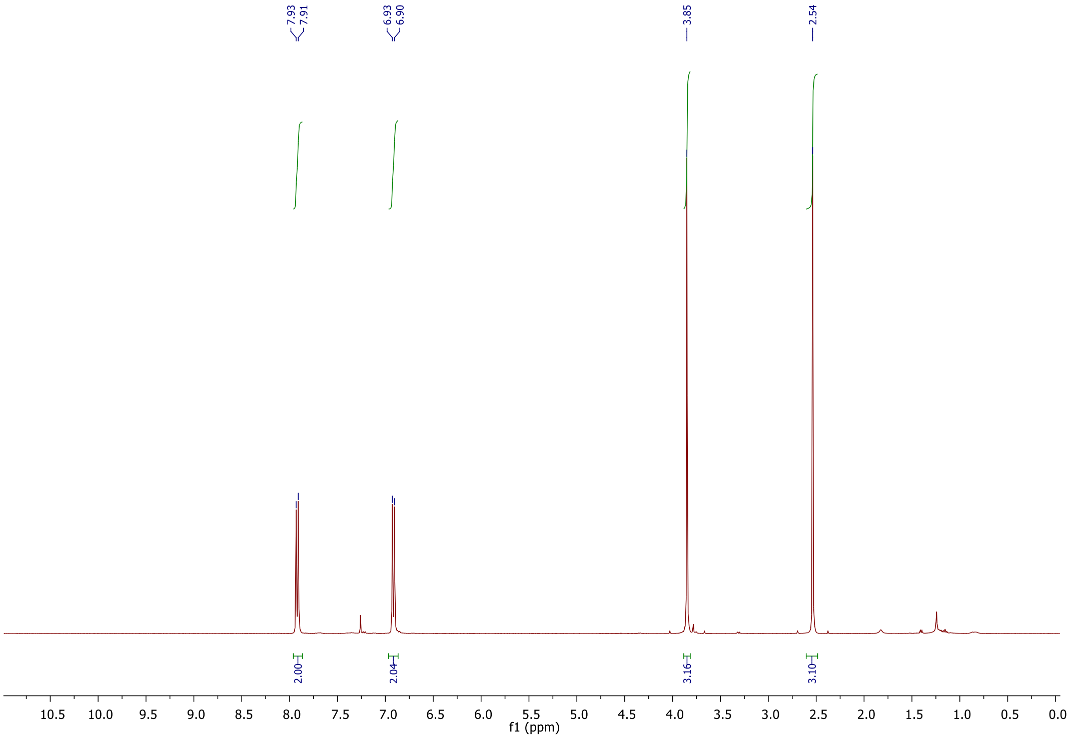

Figure S43. ^{1}H NMR (400 MHz, $CDCl_3$) of **10d**.

<u>10e</u>

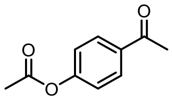

Synthesized according to the general procedure from 81.1 mg of 4-acetoxystyrene. The product was purified by flash chromatography using heptane/EtOAc as eluent to yield a white solid (75.0 mg, 0.42 mmol, Y = 84%). The analytical data was in good accordance with the literature.[63]

^{1}H NMR (400 MHz, $CDCl_3$) δ 8.00 (d, *J* = 8.7 Hz, 1H), 7.20 (d, *J* = 8.7 Hz, 1H), 2.60 (s, 2H), 2.33 (s, 2H).

HRMS (EI): exact mass calculated for $[M\text{-}COCH_3]^+$ ($C_8H_8O_2^{\bullet+}$) requires *m/z* = 136.0524, found *m/z* = 136.0524.

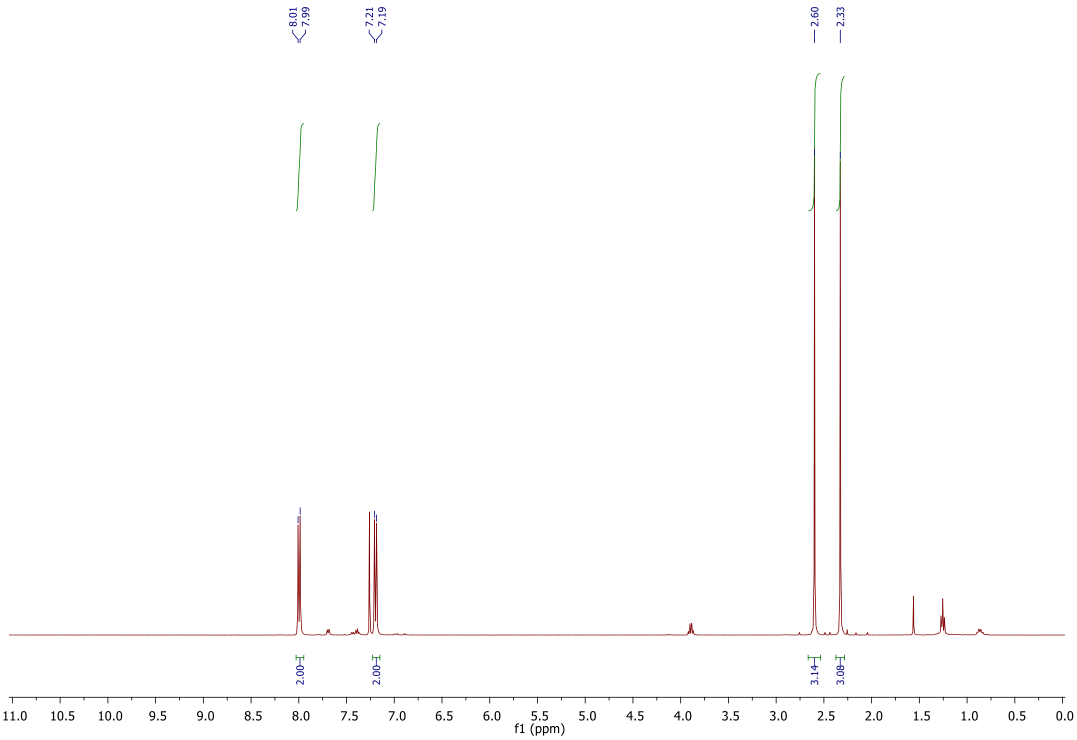

Figure S44. ^{1}H NMR (400 MHz, $CDCl_3$) of **10e**.

10f

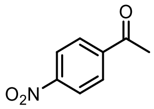

Synthesized according to the general procedure using 74.6 mg of 4-nitrostyrene. The product was purified by flash chromatography using heptane/EtOAc to yield a light solid (74.5 mg, 0.45 mmol, Y = 90%). The analytical data was in good accordance with the literature.[63]

^{1}H NMR (400 MHz, $CDCl_3$) δ 8.32 (d, J = 8.7 Hz, 2H), 8.11 (d, J = 8.7 Hz, 2H), 2.68 (s, 3H).

HRMS (EI): exact mass calculated for $[M–CH_3]^+$ ($C_7H_4NO_3^{\bullet+}$) requires m/z = 150.0191, found m/z = 150.0181.

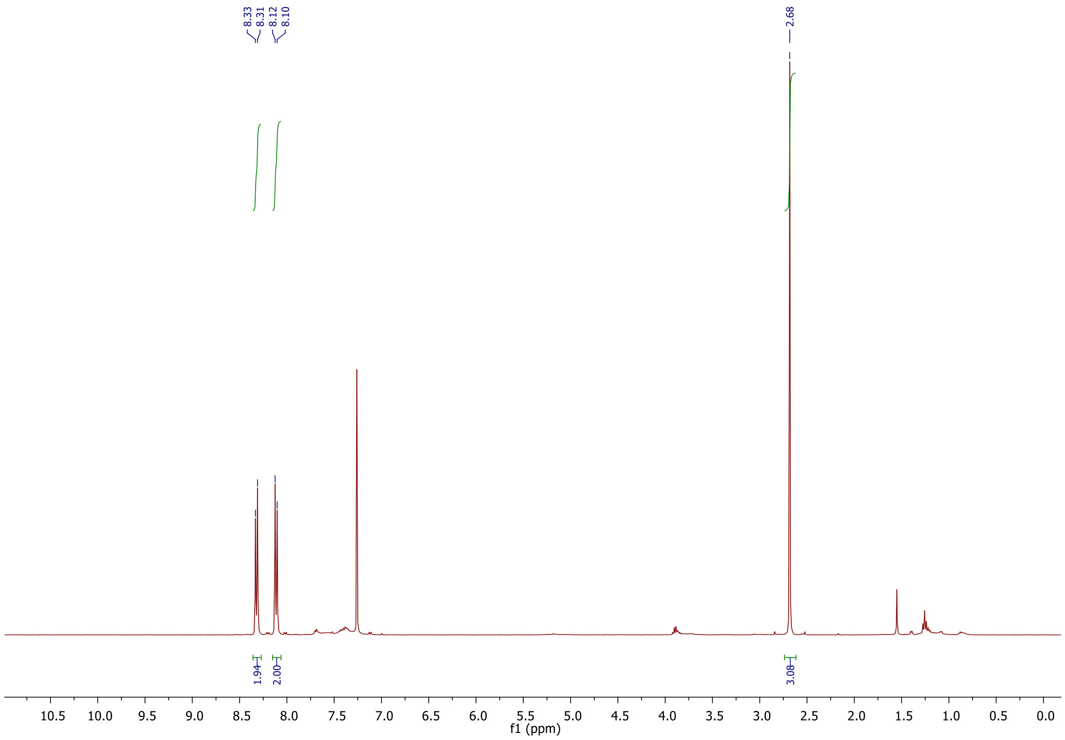

Figure S45. ^{1}H NMR (400 MHz, $CDCl_3$) of **10f**.

10g

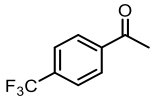

Synthesized according to the general procedure from 86.1 mg of 4-trifluoromethylstyrene. The product was purified by flash chromatography using heptane/EtOAc as eluent to yield a colorless oil (50.3 mg, 0.27 mmol, Y = 54%). The analytical data was in good accordance with the literature.[64]

^{1}H NMR (400 MHz, $CDCl_3$) δ 8.06 (d, *J* = 8.2 Hz, 2H), 7.74 (d, *J* = 8.3 Hz, 2H), 2.65 (s, 3H).

HRMS (EI): exact mass calculated for $[M-CH_3]^+$ ($C_8H_4F_3O^{\bullet+}$) requires *m/z* = 173.0214, found *m/z* = 173.0209.

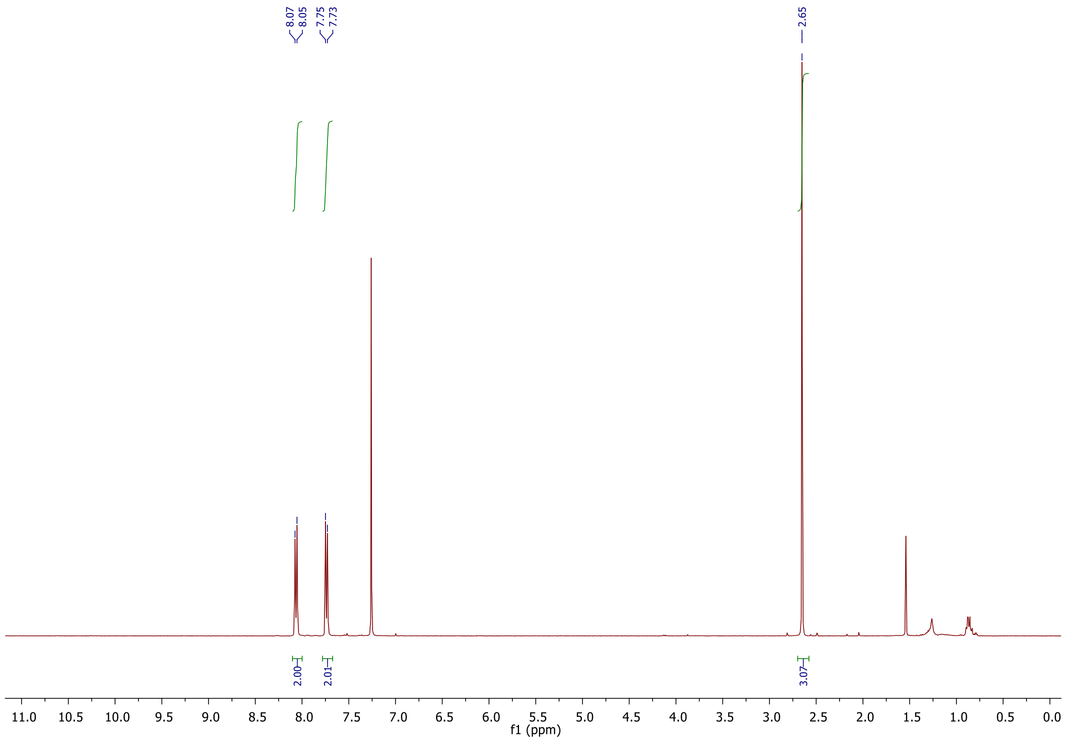

Figure S46. ^{1}H NMR (400 MHz, $CDCl_3$) of **10g**.

10h

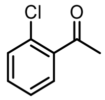

Synthesized according to the general procedure from 69 mg of 2-chlorostyrene. The product was purified by flash chromatography using heptane/EtOAc as eluent to yield a colorless oil (61 mg, 0.40 mmol, Y = 79%). The analytical data was in good accordance with literature.[64]

^{1}H NMR (400 MHz, $CDCl_3$) δ 7.55 (dd, *J* = 7.6, 1.5 Hz, 1H), 7.44 – 7.36 (m, 2H), 7.33 (m, 1H), 2.65 (s, 3H).

HRMS (EI): exact mass calculated for $[M\text{-}CH_3]^+$ ($C_7H_4ClO^{\bullet+}$) requires *m/z* = 138.9951, found *m/z* = 138.9952.

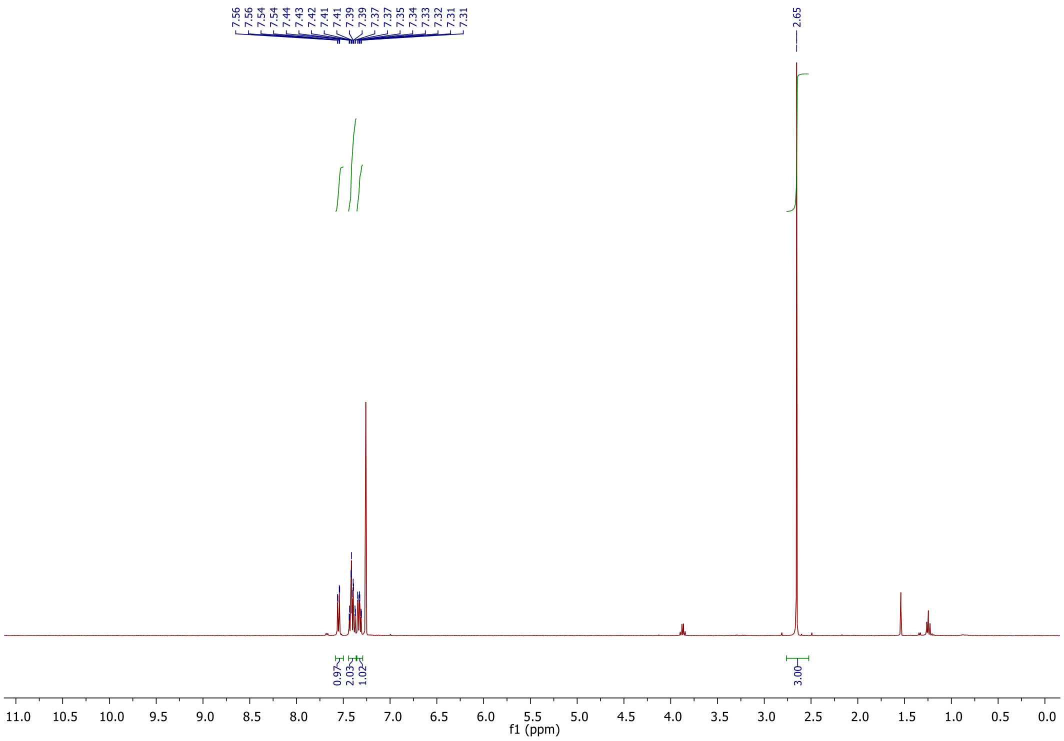

Figure S47. ^{1}H NMR (400 MHz, $CDCl_3$) of **10h**.

10i

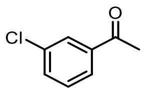

Synthesized according to the general procedure from 69 mg of 3-chlorostyrene. The product was purified by flash chromatography using heptane/EtOAc as eluent to yield a colorless oil (69 mg, 0.45 mmol, Y = 89%). The analytical data was in good accordance with literature.[64]

^{1}H NMR (400 MHz, CDCl$_3$) δ 7.93 (t, *J* = 1.8 Hz, 1H), 7.83 (m, 1H), 7.54 (m, 1H), 7.41 (t, *J* = 7.8 Hz, 1H), 2.60 (s, 3H).

HRMS (EI): exact mass calculated for $[M-CH_3]^+$ ($C_7H_4ClO^{\bullet+}$) requires *m/z* = 138.9951, found *m/z* = 138.9943.

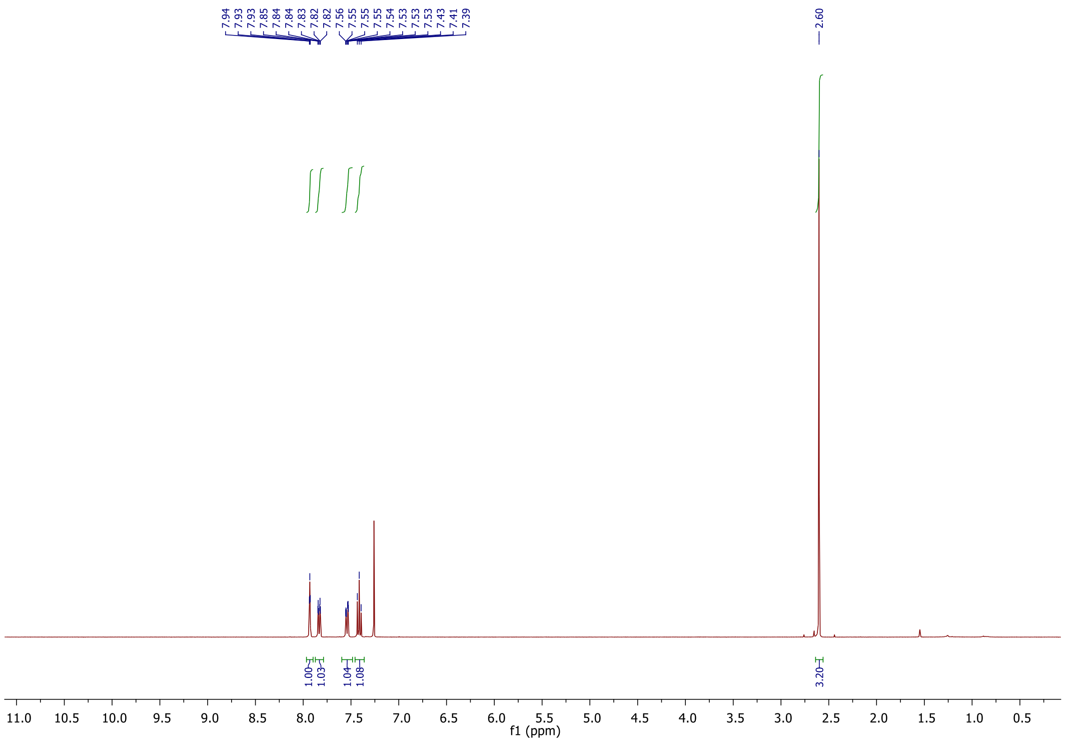

Figure S48. ^{1}H NMR (400 MHz, CDCl$_3$) of **10i**.

10j

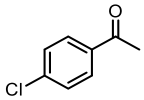

Synthesized according to the general procedure from 71.4 mg of 4-chlorostyrene. The product was purified by flash chromatography using heptane/EtOAc as eluent to yield a colorless oil (56 mg, 0.36 mmol, Y = 72%). The analytical data was in good accordance with literature.[65]

^{1}H NMR (400 MHz, $CDCl_3$) δ 7.93 – 7.84 (m, 2H), 7.47 – 7.39 (m, 2H), 2.59 (s, 3H).

HRMS (EI): exact mass calculated for $[M\text{-}CH_3]^+$ ($C_7H_4ClO^{\bullet+}$) requires *m/z* = 138.9951, found *m/z* = 138.9942.

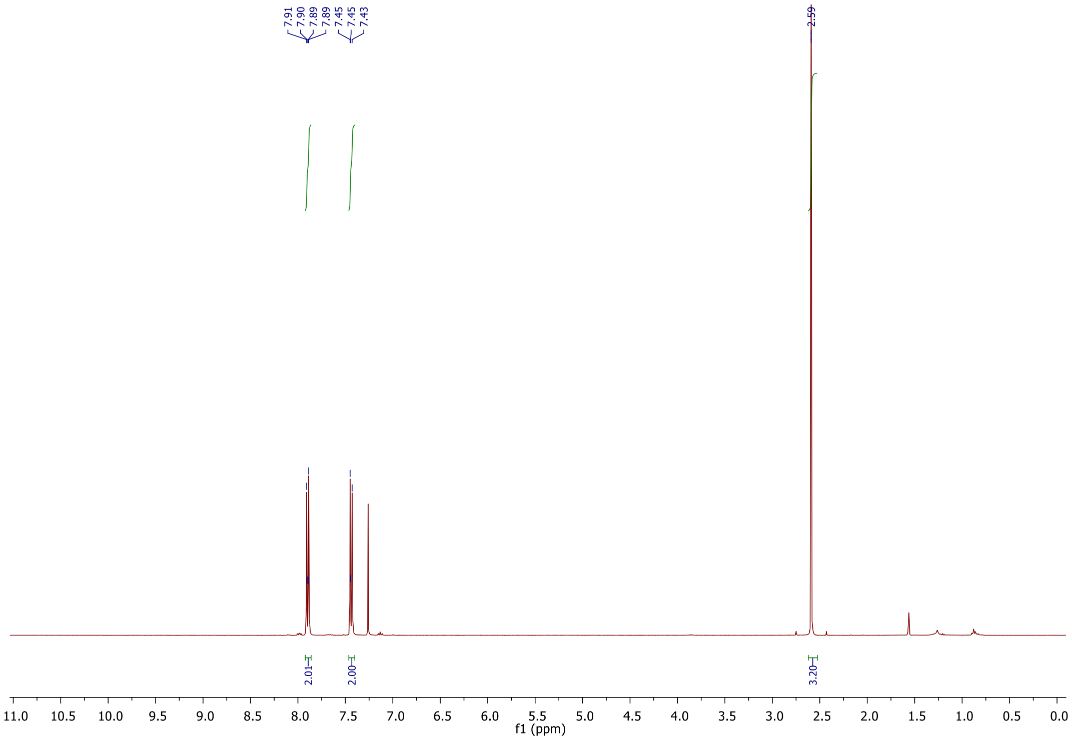

Figure S49. ^{1}H NMR (400 MHz, $CDCl_3$) of **10j**.

<u>10k</u>

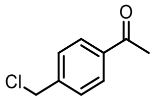

Synthesized according to the general procedure from 84.8 mg of 4-chloromethylstyrene (90% pure). The product was purified by flash chromatography using heptane/EtOAc as eluent to yield a colorless oil (50.1 mg, 0.30 mmol, Y = 59%). The analytical data was in good accordance with the literature.[66]

^{1}H NMR (400 MHz, $CDCl_3$) δ 7.96 (d, *J* = 8.3 Hz, 2H), 7.49 (d, *J* = 8.2 Hz, 2H), 4.62 (s, 3H), 2.61 (s, 3H).

HRMS (EI): exact mass calculated for $[M\text{-}COCH_3]^+$ ($C_7H_6Cl^{\bullet+}$) requires *m/z* = 125.0158, found *m/z* = 125.0165.

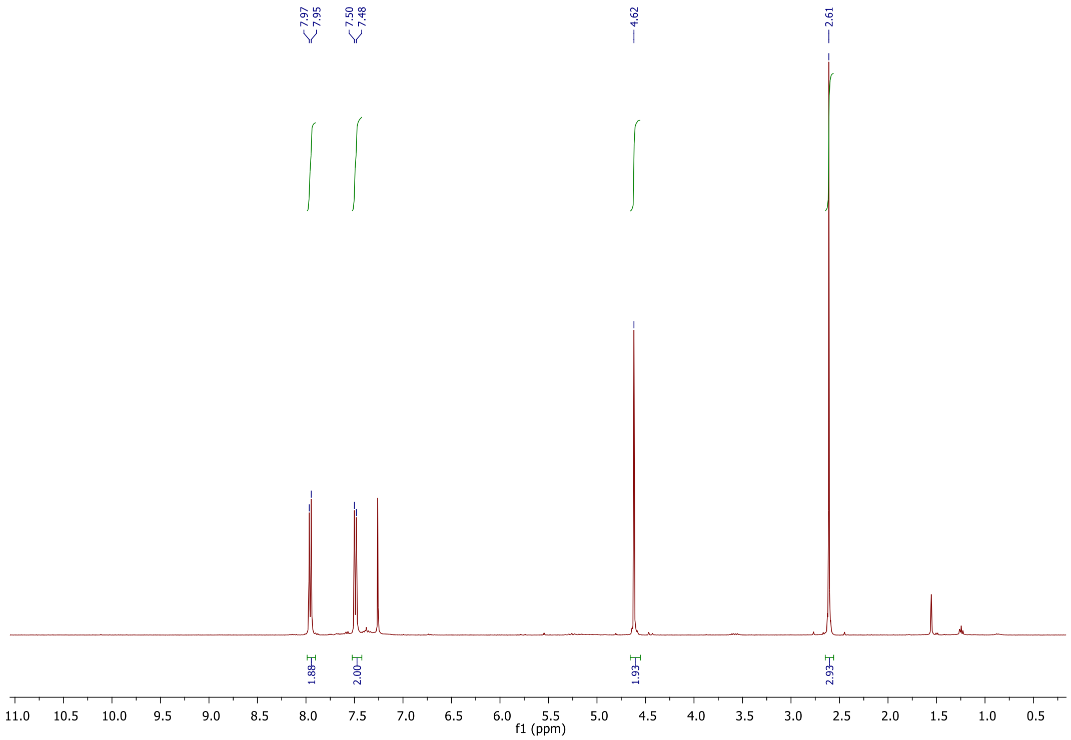

Figure S50. ^{1}H NMR (400 MHz, $CDCl_3$) of **10k**.

10l

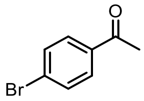

Synthesized according to the general procedure from 91.5 mg of 4-bromostyrene. The product was purified by flash chromatography using heptane/EtOAc as eluent to yield a white solid (71.3 mg, 0.36 mmol, Y = 72%). The analytical data was in good accordance with the literature.[63]

^{1}H NMR (400 MHz, $CDCl_3$) δ 7.82 (d, J = 8.5 Hz, 2H), 7.61 (d, J = 8.5 Hz, 2H), 2.59 (s, 3H).

HRMS (EI): exact mass calculated for $[M-CH_3]^+$ ($C_7H_4BrO^{\bullet+}$) requires m/z = 182.9446, found m/z = 182.9441.

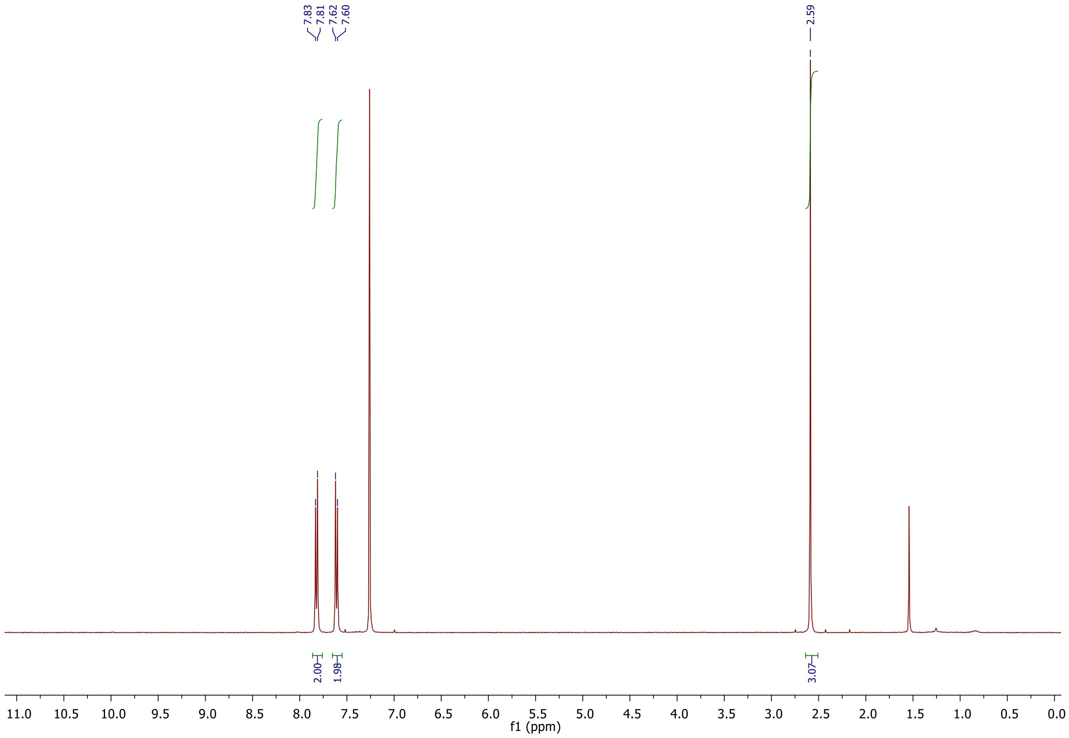

Figure S51. ^{1}H NMR (400 MHz, $CDCl_3$) of **10l**.

10m

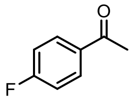

Synthesized according to the general procedure from 61.1 mg of 4-fluorostyrene. The product was purified by flash chromatography using heptane/EtOAc as eluent to yield a colorless oil (54 mg, 0.39 mmol, Y = 78%). The analytical data was in good accordance with literature.[67]

^{1}H NMR (400 MHz, $CDCl_3$) δ 7.98 (m, 2H), 7.13 (m, 2H), 2.59 (s, 3H).

HRMS (EI): exact mass calculated for $[M\text{-}CH_3]^+$ ($C_7H_4FO^{\bullet+}$) requires *m/z* = 123.0246, found *m/z* = 123.0246.

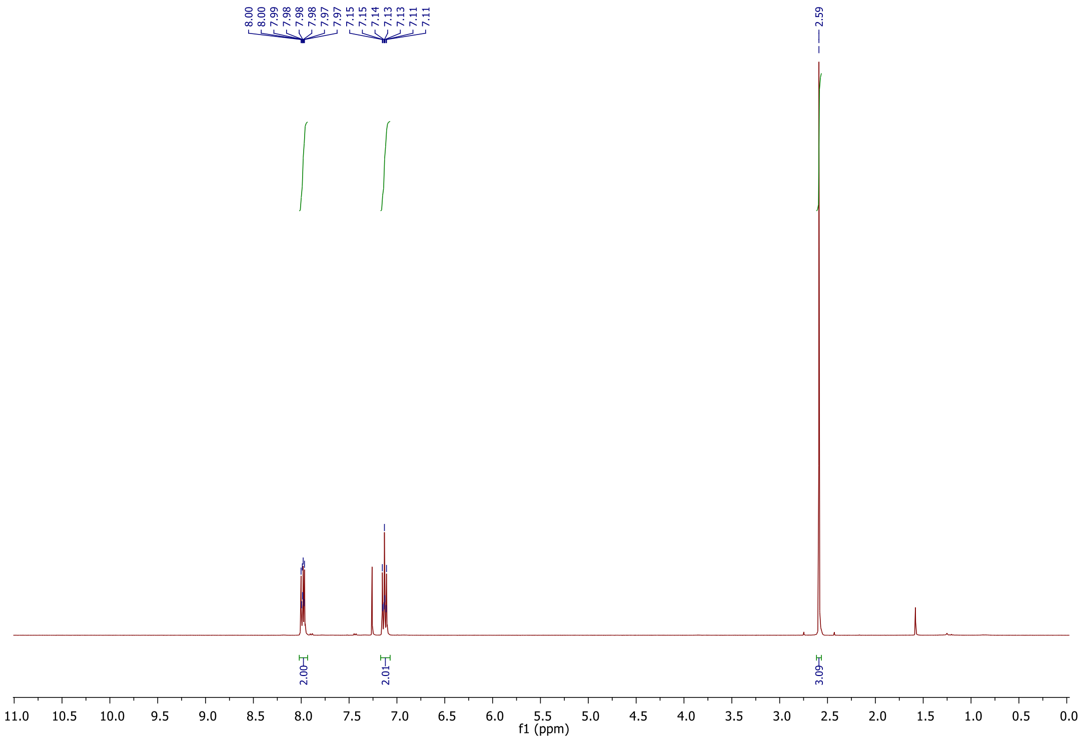

Figure S52. ^{1}H NMR (400 MHz, $CDCl_3$) of **10m**.

10n

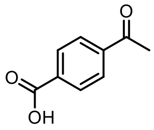

Synthesized according to the general procedure from 76.4 mg of 4-vinylbenzoic acid (97% purity). The product was purified by flash chromatography using heptane/EtOAc/EtOH/AcOH as eluent to yield a white solid (49 mg, 0.30 mmol, Y = 60%). The analytical data was in good accordance with literature.[68]

^{1}H NMR (400 MHz, DMSO) δ 8.05 (s, 4H), 2.62 (s, 3H).

HRMS (EI): exact mass calculated for $[M\text{-}CH_3]^+$ ($C_8H_5O_3^{\bullet+}$) requires *m/z* = 149.0239, found *m/z* = 143.0232.

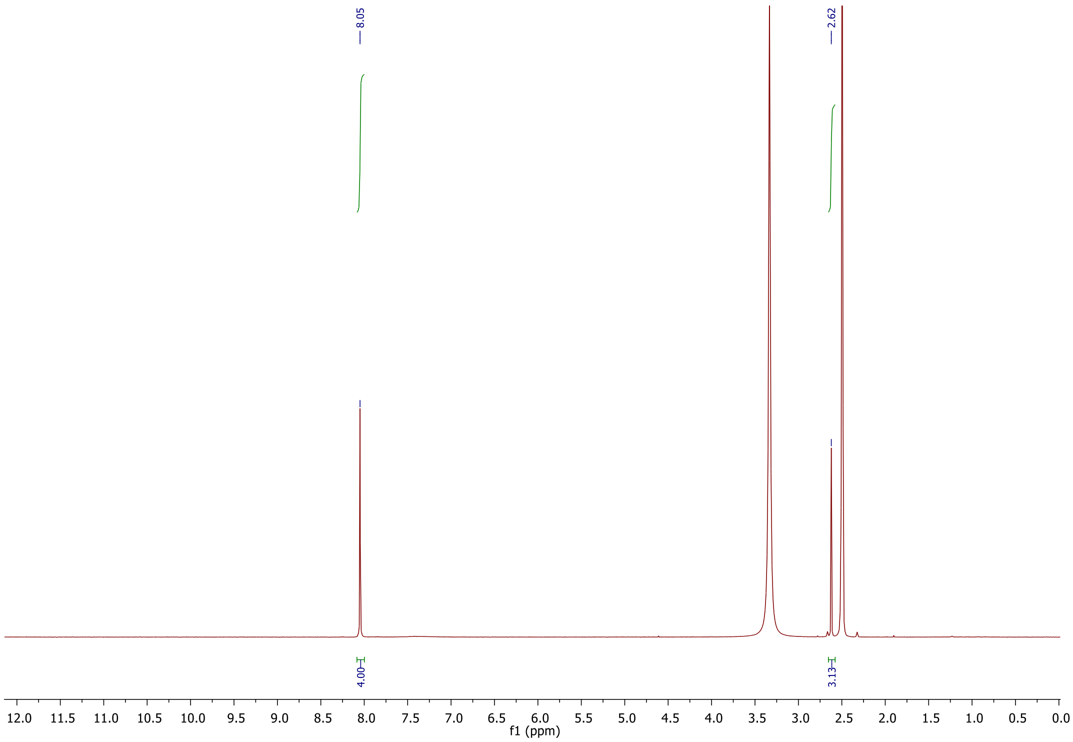

Figure S53. ^{1}H NMR (400 MHz, $CDCl_3$) of **10n**.

10o

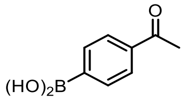

Synthesized according to the general procedure from 74.0 mg of 4-vinylphenylboronic acid. The product was purified by flash chromatography using heptane/EtOAc/EtOH as eluent to give white solid (66 mg, 0.40 mmol, Y = 81%). The analytical data was in good accordance with literature.[66]

^{1}H NMR (400 MHz, $(CD_3)_2CO$) δ 8.06 – 7.96 (m, 4H), 7.42 (s, 2H), 2.63 (s, 3H).

HRMS (EI): exact mass calculated for $[M–BO_2H–CH_3]^+$ ($C_7H_5O^{•+}$) requires *m/z* = 105.0340, found *m/z* = 105.0330.

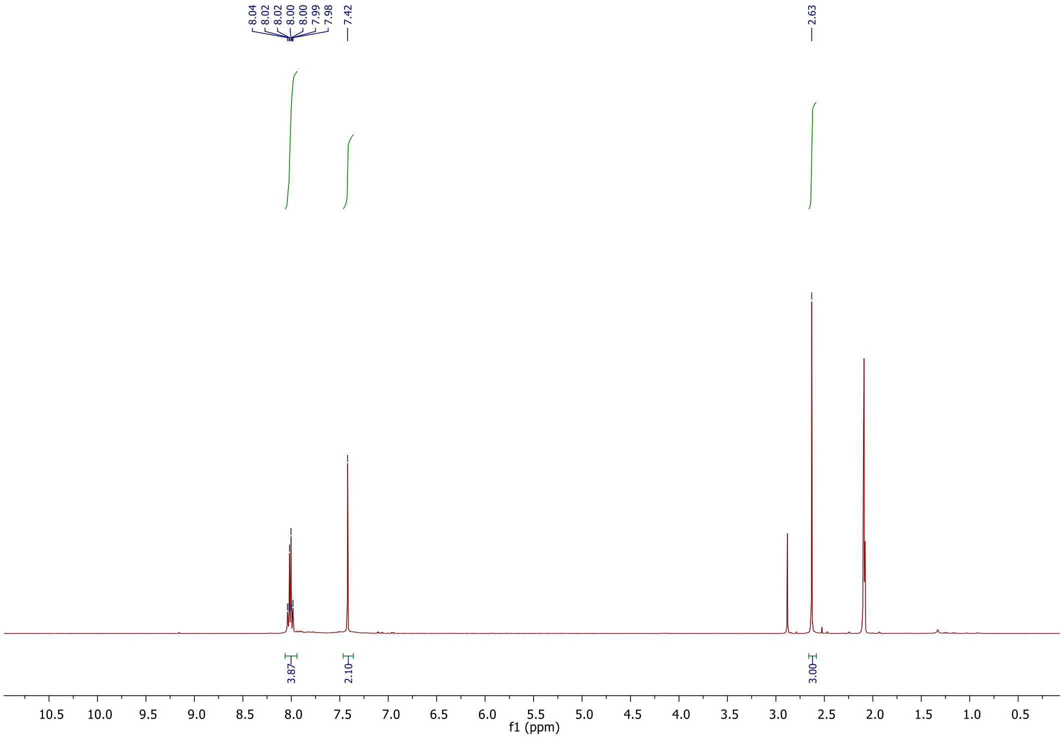

Figure S54. ^{1}H NMR (400 MHz, $CDCl_3$) of **10o**.

10p

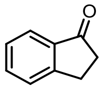

Synthesized according to the general procedure from 62.5 mg of indene (93% purity). The product was purified by flash chromatography using heptane/EtOAc as eluent to yield a yellow oil (50 mg, 0.38 mmol, Y = 76%). The analytical data was in good accordance with literature.[60]

^{1}H NMR (400 MHz, $CDCl_3$) δ 7.77 (d, *J* = 7.7 Hz, 1H), 7.59 (t, *J* = 7.4 Hz, 1H), 7.48 (d, *J* = 7.7 Hz, 1H), 7.37 (t, *J* = 7.4 Hz, 1H), 3.15 (m, 2H), 2.70 (m, 2H).

HRMS (EI): exact mass calculated for $[M]^+$, ($C_9H_8O^{\bullet+}$) requires *m/z* = 132.0575, found *m/z* = 132.0563.

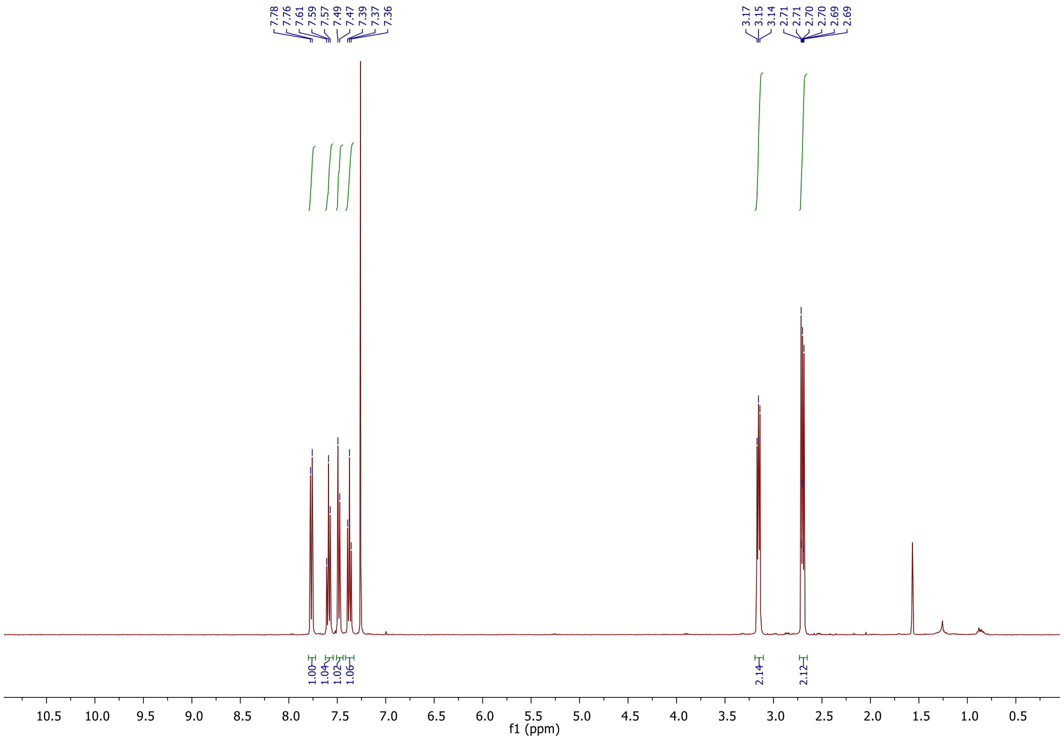

Figure S55. ^{1}H NMR (400 MHz, $CDCl_3$) of **10p**.

10q

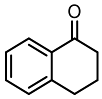

Synthesized according to the general procedure using 65 mg of **9q**. The product was purified by flash chromatography using heptane/EtOAc to yield a colorless oil (39 mg, 0.27 mmol, Y = 53%). The analytical data was in good in accordance with the literature.[69]

^{1}H NMR (400 MHz, $CDCl_3$) δ 8.04 (dd, *J* = 7.8, 1.1 Hz, 2H), 7.47 (td, *J* = 7.5, 1.4 Hz, 1H), 7.31 (t, *J* = 7.5 Hz, 1H), 7.26 (m, 1H)*, 2.97 (t, *J* = 6.1 Hz, 2H), 2.66 (m, t, *J* = 6.7 Hz, 2H), 2.15 (m, 2H). * under the solvent peak.

HRMS (EI): exact mass calculated for $[M]^+$ ($C_{10}H_{10}O^{\bullet+}$) requires *m/z* = 146.0732, found *m/z* = 146.0721.

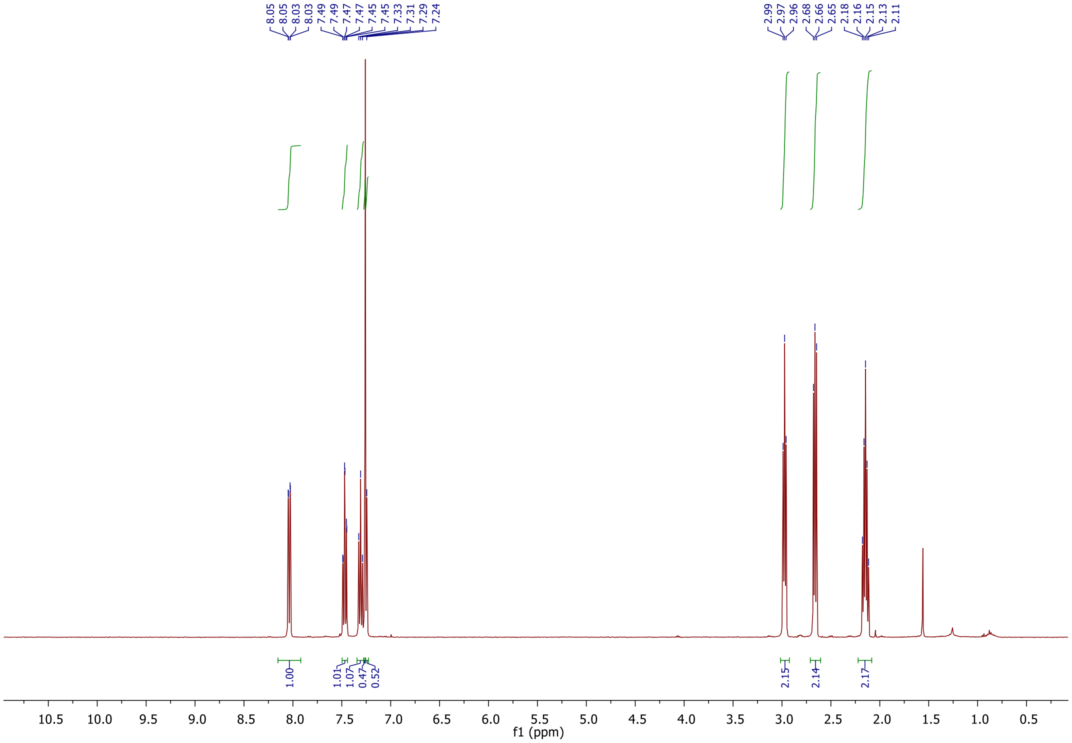

Figure S56. ^{1}H NMR (400 MHz, $CDCl_3$) of **10q**.

10r

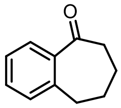

Synthesized according to the general procedure from 76 mg of **9r**. The product was purified by flash chromatography using heptane/EtOAc as eluent to yield a colorless oil (35 mg, 0.22 mmol, Y = 44%). The analytical data was in accordance with the literature.[70]

^{1}H NMR (400 MHz, $CDCl_3$) δ 7.72 (dd, *J* = 7.7, 1.3 Hz, 1H), 7.42 (td, *J* = 7.5, 1.4 Hz, 1H), 7.30 (td, *J* = 7.6, 1.0 Hz, 1H), 7.20 (d, *J* = 7.5 Hz, 1H), 2.93 (m, 2H), 2.73 (dd, *J* = 6.9, 5.1 Hz, 2H), 1.85 (m, 4H).

HRMS (EI): exact mass calculated for $[M]^+$ ($C_{11}H_{12}O^{•+}$) requires *m/z* = 160.0888, found *m/z* = 160.0881.

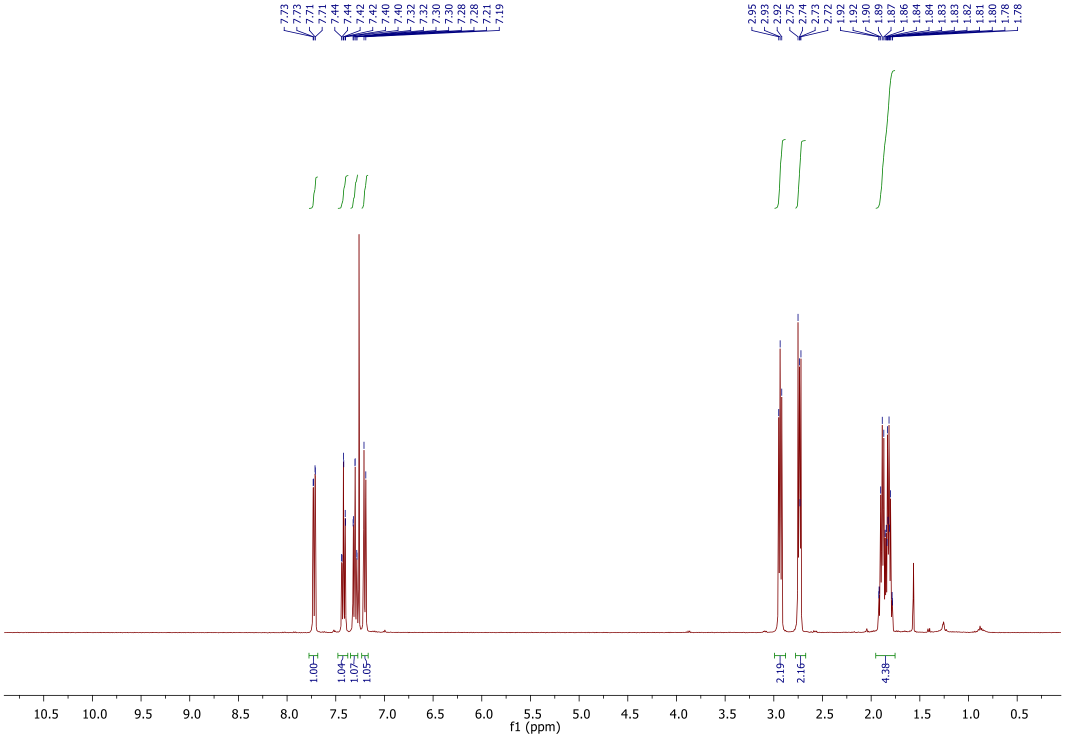

Figure S57. ^{1}H NMR (400 MHz, $CDCl_3$) of **10r**.

10s

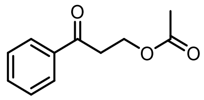

Synthesized according to the general procedure from 88 mg of cinnamylacetate. The product was purified by flash chromatography using heptane/EtOAc as eluent to yield a light-yellow waxy oil (75 mg, 0.39 mmol, Y = 78%). The analytical data was in good accordance with literature.[63]

^{1}H NMR (400 MHz, $CDCl_3$) δ 7.97 (d, *J* = 7.8 Hz, 2H), 7.59 (t, *J* = 7.5 Hz, 1H), 7.48 (t, *J* = 7.6 Hz, 2H), 4.53 (t, *J* = 6.3 Hz, 2H), 3.32 (t, *J* = 6.4 Hz, 2H), 2.04 (s, 3H).

HRMS (EI): exact mass calculated for $[M\text{-}CH_2CH_2OCOCH_3]^+$ ($C_7H_5O^{\bullet+}$) requires *m/z* = 105.0340, found *m/z* = 105.0331.

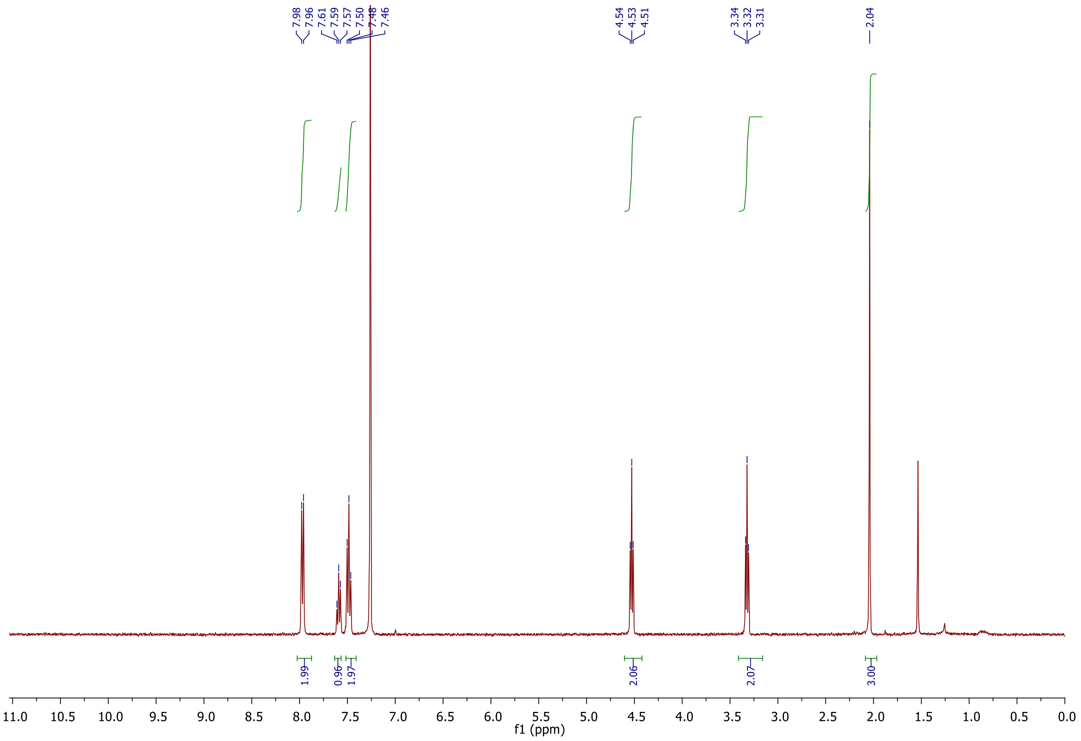

Figure S58. ^{1}H NMR (400 MHz, $CDCl_3$) of **10s**.

10t

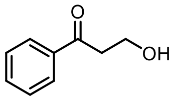

Synthesized according to the general procedure from 67 mg of cinnamylalcohol. The product was purified by flash chromatography using heptane/EtOAc as eluent to yield a light-yellow oil (44 mg, 0.29 mmol, Y = 59%). The analytical data was in good accordance with literature.[71]

^{1}H NMR (400 MHz, CDCl$_3$) δ 7.97 (d, *J* = 8.0 Hz, 2H), 7.59 (m, 1H), 7.49 (t, *J* = 7.6 Hz, 2H), 4.04 (q, *J* = 5.6 Hz, 2H), 3.24 (t, *J* = 5.3 Hz, 2H), 2.63 (t, *J* = 6.6 Hz, 1H).

HRMS (EI): exact mass calculated for $[M-CH_2CH_2OH]^+$ ($C_7H_5O^{\bullet+}$) requires *m/z* = 105.0340, found *m/z* = 105.0332.

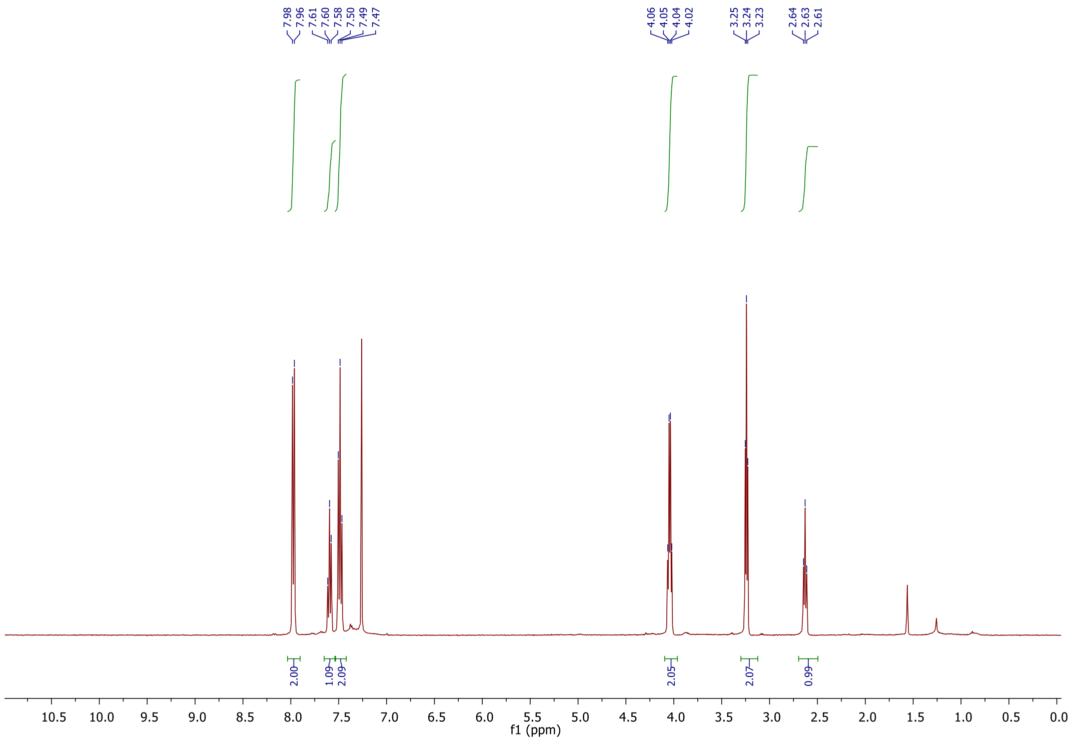

Figure S59. ^{1}H NMR (400 MHz, CDCl$_3$) of **10t**.

<u>10u</u>

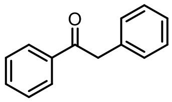

Synthesized according to the general procedure from 90 mg of *cis*-stillbene. The product was purified by flash chromatography using heptane/EtOAc as eluent to yield a white solid (49 mg, 0.25 mmol, Y = 50%). The analytical data was in good accordance with literature.[72]

^{1}H NMR (400 MHz, $CDCl_3$) δ 7.93 (m, 2H), 7.51 – 7.44 (m, 1H), 7.41 – 7.33 (m, 2H), 7.33 – 7.22 (m, 2H), 7.21 – 7.13 (m, 3H), 4.20 (s, 2H).

HRMS (ESI$^+$): exact mass calculated for $[M+Na]^+$ $(C_{14}H_{12}ONa)^+$ requires *m/z* = 219.0780, found *m/z* = 219.0784.

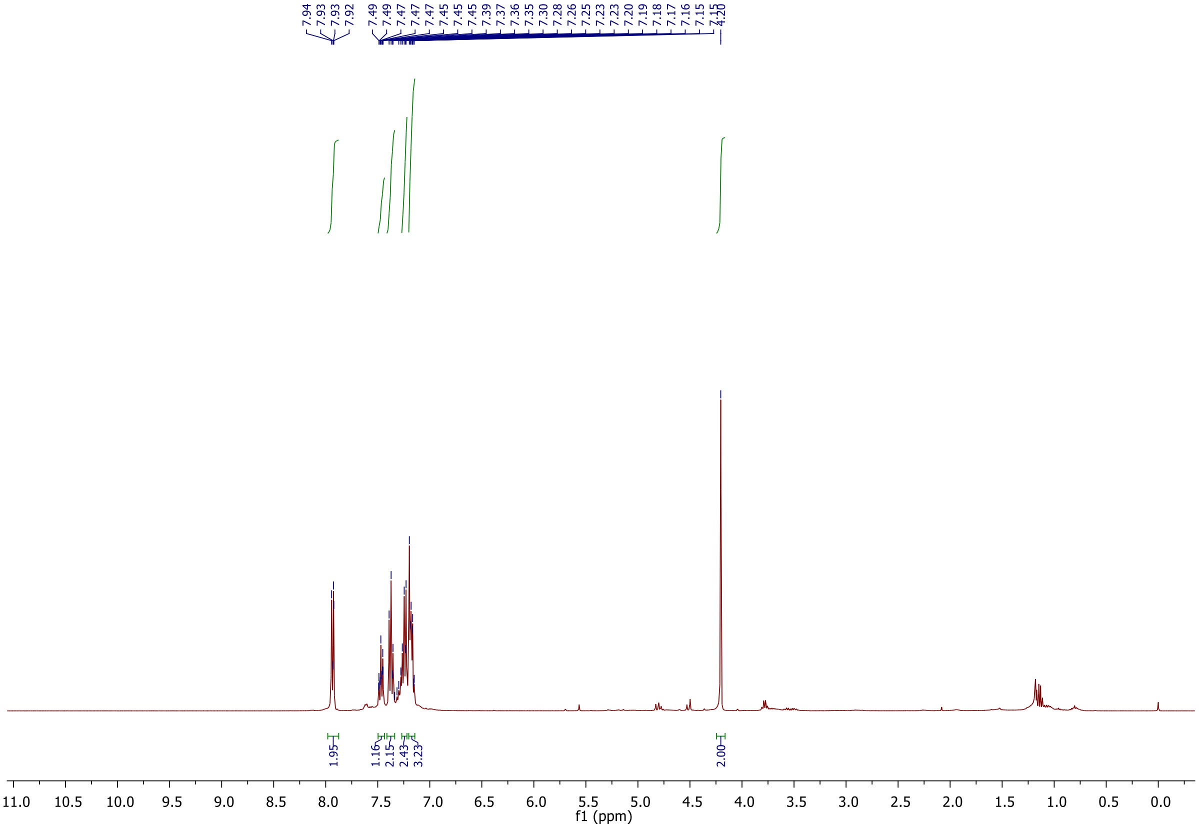

Figure S60. ^{1}H NMR (400 MHz, $CDCl_3$) of **10u**.

10v

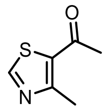

Synthesized according to the general procedure using 65.2 mg of 4-methyl-5-vinylthiazol (96% purity). The product was purified by flash chromatography using heptane/EtOAc as eluent to yield a light-yellow oil (47 mg, 0.33 mmol, Y = 67%).

^{1}H NMR (700 MHz, $CDCl_3$) δ 8.76 (s, 1H), 2.78 (s, 3H), 2.57 (s, 3H).

^{13}C NMR (176 MHz, $CDCl_3$) δ 190.7, 159.1, 154.7, 131.5, 31.0, 18.4.

HRMS (ESI+): *m/z* calculated for $[M+H]^+$ ($C_6H_8NOS^+$) = 142.0321, found *m/z* = 142.0315.

IR (neat) ν_{max}: 1671, 1656, 1501, 1357, 1311.

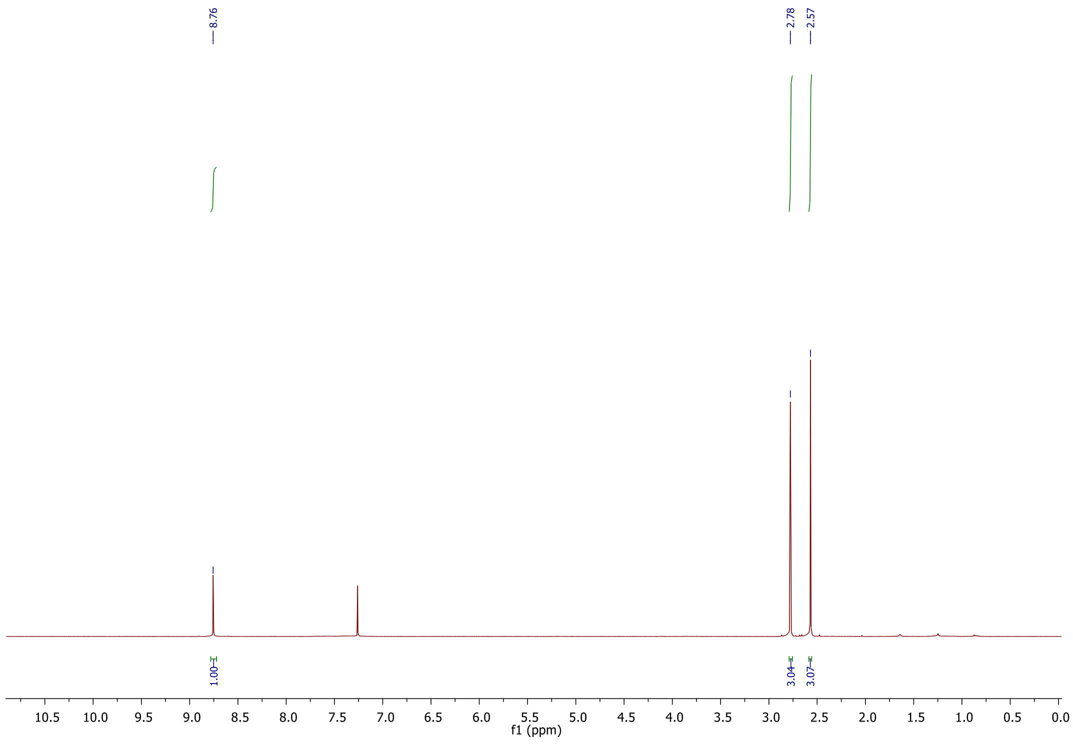

Figure S61. ^{1}H NMR (400 MHz, $CDCl_3$) of **10v**.

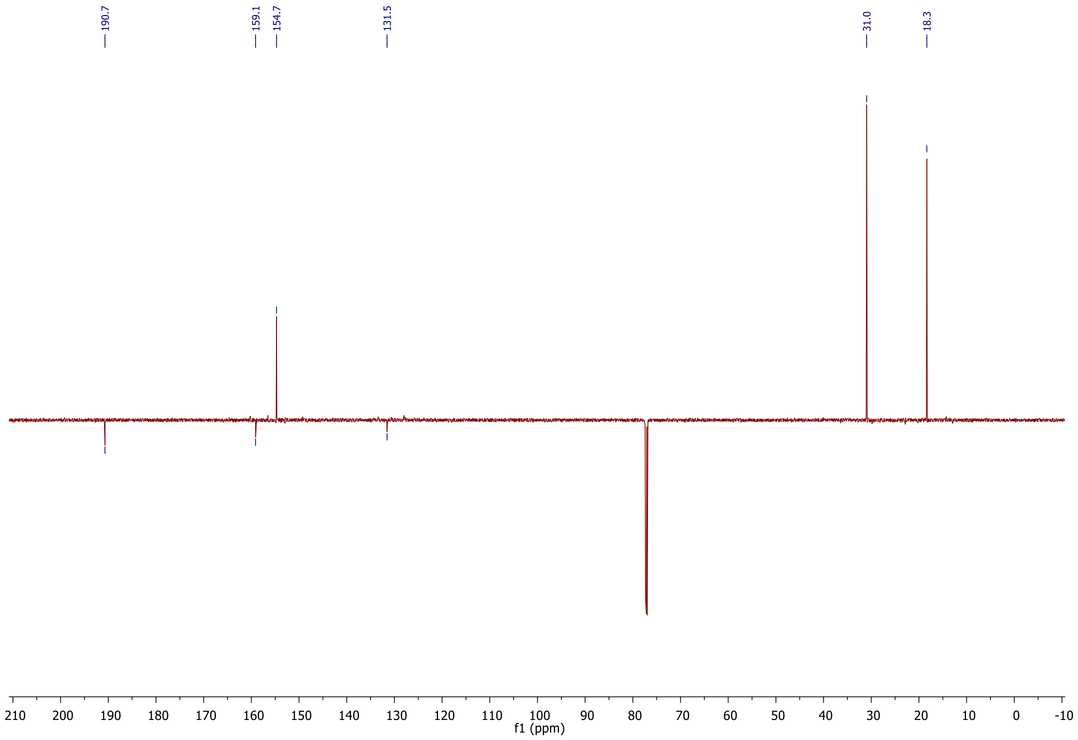

Figure S62. ^{13}C NMR (176 MHz, $CDCl_3$) of **10v**.

10w

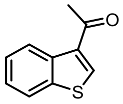

Synthesized according to the general procedure from 80 mg of **9w**. The product was purified by flash chromatography using heptane/EtOAc as eluent to yield an orange oil (55 mg, 0.31 mmol, Y = 62%). The analytical data was in good accordance with literature.[73]

^{1}H NMR (400 MHz, CDCl$_3$) δ 8.77 (d, *J* = 8.2 Hz, 1H), 8.29 (s, 1H), 7.87 (d, *J* = 8.0 Hz, 1H), 7.53 (m, 1H), 7.43 (m, 1H), 2.66 (s, 3H).

HRMS (EI): exact mass calculated for $[M]^+$ ($C_{10}H_8OS^{\bullet+}$) requires *m/z* = 176.0296, found *m/z* = 176.0285.

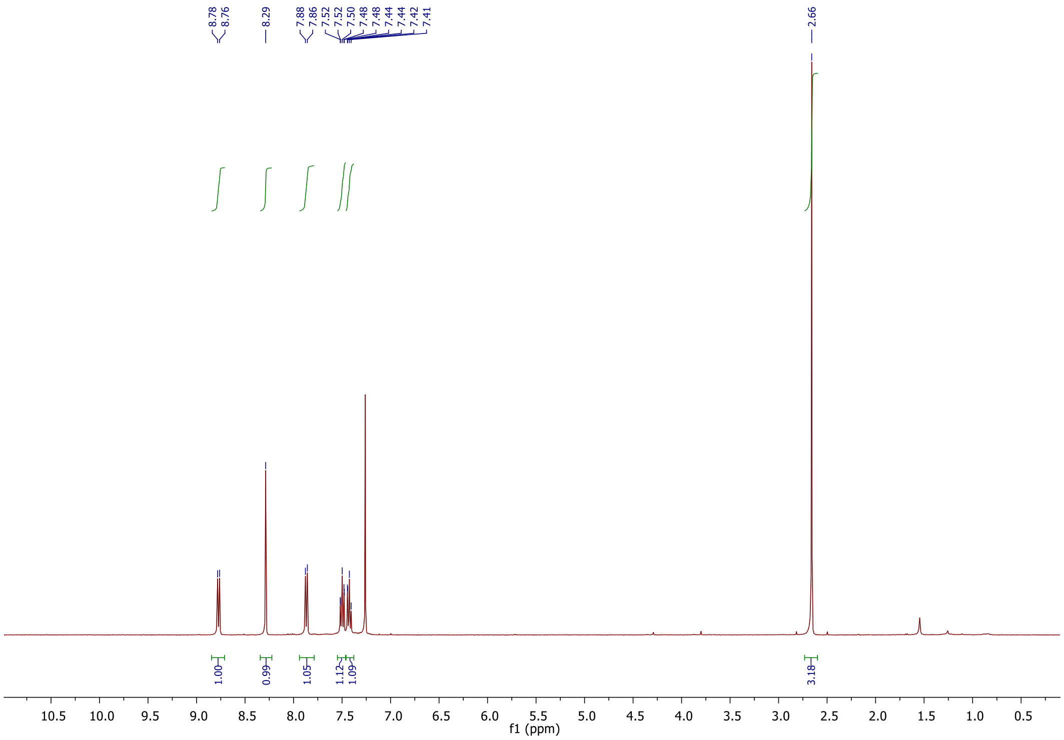

Figure S63. ^{1}H NMR (400 MHz, $CDCl_3$) of **10w**.

10x

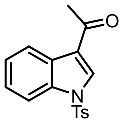

Synthesized according to the general procedure from 80 mg of **10x**. The product was purified by flash chromatography using heptane/EtOAc as eluent to yield a white solid (96 mg, 0.30 mmol, Y = 61%). The analytical data was in good accordance with literature.[74]

^{1}H NMR (400 MHz, CDCl3) δ 8.33 (d, *J* = 7.2 Hz, 1H), 8.20 (s, 1H), 7.92 (d, *J* = 7.6 Hz, 1H), 7.84 (d, *J* = 8.0 Hz, 2H), 7.41 – 7.30 (m, 3H), 7.29 (d, *J* = 8.2 Hz, 3H), 2.57 (s, 3H), 2.38 (s, 3H).

HRMS (ESI$^+$): exact mass calculated for $[M+Na]^+$ $(C_{17}H_{15}NO_3SNa)^+$ requires *m/z* = 336.0665, found *m/z* = 336.0659.

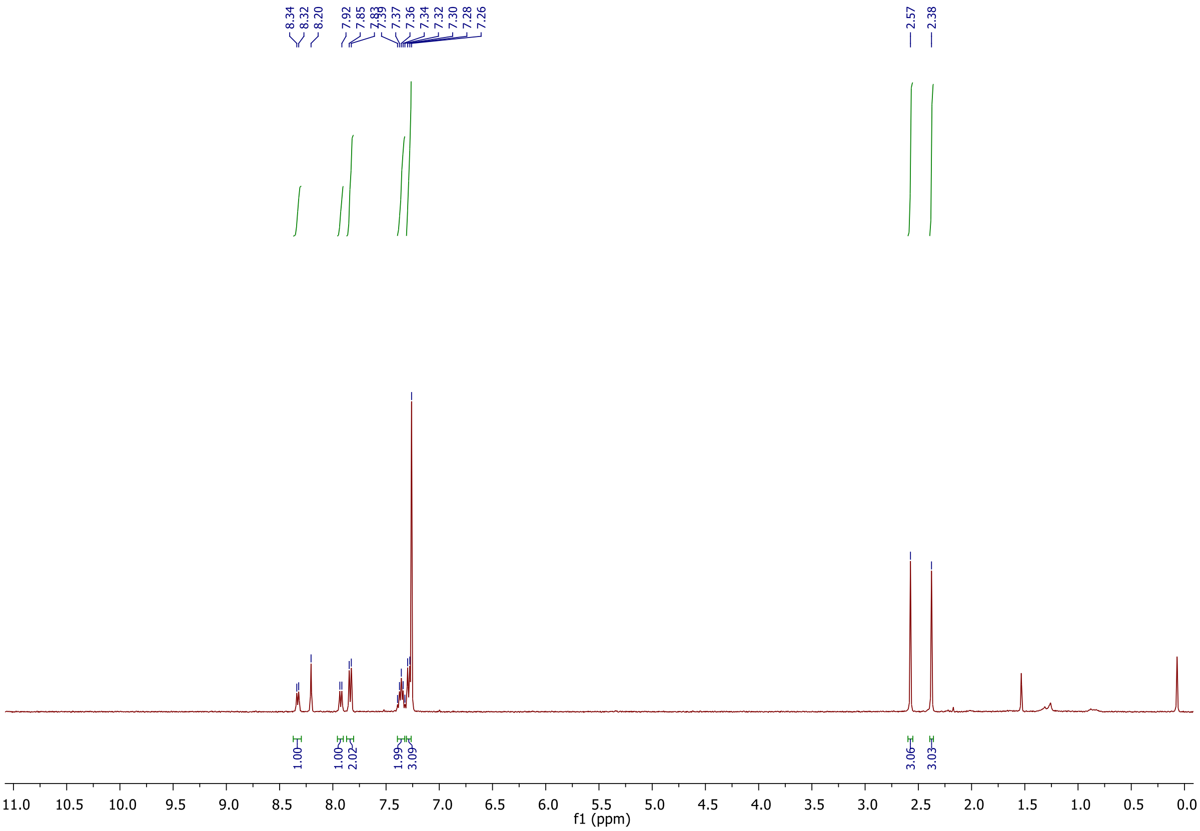

Figure S64. ^{1}H NMR (400 MHz, $CDCl_3$) of **10x**.

10y

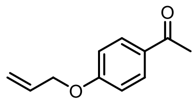

Synthesized according to the general procedure from 80.1 mg of **10y**. The product was purified by flash chromatography using heptane/EtOAc as eluent to yield a colorless oil (56.2 mg, 0.32 mmol, Y = 64%). The analytical data was in good accordance with the literature.[75]

^{1}H NMR (400 MHz, $CDCl_3$) δ 7.93 (d, *J* = 8.8 Hz, 2H), 6.95 (d, *J* = 8.8 Hz, 2H), 6.05 (m, 1H), 5.43 (dd, *J* = 17.3, 1.4 Hz, 1H), 5.32 (dd, *J* = 10.5, 1.2 Hz, 1H), 4.61 (d, *J* = 5.3 Hz, 2H), 2.56 (s, 3H).

HRMS (EI): exact mass calculated for $[M]^+$ ($C_{11}H_2O_2^{\bullet+}$) requires *m/z* = 176.0837, found *m/z* = 176.0827.

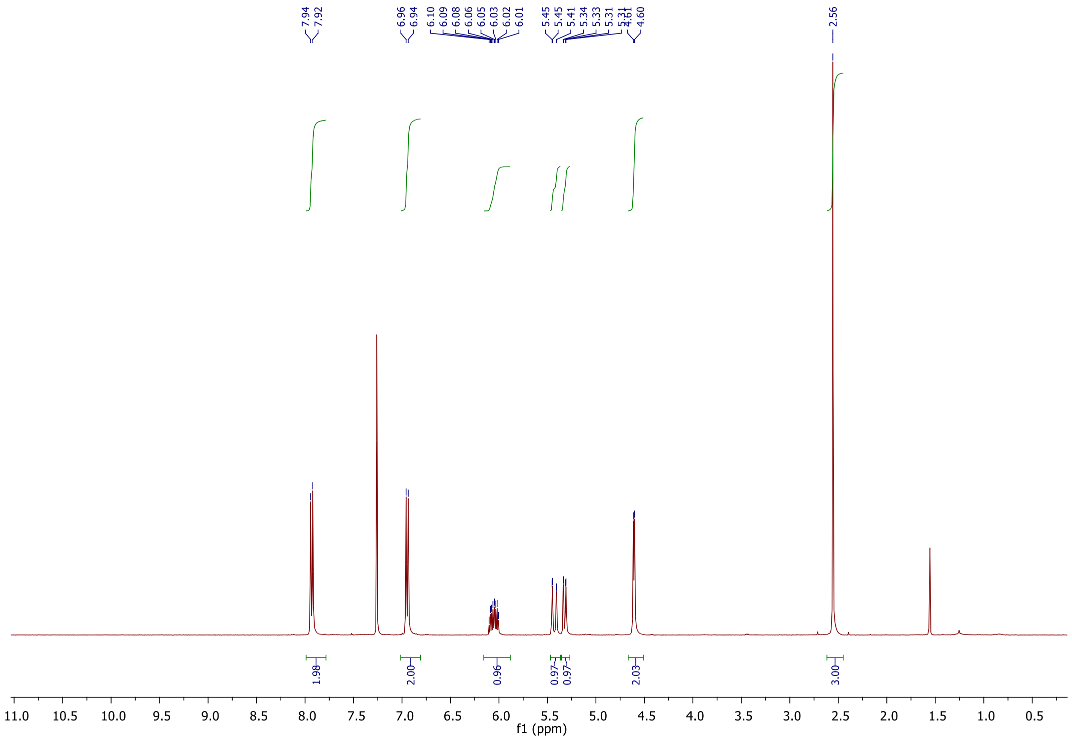

Figure S65. ^{1}H NMR (400 MHz, $CDCl_3$) of **10y**.

10z

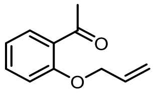

Synthesized according to the general procedure from 80.1 mg of **10z**. The crude mixture was purified by flash chromatography using heptane/EtOAc as eluent to yield a light-yellow oil (38 mg, 0.22 mmol, Y = 43%). Analytical data was in good accordance with literature.[76]

^{1}H NMR (400 MHz, $CDCl_3$) δ 7.73 (dd, *J* = 7.7, 1.8 Hz, 1H), 7.44 (ddd, *J* = 8.4, 7.4, 1.8 Hz, 1H), 7.00 (td, *J* = 7.5, 0.9 Hz, 1H), 6.95 (d, *J* = 8.4 Hz, 1H), 6.09 (ddt, *J* = 17.2, 10.6, 5.3 Hz, 1H), 5.44 (ddd, *J* = 17.3, 3.0, 1.5 Hz, 1H), 5.33 (ddd, *J* = 10.5, 2.6, 1.3 Hz, 1H), 4.65 (dt, *J* = 5.3, 1.5 Hz, 2H), 2.64 (s, 3H).

HRMS (EI): exact mass calculated for $[M-CH_3]^+$ ($C_{10}H_9O_2^{\bullet+}$) requires *m/z* = 161.0603, found *m/z* = 161.0589.

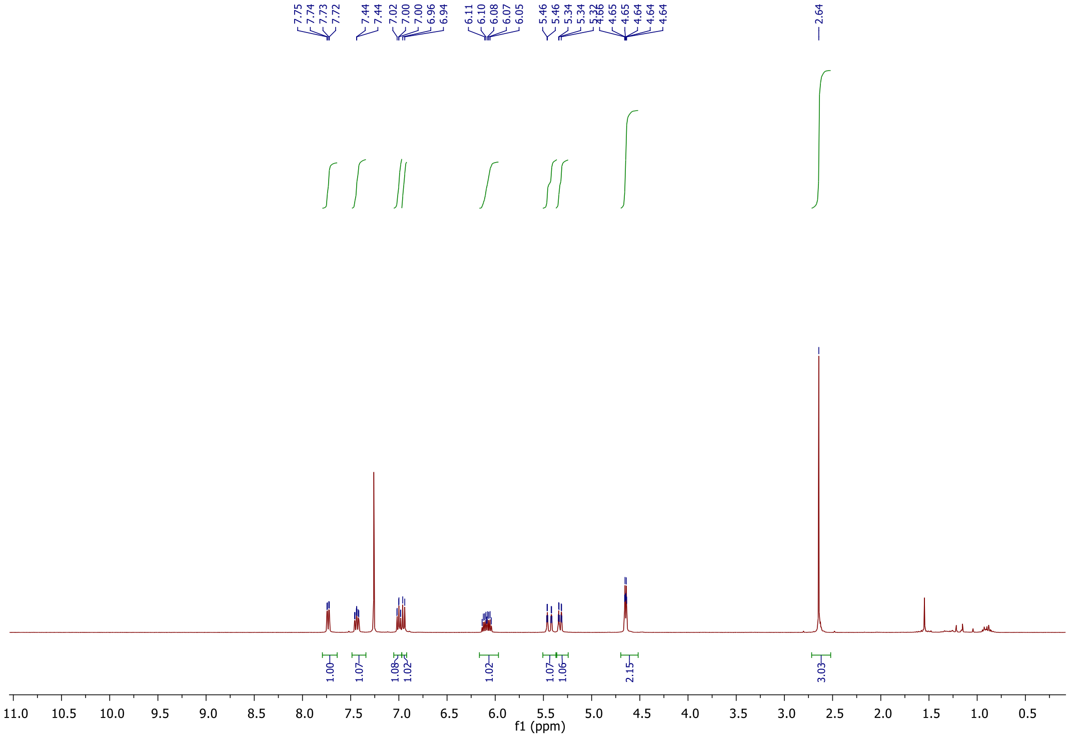

Figure S66. ^{1}H NMR (400 MHz, $CDCl_3$) of **10z**.

10aa

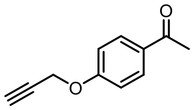

Synthesized according to the general procedure from 79.1 mg of **9aa**. The product was purified by flash chromatography using heptane/EtOAc as eluent to yield a white solid (58 mg, 0.33 mmol, Y = 67%). The analytical data was in good accordance with literature.[77]

^{1}H NMR (400 MHz, CDCl$_3$) δ 7.96 (d, *J* = 8.9 Hz, 2H), 7.02 (d, *J* = 8.9 Hz, 2H), 4.76 (d, *J* = 2.3 Hz, 2H), 2.57 (m, 4H).

HRMS (EI): exact mass calculated for $[M\text{-}CH_3]^+$ ($C_{10}H_7O_2^{\bullet+}$) requires *m/z* = 159.0446, found *m/z* = 159.0437.

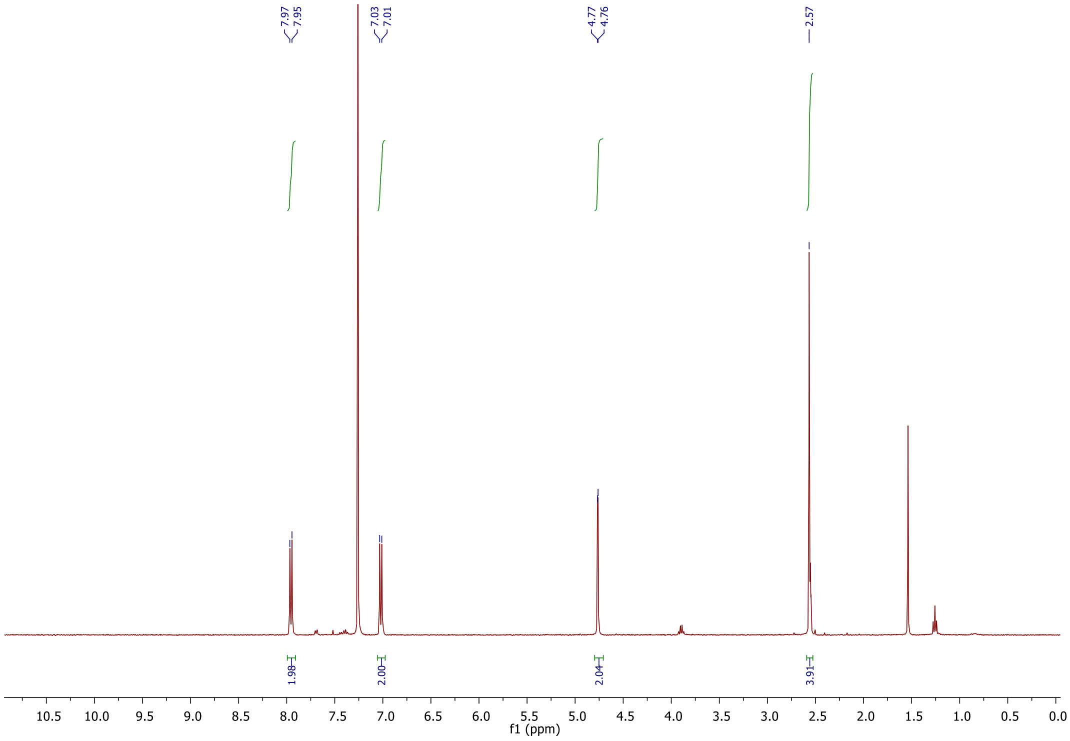

Figure S67. ^{1}H NMR (400 MHz, CDCl$_3$) of **10aa**.

10ab

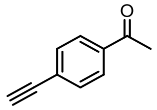

Synthesized according to the general procedure from 64 mg of **9ab**. The product was purified by flash chromatography using heptane/EtOAc as eluent to yield a colorless oil (46 mg, 0.26 mmol, Y = 67%). The analytical data was in good accordance with literature.[78]

^{1}H NMR (400 MHz, $CDCl_3$) δ 7.84 (d, *J* = 8.5 Hz, 2H), 7.51 (d, *J* = 8.5 Hz, 2H), 3.18 (s, 1H), 2.54 (s, 3H).

HRMS (ESI$^+$): exact mass calculated for $[M+H]^+$ ($C_{10}H_9O^+$) requires *m/z* = 145.0648, found *m/z* = 145.0648.

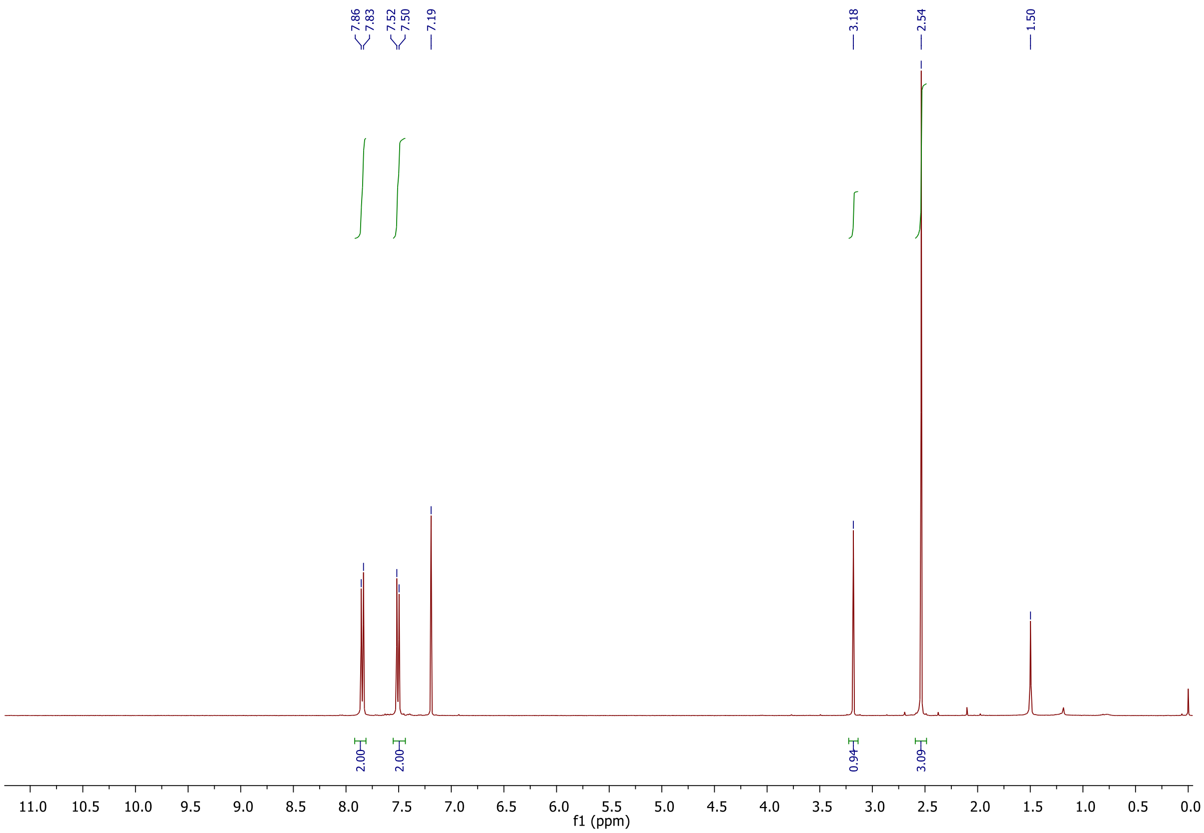

Figure S68. ^{1}H NMR (400 MHz, $CDCl_3$) of **10ab**.

10ac

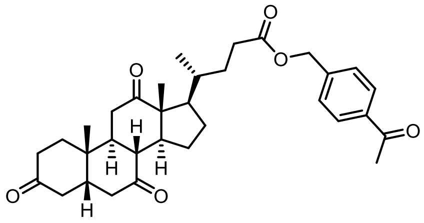

Synthesized according to the general procedure from 259 mg of **9ac** and using THF instead of EtOH (solubility issues). The product was purified by flash chromatography by using heptane/EtOAc as eluent to yield a white solid (171 mg, 0.32 mmol, Y = 64%).

^{1}H NMR (400 MHz, $CDCl_3$) δ 7.95 (d, J = 8.3 Hz, 2H), 7.44 (d, J = 8.2 Hz, 2H), 5.25 – 5.10 (m, 2H), 2.98 – 2.75 (m, 3H), 2.61 (s, 3H), 2.52 – 1.77 (m, 17H), 1.58 – 1.29 (m, 7H), 1.04 (s, 3H), 0.85 (d, J = 6.6 Hz, 3H).

^{13}C NMR (151 MHz, $CDCl_3$) δ 212.0, 209.1, 208.8, 197.8, 173.8, 141.5, 137.0, 128.7 (2C), 128.1 (2C), 65.4, 57.0, 51.9, 49.1, 47.0, 45.7, 45.7, 45.1, 42.9, 38.8, 36.6, 36.2, 35.6, 35.4, 31.5, 30.5, 27.8, 26.8, 25.3, 22.0, 18.8, 12.0.

HRMS (ESI$^+$): exact mass calculated for $[M+H]^+$ ($C_{33}H_{43}O_6^+$) requires m/z = 535.3054, found m/z = 535.3054.

IR (neat) ν_{max}: 2969, 1703, 1266, 1164, 701.

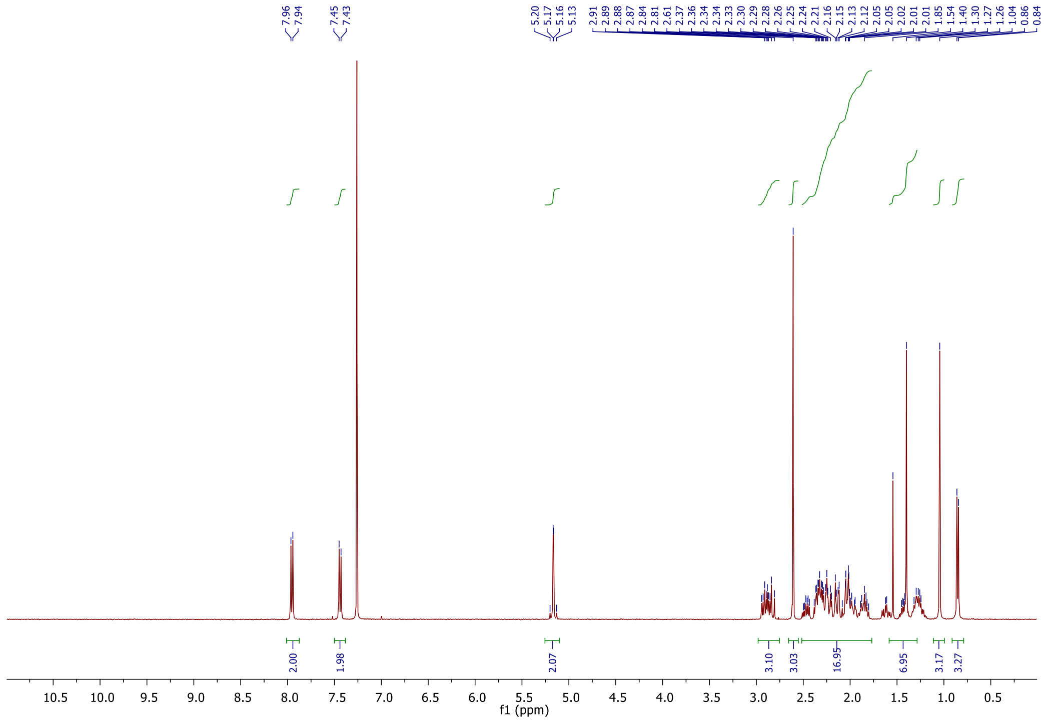

Figure S69. ^{1}H NMR (400 MHz, $CDCl_3$) of **10ac**.

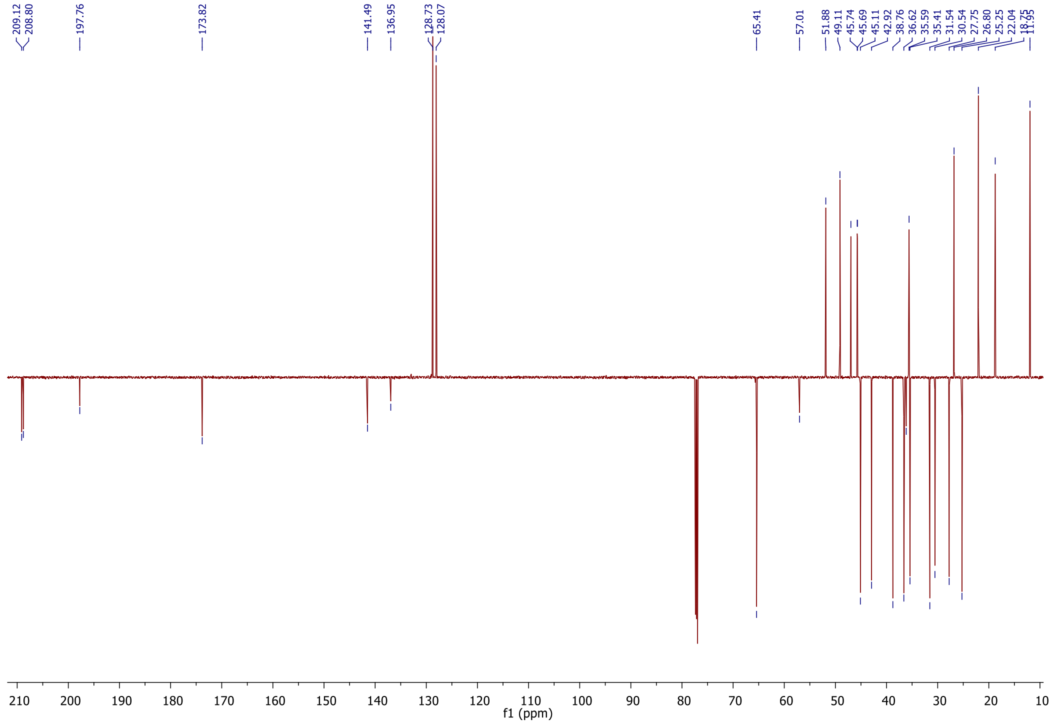

Figure S70. ^{13}C NMR (151 MHz, $CDCl_3$) of **10ac**.

10ad

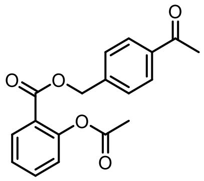

Synthesized according to the general procedure from 148 mg of **9ad** and using THF instead of EtOH (solubility issues). The product was purified by flash chromatography using heptane/EtOAc as eluent to yield a colorless oil (112 mg, 0.36 mmol, Y = 71%).

^{1}H NMR (400 MHz, $CDCl_3$) δ 8.07 (dd, *J* = 7.9, 1.7 Hz, 1H), 7.98 (d, *J* = 8.3 Hz, 2H), 7.58 (td, *J* = 7.8, 1.7 Hz, 1H), 7.51 (d, *J* = 8.2 Hz, 2H), 7.33 (td, *J* = 7.7, 1.1 Hz, 1H), 7.11 (dd, *J* = 8.1, 0.9 Hz, 1H), 5.36 (s, 2H), 2.62 (s, 3H), 2.20 (s, 3H).

^{13}C NMR (151 MHz, $CDCl_3$) δ 197.7, 169.8, 164.3, 150.9, 141.0, 137.1, 134.3, 132.0, 128.8 (2C), 128.2 (2C), 126.3, 124.1, 123.1, 66.2, 26.8, 21.0.

HRMS (ESI^+): exact mass calculated for $[M+Na]^+$ ($C_{18}H_{16}NaO_5^+$) requires *m*/*z* = 335.0890, found *m*/*z* = 335.0891.

IR (neat) ν_{max}: 1681, 1134, 753.

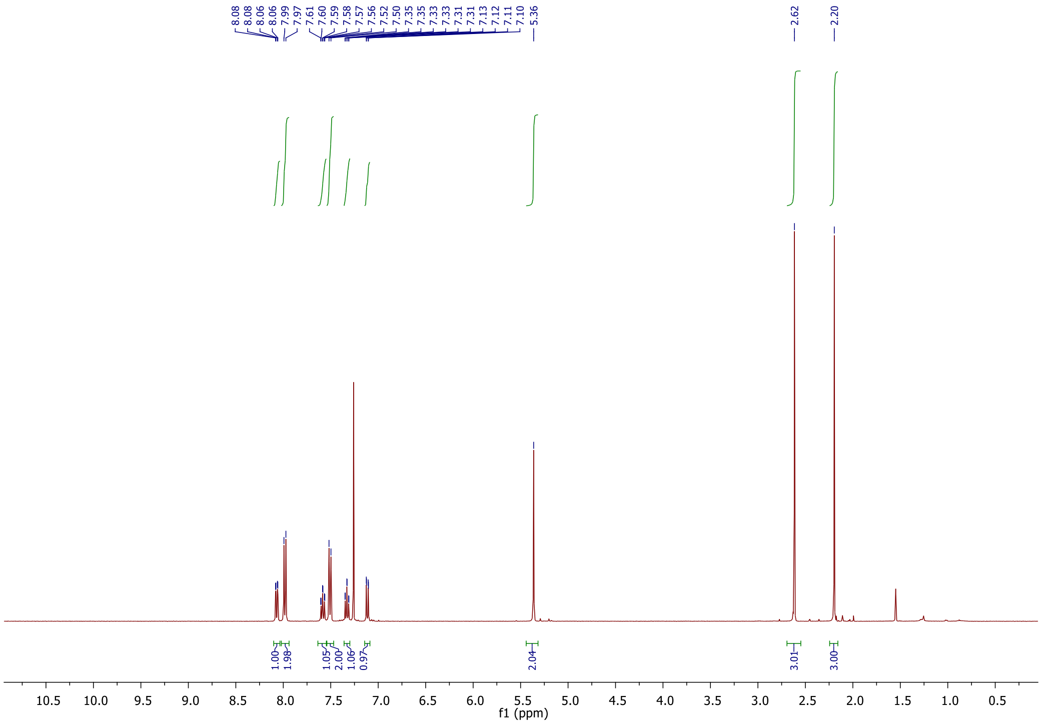

Figure S71. ^{1}H NMR (400 MHz, $CDCl_3$) of **10ad**.

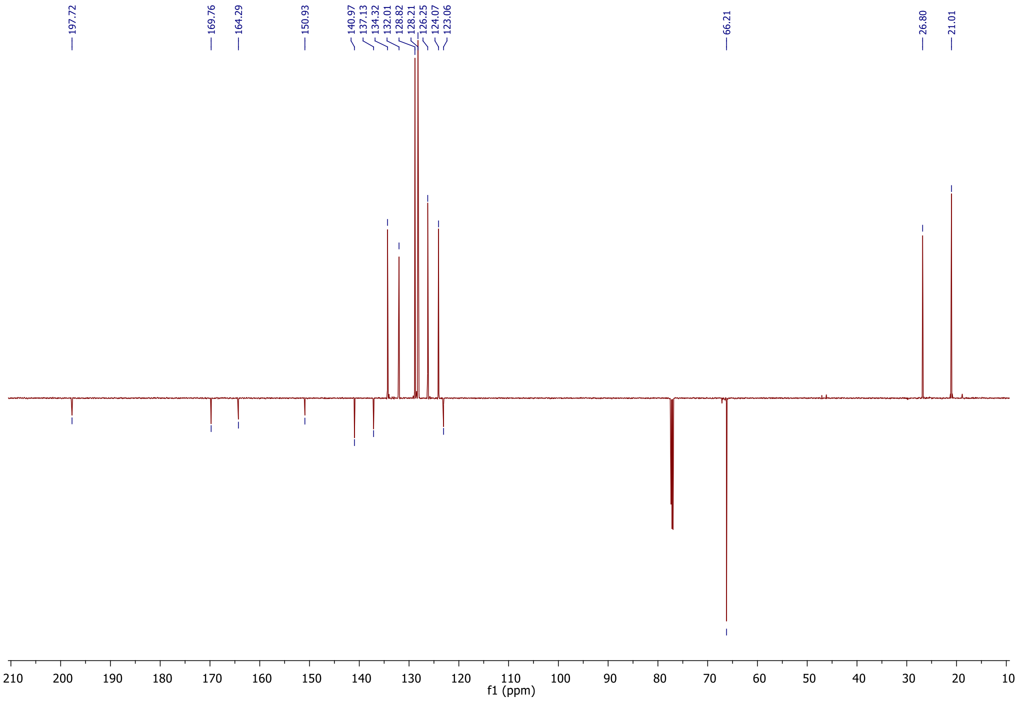

Figure S72. ^{13}C NMR (151 MHz, $CDCl_3$) of **10ad**.

10ae

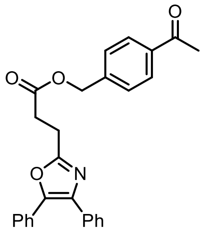

Synthesized according to the general procedure from 205 mg of **9ae**. The product was purified by flash chromatography by using heptane/EtOAc as eluent to yield a colorless solid (129 mg, 0.30 mmol, Y = 60%).

^{1}H NMR (700 MHz, $CDCl_3$) δ 7.84 (d, *J* = 8.2 Hz, 2H), 7.63 (d, *J* = 7.2 Hz, 2H), 7.55 (d, *J* = 7.0 Hz, 2H), 7.40 (d, *J* = 8.2 Hz, 2H), 7.38 – 7.29 (m, 6H), 5.22 (s, 2H), 3.21 (t, *J* = 7.2 Hz, 2H), 3.01 (t, *J* = 7.3 Hz, 2H), 2.53 (s, 3H).

^{13}C NMR (176 MHz, $CDCl_3$) δ 197.5, 171.7, 161.6, 145.5, 141.0, 136.7, 135.1, 132.4, 128.9, 128.7 (2C), 128.6 (2C), 128.5 (2C), 128.1 (2C), 127.9 (2C), 127.7 (2C), 126.5 (2C), 65.7, 31.0, 26.6, 23.5.

HRMS (ESI$^+$): exact mass calculated for $[M+H]^+$ ($C_{27}H_{24}NO_4^+$) requires *m/z* = 426.1700, found *m/z* = 426.1701.

IR (neat) ν_{max}: 1736, 1681, 1609, 1571, 1502, 1483, 1446, 1413, 1381, 1355, 1265, 1212, 1154, 1073, 1056, 1023, 998, 959, 915, 845, 815, 762, 692, 671, 642, 593, 519, 491, 450.

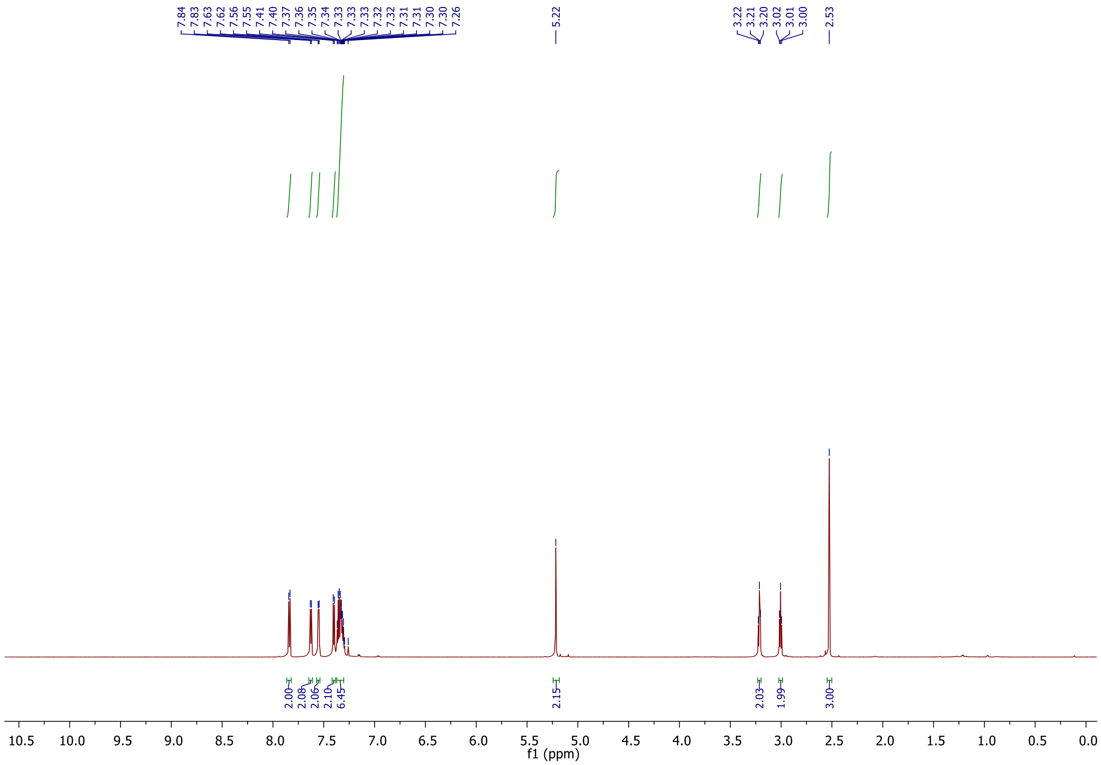

Figure S73. ^{1}H NMR (700 MHz, $CDCl_3$) of **10ae**.

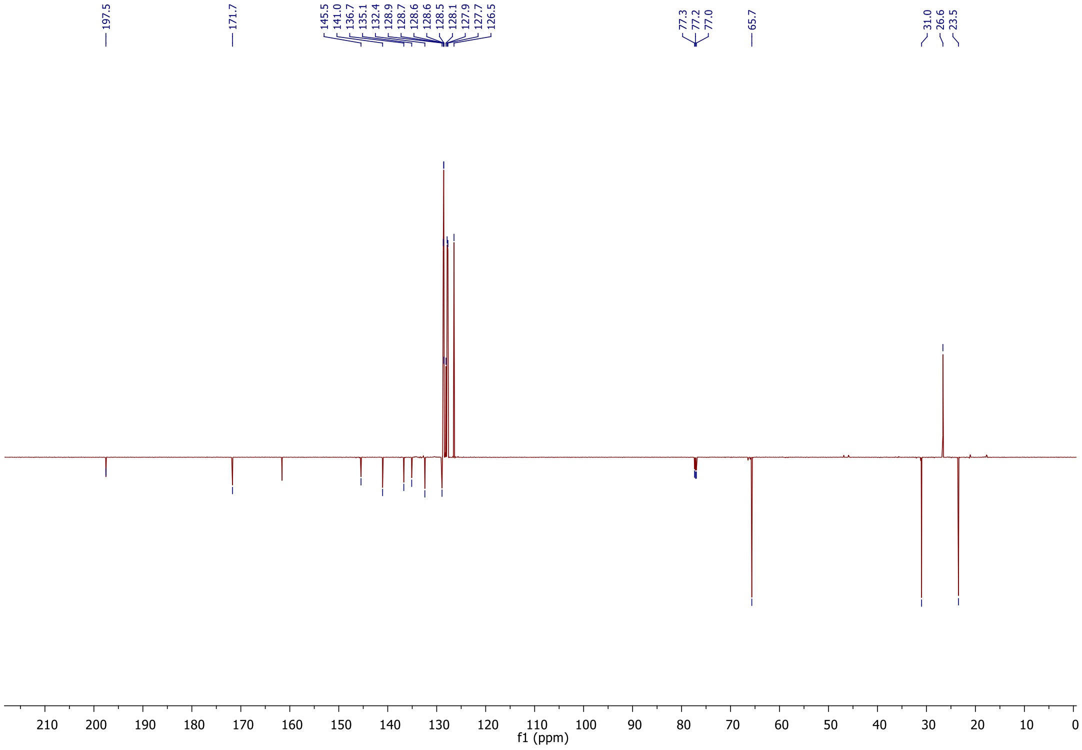

Figure S74. ^{13}C NMR (176 MHz, $CDCl_3$) of **10ae**.

10af

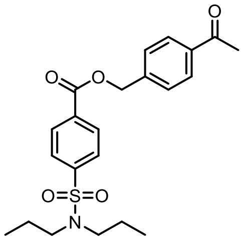

Synthesized according to the general procedure from 201 mg of **9af** and using THF instead of EtOH (solubility issues). The product was purified by flash chromatography by using heptane/EtOAc as eluent to yield a white solid (150 mg, 0.36 mmol, Y = 72%).

^{1}H NMR (400 MHz, $CDCl_3$) δ 8.19 (d, *J* = 8.6 Hz, 2H), 7.99 (d, *J* = 8.3 Hz, 2H), 7.89 (d, *J* = 8.6 Hz, 2H), 7.54 (d, *J* = 8.4 Hz, 2H), 5.44 (s, 2H), 3.10 (dd, *J* = 8.6, 6.7 Hz, 4H), 2.62 (s, 3H), 1.54 (dd, *J* = 8.6, 6.6 Hz, 4H), 0.87 (t, *J* = 7.4 Hz, 6H).

^{13}C NMR (151 MHz, $CDCl_3$) δ 197.7, 165.1, 144.8, 140.8, 137.3, 133.2, 130.5 (2C), 128.9 (2C), 128.2 (2C), 127.2 (2C), 66.6, 50.1 (2C), 26.8, 22.1 (2C), 11.3 (2C).

HRMS (ESI^+): exact mass calculated for $[M+H]^+$ ($C_{22}H_{28}NO_5S^+$) requires *m*/*z* = 418.1683, found *m*/*z* = 418.1683.

IR (neat) ν_{max}: 2967, 1681, 1262, 1084, 694, 600.

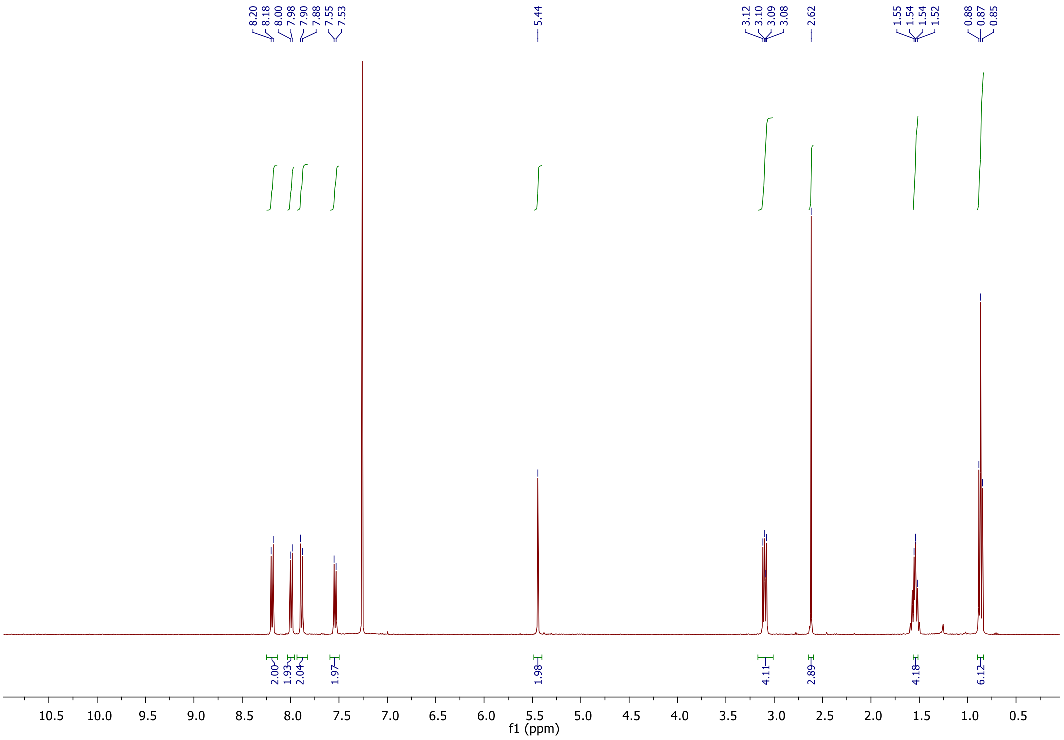

Figure S75. ^{1}H NMR (400 MHz, $CDCl_3$) of **10af**.

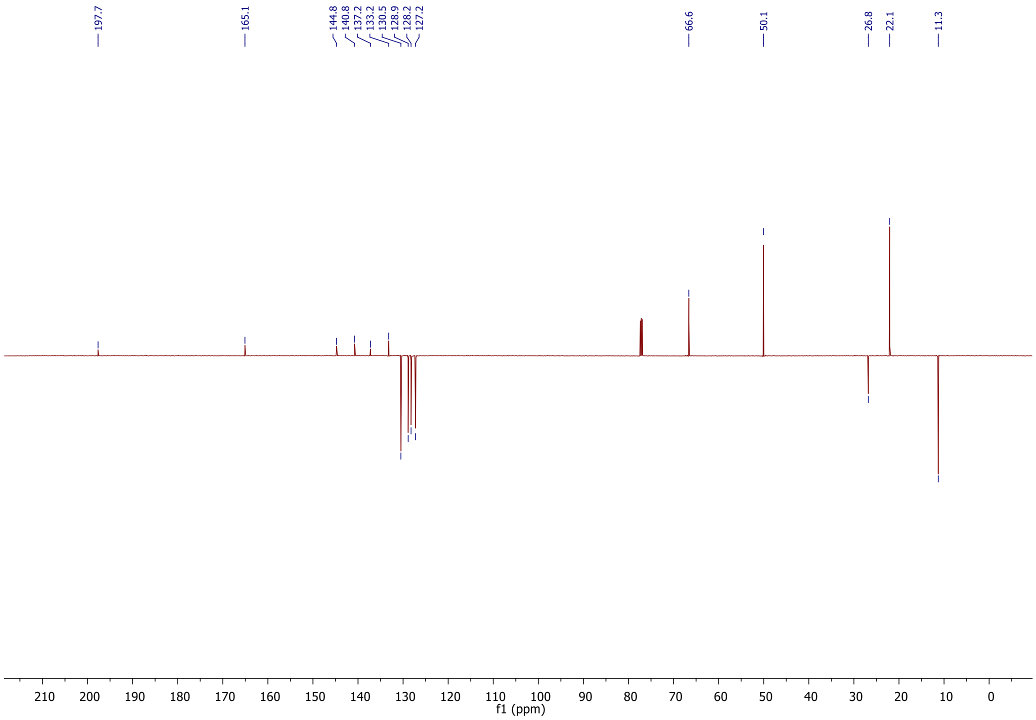

Figure S76. ^{13}C NMR (151 MHz, $CDCl_3$) of **10af**.

Mechanistic studies

10ag – TEMPO adduct

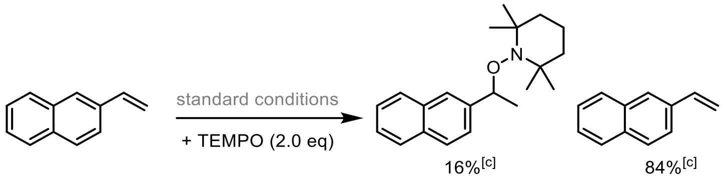

In a vial charged with the Co precatalyst (2.9 mg, 1 mol%) and a stirring bar, 77.1 mg of 2-vinylnapththalene (77.1 mg, 0.5 mmol, 1.0 eq) were added followed by EtOH (4 mL). The reaction mixture was stirred at 1000 rpm without being in direct contact with the stirring plate. After 1 min, phenylsilane (62 µL, 0.5 mmol, 1.0 eq) was added followed by TEMPO (159 mg, 2.0 eq, 1.0 mmol). After 18 h, the mixture was concentrated under reduced pressure and the residue was analyzed by ^{1}H NMR spectroscopy. Based on the conversion of the starting material, 16% of the TEMPO adduct was observed. The crude mixture was purified by flash chromatography using heptane/EtOAc as eluent to yield a mixture of the TEMPO adduct and triethoxy(phenyl)silane (96 mg, 13% purity, 0.04 mmol, Y = 8%). The characteristic NMR signals were found to be in good agreement with the literature.[79,80] The starting material could also be reisolated as a white solid (50 mg, 0.32 mmol, 65%).

HRMS (ESI$^+$): exact mass calculated for $[M+H]^+$ ($C_{21}H_{30}NO^+$) requires *m/z* = 312.2322, found *m/z* = 312.2320.

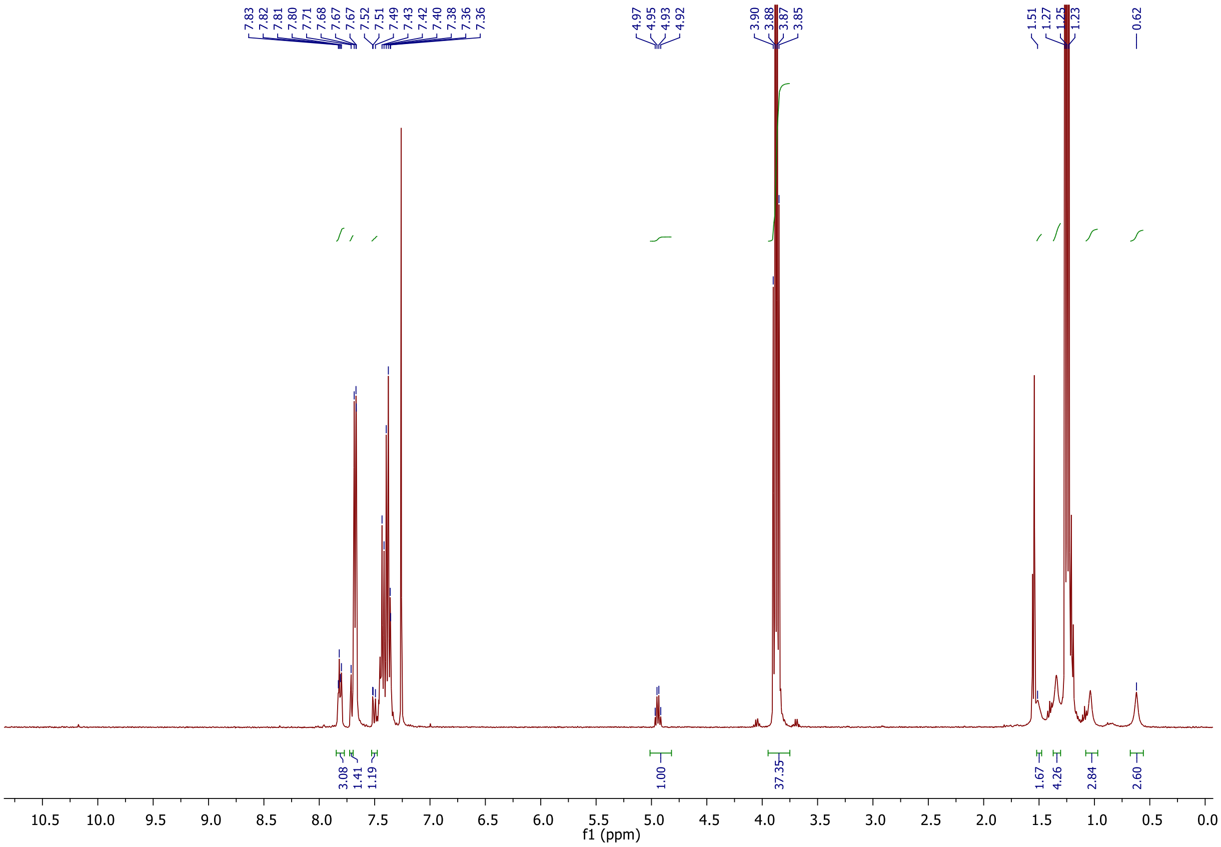

Figure S77. ^{1}H NMR (400 MHz, $CDCl_3$) of **10ag**.

$PhSiD_3$

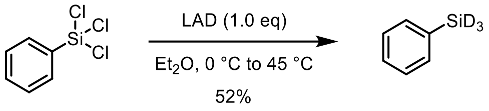

qNMR with 4.8 mg of product and 1.9 mg of 1,3,5-trimethoxybenzene

Lithium aluminum deuteride (168 mg, 4 mmol, 1.0 eq) was added to 10 mL round-bottomed flask, followed by diethyl ether (5 mL). The mixture was cooled with an ice-bath and phenyltrichlorosilane (641 μL, 4 mmol, 1.0 eq) was slowly added. After 5 min, the ice-bath was removed, and the mixture was stirred at 45 °C for 1.5 h. After this time, the mixture was allowed to cool to ambient temperature. The grey solution was filtered through Celite and eluted with diethyl ether. Cold water (~ 0 °C) was slowly added to the filtrate and the phases were separated. The organic phase was washed with cold water, filtered over $MgSO_4$ and concentrated under reduced pressure (920 mBar, 40 °C). When most of the solvent was removed, the residual oil was filtered with a syringe equipped with a filter and transferred to a vial. The oil was concentrated under reduced pressure (900 mBar, 40 °C to 450 mBar followed by 45 mBar, 0 °C) to yield a light-yellow oil (300 mg, determined at 77% by qNMR, 2.01 mmol, Y = 52%) which was found to be in good accordance with the literature.[81]

^{1}H NMR (400 MHz, $CDCl_3$) δ 7.62 – 7.57 (m, 2H), 7.46 – 7.30 (m, 3H).

Note on the purity of the D_3-phenylsilane: The compound was found to contain diethyl ether and other impurities present in the solvent used for the work-up.

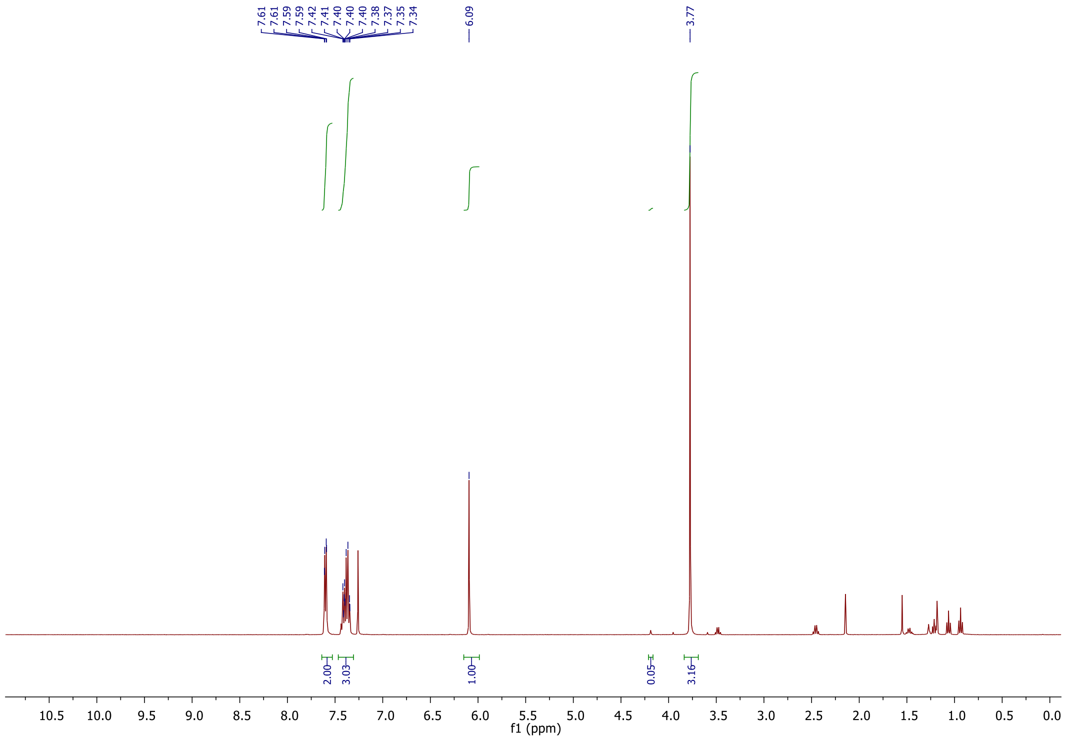

Figure S78. ^{1}H NMR (400 MHz, $CDCl_3$) of **$PhSiD_3$**.

[D]-10a

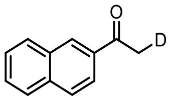

Synthesized according to the general procedure from 77.1 mg of 2-vinylnaphthalene and 72.2 mg of D_3-phenysilane (77% pure). The product was purified by flash chromatography using heptane/EtOAc as eluent to yield a white solid (63.3 mg, 0.37 mmol, Y = 74%). The analytical data was found to be in good accordance with the literature.[79]

^{1}H NMR (400 MHz, CDCl$_3$) δ 8.47 (s, 1H), 8.04 (dd, *J* = 8.6, 1.6 Hz, 1H), 7.97 (d, *J* = 8.0 Hz, 1H), 7.93 – 7.84 (m, 2H), 7.64 – 7.50 (m, 2H), 2.72 (m, 2H).

HRMS (ESI+): *m/z* calculated for $[M+Na]^+$ ($C_{12}H_9DONa^+$) = 194.0687, found *m/z* = 194.0685.

#	m/z	I
1	193.0621	6613
2	194.0685	154790
3	195.0725	25894

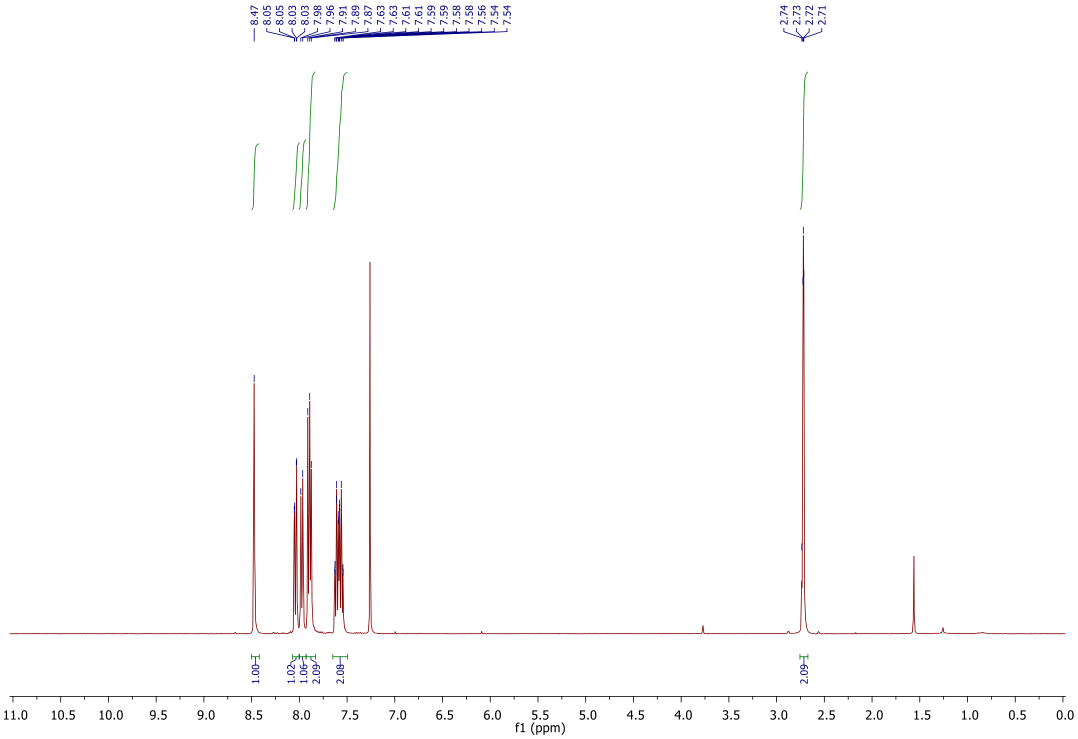

Figure S79. ^{1}H NMR (400 MHz, CDCl$_3$) of **[D]-10a**.

The chemical transformations of $PhSiH_3$ in the presence of 2 followed by ^{1}H NMR spectroscopy

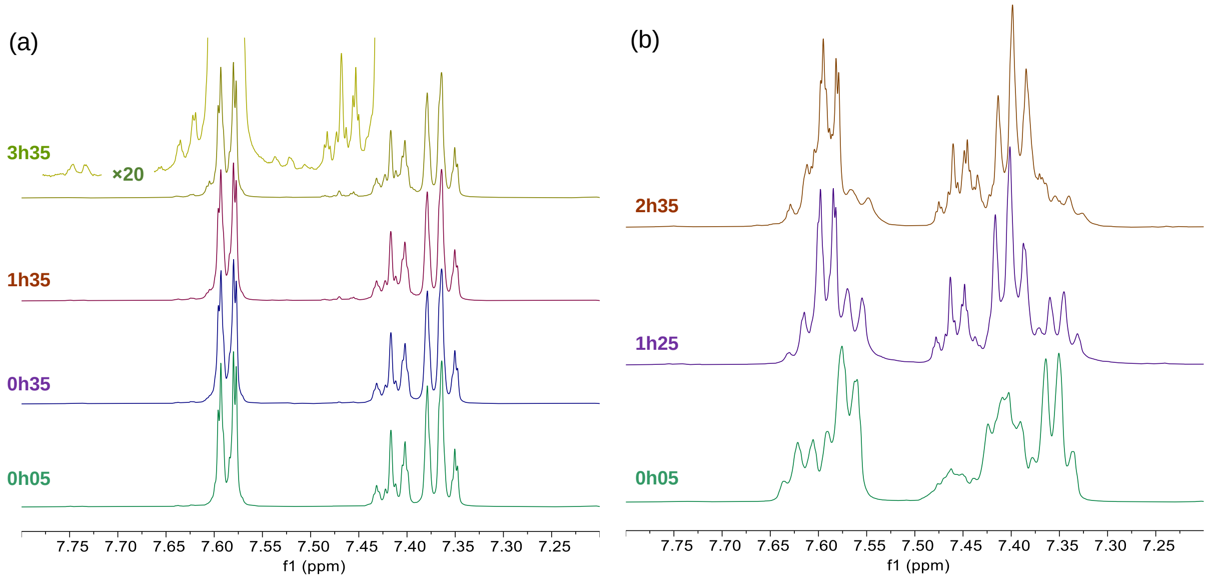

Figure S80. (a) ^{1}H NMR spectra of $PhSiH_3$ in MeOH-d_4 measured over time; (b) ^{1}H NMR spectra of $PhSiH_3$ in the presence of **2** MeOH-d_4 measured over time.

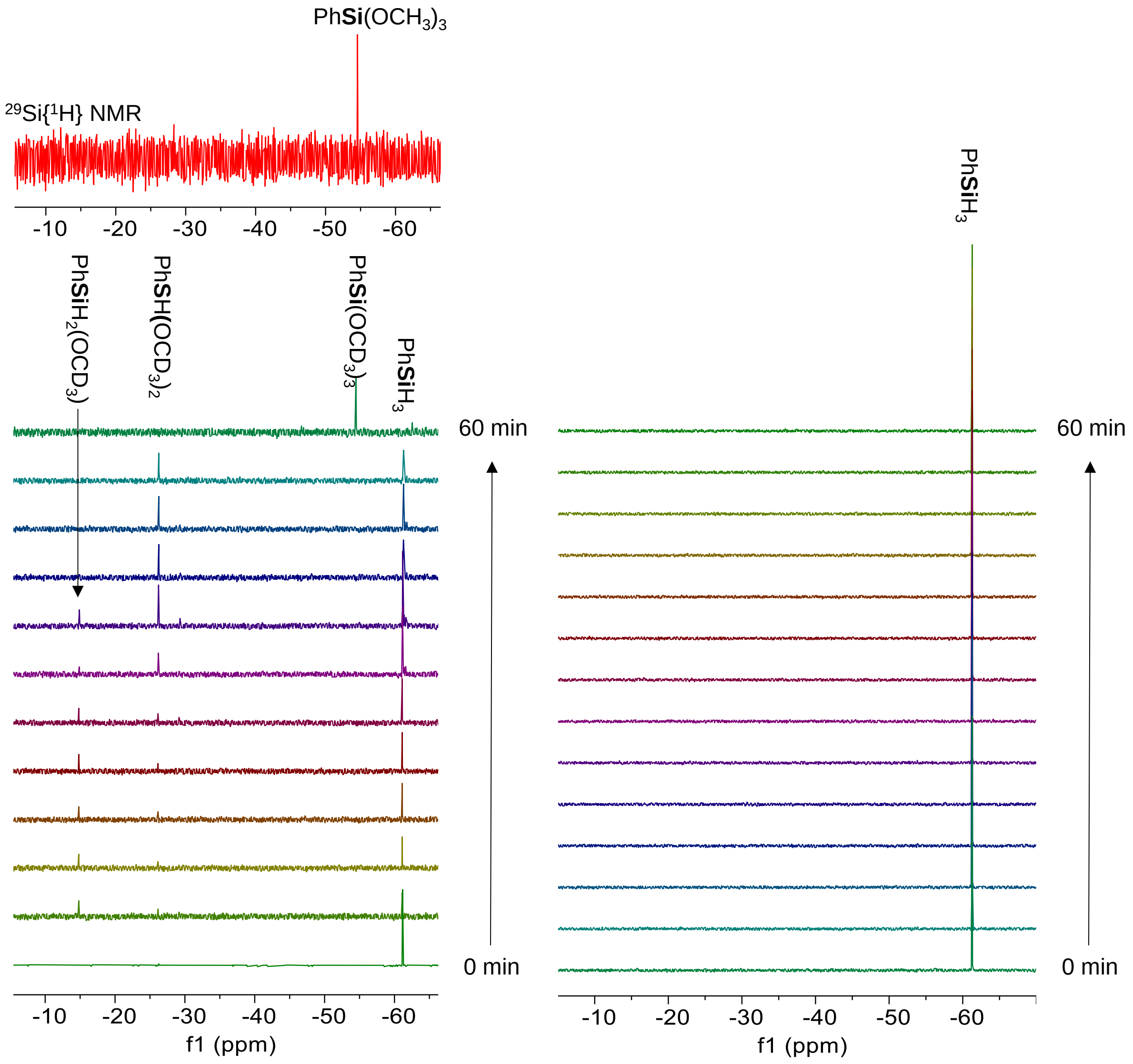

Figure S81. (left) Time-dependent $^{29}Si\{^{1}H\}$ NMR spectra of $PhSiH_3$ in the presence of compound **2**; (right) Time-independent $^{29}Si\{^{1}H\}$ NMR spectra of $PhSiH_3$ in MeOH-d_4.

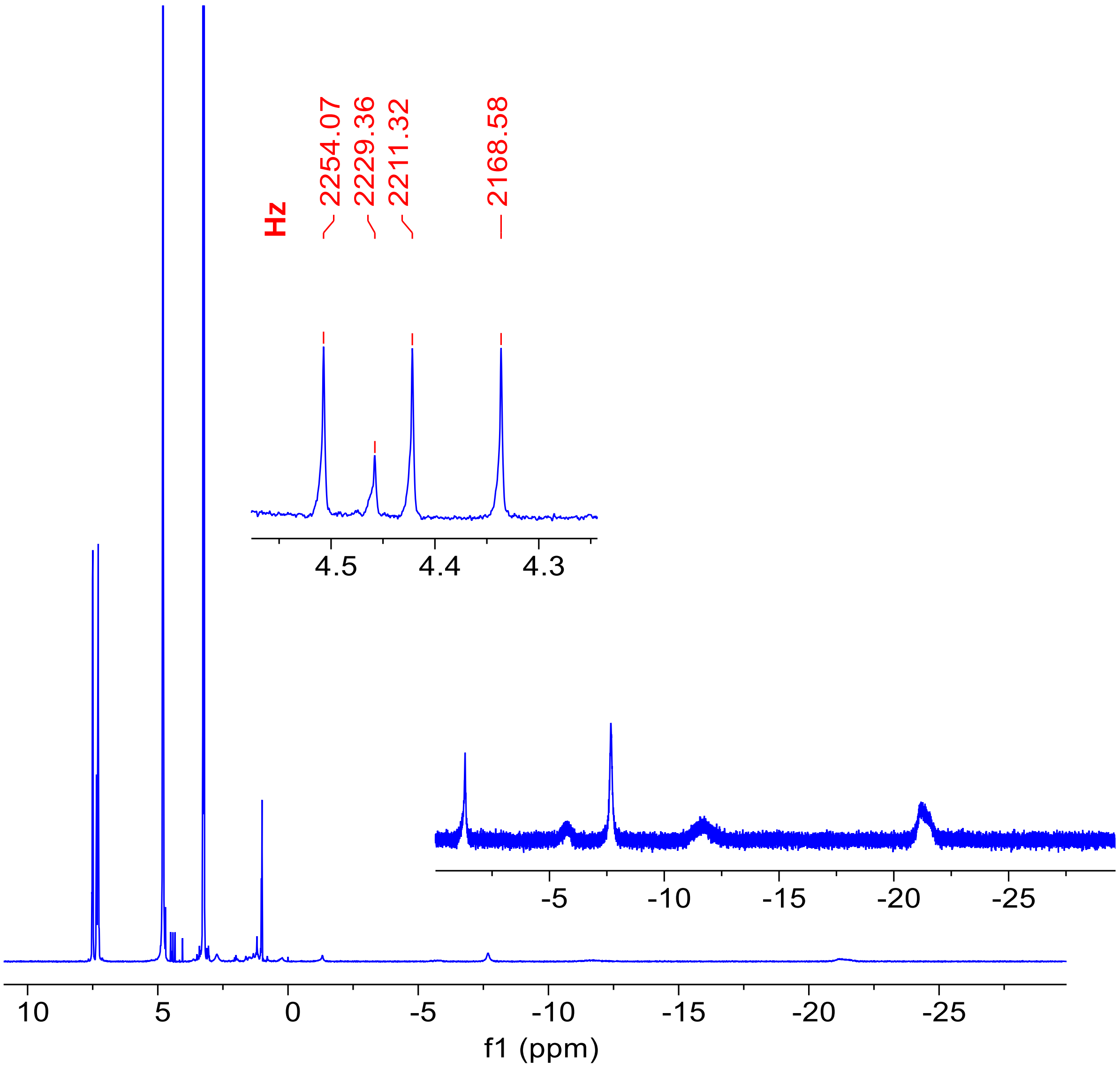

Figure S82. ^{1}H NMR spectrum of [Co^{III}(H$_2$L^{SCD3})I]I·CH$_3$OH in the presence of PhSiH$_3$ (1 equiv) in MeOH-*d*$_4$. The spectrum was recorded after 96 h on 502 MHz Bruker NMR spectrometer. Inset: the chraracteristic 1:1:1 triplet at 4.42 ppm for HD (with J^{HD} = 43 Hz) and the singlet at 4.46 ppm for H$_2$ in agreement with literature data.[82]

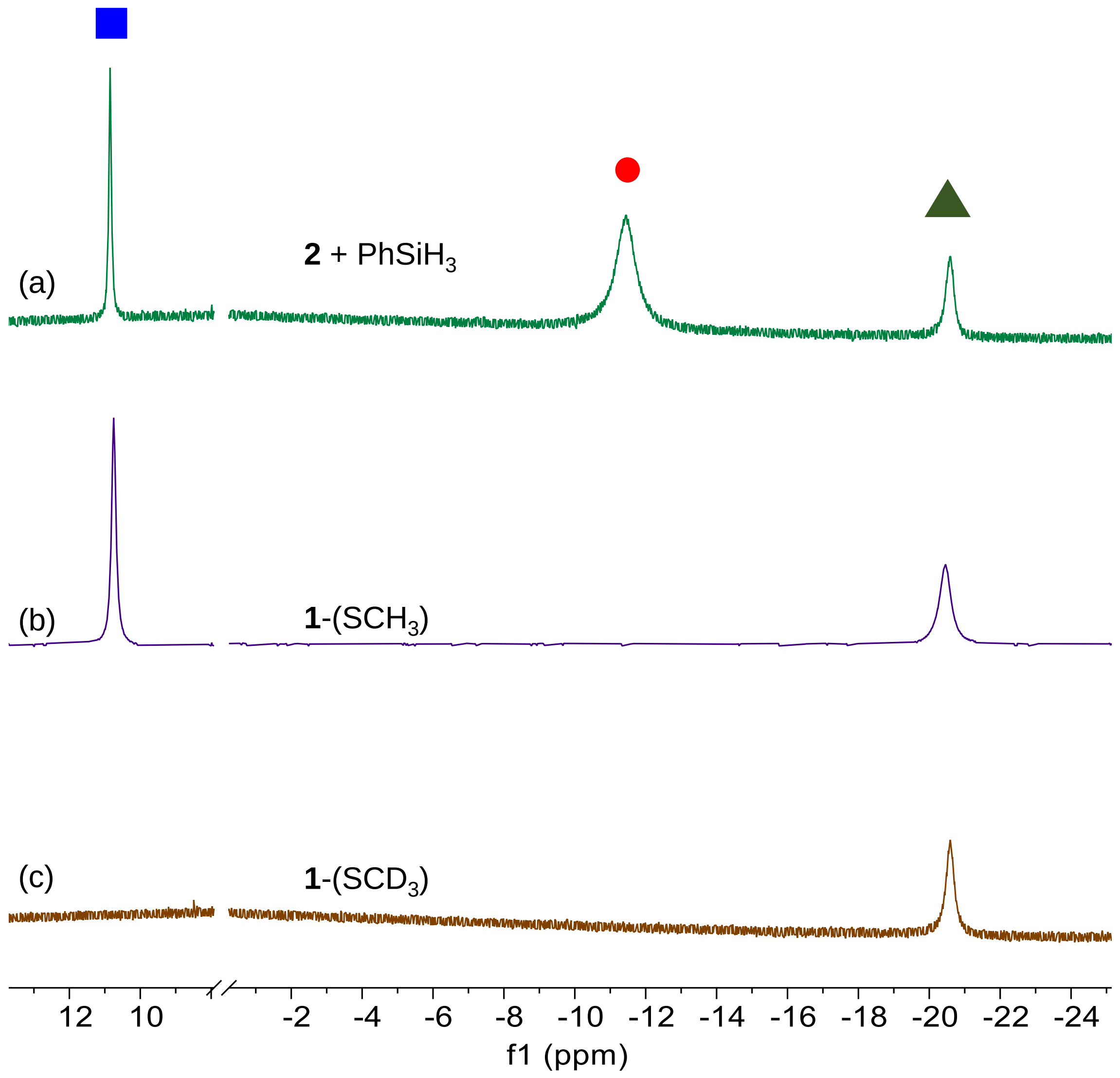

Figure S83. ^{1}H NMR spectra of (a) **2** + $PhSiH_3$ (b) **1** and (c) deuterated form of **1**-(SCD_3) in MeOH-d_4 at 298K (diamagnetic region omitted for clarity).

Section 8: Further investigation of catalytically active Co-species

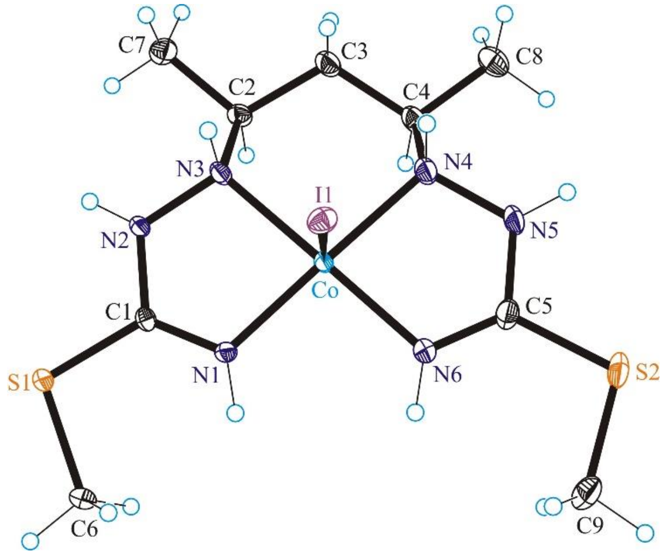

Figure S84. ORTEP view of the complex cation $[Co^{II}(H_4L^{SMe,red})I]^+$ in complex **12** with thermal ellipsoids drawn at 50% probability level. Iodide counteranion and one interstitial MeCN molecule are not shown. Selected bond lengths (Å) and bond angles (deg): Co–I1 2.8174(4), Co–N1 1.8938(18), Co–N3 1.9822(19), Co–N4 1.9659(19), Co–N6 1.9080(19), N1–C1 1.283(3), C1–N2 1.357(3), N2–N3 1.424(3), N3–C2 1.495(3), C2–C3 1.532(3), C3–C4 1.524(3), C4–N4 1.499(3), N4–N5 1.441(3), N5–C5 1.361(3), C5–N6 1.285(3); N1–Co–N3 83.21(8), N3–Co–N4 96.40(8), N4–Co–N6 83.24(8).

The complex is square-pyramidal with four nitrogen atoms (N1, N3, N4 and N6) in the basal plane and iodido co-ligand I1 in apical position. Reduction of two azomethine bonds resulted in chirality of four atoms (C2 and N3 with *S* configuration, while C4 and N4 with *R* configuration).

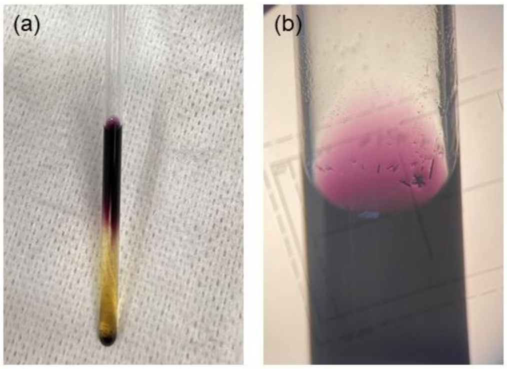

Figure S85. (a) The sample in Young-tube prepared in glovebox and left to stand in air. The photo of the tube was captured after 24 h; (b) The photo of the crystals of $[Co^{III}(L^{SMe\bullet\bullet})I]$ under the microscope. The crystals were formed on the top of the violet solution after 96 h.

Small differences in the bond distances in the two ligands are due to the *cis*-arrangement of the two isothiosemicarbazide moieties which are now incorporated into a tetradentate *N*,*N*,*N*,*N*-ligand platform PBIT and the presence of electron-donating aliphatic groups of the reduced Hacac moiety in [Co^{III}(L$^{SMe••}$)I] instead of electron-withdrawing Ph groups in the two *S*-methyl-1-phenyl-isothiosemicarbazide ligands in *trans*-[Co^{III}(Q$^{Me•}$)$_2$I] (see Chart 2 in the main text).

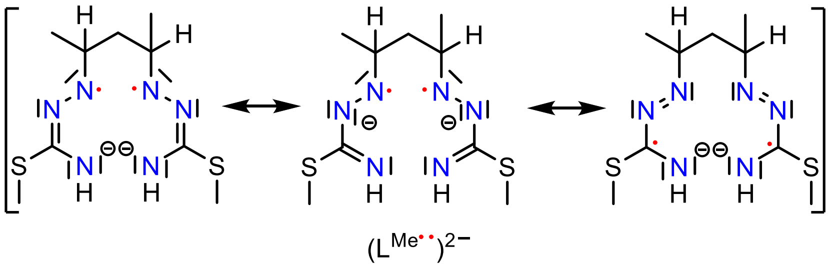

Chart S1. Resonance structures for species (L$^{Me••}$)$^{2–}$.

Table S11. Total B3LYP/def2-TZVP energies, DFT energies (ΔE), expectation values of S^2 and localization orbital analysis, MPA charges and spin on Co, *J*-coupling for studied complexes.

B3LYP	E [hartree]	ΔE [kJ/mol]	S^2	Co d LOC	Charge/spin	*J* / cm^{-1}
**^{1}[Co$^{•}$(L$^{SMe•}$)I]^{0}**	–3159.68575	5.8	0		0.055	
**^{3}[Co$^{•}$(L$^{SMe•}$)I]^{0}**	–3159.67462	35.0	2.064	4α+2.5*β	0.165/1.327	–1947.501
**^{u1}[Co$^{•}$(L$^{SMe•}$)I]^{0}**	**–3159.68796**	**0**	**0.561**	**4α+2.5*β**	**0.105/0.853**	
**^{1}[Co$^{•}$(L$^{SMe•}$)]$^{+}$**	–2861.62782	25.5	0		0.134	
**^{3}[Co$^{•}$(L$^{SMe•}$)]$^{+}$**	–2861.63498	6.7	2.013	4α+3β	0.201/1.186	–543.317
**^{u1}[Co$^{•}$(L$^{SMe•}$)]$^{+}$**	**–2861.63752**	**0**	**0.986**	**4α+2.5β***	**0.226/1.114**	
**^{1}[Zn(L$^{SMe••}$)]^{0}**	–3258.41201	56.8	0		0.191	
**^{3}[Zn(L$^{SMe••}$)]^{0}**	**3258.43365**	**0**	**2.010**	**5α+5β**	**0.261/0.003**	**101.574**
**^{u1}[Zn(L$^{SMe••}$)]^{0}**	–3258.43318	1.2	1.005	5α+5β	0.261/0.0009	
**^{1}[Ni(L$^{SMe••}$)]^{0}**	–2987.40237	16.0	0		0.111	
**^{3}[Ni(L$^{SMe••}$)]^{0}**	**–2987.40846**	**0**	**2.030**	**4α+4β**	**0.116/-0.002**	**1223.789**
**^{u1}[Ni(L$^{SMe••}$)]^{0}**	–2987.40124	18.9	0.736	4α+4β	0.128/0.096	

*two localized π(Co-N) orbitals have Co population of ca. 0.25 each

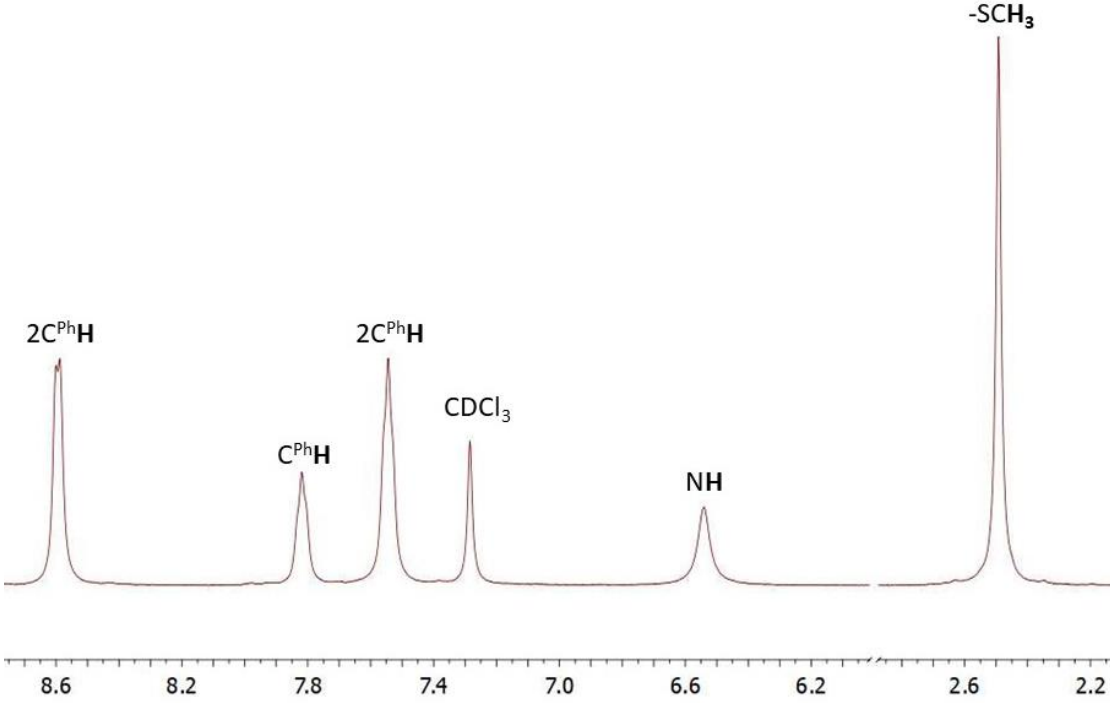

Figure S86. ^{1}H NMR spectrum of the complex $[Co^{III}(Q^{Me\bullet})_2I]$ in $CDCl_3$.

References

(1) Arion, V.; Wieghardt, K.; Weyhermueller, T.; Bill, E.; Leovac, V.; Rufinska, A. Synthesis, Structure, Magnetism, and Spectroscopic Properties of Some Mono- and Dinuclear Nickel Complexes Containing Noninnocent Pentane-2,4-Dione Bis(*S*-Alkylisothiosemicarbazonate)-Derived Ligands. *Inorg. Chem.* **1997**, *36*, 661–669.

(2) Freund, M.; Paradies, T. Zur Kentniss Des Tetrazols. *Ber. Dtsch. Chem. Ges.* **1901**, *34*, 3110–3122.

(3) SAINT Plus, 2016.

(4) Stoe; Cie. X-AREA, X-RED and X-SHAPE.

(5) Sheldrick, G. M. A Short History of *SHELX*. *Acta Crystallogr: A: Found. Crystallogr.* **2008**, *64*, 112–122.

(6) Burnett, M. N.; Johnson, C. K. *ORTEP-III: Oak Ridge Thermal Ellipsoid Plot Program for Crystal Structure Illustrations*; ORNL--6895, 369685; 1996.

(7) Gerbeleu, N. V.; Arion, V. B.; Simonov, Yu. A.; Zavodnik, V. E.; Stavrov, S. S.; Turta, K. I.; Gradinaru, D. I.; Birca, M. S.; Pasynskii, A. A.; Ellert, O. Synthesis, Geometrical and Electronic Structure of Iron Mononitrosyl Complexes with Bis(*S*-Alkylisothiosemicarbazones) of β-Dicarbonyl Compounds. *Inorg. Chim. Acta* **1992**, *202*, 173–181.

(8) Knof, U. Die Koordinationschemie von "Non-Innocent" Bis(*S*-Alkylisothiosemicarbazonat)-Liganden Mit Übergangsmetallionen, Ruhr-Universität Bochum, Bochum, 1995.

(9) Bilyj, J. K.; Silajew, N. V.; Bernhardt, P. V. Nickel Coordination Chemistry of Bis(Dithiocarbazate) Schiff Base Ligands; Metal and Ligand Centred Redox Reactions. *Dalton Trans.* **2021**, *50*, 612–623.

(10) Peng, S.-M.; Liaw, D.-S.; Wang, Y.; Simon, A. Cofacial Dimer of a Diiminosuccinonitrile Complex Containing a Co^{II}–Co^{II} Bond and Its Reduction to Monomeric Co^{I} Complex. *Angew. Chem. Int. Ed. Engl.* **1985**, *24*, 210–211.

(11) You, Y.-S.; Lee, G.-H.; Peng, S.-M. Chemical Transformations of (2,3,9,10-Tetramethyl-1,4,5,7,8,11,12,14-Octa-Azacyclotetradeca-1,3,8,10-Tetraenato)Cobalt(II)Perchlorate. *Jnl Chinese Chemical Soc* **1996**, *43*, 261–276.

(12) Chern, S.-S.; Liaw, M.-C.; Peng, S.-M. Eclipsed Cofacial Dimers of Metal Complexes Containing d^7–d^7 and d^8–d^8 Metal Bonds. Crystal Structure of Bis[Bis{(Phenanthrene-9,10-Diamin)ylato}cobalt(II)] and Bis[{(2,3-Dicyanoethenediamin)ylato}dicarbonyl rhodium(I)]. *J. Chem. Soc., Chem. Commun.* **1993**, 359–361.

(13) Ravel, B.; Newville, M. *ATHENA* , *ARTEMIS* , *HEPHAESTUS* : Data Analysis for X-Ray Absorption Spectroscopy Using *IFEFFIT*. *J. Synchrotron Rad.* **2005**, *12*, 537–541.

(14) Hall, J. W.; Marsh, W. E.; Weller, R. R.; Hatfield, W. E. Exchange Coupling in the Alternating-Chain Compounds Catena-Di-μ-Chloro-Bis(4-Methylpyridine)Copper(II), Catena-Di-μ-Bromobis(N-Methylimidazole)Copper(II), Catena-[Hexanedione)Bis(Thiosemicarbazonato)]Copper(II), and Catena-[Octanedione Bis (Thiosemicarbazonato)]Copper(II). *Inorg. Chem.* **1981**, *20*, 1033–1037.

(15) Hatfield, W. E. New Magnetic and Structural Results for Uniformly Spaced, Alternatingly Spaced, and Ladder-like Copper(II) Linear Chain Compounds. *J. Appl. Phys.* **1981**, *52*, 1985–1990.

(16) Nishida, Y.; Kida, S. Ground States of the Square Planar Low-Spin Cobalt(II) Complexes. *Bull. Chem. Soc. Japan* **1978**, *51*, 143–149.
(17) McGarvey, B. R. Theory of the Spin Hamiltonian Parameters for Low Spin Cobalt(II) Complexes. *Can. J. Chem.* **1975**, *53*, 2498–2511.
(18) Dey, S.; Wayland, B. B.; Zdilla, M. J. Solution and Solid State Properties for Low-Spin Cobalt(II) Dibenzotetramethyltetraaza[14]Annulene [(Tmtaa)Co^{II}] and the Monopyridine Complex. *Inorg. Chem.* **2019**, *58*, 1224–1233.
(19) Inoue, M.; Kubo, M. Magnetic Susceptibilities of Alternating Linear Ising Antiferromagnets and Two Coupled Ising Chains. *J. Magn. Res.* **1971**, *4*, 175–183.
(20) Hara, K. ichi; Inoue, M.; Emori, S.; Kubo, M. Magnetic Interaction between Binuclear Clusters in Potassium Trihalocuprates(II) and Dimethylammonium Trihalocuprates(II). *J. Magn. Res.* **1971**, *4*, 337–346.
(21) Drulis, H.; Dyrek, K.; Hoffmann, K. P.; Hoffmann, S. K.; Weselucha-Birczynska, A. EPR Spectra of Low-Symmetry Tetrahedral High-Spin Cobalt(II) in a Cinchoninium Tetrachlorocobaltate(II) Dihydrate Single Crystal. *Inorg. Chem.* **1985**, *24*, 4009–4012.
(22) van Stapele, R. P.; Beljers, H. G.; Bongers, P. F.; Zijlstra, H. Ground State of Divalent Co Ions in Cs_3CoCl_5 and Cs_3CoBr_5. *J. Chem. Phys.* **1966**, *44*, 3719–3725.
(23) Connelly, N. G.; Geiger, W. E. Chemical Redox Agents for Organometallic Chemistry. *Chem. Rev.* **1996**, *96*, 877–910.
(24) Ohui, K.; Stepanenko, I.; Besleaga, I.; Babak, M. V.; Stafi, R.; Darvasiova, D.; Giester, G.; Pósa, V.; Enyedy, E. A.; Vegh, D.; Rapta, P.; Ang, W. H.; Popović-Bijelić, A.; Arion, V. B. Triapine Derivatives Act as Copper Delivery Vehicles to Induce Deadly Metal Overload in Cancer Cells. *Biomolecules* **2020**, *10*, 1336.
(25) Römelt, C.; Weyhermüller, T.; Wieghardt, K. Structural Characteristics of Redox-Active Pyridine-1,6-Diimine Complexes: Electronic Structures and Ligand Oxidation Levels. *Coord. Chem. Rev.* **2019**, *380*, 287–317.
(26) Brown, S. N. Metrical Oxidation States of 2-Amidophenoxide and Catecholate Ligands: Structural Signatures of Metal–Ligand π Bonding in Potentially Noninnocent Ligands. *Inorg. Chem.* **2012**, *51*, 1251–1260.
(27) Becke, A. D. Density-Functional Exchange-Energy Approximation with Correct Asymptotic Behavior. *Phys. Rev. A* **1988**, *38*, 3098–3100.
(28) Lee, C.; Yang, W.; Parr, R. G. Development of the Colle-Salvetti Correlation-Energy Formula into a Functional of the Electron Density. *Phys. Rev. B* **1988**, *37*, 785–789.
(29) Becke, A. D. Density-functional Thermochemistry. III. The Role of Exact Exchange. *J. Chem. Phys.* **1993**, *98*, 5648–5652.
(30) Vosko, S. H.; Wilk, L.; Nusair, M. Accurate Spin-Dependent Electron Liquid Correlation Energies for Local Spin Density Calculations: A Critical Analysis. *Can. J. Phys.* **1980**, *58*, 1200–1211.
(31) Krishnan, R.; Binkley, J. S.; Seeger, R.; Pople, J. A. Self-consistent Molecular Orbital Methods. XX. A Basis Set for Correlated Wave Functions. *J. Chem. Phys.* **1980**, *72*, 650–654.
(32) McLean, A. D.; Chandler, G. S. Contracted Gaussian Basis Sets for Molecular Calculations. I. Second Row Atoms, *Z* =11–18. *J. Chem. Phys.* **1980**, *72*, 5639–5648.
(33) MOLDRAW: Molecular Graphics on a Personal Computer. *Z. Kristallogr. – Cryst. Mater.* **1993**, *207*, 9–23.

(34) Neese, F. The ORCA Program System. *WIREs Comput Mol Sci* **2012**, *2*, 73–78.
(35) Bauernschmitt, R.; Ahlrichs, R. Treatment of Electronic Excitations within the Adiabatic Approximation of Time Dependent Density Functional Theory. *Chem. Phys. Lett.* **1996**, *256*, 454–464.
(36) Scalmani, G.; Frisch, M. J.; Mennucci, B.; Tomasi, J.; Cammi, R.; Barone, V. Geometries and Properties of Excited States in the Gas Phase and in Solution: Theory and Application of a Time-Dependent Density Functional Theory Polarizable Continuum Model. *J. Chem. Phys.* **2006**, *124*, 094107.
(37) Neese, F. Software Update: The ORCA Program System, Version 4.0. *WIREs Comput. Mol. Sci.* **2018**, *8*, e1327.
(38) Neese, F.; Wennmohs, F.; Becker, U.; Riplinger, C. The ORCA Quantum Chemistry Program Package. *J. Chem. Phys.* **2020**, *152*, 224108.
(39) DeBeer George, S.; Petrenko, T.; Neese, F. Prediction of Iron K-Edge Absorption Spectra Using Time-Dependent Density Functional Theory. *J. Phys. Chem. A* **2008**, *112*, 12936–12943.
(40) Mao, W.; Fehn, D.; Heinemann, F. W.; Scheurer, A.; van Gastel, M.; Jannuzzi, S. A. V.; DeBeer, S.; Munz, D.; Meyer, K. Umpolung in a Pair of Cobalt(III) Terminal Imido/Imidyl Complexes. *Angew. Chem. Int. Ed.* **2022**, *61*, e202206848.
(41) Dirac, P. A. M. Quantum Mechanics of Many-Electron Systems. *Proceedings of the Royal Society of London. Series A, Containing Papers of a Mathematical and Physical Character* **1929**, *123*, 714–733.
(42) Slater, J. C. A Simplification of the Hartree-Fock Method. *Phys. Rev.* **1951**, *81*, 385–390.
(43) Neese, F.; Wennmohs, F.; Hansen, A.; Becker, U. Efficient, Approximate and Parallel Hartree–Fock and Hybrid DFT Calculations. A 'Chain-of-Spheres' Algorithm for the Hartree–Fock Exchange. *Chem. Phys.* **2009**, *356*, 98–109.
(44) Weigend, F.; Ahlrichs, R. Balanced Basis Sets of Split Valence, Triple Zeta Valence and Quadruple Zeta Valence Quality for H to Rn: Design and Assessment of Accuracy. *Phys. Chem. Chem. Phys.* **2005**, *7*, 3297–3305.
(45) Weigend, F. Accurate Coulomb-Fitting Basis Sets for H to Rn. *Phys. Chem. Chem. Phys.* **2006**, *8*, 1057–1065.
(46) Plasser, F.; Wormit, M.; Dreuw, A. New Tools for the Systematic Analysis and Visualization of Electronic Excitations. I. Formalism. *J. Chem. Phys.* **2014**, *141*, 024106.
(47) Wang, R.; Chen, Y.; Shu, M.; Zhao, W.; Tao, M.; Du, C.; Fu, X.; Li, A.; Lin, Z. $AuCl_3$ -Catalyzed Ring-Closing Carbonyl–Olefin Metathesis. *Chem. Eur. J.* **2020**, *26*, 1941–1946.
(48) Yu, S.; Noble, A.; Bedford, R. B.; Aggarwal, V. K. Methylenespiro[2.3]Hexanes via Nickel-Catalyzed Cyclopropanations with [1.1.1]Propellane. *J. Am. Chem. Soc.* **2019**, *141*, 20325–20334.
(49) Yan, X.-B.; Li, L.; Wu, W.-Q.; Xu, L.; Li, K.; Liu, Y.-C.; Shi, H. Ni-Catalyzed Hydroalkylation of Olefins with N-Sulfonyl Amines. *Nat. Commun.* **2021**, *12*, 5881.
(50) Woods, J.; Masterson, M. Method for Production of Allyloxystyrene Compounds. EP0881205A1, 1997.
(51) Paul, C. E.; Rajagopalan, A.; Lavandera, I.; Gotor-Fernández, V.; Kroutil, W.; Gotor, V. Expanding the Regioselective Enzymatic Repertoire: Oxidative Mono-Cleavage of Dialkenes Catalyzed by Trametes Hirsuta. *Chem. Commun.* **2012**, *48*, 3303–3305.

(52) Calder, E. D. D.; Sharif, S. A. I.; McGonagle, F. I.; Sutherland, A. One-Pot Synthesis of 5-Amino-2,5-Dihydro-1-Benzoxepines: Access to Pharmacologically Active Heterocyclic Scaffolds. *J. Org. Chem.* **2015**, *80*, 4683–4696.
(53) Yang, F.; Rauch, K.; Kettelhoit, K.; Ackermann, L. Aldehyde-Assisted Ruthenium(II)-Catalyzed C⍰H Oxygenations. *Angew. Chem. Int. Ed.* **2014**, *53*, 11285–11288.
(54) Baillet, J.; Gaubert, A.; Bassani, D. M.; Verget, J.; Latxague, L.; Barthélémy, P. Supramolecular Gels Derived from Nucleoside Based Bolaamphiphiles as a Light-Sensitive Soft Material. *Chem. Commun.* **2020**, *56*, 3397–3400.
(55) Zhang, X.; Xie, X.; Liu, Y. Nickel-Catalyzed Highly Regioselective Hydrocyanation of Terminal Alkynes with Zn(CN)$_2$ Using Water as the Hydrogen Source. *J. Am. Chem. Soc.* **2018**, *140*, 7385–7389.
(56) Chen, F.; Tang, Y.; Li, X.; Duan, Y.; Chen, C.; Zheng, Y. Oxoammonium Salt-Mediated Vicinal Oxyazidation of Alkenes with NaN$_3$: Access to β -Aminooxy Azides. *Adv. Synth. Catal.* **2021**, *363*, 5079–5084.
(57) Breviglieri, G.; Bruno, G.; Contrini, S.; Assanelli, C. Process for the Preparation of 4.5-Diphenyloxazole-2-Propanoic Acid. US Patent, 2000.
(58) Ma, J.; Zou, Q.; Wang, C.; Yin, G.; Li, F. Development of β,β-Dibrominated Secondary Enamides and Their Application to the Synthesis of 5-Br Oxazoles. *J. Org. Chem.* **2022**, *87*, 15670–15678.
(59) Havare, N.; Plattner, D. A. Oxidative Cleavage of α-Aryl Aldehydes Using Iodosylbenzene. *Org. Lett.* **2012**, *14*, 5078–5081.
(60) Leduc, A. B.; Jamison, T. F. Continuous Flow Oxidation of Alcohols and Aldehydes Utilizing Bleach and Catalytic Tetrabutylammonium Bromide. *Org. Process Res. Dev.* **2012**, *16*, 1082–1089.
(61) Zhou, S.; Johnson, M.; Veinot, J. G. C. Iron/Iron Oxide Nanoparticles: A Versatile Support for Catalytic Metals and Their Application in Suzuki–Miyaura Cross-Coupling Reactions. *Chem. Commun.* **2010**, *46*, 2411–2413.
(62) Hyder, Z.; Ruan, J.; Xiao, J. Hydrogen-Bond-Directed Catalysis: Faster, Regioselective and Cleaner Heck Arylation of Electron-Rich Olefins in Alcohols. *Chem. Eur. J.* **2008**, *14*, 5555–5566.
(63) Moriyama, K.; Takemura, M.; Togo, H. Direct and Selective Benzylic Oxidation of Alkylarenes via C–H Abstraction Using Alkali Metal Bromides. *Org. Lett.* **2012**, *14*, 2414–2417.
(64) Mizuta, S.; Stenhagen, I. S. R.; O'Duill, M.; Wolstenhulme, J.; Kirjavainen, A. K.; Forsback, S. J.; Tredwell, M.; Sandford, G.; Moore, P. R.; Huiban, M.; Luthra, S. K.; Passchier, J.; Solin, O.; Gouverneur, V. Catalytic Decarboxylative Fluorination for the Synthesis of Tri- and Difluoromethyl Arenes. *Org. Lett.* **2013**, *15*, 2648–2651.
(65) Muthaiah, S.; Hong, S. H. Acceptorless and Base-Free Dehydrogenation of Alcohols and Amines Using Ruthenium-Hydride Complexes. *Adv. Synth. Catal.* **2012**, *354*, 3045–3053.
(66) Liu, B.; Jin, F.; Wang, T.; Yuan, X.; Han, W. Wacker-Type Oxidation Using an Iron Catalyst and Ambient Air: Application to Late-Stage Oxidation of Complex Molecules. *Angew. Chem. Int. Ed.* **2017**, *56*, 12712–12717.

(67) Deng, Y.; Wei, X.; Wang, H.; Sun, Y.; Noël, T.; Wang, X. Disulfide-Catalyzed Visible-Light-Mediated Oxidative Cleavage of C=C Bonds and Evidence of an Olefin–Disulfide Charge-Transfer Complex. *Angew. Chem. Int. Ed.* **2017**, *56*, 832–836.

(68) Friis, S. D.; Andersen, T. L.; Skrydstrup, T. Palladium-Catalyzed Synthesis of Aromatic Carboxylic Acids with Silacarboxylic Acids. *Org. Lett.* **2013**, *15*, 1378–1381.

(69) Chen, Y.-X.; He, J.-T.; Wu, M.-C.; Liu, Z.-L.; Tang, K.; Xia, P.-J.; Chen, K.; Xiang, H.-Y.; Chen, X.-Q.; Yang, H. Photochemical Organocatalytic Aerobic Cleavage of C═C Bonds Enabled by Charge-Transfer Complex Formation. *Org. Lett.* **2022**, *24*, 3920–3925.

(70) Sherwood, T. C.; Xiao, H.-Y.; Bhaskar, R. G.; Simmons, E. M.; Zaretsky, S.; Rauch, M. P.; Knowles, R. R.; Dhar, T. G. M. Decarboxylative Intramolecular Arene Alkylation Using *N* -(Acyloxy)Phthalimides, an Organic Photocatalyst, and Visible Light. *J. Org. Chem.* **2019**, *84*, 8360–8379.

(71) Nikitas, N. F.; Tzaras, D. I.; Triandafillidi, I.; Kokotos, C. G. Photochemical Oxidation of Benzylic Primary and Secondary Alcohols Utilizing Air as the Oxidant. *Green Chem.* **2020**, *22*, 471–477.

(72) Cutulic, S. P. Y.; Findlay, N. J.; Zhou, S.-Z.; Chrystal, E. J. T.; Murphy, J. A. Metal-Free Reductive Cleavage of C−O σ-Bonds in Acyloin Derivatives by an Organic Neutral Super-Electron-Donor. *J. Org. Chem.* **2009**, *74*, 8713–8718.

(73) Okuyama, T.; Tani, Y.; Miyake, K.; Yokoyama, Y. Chiral Helicenoid Diarylethene with Large Change in Specific Optical Rotation by Photochromism. *J. Org. Chem.* **2007**, *72*, 1634–1638.

(74) Kuwano, R.; Kashiwabara, M.; Sato, K.; Ito, T.; Kaneda, K.; Ito, Y. Catalytic Asymmetric Hydrogenation of Indoles Using a Rhodium Complex with a Chiral Bisphosphine Ligand PhTRAP. *Tetrahedron: Asymmetry* **2006**, *17*, 521–535.

(75) Bieniek, M.; Bujok, R.; Milewski, M.; Arlt, D.; Kajetanowicz, A.; Grela, K. Making the Family Portrait Complete: Synthesis of Electron Withdrawing Group Activated Hoveyda-Grubbs Catalysts Bearing Sulfone and Ketone Functionalities. *J. Organomet. Chem.* **2020**, *918*, 121276.

(76) Cheung, W.-H.; Zheng, S.-L.; Yu, W.-Y.; Zhou, G.-C.; Che, C.-M. Ruthenium Porphyrin Catalyzed Intramolecular Carbenoid C−H Insertion. Stereoselective Synthesis of Cis-Disubstituted Oxygen and Nitrogen Heterocycles. *Org. Lett.* **2003**, *5*, 2535–2538.

(77) Srinivasan, R.; Uttamchandani, M.; Yao, S. Q. Rapid Assembly and in Situ Screening of Bidentate Inhibitors of Protein Tyrosine Phosphatases. *Org. Lett.* **2006**, *8*, 713–716.

(78) Xu, C.; Du, W.; Zeng, Y.; Dai, B.; Guo, H. Reactivity Switch Enabled by Counterion: Highly Chemoselective Dimerization and Hydration of Terminal Alkynes. *Org. Lett.* **2014**, *16*, 948–951.

(79) Puls, F.; Knölker, H.-J. Conversion of Olefins into Ketones by an Iron-Catalyzed Wacker-Type Oxidation Using Oxygen as the Sole Oxidant. *Angew. Chem. Int. Ed.* **2018**, *57*, 1222–1226.

(80) Cardoso, J. M. S.; Lopes, R.; Royo, B. Dehydrogenative Silylation of Alcohols Catalysed by Half-Sandwich Iron N-Heterocyclic Carbene Complexes. *J. Organomet. Chem.* **2015**, *775*, 173–177.

(81) Huang, G.; Ke, M.; Tao, Y.; Chen, F. Specific *Z* -Selectivity in the Oxidative Isomerization of Allyl Ethers to Generate Geometrically Defined *Z*-Enol Ethers Using a Cobalt(II)(Salen) Complex Catalyst. *J. Org. Chem.* **2020**, *85*, 5321–5329.

(82) Marinescu, S. C.; Winkler, J. R.; Gray, H. B. Molecular Mechanisms of Cobalt-Catalyzed Hydrogen Evolution. *Proc. Natl. Acad. Sci. U.S.A.* **2012**, *109*, 15127–15131.
